# Genetic Factors for Coronary Heart Disease and Their Mechanisms: A Meta-Analysis and Comprehensive Review of Common Variants from Genome-Wide Association Studies

**DOI:** 10.3390/diagnostics12102561

**Published:** 2022-10-21

**Authors:** Khairul Anwar Zarkasi, Noraidatulakma Abdullah, Nor Azian Abdul Murad, Norfazilah Ahmad, Rahman Jamal

**Affiliations:** 1 UKM Medical Molecular Biology Institute (UMBI), Universiti Kebangsaan Malaysia (UKM), Kuala Lumpur 56000, Malaysia; 2 Biochemistry Unit, Faculty of Medicine and Defence Health, Universiti Pertahanan Nasional Malaysia (UPNM), Kuala Lumpur 57000, Malaysia; 3 Faculty of Health Sciences, Universiti Kebangsaan Malaysia (UKM), Kuala Lumpur 50300, Malaysia; 4 Epidemiology and Statistics Unit, Department of Community Health, Faculty of Medicine, Universiti Kebangsaan Malaysia (UKM), Kuala Lumpur 56000, Malaysia

**Keywords:** common variants, coronary heart disease, genome-wide association study, meta-analysis

## Abstract

Genome-wide association studies (GWAS) have discovered 163 loci related to coronary heart disease (CHD). Most GWAS have emphasized pathways related to single-nucleotide polymorphisms (SNPs) that reached genome-wide significance in their reports, while identification of CHD pathways based on the combination of all published GWAS involving various ethnicities has yet to be performed. We conducted a systematic search for articles with comprehensive GWAS data in the GWAS Catalog and PubMed, followed by a meta-analysis of the top recurring SNPs from ≥2 different articles using random or fixed-effect models according to Cochran Q and *I*^2^ statistics, and pathway enrichment analysis. Meta-analyses showed significance for 265 of 309 recurring SNPs. Enrichment analysis returned 107 significant pathways, including lipoprotein and lipid metabolisms (rs7412, rs6511720, rs11591147, rs1412444, rs11172113, rs11057830, rs4299376), atherogenesis (rs7500448, rs6504218, rs3918226, rs7623687), shared cardiovascular pathways (rs72689147, rs1800449, rs7568458), diabetes-related pathways (rs200787930, rs12146487, rs6129767), hepatitis C virus infection/hepatocellular carcinoma (rs73045269/rs8108632, rs56062135, rs188378669, rs4845625, rs11838776), and miR-29b-3p pathways (rs116843064, rs11617955, rs146092501, rs11838776, rs73045269/rs8108632). In this meta-analysis, the identification of various genetic factors and their associated pathways associated with CHD denotes the complexity of the disease. This provides an opportunity for the future development of novel CHD genetic risk scores relevant to personalized and precision medicine.

## 1. Introduction

Coronary heart disease (CHD) is a leading cause of mortality, with almost nine million deaths reported annually worldwide [1]. Also known as coronary artery disease (CAD) or ischemic heart disease (IHD), the CHD spectrum consists of stable angina, unstable angina, ST-segment elevation myocardial infarction (STEMI), and non-ST-segment elevation myocardial infarction (non-STEMI), with the latter three conditions being collectively termed acute coronary syndrome (ACS) [2]. The most common pathophysiology for CHD is atherosclerotic plaque build-up in the coronary arterial lumen [3]. Various modifiable and non-modifiable risk factors can influence the formation of atherosclerotic plaque, including age, gender, smoking habit, intake of alcohol, physical inactivity, overweight/obesity, abnormal lipid profile, high blood pressure, as well as family history of CHD [4]. Additionally, the disease has also been linked to various genetic factors that explain at least 30–40% of CHD susceptibility [5].

Previously, genetic causes of CHD were only attributed to rare mutations of single causal genes that involved the lipid and lipoprotein metabolisms resulting in severe hypercholesterolemia. These include 5-kb deletion of *LDLR* gene resulting in truncated low-density lipoprotein (LDL) receptor, missense mutation of *APOB* gene, which decreases the apolipoprotein B100 (apo-B100) protein affinity toward LDLR, or loss-of-function mutation of cholesterol transporters ATP-binding cassette G5 (*ABCG5*) and *ABCG8* genes [6]. Formerly, studies of genetic causes for CHD have often been performed using candidate genes or genetic linkage studies that had DNA coverage limitations [7]. Since 2007, several researchers have performed genome-wide association studies (GWAS) to identify other genetic risks for CHD. GWAS is a study approach that utilizes microarray technologies to interrogate thousands of known variants or single-nucleotide polymorphisms (SNPs) associated with the disease. This approach supersedes other traditional methods since GWAS does not require an a priori hypothesis, at least in terms of which genes are involved [8]. Additionally, GWAS prevents missing potential causative variants as well as unbiased gene selection as seen in candidate gene studies [9], enabling the discovery of previously unknown CHD pathways. The prediction of an individual’s risk for certain diseases using genetic risk factors, as well as the identification of pathobiological mechanisms contributing to disease susceptibility to develop new therapeutic approaches, are the ultimate goals of GWAS [10].

Currently, genetic assessment via GWAS has identified approximately 163 loci related to CHD [9]. The associated genes take part in various processes, such as glutamic acid metabolism by glutamate-ammonia ligase (GLUL), that could potentially lower the natural antioxidant glutathione and its γ-glutamylcysteine precursor, leading to oxidative stress [11,12]. Additionally, GWAS has identified thrombospondin type-1 domain-containing protein-7A (*THSD7A*) loci that might influence adhesion molecule expression due to actin cytoskeleton rearrangements as well as endothelial cell (EC) migration [13]. Certainly, these findings expanded the plausible explanations beyond the previous knowledge regarding the disease mechanisms. Most GWAS publications emphasize novel or replicated SNPs that reach genome-wide significance with a *p*-value of <5.0 × 10^−8^ while other SNPs have remained less highlighted [11,14,15,16]. Although the significance threshold is widely used in GWAS publications to correct for multiple testing [17], it focuses on potential CHD mechanisms based on single SNPs rather than the collective presence of several SNPs constituting the pathways. Furthermore, each GWAS has usually been performed on a large cohort of a single homogenous population, such as Chinese [13], Caucasians [18], or Japanese [19], while the CHD-associated pathways based on the combination of all published GWAS involving various ethnic backgrounds have yet to be determined. Therefore, this paper aimed to systematically assemble all top SNPs reported in the main and Supplementary data of the GWAS publications, examine their overall effect sizes, and identify the related CHD pathways via enrichment analysis.

## 2. Materials and Methods

### 2.1. Search Strategy and Selection Criteria

The GWAS Catalog v1.0.3 was downloaded on 20 November 2020 from https://www.ebi.ac.uk/gwas/, while a parallel online search was performed on the PubMed (https://pubmed.ncbi.nlm.nih.gov/, accessed on 22 November 2020) database with an upper limit of publication date filter applied. Potential papers were identified using the keyword combinations “genome-wide”, “GWAS”, “coronary heart disease”, “coronary artery disease”, “ischemic heart disease”, “myocardial infarction”, “atherosclerosis”, and “acute coronary syndrome”. Relevant citations from the reference section of the retrieved articles were also reviewed.

All discovery and validation GWAS, including original articles, letters, and brief/short communications with comprehensive GWAS data as well as written in English, were included in this review. The outcome of interest is CHD status, stent thrombosis, major adverse cardiovascular events in CHD, as well as clinical and subclinical coronary atherosclerosis. Meanwhile, the definition of CHD included CAD, IHD, myocardial infarction (MI), coronary atherosclerosis, and ACS. Papers that performed Mendelian randomization studies without the GWAS components, haplotypic analyses, candidate gene studies, longitudinal GWAS, commentaries, or review papers including narrative, systematic, and scoping reviews were excluded. As we only focused on the binary outcome, articles with continuous outcomes such as continuous measures of clinical or subclinical coronary atherosclerosis were excluded as well.

### 2.2. Articles Screening for Eligibility

**Figure 1:**
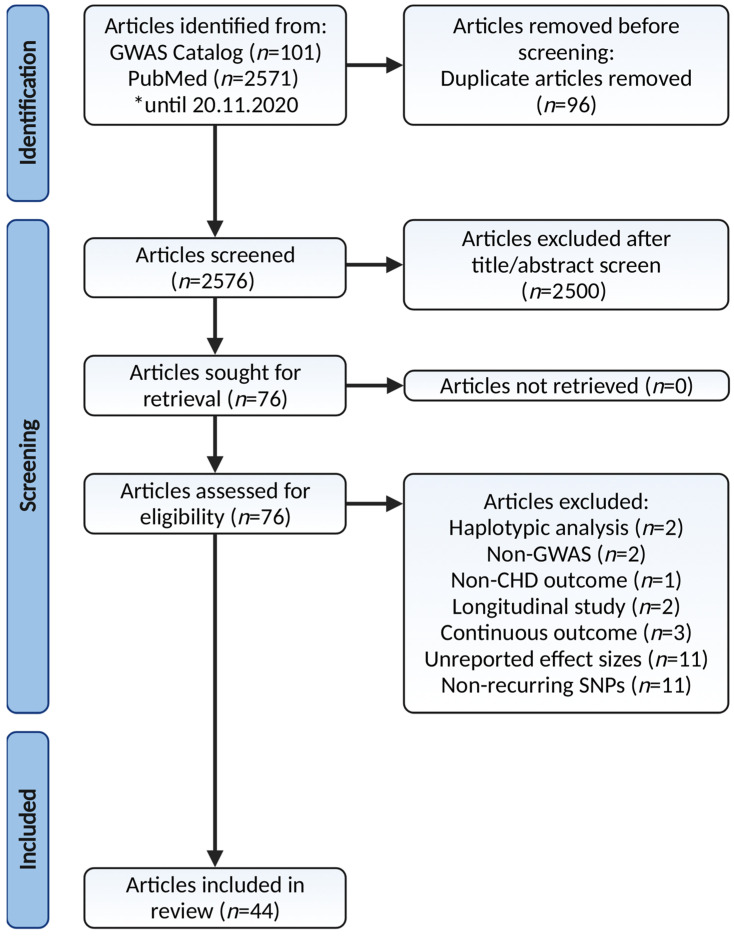
Flowchart of the article selection process. * The list of publications in PubMed was limited until 20 November 2020, similar to the download date of the GWAS Catalog v1.0.3.

Article screening was performed by two authors (K.A.Z. and N.A.) independently. The list of included articles after completing each stage of identification, screening, and inclusion was compared and any discrepancy was resolved by consensus. An online literature search returned 2672 articles and 2576 of them were available after duplicate removal. A further 2500 articles were excluded as the titles and abstracts were irrelevant to the current paper, they were written in a non-English language, or they were animal experimental studies, commentaries, case reports, narrative reviews, or meta-analyses of candidate genes, leaving 76 articles eligible for full-text assessment. Another five articles were excluded as the studies employed a non-GWAS approach, outcomes other than CHD, or studies focusing only on haplotype analysis rather than assessing the SNPs association with CHD individually. An additional 16 articles were further removed due to their longitudinal study approach, continuous outcomes, and unreported effect sizes. Data extraction from the remaining 55 articles produced 14,553 rows of SNPs (data not shown). Two authors (K.A.Z. and N.A.) entered these data into two separate spreadsheets independently, and any discrepancy was resolved by consensus. Of these, another 11 articles were further excluded as they contained non-recurring SNPs. Finally, 44 articles were included in the final review and meta-analysis. The complete flow of the article selection process is depicted in Figure 1 according to the latest Preferred Reporting Items for Systematic Reviews and Meta-Analyses (PRISMA) guidelines [20].

### 2.3. Quality Assessment

Quality assessment was performed according to the Newcastle–Ottawa Quality Assessment Form for Case–Control Studies [21]. Parameters of interest were generally divided into three subheadings comprising selection, comparability, and outcome/exposure. Two authors (K.A.Z. and N.A.) independently scored each of the 44 included papers and the average was taken as the final score. The papers’ overall quality was marked as low, moderate, or high if their final scores were between 0–3, 4–6, or 7–9, respectively [22].

### 2.4. Data Extraction

The data extracted from the selected articles were as follows: (1) study title, author, and publication year; (2) study population; (3) total sample size; (4) study design; (5) disease outcome; and (6) list of SNPs, effect alleles, along with their effect sizes. All effect sizes were standardized in the form of odds ratio (OR) and 95% confidence interval (CI).

### 2.5. Statistical Analysis

The OR and 95% CI of the SNPs that appeared in at least two different articles were transformed into their corresponding log(OR) and standard error (SE), followed by meta-analysis using the Generic Inverse Variance Method in MedCalc^®^ v20 software. Heterogeneity among studies was assessed by Cochran Q and *I*^2^ statistics. Q-statistics with *p* < 0.10 or *I*^2^ > 50% indicated significant heterogeneity among studies; hence, the random effect model was used instead of a fixed-effect model to generate the pooled effect sizes [23]. The meta-analysis *p*-value of <0.05 was set as the significance threshold. Similar analyses were performed using Review Manager v5.4 to confirm the results based on the aforementioned criteria as well as to generate the forest and funnel plots.

### 2.6. Publication Bias

Potential publication bias was determined using Egger’s and Begg’s tests following cut-off *p*-values of <0.05. Significant results for any of the tests would indicate publication bias only if the pooled effect sizes comprised ≥10 GWAS publications [24,25]. For pooled effect sizes generated from <10 studies, publication bias was indicated when the *p*-values were significant for both tests. Since Egger’s test has tendencies for false-positivity and is more recommended for studies with continuous outcomes, while Begg’s test does not consider inter-study heterogeneity and is less accurate for a small number of studies [26,27,28], we combined the results from these two tests to complement their limitations. Any publication bias suggested by these statistical tests was confirmed by manually inspecting the funnel plot.

### 2.7. Pathway Enrichment Analysis

A list of significant SNPs produced following meta-analysis was entered into the Functional Profiling module in the g:Profiler website (https://biit.cs.ut.ee/gprofiler/gost, accessed on 17 June 2021) for pathway enrichment analysis. The settings used were as follows: (1) Organism: *Homo sapiens*; (2) Statistical domain scope: Only annotated genes; (3) Significant threshold: g:SCS threshold algorithm; (4) User threshold: 0.05; and (5) Numeric IDs treated as: ENTREZGENE_ACC. We selected all data sources available in the tools including the Gene Ontology for Molecular Function (GO:MF), Cellular Component (GO:CC), and Biological Process (GO:BP); Kyoto Encyclopedia of Genes and Genomes (KEGG); Reactome (REAC); WikiPathways (WP); Transcription Factor Database (TRANSFAC); microRNA-Target Interaction Database (miRTarBase); Human Protein Atlas (HPA); The Comprehensive Resource of Mammalian Protein Complexes (CORUM); as well as Human Phenotype (HP) Ontology.

## 3. Results

All articles included in this paper are listed in Table 1. The majority of these papers (77.3%) performed multi-stage GWAS discovery and replication, seven studies performed single-stage GWAS (15.9%), two papers performed GWAS focusing only on protein-coding exomes (4.5%), while one paper performed GWAS with secondary data analysis (2.3%). Overall, Caucasians represented the largest population in all CHD GWAS (also termed as Whites, non-Hispanic Whites, and of European descent), followed by East Asian ancestry (i.e., Chinese, Japanese, and Koreans), as well as Blacks (i.e., African Americans and British Blacks); whereas South Asian ancestry (i.e., Pakistanis and Indians), Saudi Arabs, Lebanese, and Malays were the minority populations. Meanwhile, some of the papers recruited trans-ethnic international participants with mixed ancestry backgrounds from various countries.

The results of the quality assessment according to the reviewers’ judgment were summarized in Table 2. All included papers were published in reputable journals with good impact factors. As such, 42 of 44 papers were of high quality with a total score between 7–9. The remaining two papers had a score of six, hence, they were marked as moderate quality. The selection of control subjects from hospital settings and not community-based, as well as the lack of information regarding the control of confounders either in the design or during statistical analysis were the main contributors to the reduced quality of the two papers.

Initially, the 44 final articles generated 1091 rows of data, corresponding to 309 recurring SNPs (Tables S1 and S2). After performing a meta-analysis, 265 SNPs achieved statistical significance with *p* < 0.05. The SNPs ID, effect allele, heterogeneity test results (i.e., *I*^2^ and Q-statistic *p*-value), models used, pooled effect sizes, and their corresponding gene names can be identified in the Supplementary data (Tables S2 and S3). The rs2074356 (*HECTD4*), rs671 (*ALDH2*), rs2596548, rs12229654, and rs11823828 (*TRIM5*, *OR52E4*) were top-five SNPs having the largest positive effect sizes, with their corresponding pooled ORs ranged from 1.29 to 1.48 (*p* < 0.05) (Table 3). The top five SNPs with the largest negative effect sizes include rs146092501 (*COL6A3*), rs61734696 (*MARCHF1*, *ANP32C*), rs115287176 (*TMOD4*), rs146879198 (*ZNF77*), and rs188378669 (*CXCL8*), all of them had pooled ORs of 0.16 (*p* < 0.05). Meanwhile, rs11206510, rs11556924 (*ZC3HC1*), rs6725887 (*WDR12*), rs9349379 (*PHACTR1*), and rs4977574 (*CDKN2B*-AS1) were the top five most recurring SNPs during meta-analysis with pooled ORs ranged from 1.03 to 1.07 (*p* < 0.05) (Table 3). Forest plots for all 309 meta-analyzed SNPs are shown in Figure S1. Since the GWAS was performed on populations with ethnically diverse backgrounds, the effects of certain SNPs towards CHD were highly heterogenous, as shown in the Cochran Q and *I*^2^ statistics, especially those that recurred in a larger number of studies (Table S2).

Out of the 265 significant SNPs, only rs4593108 (OR: 1.03, 95% CI: 1.02–1.03, *p* < 0.0001) showed evidence of publication bias with significant results on both the Egger’s and Begg’s tests (*p* = 0.0159 and 0.0441, respectively), as well as a skewed funnel plot (Table S2, Figure S2). This variant appeared in six different GWAS publications, of which five studies reported positive associations with CHD (OR: 1.01–1.07) and one study mentioned the opposite (OR: 0.93) (Table S1). Upon removal of the latter, publication bias for rs4593108 disappeared (Egger’s *p* = 0.0875, Begg’s *p* = 0.1736) while the effect size and meta-analysis *p*-value remained unchanged (Figure S2). For the rest, 130 SNPs that appeared in <10 studies had significant *p*-values only in Egger’s but not Begg’s test, while another 134 SNPs had *p* > 0.05 in both tests, indicating no publication bias. Funnel plots for all the SNPs are depicted in Figure S3.

**Figure 2:**
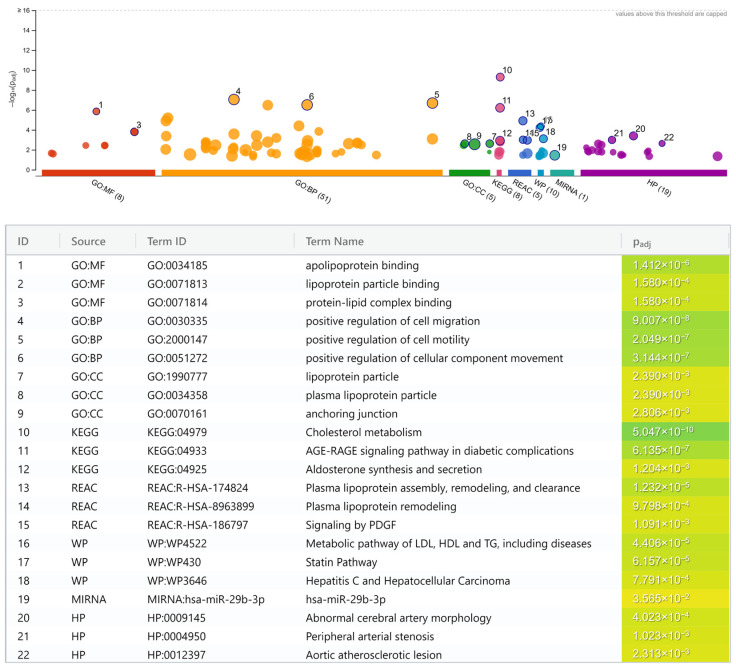
Pathway enrichment analysis using the g:Profiler online tool. The top three significant pathways from each database source were highlighted and labeled numerically. Abbreviations: GO:BP (Gene Ontology:Biological Process); GO:CC (GO:Cellular Component); GO:MF (GO:Molecular Function); HP (Human Phenotype Ontology); KEGG (Kyoto Encyclopedia of Genes and Genome); MIRNA (mirTarBase); REAC (Reactome); WP (WikiPathways).

Using the g:Profiler online tool, pathway enrichment analysis of the 265 SNPs returned 107 significant pathways with *p* < 0.05 (Table S4). The top three results totaling 22 pathways from cross-referencing to each database source are presented in Figure 2, except for TRANSFAC, HPA, and CORUM since they did not return any result. In general, these pathways can be broadly classified into five main groups:

1. Lipoprotein and lipid metabolisms.
  (a) Cholesterol metabolism (KEGG; *p* = 5.0 × 10^−10^).
  (b) Apolipoprotein binding (GO:MF; *p* = 1.4 × 10^−6^).
  (c) Plasma lipoprotein assembly, remodeling, and clearance (REAC; *p* = 1.2 × 10^−5^).
  (d) Metabolic pathway of LDL, high-density lipoprotein (HDL), and triglyceride (TG), including diseases (WP; *p* = 4.4 × 10^−5^).
  (e) Statin pathway (WP; *p* = 6.2 × 10^−5^).
  (f) Lipoprotein particle binding (GO:MF; *p* = 1.6 × 10^−4^).
  (g) Protein-lipid complex binding (GO:MF; *p* = 1.6 × 10^−4^).
  (h) Plasma lipoprotein remodeling (REAC; *p* = 9.8 × 10^−4^).
  (i) Lipoprotein particle (GO:CC; *p* = 2.4 × 10^−3^).
  (j) Plasma lipoprotein particle (GO:CC; *p* = 2.4 × 10^−3^).
2. Atherogenesis.
  (a) Positive regulation of cell migration (GO:BP; *p* = 9.0 × 10^−8^).
  (b) Positive regulation of cell motility (GO:BP; *p* = 2.0 × 10^−7^).
  (c) Positive regulation of cellular component movement (GO:BP; *p* = 3.1 × 10^−7^).
  (d) Anchoring junction (GO:CC; *p* = 2.8 × 10^−3^).
3. Shared cardiovascular pathways.
  (a) Abnormal cerebral artery morphology (HP; *p* = 4.0 × 10^−4^).
  (b) Peripheral arterial stenosis (HP; *p* = 1.0 × 10^−3^).
  (c) Aortic atherosclerotic lesion (HP; *p* = 2.3 × 10^−3^).
4. Diabetes-related pathways.
  (a) Advanced glycation end product (AGE)-receptor for AGE (RAGE) signaling pathway in diabetic complication (KEGG; *p* = 6.1 × 10^−7^).
  (b) Signaling by platelet-derived growth factor (PDGF) (REAC; *p* = 1.1 × 10^−3^).
  (c) Aldosterone synthesis and secretion (KEGG; *p* = 1.2 × 10^−3^).
5. Miscellaneous.
  (a) Hepatitis C virus (HCV)-infection and hepatocellular carcinoma (HCC) pathway (WP; *p* = 7.8 × 10^−4^).
  (b) MiR-29b-3p pathway (miRTarBase; *p* = 3.6 × 10^−2^).

## 4. Discussion and Narrative Synthesis

**Figure 3:**
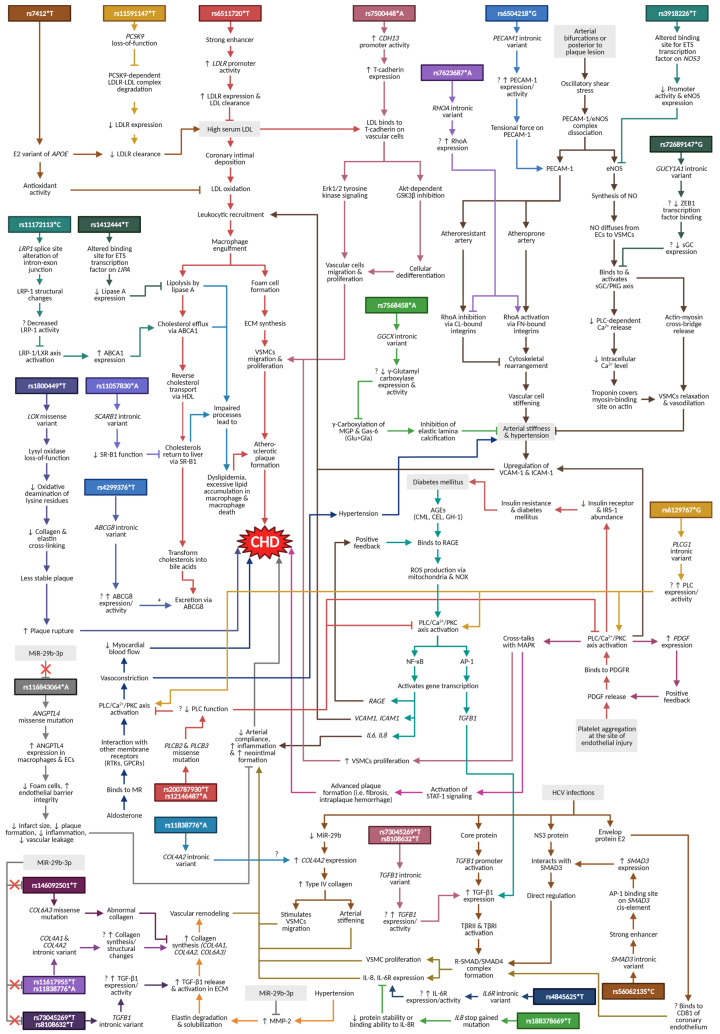
Selected single-nucleotide polymorphisms from pathway enrichment analysis and their proposed mechanisms with either positive or negative associations leading to coronary heart disease.

The lipoprotein and lipid metabolisms (e.g., cholesterol metabolism; plasma lipoprotein assembly, remodeling, and clearance) dominated the enrichment analysis results, indicating that they are the most important mechanisms in CHD pathogenesis. Other pathways, for example, atherogenesis (e.g., positive regulation of cell migration; anchoring junction), shared cardiovascular pathways (e.g., peripheral arterial stenosis; aortic atherosclerotic lesion), as well as diabetes-related pathways (e.g., AGE-RAGE signaling pathway in diabetic complication), are commonly known to be associated with CHD. Interestingly, miscellaneous pathways of the HCV-infection/HCC as well as miR-29b-3p are among the significant results in the pathway enrichment analysis. These two pathways are certainly less publicly known with regard to their association with the disease. Selected SNPs from these pathways and their proposed mechanisms in relation to CHD are presented in Figure 3.

### 4.1. Lipoprotein and Lipid Metabolisms

SNPs that were involved in the lipoprotein and lipid metabolisms include rs7412 (*APOE*: apolipoprotein E), rs6511720 (*LDLR*: LDL receptor), rs11591147 (*PCSK9*: proprotein convertase subtilisin/Kexin type 9), rs1412444 (*LIPA*: lipase A or lysosomal acid lipase), rs11172113 (*LRP1*: LDLR-related protein 1), rs11057830 (*SCARB1*: scavenger receptor class B member 1), and rs4299376 (*ABCG8*: ATP-binding cassette G8) (Table S4).

#### 4.1.1. APOE

Lipid transportation from the liver to the peripheral tissues is initially carried by the very-low-density lipoprotein (VLDL). Lipolysis transforms VLDL to intermediate-density lipoprotein (IDL) and LDL [65], which subsequently returns to the liver for recycling. These lipoproteins contain apolipoprotein E (apo-E), a surface protein that mediates LDL attachment to the LDLR for liver reuptake. Variations in the *APOE* gene give rise to apo-E2, apo-E3, and apo-E4 isoforms. The rs7412*T signifies an apo-E2 isoform that has the lowest LDLR binding affinity compared to others [66]. This could cause delayed LDL clearance and increased serum LDL level [67], increasing the chances for LDL deposition in the coronary intima, thus making the apo-E2 a likely risk factor for CHD. A recent meta-analysis of candidate gene studies seemed to support this theory since rs7412*T was associated with a 1.38-times-higher CHD risk (95% CI: 1.18–1.62) [68]. In contrast, our meta-analysis of GWAS publications yielded the opposite result, wherein the rs7412*T conferred a protective effect against CHD (OR: 0.93, 95% CI: 0.91–0.96) (Table S2). Other than mediating LDL clearance, the apo-E proteins reportedly bound certain metal ions such as ferric, ferrous, cupric, and zinc [69]. These metals imparted direct antioxidant activities to the apo-E proteins with the following strengths: apo-E2 > E3 > E4 [69]. Therefore, we could deduce that despite causing delayed LDL clearance, apo-E2 might be able to prevent oxidation of deposited LDL in the coronary intima with effects large enough to turn apo-E2 into a CHD protective variant instead of a risk factor.

#### 4.1.2. LDLR and PCSK9

Meanwhile, variation of the *LDLR* gene might produce an abnormal receptor that could also contribute to inefficient LDL clearance. *Ldlr* knocked-out gene in animal models exhibited an increased tendency for aortic atherosclerosis as early as three months when fed with a high-fat diet which was closely accompanied by significant dyslipidemia [70]. The common variant rs6511720*T functions as a strong enhancer that increases the *LDLR* promoter activity in the hepatocyte [71]. It has been shown to strongly associate with lower plasma LDL and TC levels (*β =* −0.22 and −0.19, respectively) [71]. Since rs6511720*T was identified to decrease CHD risk in this meta-analysis (OR: 0.95, 95% CI: 0.94–0.95) (Table S2), we hypothesize that the underlying mechanism could be related to improved lipid levels due to increased LDLR expression. The availability of the LDLR on the hepatocyte’s plasma membrane is also regulated by PCSK9, a protein released by the liver that attaches to the LDLR on the hepatocyte surface. It mediates lysosomal degradation of the PCSK9-LDLR-LDL complex resulting in reduced LDLR availability and hampered LDL reuptake [72]. Given its functional importance, targeted PCSK9 inhibition by monoclonal antibodies successfully prevented LDLR degradation [73]. This allows for LDLR recycling with better serum LDL control in individuals at risk [73]. The rs11591147*T is a loss-of-function *PCSK9* variant that has been shown to reduce CHD risk by lowering the lipid risk factors [74]. Our GWAS meta-analysis produces similar results, where *PCSK9* loss-of-function variant rs11591147*T significantly decreases CHD risk by 0.89 times (95% CI: 0.88–0.91) (Table S2).

#### 4.1.3. LIPA and LRP1

Increased circulating LDL that gets deposited within the arterial wall could be modified to form oxidized LDL (oxLDL). To maintain homeostasis, deposited lipids will be transferred back to the liver via reverse cholesterol transport which requires two important processes of lipolysis and cholesterol efflux. In the first process, lipolysis of engulfed native and oxLDL within the macrophage is important to form free cholesterol [75]. It requires lipase A, also known as lysosomal lipase, an enzyme encoded by the *LIPA* gene. Variation of *LIPA*, for example, rs1412444*T alters nucleotide sequence at the transcription factor E-twenty-six (ETS)-binding domain [76]. The nucleotide change was predicted to cause reduced transcriptional factor binding to the gene, subsequently, reducing lipase A expression [76].

In the second process, cholesterol efflux requires ATP-binding cassette A1 (ABCA1), a protein channel that transfers free cholesterol from macrophage to nascent HDL. Increased free cholesterol following lipolysis in the macrophage would stimulate *ABCA1* transcription via liver-X-receptor/retinoid-X-receptor (LXR/RXR) activation [77]. Additionally, a study by Xian et al. (2017) found that phosphorylation of LDL-related protein 1 (LRP-1) on the macrophage surface could also increase ABCA1 protein expression by activating the LRP-1/SHC1/PI3K/AKT/PPAR-γ/LXR axis [78]. The rs11172113*C is a common variant of *LRP1* that could cause splice site alteration in one of the gene’s exon-intron junctions [79]. This could result in LRP-1 structural change [79], which could potentially impair the protein activity. Since our findings showed that both rs1412444*T (*LIPA*) and rs11172113*C (*LRP1*) increased CHD risk 1.02-fold (Table S2), we hypothesize that the underlying mechanisms might be related to reduced lipase A enzyme expression and structural variation of the LRP-1 protein that renders it less functional. These changes could impair both lipolysis and cholesterol efflux processes within the macrophage. Excessive lipid accumulation would then result in macrophage death, which is an important pathological finding in atherosclerotic lesions [78,80].

#### 4.1.4. SCARB1 and ABCG8

Following reverse cholesterol transport, HDL returns the excess cholesterol to the liver via the scavenger receptor class B member 1 (SR-B1), where they are turned into bile acids and excreted through ABCG8 into the intestine [81]. One of the common variants for the *SCARB1* gene that encodes the SR-B1 receptor is rs11057830. Wang and Xu (2019) observed that the levels of serum HDL (*β =* −0.018), LDL (*β =* 0.025), TC (*β =* 0.022), and TG (*β =* 0.022) were altered in individuals harboring rs11057830*A polymorphism [82]. These findings suggest that rs11057830*A might cause reduced SR-B1 function, although the exact mechanism is still unknown as the variant is located in the intron and not the protein-coding sequence [83]. Meanwhile, *ABCG8* deficiency had been shown to cause the failure of biliary cholesterol secretion [84], whereas its overexpression attenuated hypercholesterolemia and decreased atherosclerotic lesions by as much as 70% in susceptible mice [85]. Similar to rs11057830 of *SCARB1*, rs4299376 is an intronic variant of the *ABCG8* gene. Individuals with rs4299376*T had been shown to have more favorable lipid levels, marked by reduced serum LDL (*β =* −2.75), TC (*β =* −3.01), and TG (*β =* −1.08), along with increased serum HDL (*β =* 0.05) [86]. Based on this information, we hypothesized that rs11057830*A is a risk variant, while rs4299376*T is protective against CHD since they had opposite effects on serum lipids. Our meta-analysis showed that rs11057830*A (*SCARB1*) increased CHD risk by 1.03 times (95% CI: 1.03–1.04) while rs4299376*T (*ABCG8*) was associated with a decrease in CHD risk by 0.98 times (95% CI: 0.97–0.98).

### 4.2. Atherogenesis

Dyslipidemia increases the risk of LDL deposition within the arterial wall. Once oxidized, the oxLDL triggers cascades of processes including leukocyte recruitment, foam cell formation, ECM synthesis, and VSMC migration and proliferation in the intimal layer of coronary arteries [87]. As a result, the lipid-containing plaque impedes blood flow to the heart, increasing the risk of myocardial hypoxia. Pathway enrichment analysis in this paper revealed various SNPs that have a role in atherogenesis. These include rs7500448 (*CDH13*: cadherin 13 or T-cadherin), rs6504218 (*PECAM1*: platelet endothelial cell adhesion molecule 1), rs3918226 [*NOS3*: nitric oxide synthase 3 or endothelial NOS (eNOS)], and rs7623687 (*RHOA*: Ras homolog family member A or RhoA) (Table S4).

#### 4.2.1. CDH13

Apart from direct deposition into the intimal layer of the arterial wall, LDL particles can also trigger atherogenesis by binding to T-cadherin on the surface of vascular cells including ECs and VSMCs. This causes intracellular calcium (Ca^2+^) store mobilization via the Erk1/2 tyrosine kinase pathway, resulting in the proliferation and migration of ECs and VSMCs [88,89,90]. Additionally, the proliferative and migratory capacities of vascular cells could be attained by dedifferentiation. This mechanism had been shown in smooth muscle cells (SMCs) overexpressing T-cadherin via the T-cadherin/Akt-dependent GSK3β inhibition [91]. These cells exhibited less differentiated phenotypes, such as spindle morphology loss and fiber disorganization, coupled with a marked increase in their proliferative and migratory capacities [91]. Rs7500448*A is one of the common variants of *CDH13*, a gene that encodes for T-cadherin, which in our meta-analysis exerted 1.03 times (95% CI: 1.02–1.03) increased CHD risk (Table S2). A recent study reported that rs7500448 had a strong co-accessibility with the *CDH13* gene promoter specifically in the artery [92]. This suggests that rs7500448*A could support atherogenesis by increasing *CDH13* gene and protein expression by activating its promoter, which might result in enhanced vascular cell migration and proliferation during the early stages of atherogenesis.

#### 4.2.2. PECAM1, NOS3, and RHOA

Lipid plaque formation usually occurs at the arterial bifurcations where there is oscillating shear stress on the vascular wall due to changes from laminar to turbulent blood flow [93]. Several mechanisms that connect shear stress and atherosclerosis have been described, involving PECAM-1, eNOS, and RhoA. PECAM-1, also known as cluster of differentiation 31 (CD31), is an adhesion molecule located at the inter-endothelial junctions where it forms a complex with eNOS [94]. Shear stress gradient causes transient PECAM-1 dissociation from the complex, which in turn activates nitric oxide (NO) synthesis by eNOS, resulting in vascular dilatation [94,95]. Since endothelial dysfunction marked by impaired vasodilation is a risk for atherosclerosis [96], functional PECAM-1 and eNOS are essential for normal coronary artery physiology and CHD prevention. Concerning this, previous studies observed that ECs lacking PECAM-1 displayed higher basal NO production than cells with normal PECAM-1 expression [97], while dyslipidemic mice with *PECAM1* knocked-out genes suffered less severe atherosclerotic lesions compared to wild-type (WT) controls [98]. Other than promoting vasodilation, NO could also decrease the expression of certain proteins that are important during mitosis [99]. This would result in cell cycle arrest as well as inhibition of VSMC proliferation [99]. On the contrary, lack of NO following eNOS deficiency was associated with marked neointimal hyperplasia and vessel wall thickening [100], providing further evidence of the atheroprotective roles of eNOS.

In an atheroprone artery, shear stress changes would exert tensional force at PECAM-1, which in turn, activate RhoA via fibronectin (FN)-bound integrins resulting in cytoskeleton architectural realignment, as well as ECs stiffening [101,102]. In contrast, the atheroresistant artery exhibited RhoA inhibition via collagen (CL)-bound integrins causing inhibition of cytoskeleton realignment that resulted in more compliance ECs, albeit the response was similarly triggered by PECAM-1 [101]. Other than ECs, the PECAM-1 and RhoA proteins are also expressed by other vascular cells, including VSMCs. Previous works have reported that PECAM-1 is an important feature of differentiated VSMCs [103], whereas the hypercontractility phenotype of differentiated VSMCs usually seen in diabetes and hypertension was correlated with RhoA upregulation [104]. Moreover, spontaneously hypertensive rats did not suffer increased arterial stiffness alone [105]. Additionally, proteomics analysis of their aortic tissues revealed higher expression of RhoA, along with other proteins involved in the actin cytoskeleton organization [105], indicating that arterial stiffness, as well as hypertension, were dependent on a similar mechanism governing cellular stiffening. Vascular stiffening and hypertension had been documented to precede atherosclerotic plaque formation [106]. Endothelial and vascular stiffening had been shown to upregulate intercellular and vascular adhesion molecules 1 (ICAM-1 and VCAM-1), which were influenced by shear stress [107,108]. These would promote leukocyte transendothelial migration during early atherogenesis [87].

Following meta-analysis of CHD GWAS, we discovered that rs3918226*T of *NOS3* (OR: 1.04, 95% CI: 1.03–1.06), rs6504218*G of *PECAM1* (OR: 1.02, 95% CI: 1.01–1.02), and rs7623687*A of *RHOA* (OR: 1.03, 95% CI: 1.02–1.03) were significantly associated with increased CHD risk (Table S2). The common variant rs3918226 is located at the promoter region of the *NOS3* gene, which served as a binding site for the ETS transcription factors [109,110]. Previously, an in vitro study discovered that cells harboring rs3918226*T had decreased *NOS3* promoter activities [110], suggesting that the risk allele could cause suboptimal binding of transcription factors to the DNA that could cause impaired transcription as well as eNOS under-expression. In contrast, the mechanisms on how rs6504218 and rs7623687 might impact the PECAM-1 and RhoA proteins were unknown, since they were intronic variants that were not translated into proteins [111,112]. Hence, further studies are required to unveil their exact mechanisms in elevating the CHD risk.

### 4.3. Shared Cardiovascular Pathways

As discussed previously, eNOS activation by shear stress synthesizes NO, which then diffuses from EC into the underlying VSMC and binds to the heme moiety of soluble guanylate cyclase (sGC) protein [113]. The sGC acts as an NO receptor and is encoded by the guanylate cyclase 1 soluble subunit α1 (*GUCY1A1*) gene, synonymously termed *GUCY1A3*, in humans.

#### 4.3.1. GUCY1A1

Upon interaction with NO, the sGC converts guanosine triphosphate (GTP) to cyclic guanosine monophosphate (cGMP), promoting VSMC relaxation via protein kinase G (PKG) [113]. The VSMC relaxation through PKG is achieved via several mechanisms. First, since VSMC contraction relies on actin–myosin cross-bridging, the PKG stimulates myosin light chain dephosphorylation, resulting in cross-bridge release [114]. Second, Ca^2+^ binding to the troponin is essential to uncover the myosin-binding site on the actin filament, which is normally covered by the troponin, before actin–myosin cross-bridge formation [115]. Hence, PKG would decrease intracellular Ca^2+^ content by inhibiting the L-type Ca^2+^ channel at the plasma membrane, blocking phospholipase C (PLC)-dependent Ca^2+^ release, while initiating Ca^2+^ storage into the sarcoplasmic reticulum [114]. In addition to vasorelaxation, the sGC had been shown to have VSMC anti-migratory and antiproliferation activities which aided in the prevention of neointimal hyperplasia following vascular endothelial injury [116]. Earlier discoveries linked *GUCY1A1* mutation with moyamoya disease, a condition manifested with cerebrovascular angiopathy predisposing to the anterior and middle cerebral artery infarct [117,118]. However, the gene mutation is also related to CHD. The previously discussed molecular roles of sGC were of particular importance since SNP that caused a higher *GUCY1A1* expression had been observed to protect mice against atherosclerosis by reducing VSMC migration and proliferation [119].

The rs7692387 of *GUCY1A1* is an intronic variant with a significant risk for atherosclerosis and CHD [119,120]. In silico analysis by Kessler et al. (2017) found that the variant was a binding site for the zinc finger E box-binding homeobox 1 (ZEB1) transcription factor, which was experimentally confirmed using the HEK293 and human aortic smooth muscle cell lines [119]. Homozygous individuals with rs7692387*A>G variation had a significantly reduced whole blood *GUCY1A1* mRNA and α_1_-sGC protein levels [119], indicating that the rs7692387*G risk allele caused inefficient ZEB1 binding resulting in *GUCY1A1* downregulation. This risk allele also had a negative impact on the NO pathway due to delayed PKG activation [119], a protein that is directly downstream of the sGC. Another intronic variant of *GUCY1A1*, rs72689147*G, had been reported to have a significant association with CHD [120], with an OR of 1.03 (95% CI: 1.02–1.04) in our meta-analysis (Table S2). Although rs72689147 is yet to be functionally characterized, it might alter the sGC activities and the NO pathway in the same manner as rs7692387. This hypothesis is made since rs72689147*G and rs7692387*G risk alleles of *GUCY1A1* are in high linkage disequilibrium (*D*′ = 0.994, *R*^2^ = 0.968, *p* < 0.0001) when examined using the LDpair tool of the LDlink website [121].

#### 4.3.2. LOX

Lysyl oxidase (LOX) catalyzes the oxidative deamination of lysine residues on tropoelastin and tropocollagen to reactive aldehydes allysine. Several allysine residues interact with unaltered lysine, resulting in cross-linking of tropocollagen and tropoelastin fibrils, forming mature elastin and collagen in the extracellular matrix (ECM). Elastin is an interconnected rubbery network that can bend when pressure is exerted, whereas collagen is a much stiffer ECM component. Together, they provide tissue elasticity and tensile strength to the blood vessels. Apart from its role in maintaining ECM stability, LOX dysregulation is involved in every phase of atherosclerosis. Reduced LOX expression was associated with endothelial dysfunction, fatty streak formation, and rupture of the atherosclerotic plaque, whereas increased LOX expression was positively correlated with vascular calcification as well as vascular stiffness [122].

During the oxidative deamination process catalyzed by LOX, reactive oxygen species, particularly hydrogen peroxides (H_2_O_2_), are released as by-products [123]. This can cause oxidative stress with superoxide production as well as p38 mitogen-activated protein kinase (p38-MAPK) pathway activation, resulting in disturbed elastin structure and increased vascular stiffness [123]. Vascular stiffness refers to reduced vascular compliance or altered vascular wall properties. On the other hand, vascular compliance is important in protecting smaller vessels and organs from the damaging effects of pressure generated by the pulsatile movement of the blood [124]. The mechanism of vascular stiffness is partly governed by gradual fracture and loss of elastin fiber, causing intraluminal pressure to be transferred directly to the underlying stiffer collagen [125]. These microscopic changes could be observed in mice with *LOX* deficiency [126]. The stiffening of blood vessels predisposes to hypertension, which precedes CHD occurrence [106]. Increased pressure on the vascular wall in hypertension would cause endothelial injury and trigger inflammation that precipitate lipid deposition and narrowing of the vascular lumen [127]. Therefore, variation in the *LOX* gene might play a role in CHD development by influencing the ECM compositions and vascular stiffness.

In a primate model of myocardial infarction (MI), Xiao et al. (2016) observed a marked increase in *LOX* mRNA and LOX activity, with an accumulation of LOX protein in the myofibroblasts, endothelial cells, and residual cardiomyocytes associated with enhanced collagen type I and III depositions in the infarcted area [128]. Increased LOX expression and activity following MI is an efficient repair mechanism for maintaining myocardial tissue integrity following tissue loss. However, this process is associated with cardiac fibrosis and dysfunction, increasing the patients’ risk for long-term complications including arrhythmias, myocardial stiffness, and cardiomyocyte necrosis, leading to heart failure as well as sudden cardiac death [129]. This evidence indicates that higher LOX expression and activity are unfavorable toward CHD. Other studies have also found increased LOX expression in the atherosclerotic lesion, especially in the fibrous cap and areas of ongoing fibrogenesis which was positively correlated with type I and III collagen expression [130]. However, instead of predisposing to MI, increased LOX expression promoted healing of the atherosclerotic lesion by the formation of a stable fibrous cap associated with significantly lower MI incidence over an eight-year follow-up [130]. These seemingly contradictory effects indicate that increased LOX expression would either be protective or predispose to CHD, depending on its location. Increased LOX expression in the vascular wall and within the atherosclerotic lesion stabilizes the plaque against rupture and subsequent MI, whereas increased LOX expression in the infarcted cardiac area promotes fibrosis and subsequent post-MI sequelae such as cardiac dysfunction and heart failure.

A missense variant involving rs1800449 causes arginine to glutamine or leucine substitution at the 158th amino acid position, producing loss-of-function mutation at the pro-peptide domain of the LOX protein [131]. Unlike the native protein, abnormal LOX due to rs1800449 point mutation failed to inhibit the invasiveness of NF639 breast cancer cells [131]. With regard to CHD, a candidate gene study among the Chinese Han population observed that individuals harboring the rs1800449 risk allele had increased CHD risk by 1.38 times (*p* = 0.001) and 1.93 times (*p* = 0.002) in the allelic and recessive inheritance models, respectively [132]. Previous studies have shown that individuals carrying the rs1800449*T risk allele exhibited higher serum tumor necrosis factor α (TNF-α) and soluble *p*-selectin, protein biomarkers that play a role during the initial phase of atherogenesis involving leukocyte recruitment and extravasation into subendothelial space, respectively [133]. Additionally, patients carrying native rs1800449*C had a 0.18-times-lower degree of atherosclerosis [134]. Indeed, our GWAS meta-analysis results reflected these findings, in which, higher CHD susceptibility was conferred by the rs1800449*T risk variant with an OR of 1.03 (95% CI: 1.02–1.03) (Table S2). We postulate that rs1800449*T might result in loss-of-function mutation and impairment of LOX activity, particularly in the vascular wall. Consequently, it could lead to vascular inflammation, lipid deposition, and atherosclerotic plaque instability, eventually resulting in CHD. The role of the rs1800449 variant is currently being studied extensively in cancers with limited evidence on CHD, providing an opportunity for future exploration.

#### 4.3.3. GGCX

Gamma-glutamyl carboxylase, encoded by the *GGCX* gene, is a protein that catalyzes post-translational modifications of vitamin K-dependent pro-proteins (e.g., matrix GLA proteins (MGP) and growth arrest-specific gene 6 (Gas-6)) into their active forms [135]. These modifications occur at the glutamic acid (Glu) residues which transform them into γ-carboxyglutamic acid (Gla) residues along with oxidation of co-factor vitamin K hydroxyquinone to vitamin K epoxide [136]. One of the earliest reports linked *GGCX* loss-of-function mutations with defective carboxylation of MGP and osteocalcin (OC), both of which were mineralization inhibitors [137]. These mutations involving the *GGCX* gene resulted in ectopic mineralization marked by loss of skin elasticity as well as cerebral artery aneurysms [138]. A systematic review by De Vilder et al. (2017) reported that cardiac manifestations involving the *GGCX* mutations were mostly congenital, such as septal closure defects, persistent ductus arteriosus, and congenital supravalvular pulmonary stenosis [139].

Meanwhile, evidence linking *GGCX* polymorphism with CHD is scarce. In a study by Assimes et al. (2016) using the CardioMetaboChip containing 9087 CHD SNPs among the Taiwanese population, it was observed that rs6738645 of *GGCX* loci had a significant association with CHD (*p* = 6.3 × 10^−5^) [140]. In the same manner, our meta-analysis revealed that an intronic variant of *GGCX* loci, rs7568458*A, was associated with a 1.03-fold increase in CHD risk (95% CI: 1.02–1.03) (Table S2). Animals with functional vitamin K deficiency had increased serum undercarboxylated MGP with overt aortic and cardiac calcification [141]. Apart from that, decreased Gas-6 expression as well as *MGP* deficiency in rodents, which activations depends on vitamin K, were correlated with significant arterial calcifications possibly by affecting vascular ECM architecture in the elastic laminae [142,143]. Based on this information, we deduce that rs7568458*A might cause a decrease in *GGCX* activity, resulting in impaired γ-carboxylation of its substrate pro-proteins MGP and Gas-6. In turn, these inactivated proteins would be unable to inhibit elastic laminae calcification followed by arterial stiffening that led to CHD. Nevertheless, the exact mechanism of rs7568458 on CHD is still unknown.

### 4.4. Diabetes-Related Pathways

The risk of CHD is four times higher among diabetic than non-diabetic individuals [144], which decreases by 23% for each 1.0 mmol/L reduction of fasting blood glucose (FBG) [145]. This indicates that tight glycemic control is essential in T2DM management to prevent CHD complications. Hyperglycemia could accelerate the formation of toxic compounds such as N*ε*-(carboxymethyl)lysine (CML) and N*ε*-(carboxyethyl)lysine (CEL) which are collectively termed advanced glycation end products (AGEs). The interaction of AGE and its receptor, the RAGE, would generate ROS through NADPH oxidase (NOX) and mitochondria [146]. Subsequently, this AGE-RAGE axis would activate nuclear factor kappa-light-chain-enhancer of activated B cell (NF-κB) and AP-1 transcription factors via several pathways notably the Ca^2+^ signaling to activate their target genes expressions [147]. One of the molecules responsible for activating the Ca^2+^ signal is phospholipase C (PLC). Several members of the PLC protein family including PLC-β2, PLC-β3, and PLC-γ1 are involved in the AGE-RAGE axis [147]. These protein isoforms are encoded by their respective genes, namely the *PLCB2*, *PLCB3*, and *PLCG1*.

#### PLCB2, PLCB3, and PLCG1

Upon activation, PLC catalyzes the conversion of phosphoinositol 4,5-bisphosphate (PIP_2_) to diacylglycerol (DAG) and inositol 1,4,5-trisphosphate (IP_3_) [147]. Henceforth, the IP_3_ would increase intracellular Ca^2+^ levels, which in turn activate protein kinase C (PKC) with downstream activation and translocation of NF-κB and AP-1 into the nucleus [147]. Both transcription factors could activate genes related to inflammation (e.g., *IL6*, *IL8*), atherosclerosis (e.g., *VCAM1*, *ICAM1*), and positive feedback of AGE-RAGE axis (i.e., *RAGE*), while AP-1 could additionally induce transcription of the transforming growth factor β1 (*TGFB1*) gene of the TGF-β/SMAD pathway [147]. The AGE-RAGE axis via PLC had been implicated in the development of diabetes and its CHD complication. For instance, diabetic patients had 81%, 100%, and 76% significantly higher levels of CML, CEL, and total AGEs, respectively, compared to non-diabetic controls in either their tissue or serum samples [148,149]. These AGEs were negatively associated with serum insulin levels and pancreatic β-cell function measured by the homeostasis model assessment of β-cell function (HOMA-B) [150]. Meanwhile, CEL and another AGE compound called glyoxal hydroimidazolone (G-H1) were significant predictors for subclinical atherosclerosis severity in a cohort of diabetic patients [151]. Patients with T2DM reportedly had impaired non-coding RNA expressions which were involved in the AGE-RAGE pathway dysregulation via PLC [152]. Similarly, human ECs incubated with AGE not only developed higher levels of inflammatory markers and became less viable, but AGE exposure also led to increased PLC expressions along with intracellular Ca^2+^ concentration [153].

Another signaling pathway that is dependent on PLC is aldosterone, a hormone that is usually known to maintain blood pressure by regulating renal sodium and water retention at the distal tubule of the nephron. Typically, aldosterone would bind to the mineralocorticoid receptor (MR) located in the cytosol to activate gene expression in the so-called genomic actions of aldosterone [154]. Aldosterone–MR complex could also interact with other membrane receptors at caveola such as receptor tyrosine kinases (e.g., epidermal growth factor (EGFR), platelet-derived growth factor (PDGFR)) and G protein-coupled receptors (e.g., angiotensin II receptor 1 (AT1R), G protein-coupled estrogen receptor 1 (GPER1)) to produce non-genomic effects [154]. Interaction between the aldosterone–MR complex with membrane receptors could mediate afferent and efferent arteriolar vasoconstriction in the kidney [155]. These effects were abolished by neomycin and nifedipine drugs, which acted as PLC and Ca^2+^ channel inhibitors, respectively [155], indicating that they were dependent on PLC similar to the AGE-RAGE axis. Apart from the renal vasculature, vascular effects of aldosterone were also seen in the thoracic aorta and coronary artery [156,157]. Selective aldosterone perfusion into the left anterior descending (LAD) coronary artery significantly decreased blood flow and myocardial contractility, which were reversed by a PKC inhibitor GF109203X [157], a pathway that was directly downstream of PLC. Furthermore, aldosterone could contribute to the development of hypertension following diabetes, which serves as one of the CHD risk factors. Hyperglycemic environment activated aldosterone synthase (*CYP11B2*) gene expression in human adrenal H295R cells, coupled with enhanced aldosterone secretion into the media [158]. These changes were also Ca^2+^ dependent, and were significantly inhibited by different types of Ca^2+^-channel blockers [158], which are commonly used clinically as first-line antihypertensive agents.

At the site of vascular injury, aggregation of platelets by adhering to the subendothelial collagen would release platelet-derived growth factor (PDGF), an angiogenic factor that participates in the healing process [159]. Similar to the AGE-RAGE axis and aldosterone signaling, second messengers for the PDGF signaling pathway include the IP_3_ produced by PLC [160]. Interaction between PDGF and its receptor causes PDGFR dimerization and trans-autophosphorylation of its intracellular tyrosine residues [160]. It then recruits the Src protein with subsequent phosphorylation and activation of PLC [160]. Thereafter, PLC could activate PKC with subsequent cross-talk with the MAPK pathway [161]. Apart from its physiological function, pathological roles of PDGF signaling via PLC in diabetes and CHD had been backed by several studies. In the *Apoe* knock-out mice, diabetes induction by streptozotocin (STZ) for 20 weeks resulted in dyslipidemia, elevated pro-inflammatory and pro-sclerotic markers, and 5-fold larger aortic atherosclerotic plaque areas compared to the control [162]. These changes were associated with increased PDGF-β levels and activated PDGFR-β expression in the aorta which was abolished by a tyrosine kinase inhibitor imatinib [162]. Likewise, overexpression of PDGF increased the risk for T2DM, hyperglycemia, as well as insulin resistance by decreasing the insulin receptor and insulin receptor substrate 1 (IRS-1) protein abundance [163]. PDGF-α also appeared to promote its own positive feedback mechanism by increasing the PKC activity downstream to PLC resulting in increased PDGF-α mRNA and protein expression [163]. With regard to CHD, Pang et al. (2020) reported that ACS patients at baseline and after a 3-year follow-up for major adverse cardiovascular and cerebrovascular events (MACCE) exhibited higher levels of PDGF in their peripheral and coronary blood [164]. Both parameters also correlated positively with coronary stenosis severity based on Gensini score (PDGF_peripheral_ *r* = 0.273, *p* = 0.014; PDGF_coronary_ *r* = 0.240, *p* = 0.007) [164]. Meanwhile, signaling by the PDGFR-β activated chemokine secretion with the consecutive accumulation of leukocytes at the aortic media and adventitia [165]. Overexpression of PDGFR-β in hypercholesterolemic mice with *Apoe* and *Ldlr* deficiency not only enhanced atherosclerosis via signal transducer and activator of transcription 1 (STAT-1) signaling, it also increased advanced plaques formation marked by plaque fibrosis and intraplaque hemorrhage [165]. The mechanism by which PDGF increases atherosclerosis via PLC is most likely related to VSMC proliferation. Certainly, the PDGF and Src/PLC/PKC/MAPK signaling pathways has been implicated in the VSMCs proliferation measured by water-soluble tetrazolium 1 (WST-1) cell proliferation assay [161].

In this GWAS meta-analysis, we discovered three SNPs related to PLC that are significantly associated with CHD. These include rs200787930*T of *PLCB2* (OR: 0.16, 95% CI: 0.11–0.22), rs12146487*A of *PLCB3* (OR: 0.98, 95% CI: 0.98–0.99), and rs6129767*G of *PLCG1* (OR: 1.02, 95% CI: 1.01–1.02) (Tables S2 and S4). Both rs200787930 and rs12146487 cause missense mutation to the PLC-β2 and PLC-β3, respectively, while rs6129767 is an intronic variant of the PLC-γ1 [166]. At present, functional studies involving these SNPs are lacking. Perhaps rs200787930*T and rs12146487*A render the PLC-β2 and PLC-β3 protein isoforms less functional and impair their ability to propagate AGE-RAGE, aldosterone, and PDGF signaling. Subsequently, this results in decreased inflammation and atherosclerosis, with ultimate reduction of CHD risk, as shown in our findings. On the other hand, the rs6129767*G intronic variant might increase PLC-γ1 expression or introduce gain-of-function effects causing enhanced PLC signaling that increases the risk for inflammation and atherosclerosis leading to CHD.

### 4.5. HCV Infection/HCC Pathway

Chronic infection with HCV has been shown to pose a significant risk for CHD in the last two decades, although this relationship has been less emphasized, publicly. One of the major earliest studies that confirmed this association was a retrospective cohort study involving 82,083 HCV-infected and 89,582 HCV-uninfected American veterans [167]. The HCV infection significantly increased CHD risk throughout an 8-year follow-up with a hazard ratio (HR) of 1.27 (*p* < 0.001) [167]. Apart from raising the disease risk, HCV infection appeared to influence the number of coronary vessels involved in CHD. In a cross-sectional study among 100 Egyptians undergoing diagnostic coronary angiogram, HCV seropositive patients were highly likely of having two-vessel (OR: 1.34; *p* = 0.02) and three-vessel diseases (OR: 7.30; *p* = 0.008) compared to the HCV seronegative patients [168]. Recent meta-analyses of eight cohort studies and six Case–Control/cross-sectional studies concluded that HCV infection was indeed a significant risk factor for CHD with a risk ratio (RR) of 1.25 and an OR of 1.94, respectively [169]. Although hepatocellular carcinoma (HCC) is a common sequelae of chronic HCV infection, its association with CHD is largely unknown. In this paper, pathway enrichment analysis of significant CHD SNPs also supports HCV infection as one of the significant pathways for CHD. The majority of these SNPs and their corresponding genes related to this pathway are involved in the inflammatory process. They include rs73045269 and rs8108632 (*TGFB1*: transforming growth factor β1), rs56062135 (*SMAD3*: SMAD family member 3), rs188378669 (*CXCL8*: C-X-C motif chemokine ligand 8 or interleukin (IL)-8), as well as rs4845625 (*IL6R*: IL-6 receptor) (Table S4).

#### 4.5.1. TGFB1, SMAD3, IL8, and IL6R

The TGF-β1 is a member of the transforming growth factor superfamily of cytokines. The binding of the TGF-β1 ligand to the TGF-β receptor type II (TβRII) causes phosphorylation and activation of TβRI [170]. The TβRI phosphorylates and activates SMAD2/3, which then associates with SMAD4 to form an R-SMAD/SMAD4 complex [171]. Following translocation into the nucleus, this complex further associates with specific transcriptional factors and co-factors, binds to the SMAD binding element (SBE) on the DNA, and regulates target gene expression [170,171]. Among the many genes under TGF-β/SMAD signaling regulation are IL-8 and IL-6R [172,173].

The HCV core protein directly activates the *TGFB1* promoter and increases gene transcription [174]. Together with the HCV non-structural protein 3 (NS3), the HCV core protein also has direct regulatory action on the TGF-β/SMAD signaling pathway by physically interacting with the SMAD3 protein [175]. Moreover, the serum IL-6R levels were markedly higher among HCV-infected patients with or without HCC compared to the HCV-uninfected controls [176], while the serum IL-8 levels were significantly correlated with hepatitis C viral load [177]. In comparison, serum TGF-β1 and SMAD3 were elevated in CHD patients but not healthy controls [178]; IL-6R expression was increased in the infarcted regions of the MI rat model [179]; whereas CHD patients exhibited similar serum IL-8 levels with chronic HCV infected patients (*p* = 0.22) but they were significantly higher than the healthy participants (*p* = 0.001) [180]. Apart from infecting the liver, HCV via its envelope protein E2 can bind to the CD81 at the extrahepatic sites and induce a strong inflammatory reaction with IL-8 and IL-6 release, as observed in the human thyroid cells [181]. Since CD81 is highly expressed in the human endothelium during early atherosclerosis [182], HCV infection might also be able to cause inflammation in the coronary vessels leading to CHD via the TGF-β/SMAD pathway activation.

Our meta-analysis showed that all the SNPs had significant positive associations with CHD except for IL-8. The ORs for *TGFB1* SNPs were 1.03 for rs73045269*T and 1.02 for rs8108632*T, while the CHD associations were increased 1.03-fold for rs56062135*C (*SMAD3*) and 1.02-fold for rs4845625*T (*IL6R*) (Table S2). The rs73045269*T/rs8108632*T and rs4845625*T are intronic variants that might increase the corresponding *TGFB1* and *IL6R* gene expression [166], although their exact functional impacts are still unknown. Meanwhile, rs56062135*C has a high LD with rs17293632*C (*D*′ = 0.996, *R*^2^ = 0.923, *p* < 0.0001), both of which are intron variants for *SMAD3* [121]. Previously, rs17293632*C in the primary human arterial smooth muscle cells had been shown to be a strong enhancer, functioning as an activator protein 1 (AP-1) transcription factor binding site on *SMAD3* cis-element that increased the gene expression [183]. On the contrary, *IL8* SNP rs188378669*T decreased CHD risk by 0.16-fold based on our meta-analysis. This variant caused stop-gained mutation with decreased IL-8 protein stability [184]. Altered IL-8 structure was hypothesized to affect its IL-8R binding ability resulting in impaired inflammatory processes [184].

#### 4.5.2. COL4A2

Another CHD SNP enriched in the hepatitis C and hepatocellular carcinoma pathway is rs11838776*A (*COL4A2*: type IV collagen α2 chain), which is associated with 1.03 times (95% CI: 1.02–1.04) higher CHD risk in our meta-analysis (Tables S2 and S4). Type IV collagen is responsible for both hepatic fibrotic changes in HCV infection and neointimal formation in CHD [185,186]. Active HCV infection was associated with decreased microRNA-29b (miR-29b) expression, whose target genes include COL4A2, while in contrast, miR-29b overexpression decreased HCV RNA load and extracellular matrix (ECM) collagen expression [187]. Additionally, COL4A2 expression was elevated not only in the liver tissues of preneoplastic and HCC compared to healthy controls [188], but also in the liver tissues of “HCV with HCC” compared to “HCV without HCC” patients [185], as well as in the serum samples of active chronic HCV infection with liver cirrhosis than cirrhosis-free controls [189]. On the other hand, excessive type IV collagen deposition might contribute to arterial stiffening which could decrease arterial compliance [190], while soluble type IV collagen had been observed to stimulate vascular smooth muscle cells (VSMCs) migration in vitro [186], which is an important process in the early phase of atherosclerosis. Based on the given information, it can be hypothesized that hepatic type IV collagen synthesis occurs during HCV infection progression toward fibrosis and HCC, in which some might solubilize in the bloodstream, travel to the heart, and induce arterial stiffness as well as VSMCs migration from tunica media to intima during early atherogenesis leading to CHD. Rs11838776 is an intronic variant of *COL4A2* [166], but its functional effects are unknown. Since rs11838776*A is associated with increased CHD risk, this variant might contribute to CHD via COL4A2 gene overexpression causing accelerated type IV collagen synthesis, arterial stiffness, and neointimal formation.

### 4.6. MiR-29b-3p Pathway

The miR-29b-3p is a short RNA sequence within the miR-29 family encoded by the *MIR29B1* gene. In general, miRNA primary transcript (pri-miRNA) forms a hairpin structure that is further processed into a 70-nt pre-miRNA and subsequent 20-bp double-stranded miRNA by RNase III family members Drosha and Dicer, respectively [191]. One of the two strands, or the mature 20- to 24-nt miRNA, is then incorporated into a miRNA-induced silencing complex (miRISC) [191]. It regulates gene expression by binding to the complementary site on an mRNA, which initiates translational inhibition or mRNA degradation [191]. Apart from ECM production as mentioned previously, several processes known to fall under miR-29 regulation include immune response, cell differentiation, cell proliferation, and apoptosis [192].

#### 4.6.1. ANGPTL4

Pathway enrichment analysis in this paper revealed that miR-29b-3p is a significant pathway for CHD. One of the SNPs enriched in this pathway is rs116843064 (*ANGPTL4*: angiopoietin-like 4) (Table S4). Human ANGPTL4 is an adipokine that has major roles in lipid and glucose metabolism. It is produced mainly by the adipose and liver but can also be found in other tissues including the heart [193]. In mice fed with a high-fat diet, *Angptl4* overexpression alleviated glucose intolerance and insulin resistance by promoting insulin signaling via IRS-1 phosphorylation [194]. However, the beneficial effects of Angptl4 toward glucose metabolism were counteracted by increased hepatic steatosis, altered liver function profile, hypertriglyceridemia, and hypercholesterolemia [194], probably due to accompanying lipogenesis. Additionally, ANGPTL4 could irreversibly induce active lipoprotein lipase (LPL) dimer conversion to inactive LPL monomer [195]. As a result, the LPL activity is inhibited, preventing lipid uptake into peripheral tissues from the circulating lipoproteins [195], potentially contributing to dyslipidemia. A missense variant rs116843064*G>A of the *ANGPTL4* gene had been reported to alter ANGPTL4 protein processing [196]. Inhibition of LPL requires ANGPTL4 protein oligomerization, a process that is lacking in the case of rs116843064*A, causing loss of ANGPTL4 inhibitory effects on LPL [196]. As a consequence, it resulted in decreased serum TG, total cholesterol (TC), and increased serum HDL, along with the prevention of hepatic steatosis [197]. Remarkably, this variant did not alter the ANGPTL4 effects on glucose metabolism as rs116843064*A was also observed to be significantly associated with lower serum FBG, better glucose tolerance following oral glucose tolerance test (OGTT), and improved insulin sensitivity, which was translated to 0.89-fold lower odds of T2DM [197].

With regard to the cardiovascular system, expression of *Angptl4* mRNA and Angptl4 protein in macrophages are closely related to the cells’ exposure to oxLDL [198]; however, whether the protein existed in oligomeric or monomeric forms was unknown. Increased Angptl4 expression inhibited oxLDL uptake by the macrophages, protecting the cells from lipid overload, and suppressing the formation of foam cells [198]. Expression of Angptl4 was also increased in ECs exposed to HDL [199], in which its function appeared to be to preserve normal vascular endothelial barrier integrity by inhibiting vascular endothelial growth factor receptor 2/vascular endothelial cadherin (VEGFR2/VE-cadherin) complex dissociation at the adherens junctions [200]. Collectively, these changes were accompanied by 34% less plaque (*p* < 0.05) in the atherosclerosis-prone *Apoe3*-Leiden mice [198], along with a significant reduction of inflammation, infarct size, and vascular leakage, as well as improved myocardial perfusion in the rat model of ischemia/reperfusion cardiac injury [200]. The *ANGPTL4* gene is under direct regulation by the miR-29b family [201]. MiR-29b-3p has a significant role in CHD since its serum level was two-fold higher in patients with frank CHD compared to healthy subjects (*p* = 0.05) [202], probably by promoting coronary atherosclerosis via *ANGPTL4* silencing in the macrophage causing enhanced monocytic migration and foam cell formation within the affected vessel. A recent in vitro study using cardiomyocyte H9c2 cell lines seemed to support this hypothesis. When exposed to hypoxia/reoxygenation conditions, the H9c2 cells expressed higher levels of miR-29b-3p, together with increased secretion of pro-inflammatory cytokines, including tumor necrosis factor (TNF)-α, IL-1β, and IL-6, as well as decreased cellular survival marked by accelerated apoptosis and impaired proliferation [203].

In summary, native ANGPTL4 plays a protective role in both DM and CHD, as evidenced by improved insulin signaling and glucose metabolism, enhanced vascular endothelial barrier integrity possibly limiting lipid deposition at subendothelial space, as well as reduced oxLDL engulfment by macrophage, preventing foam cell formation. These beneficial effects of ANGPTL4 are much more prominent, despite the accompanying lipogenesis with dyslipidemia and hepatic steatosis. Meanwhile, the missense variant rs116843064*A appeared to reverse the dyslipidemic effect of ANGPTL4 without compromising its protective effects on DM and CHD. A study among 220 T2DM Turkish patients revealed that individuals with rs116843064*A missense variant allele had significantly lower triglyceride levels, BMI values, and obesity percentages, but similar FBG and HbA_1c_ levels compared to rs116843064*G native allele, which was consistent in both CHD and control groups [204]. Further analysis showed that T2DM patients with rs116843064*A were 0.74 times less likely to have CHD compared to those harboring rs116843064*G [204]. These findings were also reflected in our meta-analysis, whereby rs116843064*A conferred a protective effect against CHD with an OR of 0.93 (95% CI: 0.92–0.95) (Table S2), corroborating previous findings from population-based cross-sectional (OR: 0.81) [205], case–control (OR: 0.74) [204], and prospective cohort candidate gene studies (HR: 0.63) [206]. The exact mechanism of how rs116843064*A decreases CHD risk in relation to its silencer miR-29b-3p remains to be elucidated. Perhaps miR-29b-3p fails to recognize and complementarily bind to the *ANGPTL4* mRNA harboring rs116843064*A, resulting in the translational continuation of the mutated protein.

#### 4.6.2. COL4A1 and COL6A3

Several SNPs involved in collagen synthesis are also enriched in the miR-29b-3p pathway, namely rs11617955 (*COL4A1*: collagen type IV α1 chain) and rs146092501 (*COL6A3*: collagen type VI α3 chain), in addition to previously discussed rs73045269/rs8108632 (*TGFB1*), and rs11838776 (*COL4A2*) (Table S4). Coronary heart disease is closely related to vascular wall calcification, particularly in the tunica intima which involved the actions of matrix metalloproteinase 2 (MMP-2). Hypertension could activate vascular MMP-2 expression as seen in angiotensin-II treated mice [207], whereas miR-29b-3p directly targets MMP-2 and regulates its synthesis [208]. There was a negative correlation (*R*^2^ = 0.481, *p* < 0.01) between miR-29b-3p and MMP-2 expression within the calcified rat arteries, while suppression of miR-29b-3p in rat VSMC increased MMP-2 level [208]. This could lead to elastin degradation and solubilization since MMP-2 functions as a gelatinase [209]. Consequently, latent TGF-β1 in the vessel wall ECM would be released and activated, which in turn, stimulated collagen synthesis resulting in vascular remodeling [208]. Apart from targeting *MMP2* [208], the miR-29b family also regulates *TGFB1* [210], as well as collagenous genes *COL4A1*, *COL4A2*, and *COL6A3* [187], which are downstream targets of the TGF-β/SMAD pathway [211,212]. The level of miR-29b-3p was markedly decreased in rats with arterial calcification [208]. Therefore, it is reasonable to deduce that decreased local miR-29b-3p production in the coronary arteries causes vascular remodeling via elastin degradation and new collagen synthesis. This could result in stiffening and decreased coronary arterial compliance, which serves as a precursor to atherogenesis.

The most abundant component of type IV collagen is COL4A1 and COL4A2. During synthesis, they would form a heterotrimeric collagen IV α1α1α2 triple helix, which is then combined with another collagen IV α5α5α6 triple helix to produce a hexamer [213]. Studies have shown that type IV collagen has the ability to regulate the biology of VSMCs, which is important in atherogenesis. Exposure to type IV collagen caused VSMCs to maintain their quiescent and contractility state, as evidenced by increased actin and myosin heavy chain expression as well as reduced expression of pro-inflammatory surface protein VCAM-1 and intracellular signaling pathway NF-κB [214]. Mutated COL4A1 protein failed to be properly extracellularly secreted [215], which could hamper its antiatherogenic activity via VSMCs regulation. Indeed, patients with *COL4A1* gene mutation had reduced type IV collagen abundance, higher rate of VSMC apoptosis, and unstable atherosclerotic plaque formation with thinner fibrous caps [216]. These findings were significantly correlated with higher rates of MI [216].

Meanwhile, collagen type VI is synthesized into α1α2α3 triple helix monomer [217], where the α3(VI) is encoded by the *COL6A3* gene. Type VI collagen has the ability to induce fibroblast differentiation into myofibroblast. Myofibroblasts are a cell type that excessively produces and secretes ECM components while gaining the contractile property of a smooth muscle cell, with an important role in post-MI fibrosis [218]. Type VI collagen can apparently induce atherosclerosis by stimulating the polarization of anti-inflammatory M2 to pro-inflammatory M1 macrophage [219]. The M1 macrophage is responsible for chronic vascular inflammation, monocyte recruitment, and foam cell formation which is associated with plaque progression in atherogenesis [220].

In our meta-analysis, rs73045269*T (OR: 1.03) and rs8108632*T (OR: 1.02) of *TGFB1*, rs11617955*T (OR: 1.04) of *COL4A1*, as well as rs11838776*A (OR: 1.03) of *COL4A2* genes are all intronic variants with unknown effects on the corresponding gene expressions or protein functions in relation to the miR-29b-3p pathway (Table S2) [15,166]. Nevertheless, their positive association with CHD indicates that these variants might be able to evade the inhibitory activities of miR-29b-3p causing *TGFB1*, *COL4A1*, and *COL4A2* gene dysregulations, excessive type VI collagen synthesis, and subsequent vascular remodeling leading to CHD. In contrast, rs146092501*T of *COL6A3* is a missense variant causing glutamine to lysine transition at the 1386th amino acid sequence, which was predicted to be “damaging” or “non-functional” [221]. Perhaps, even if miR-29b-3p fails to silence *COL6A3* mRNA transcript, translation of the mRNA harboring rs146092501*T variant would result in abnormal type VI collagen synthesis which is protective against vascular remodeling by promoting M1 to M2 macrophage polarization, since rs146092501*T was associated with a 0.16 times reduction in CHD risk, as seen in our meta-analysis (Table S2).

## 5. Perspectives and Concluding Remarks

The systematic approach employed in this paper has several advantages. First, it answered a specific question regarding the genetic factors and their molecular mechanisms for CHD, unlike the much broader questions usually imposed by a narrative review. Second, the systematic search in this paper followed a strict methodology and was conducted in reliable databases with pre-determined inclusion and exclusion criteria. Thus, the publications included in this systematic review are of high quality and standards as shown in the evidence table (Table 2). Third, all potential publications have been assessed with regard to their suitability to answer the research question, and publications that fulfilled the inclusion criteria were subjected to data extraction and analysis. This process is critical for limiting bias and producing results that are as accurate as possible. The large number of publications included in this paper reflects the fact that potential publications from GWAS Catalog and PubMed were searched exhaustively and extensively.

There are several limitations to this paper. First, the total number of top SNPs reported in the main and Supplementary data was highly inconsistent among studies, ranging from one to a few hundred. Therefore, the effect sizes of some SNPs might be missed in certain papers or studies, which might affect the final pooled effect sizes following meta-analysis. Second, the proposed mechanisms for selected SNPs were based on a comprehensive review of existing literature since we did not perform any validation study. However, it provides the opportunity for future studies to further validate the SNPs and their corresponding pathways’ pathophysiological roles, especially pathways with sparse evidence such as the miR-29b-3p pathway in relation to the CHD development. Third, the full list of 265 significant SNPs and their pathways has not been tested for direct clinical use, such as in the construction of polygenic risk scores (PRS) for personalized CHD management. Certainly, one of our future aims is to construct a novel PRS based on significant GWAS SNPs and examine its value in predicting CHD in a specific, less commonly studied population cohort. The PRS might be partly contributed by findings reported in this review.

Regardless of the points mentioned above, this paper’s biggest advantage perhaps lies in the combination of genetics association with CHD across various ethnic groups. To the best of our knowledge, this is the first paper to perform a meta-analysis of top variants reported in the main and Supplementary data from all GWAS publications. GWAS publications have usually emphasized SNPs and the corresponding pathways that reached genome-wide significance, while others have remained less highlighted. In this paper, the significant association of these SNPs and their corresponding pathways was examined through meta-analysis when they appeared in at least two publications. Despite the difference in approach, the majority of the CHD pathways identified in this paper are commonly known to be associated with CHD, reflecting their major roles in the disease development.

In summary, a meta-analysis of the top common variants found in published genome-wide association studies identified various genetic factors related to lipoprotein and lipid metabolisms, atherogenesis, shared cardiovascular, diabetes, hepatitis C virus infection/hepatocellular carcinoma, as well as miR-29b-3p pathways significantly associated with higher risk for coronary heart disease. These findings indicate the complexity of the disease and provide an opportunity for the future development of novel CHD genetic risk scores relevant to personalized and precision medicine.

## Figures and Tables

**Table 1:** The list of included publications sorted alphabetically according to the authors’ names.

Author (Year)	Population	Total Sample Size	Study Design	Disease Outcome
Aoki et al. (2011) [14]	Japanese	13,130	GWAS discovery and replication	MI
Burton et al. (2007) [29]	British (Whites)	16,179	GWAS	CAD
Charmet et al. (2018) [30]	European descents	6754	GWAS discovery and replication	CAD in T1DM
Choi et al. (2019) [31]	Koreans	1688	GWAS discovery and replication	SCA
Dichgans et al. (2014) [32]	Mostly (>90%) Caucasians)	109,124	GWAS	CAD
Erdmann et al. (2009) [33]	Germans	40,773	GWAS discovery and replication	CAD/MI
Erdmann et al. (2011) [34]	Germans	19,036	GWAS discovery and replication	CAD/MI
Fall et al. (2018) [18]	British (Whites, Blacks, and Asians); and European Non-British (Whites)	15,666	GWAS with secondary analysis	CAD
Hager et al. (2012) [35]	Lebanese	4741	GWAS discovery and replication	CAD severity on angiogram
Han et al. (2017) [36]	Singaporean Chinese, Malays, and Indians	11,684	GWAS discovery and replication	CAD
Helgadottir et al. (2007) [37]	Americans and Icelanders	17,354	GWAS discovery and replication	CAD/MI
Hirokawa et al. (2015) [19]	Japanese	44,673	GWAS discovery and replication	MI
Hu et al. (2016) [38]	Chinese Han	7097	GWAS discovery and replication	CAD
Kathiresan et al. (2009) [39]	Participants of 15 studies from five countries (mostly Caucasians)	19,444	GWAS discovery and replication	Early-onset MI
Kertai et al. (2015) [40]	Mostly Caucasians	3488	GWAS discovery and replication	Perioperative MI after CABG
Klarin et al. (2017) [41]	European ancestry	425,186	GWAS discovery and replication	CAD
Lee et al. (2013) [42]	Koreans and Japanese	13,742	GWAS discovery and replication	CAD
Lettre et al. (2011) [43]	African Americans	8090	GWAS discovery and replication	CHD
Li et al. (2018) [13]	Chinese Han	21,828	GWAS discovery and replication	CAD
Lu et al. (2012) [44]	Chinese Han	33,466	GWAS discovery and replication	CAD
Matsunaga et al. (2020) [16]	Japanese and Caucasians	392,241	GWAS discovery and replication	CAD
McPherson et al. (2007) [45]	Caucasians	24,425	GWAS discovery and replication	CHD
Nelson et al. (2017) [46]	Caucasians	340,799	GWAS discovery and replication	CAD
Nikpay et al. (2015) [5]	Participants of 40 international cohorts from 20 countries (mostly Caucasians, followed by Blacks, Indian-subcontinent descents, Chinese, and others)	~185,000	GWAS discovery and replication	CAD
O’Donnell et al. (2011) [47]	European descents	15,993	GWAS discovery and replication	SCA/MI
Pechlivanis et al. (2013) [48]	Germans	4329	GWAS	SCA
Peden et al. (2011) [49]	Europeans and South Asians	30,482	GWAS discovery and replication	CAD
Qi et al. (2013) [11]	Americans (Non-Hispanic Whites)	6562	GWAS discovery and replication	CHD in T2DM
Reilly et al. (2011) [50]	European descents	29,203	GWAS discovery and replication	CAD/MI
Samani et al. (2007) [51]	European descents	7383	GWAS discovery and replication	CAD
Schunkert et al. (2011) [52]	European descents	143,677	GWAS discovery and replication	CAD
Siewert and Voight (2018) [53]	Mostly European descents (>80%)	547,261	GWAS	CHD
Takeuchi et al. (2012) [54]	Japanese	13,035	GWAS discovery and replication	CAD
van der Harst and Verweij (2018) [15]	Mixed ancestry	547,261	GWAS discovery and replication	CAD
Verma et al. (2020) [55]	European descents	2750	GWAS	MACE (CVD deaths/MI)
Vujkovic et al. (2020) [56]	Multi-ancestry	1,407,282	GWAS	T2DM and related vascular outcomes including CHD
Wakil et al. (2016) [57]	Saudi Arabs	5668	GWAS discovery and replication	CAD/MI
Wang et al. (2011) [58]	Chinese Han	8053	GWAS discovery and replication	CAD
Wei et al. (2018) [59]	Mostly (97%) Whites	12,052	GWAS discovery and replication	CHD during statin therapy
Wild et al. (2011) [60]	Caucasians	64,820	GWAS discovery and replication	CAD
Winsvold et al. (2017) [61]	Mostly Caucasians	117,477	GWAS	CAD
Yamada et al. (2018a) [62]	Japanese	6926	GWAS (focused on exome)	Early-onset MI
Yamada et al. (2018b) [63]	Japanese	7256	GWAS (focused on exome)	Early-onset CAD
Zhong et al. (2017) [64]	Chinese	299	GWAS discovery and replication	CHD during clopidogreltherapy

Abbreviations: CABG (coronary artery bypass grafting); CAD (coronary artery disease); CHD (coronary heart disease); CVD (cardiovascular disease); GWAS (genome-wide association study); MACE (major adverse cardiovascular events); MI (myocardial infarction); SCA (subclinical coronary atherosclerosis); T1DM (type 1 diabetes mellitus); T2DM (type 2 diabetes mellitus).

**Table 2:** Quality assessment according to the Newcastle–Ottawa Quality Assessment Form for Case–Control Studies.

Author (Year)	1	2	3	4	5	6	7	8	Overall Quality
Aoki et al. (2011) [14]	★	★	★	★	★★	★	★	★	High
Burton et al. (2007) [29]	★	★	★	★		★	★	★	High
Charmet et al. (2018) [30]		★		★	★★	★	★	★	High
Choi et al. (2019) [31]	★	★	★	★	★★	★	★	★	High
Dichgans et al. (2014) [32]	★	★	★	★		★	★	★	High
Erdmann et al. (2009) [33]	★	★	★	★	★★	★	★	★	High
Erdmann et al. (2011) [34]	★	★	★	★	★★	★	★	★	High
Fall et al. (2018) [18]	★	★		★	★★	★	★	★	High
Hager et al. (2012) [35]	★	★		★	★★	★	★	★	High
Han et al. (2017) [36]	★	★	★	★	★★	★	★	★	High
Helgadottir et al. (2007) [37]	★	★		★	★★	★	★	★	High
Hirokawa et al. (2015) [19]	★	★	★	★		★	★	★	High
Hu et al. (2016) [38]	★	★		★	★★	★	★	★	High
Kathiresan et al. (2009) [39]	★	★	★	★	★★	★	★	★	High
Kertai et al. (2015) [40]	★	★		★	★	★	★	★	High
Klarin et al. (2017) [41]	★	★	★	★	★★	★	★	★	High
Lee et al. (2013) [42]	★	★	★		★★	★	★	★	High
Lettre et al. (2011) [43]	★	★	★	★	★★	★	★	★	High
Li et al. (2018) [13]	★	★	★	★	★★	★	★	★	High
Lu et al. (2012) [44]	★	★	★	★	★★	★	★	★	High
Matsunaga et al. (2020) [16]	★	★	★	★	★★	★	★	★	High
McPherson et al. (2007) [45]	★	★	★	★		★	★	★	High
Nelson et al. (2017) [46]	★	★	★	★	★★	★	★	★	High
Nikpay et al. (2015) [5]	★	★	★	★	★★	★	★	★	High
O’Donnell et al. (2011) [47]	★	★	★	★	★★	★	★	★	High
Pechlivanis et al. (2013) [48]	★	★	★	★	★★	★	★	★	High
Peden et al. (2011) [49]	★	★	★	★	★★	★	★	★	High
Qi et al. (2013) [11]	★	★	★	★	★	★	★	★	High
Reilly et al. (2011) [50]	★	★	★	★	★★	★	★	★	High
Samani et al. (2007) [51]	★	★	★	★	★★	★	★	★	High
Schunkert et al. (2011) [52]	★	★	★	★	★★	★	★	★	High
Siewert and Voight (2018) [53]	★	★	★	★		★	★	★	High
Takeuchi et al. (2012) [54]	★	★	★	★	★	★	★	★	High
van der Harst and Verweij (2018) [15]	★	★	★	★	★★	★	★	★	High
Verma et al. (2020) [55]	★	★	★	★	★★	★	★	★	High
Vujkovic et al. (2020) [56]	★	★	★		★★	★	★	★	High
Wakil et al. (2016) [57]	★	★		★		★	★	★	Moderate
Wang et al. (2011) [58]	★	★	★	★	★★	★	★	★	High
Wei et al. (2018) [59]	★	★		★	★★	★	★	★	High
Wild et al. (2011) [60]	★	★	★	★	★★	★	★	★	High
Winsvold et al. (2017) [61]	★	★	★	★	★★	★	★	★	High
Yamada et al. (2018a) [62]	★	★		★	★★	★	★	★	High
Yamada et al. (2018b) [63]	★	★		★	★★	★	★	★	High
Zhong et al. (2017) [64]	★	★		★		★	★	★	Moderate

Quality assessment parameters include selection (1: case definition, 2: representativeness of case, 3: selection of controls, 4: definition of controls); comparability (5: control of confounders); and outcome/exposure (6: ascertainment of exposure, 7: same method of ascertainment for cases and controls, 8: non-response rate). Overall quality is low (0–3), moderate (4–6), or high (7–9) according to the final score. The score for each parameter is indicated by ★.

**Table 3:** Top five single-nucleotide polymorphisms with the largest positive effect sizes, the largest negative effect sizes, and SNPs that recurred the most.

	SNP	Effect Allele	Mapped Gene(s)	No. of Articles	*I* ^2^	Cochran Q *p*-Value	Effect Model	OR (95% CI)	Meta *p*-Value	Egger’s *p*-Value	Begg’s *p*-Value
SNPs with the largest positive effect sizes	rs11823828	G	*TRIM5*, *OR52E4*	2	0.0	0.9538	Fixed	1.48 (1.38–1.58)	<0.0001	<0.0001	0.3173
	rs12229654	G	-	2	43.8	0.1821	Fixed	1.34 (1.22–1.46)	<0.0001	<0.0001	0.3173
	rs2596548	T	-	2	0.0	0.3801	Fixed	1.32 (1.23–1.41)	<0.0001	<0.0001	0.3173
	rs671	A	*ALDH2*	3	91.8	<0.0001	Random	1.31 (1.12–1.54)	0.0007	0.2385	0.1172
	rs2074356	T	*HECTD4*	3	86.3	0.0007	Random	1.29 (1.12–1.49)	0.0004	0.2377	0.1172
SNPs with the largest negative effect sizes	rs146092501	T	*COL6A3*	2	0.0	0.4039	Fixed	0.16 (0.11–0.22)	<0.0001	1.0000	1.0000
	rs61734696	T	*MARCHF1*, *ANP32C*	2	0.0	0.4039	Fixed	0.16 (0.11–0.22)	<0.0001	1.0000	1.0000
	rs115287176	A	*TMOD4*	2	0.0	0.4140	Fixed	0.16 (0.11–0.23)	<0.0001	<0.0001	0.3173
	rs146879198	A	*ZNF77*	2	0.0	0.4140	Fixed	0.16 (0.11–0.23)	<0.0001	<0.0001	0.3173
	rs188378669	T	*CXCL8*	2	0.0	0.3928	Fixed	0.16 (0.11–0.22)	<0.0001	<0.0001	0.3173
The most recurred SNPs	rs4977574	G	*CDKN2B*-AS1	15	98.4	<0.0001	Random	1.07 (1.05–1.09)	<0.0001	0.0961	0.3708
	rs11206510	T	-	13	77.6	<0.0001	Random	1.03 (1.02–1.04)	<0.0001	0.2483	0.5339
	rs9349379	G	*PHACTR1*	12	93.5	<0.0001	Random	1.06 (1.04–1.08)	<0.0001	0.6320	0.8358
	rs11556924	C	*ZC3HC1*	11	79.7	<0.0001	Random	1.03 (1.02–1.04)	<0.0001	0.8441	0.9350
	rs6725887	C	*WDR12*	10	87.4	<0.0001	Random	1.04 (1.02–1.07)	0.0007	0.0807	0.3252

## Data Availability

Data that support the reported results can be found in the GWAS Catalog and PubMed (National Library of Medicine) databases.

## Supplementary Materials

The following supporting information can be downloaded at: https://www.mdpi.com/article/10.3390/diagnostics12102561/s1, Figure S1: Forest plots for all single-nucleotide polymorphisms from 44 GWAS publications included in this paper except for rs4593108; Figure S2: Forest and funnel plots for rs4593108 before and after correction for publication bias; Figure S3: Funnel plots for all single-nucleotide polymorphisms from 44 GWAS publications included in this paper except for rs4593108; Table S1: Recurring single-nucleotide polymorphisms (SNPs) from the 44 included articles; Table S2: Meta-analysis results of the recurring SNPs; Table S3: Significant meta-analyzed SNPs and their corresponding gene(s); Table S4: Results of significant pathways in the Pathway Enrichment Analysis using g:Profiler.
