# Supplementary material for: Genetic Factors for Coronary Heart Disease and Their Mechanisms: A Meta-Analysis and Comprehensive Review of Common Variants from Genome-Wide Association Studies
Source: Diagnostics (Basel). 2022 Oct 21;12(10):2561. doi: 10.3390/diagnostics12102561 (PMC9601486; doi:10.3390/diagnostics12102561)
Supplement: Supplementary file 1 [file diagnostics-12-02561-s001.zip › Figure S1.pdf]

rs10139550

Study or Subgroup	log[Odds Ratio]	SE	Weight	Odds Ratio IV, Fixed, 95% CI
Charmet et al. 2018	-0.018	0.037	0.4%	0.98 [0.91, 1.06]
Nelson et al. 2017	0.021	0.003	63.7%	1.02 [1.02, 1.03]
Nikpay et al. 2015	0.025	0.004	35.8%	1.03 [1.02, 1.03]
**Total (95% CI)**			**100.0%**	**1.02 [1.02, 1.03]**

Heterogeneity: Chi² = 1.83, df = 2 (P = 0.40); I² = 0%
Test for overall effect: Z = 9.30 (P < 0.00001)

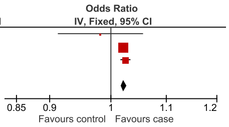

rs10168194

Study or Subgroup	log[Odds Ratio]	SE	Weight	Odds Ratio IV, Fixed, 95% CI
Nelson et al. 2017	0.017	0.004	36.0%	1.02 [1.01, 1.03]
van der Harst et al. 2018	0.012	0.003	64.0%	1.01 [1.01, 1.02]
**Total (95% CI)**			**100.0%**	**1.01 [1.01, 1.02]**

Heterogeneity: Chi² = 1.00, df = 1 (P = 0.32); I² = 0%
Test for overall effect: Z = 5.75 (P < 0.00001)

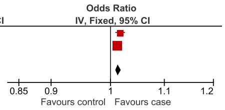

rs10237377

Study or Subgroup	log[Odds Ratio]	SE	Weight	Odds Ratio IV, Fixed, 95% CI
Charmet et al. 2018	-0.041	0.038	0.6%	0.96 [0.89, 1.03]
van der Harst et al. 2018	-0.015	0.003	99.4%	0.99 [0.98, 0.99]
**Total (95% CI)**			**100.0%**	**0.98 [0.98, 0.99]**

Heterogeneity: Chi² = 0.47, df = 1 (P = 0.50); I² = 0%
Test for overall effect: Z = 5.07 (P < 0.00001)

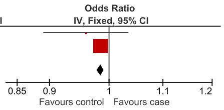

rs10455872

Study or Subgroup	log[Odds Ratio]	SE	Weight	Odds Ratio IV, Random, 95% CI
Nelson et al. 2017	0.117	0.008	59.1%	1.12 [1.11, 1.14]
Wei et al. 2018	0.09	0.014	40.9%	1.09 [1.06, 1.12]
**Total (95% CI)**			**100.0%**	**1.11 [1.08, 1.14]**

Heterogeneity: Tau² = 0.00; Chi² = 2.80, df = 1 (P = 0.09); I² = 64%
Test for overall effect: Z = 7.98 (P < 0.00001)

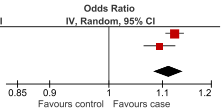

rs1050362

Study or Subgroup	log[Odds Ratio]	SE	Weight	Odds Ratio IV, Fixed, 95% CI
Charmet et al. 2018	0.045	0.037	0.3%	1.05 [0.97, 1.12]
van der Harst et al. 2018	0.015	0.002	99.7%	1.02 [1.01, 1.02]
**Total (95% CI)**			**100.0%**	**1.02 [1.01, 1.02]**

Heterogeneity: Chi² = 0.66, df = 1 (P = 0.42); I² = 0%
Test for overall effect: Z = 7.55 (P < 0.00001)

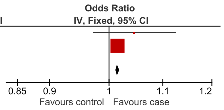

rs10738607

Study or Subgroup	log[Odds Ratio]	SE	Weight	Odds Ratio IV, Random, 95% CI
Han et al. 2017	0.082	0.014	50.3%	1.09 [1.06, 1.12]
Wakil et al. 2016	-0.108	0.02	49.7%	0.90 [0.86, 0.93]
**Total (95% CI)**			**100.0%**	**0.99 [0.82, 1.19]**

Heterogeneity: Tau² = 0.02; Chi² = 60.57, df = 1 (P < 0.00001); I² = 98%
Test for overall effect: Z = 0.13 (P = 0.90)

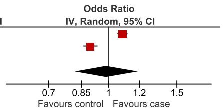

rs10738609

Study or Subgroup	log[Odds Ratio]	SE	Weight	Odds Ratio IV, Fixed, 95% CI
Han et al. 2017	0.088	0.013	65.7%	1.09 [1.06, 1.12]
Lee et al. 2013	0.097	0.018	34.3%	1.10 [1.06, 1.14]
**Total (95% CI)**			**100.0%**	**1.10 [1.07, 1.12]**

Heterogeneity: Chi² = 0.16, df = 1 (P = 0.69); I² = 0%
Test for overall effect: Z = 8.64 (P < 0.00001)

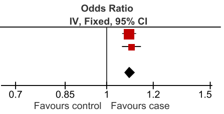

rs10746041

Study or Subgroup	log[Odds Ratio]	SE	Weight	Odds Ratio IV, Random, 95% CI
Aoki et al. 2011	-0.052	0.024	56.6%	0.95 [0.91, 1.00]
Kertai et al. 2015	-0.233	0.07	43.4%	0.79 [0.69, 0.91]
**Total (95% CI)**			**100.0%**	**0.88 [0.74, 1.05]**

Heterogeneity: Tau² = 0.01; Chi² = 5.98, df = 1 (P = 0.01); I² = 83%
Test for overall effect: Z = 1.46 (P = 0.15)

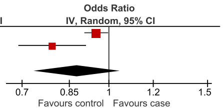

rs10757264

Study or Subgroup	log[Odds Ratio]	SE	Weight	Odds Ratio IV, Fixed, 95% CI
Samani et al. 2007	0.09	0.015	22.1%	1.09 [1.06, 1.13]
Wild et al. 2011	0.072	0.008	77.9%	1.07 [1.06, 1.09]
**Total (95% CI)**			**100.0%**	**1.08 [1.06, 1.09]**

Heterogeneity: Chi² = 1.12, df = 1 (P = 0.29); I² = 11%
Test for overall effect: Z = 10.76 (P < 0.00001)

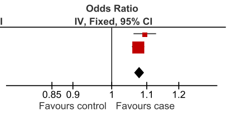

rs10757270

Study or Subgroup	log[Odds Ratio]	SE	Weight	Odds Ratio IV, Fixed, 95% CI
Han et al. 2017	0.078	0.014	42.4%	1.08 [1.05, 1.11]
Wild et al. 2011	0.09	0.012	57.6%	1.09 [1.07, 1.12]
**Total (95% CI)**			**100.0%**	**1.09 [1.07, 1.11]**

Heterogeneity: Chi² = 0.42, df = 1 (P = 0.52); I² = 0%
Test for overall effect: Z = 9.32 (P < 0.00001)

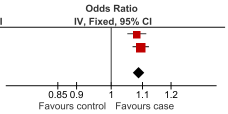

rs10757272

Study or Subgroup	log[Odds Ratio]	SE	Weight	Odds Ratio IV, Random, 95% CI
Aoki et al. 2011	0.056	0.025	20.1%	1.06 [1.01, 1.11]
Choi et al. 2019	0.511	0.095	4.1%	1.67 [1.38, 2.01]
Han et al. 2017	0.08	0.014	25.2%	1.08 [1.05, 1.11]
Lee et al. 2013	0.108	0.018	23.4%	1.11 [1.08, 1.15]
Wild et al. 2011	0.111	0.008	27.3%	1.12 [1.10, 1.14]
**Total (95% CI)**			**100.0%**	**1.11 [1.07, 1.16]**

Heterogeneity: Tau² = 0.00; Chi² = 25.75, df = 4 (P < 0.0001); I² = 84%
Test for overall effect: Z = 5.21 (P < 0.00001)

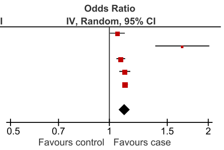

rs10757274

Study or Subgroup	log[Odds Ratio]	SE	Weight	Odds Ratio IV, Random, 95% CI
Han et al. 2017	0.086	0.014	19.8%	1.09 [1.06, 1.12]
Lu et al. 2012	0.137	0.01	20.3%	1.15 [1.12, 1.17]
Matsunaga et al. 2020	0.086	0.008	20.4%	1.09 [1.07, 1.11]
McPherson et al. 2007	0.105	0.011	20.2%	1.11 [1.09, 1.13]
Wakil et al. 2016	-0.102	0.018	19.3%	0.90 [0.87, 0.94]
**Total (95% CI)**			**100.0%**	**1.07 [1.01, 1.13]**

Heterogeneity: Tau² = 0.00; Chi² = 137.90, df = 4 (P < 0.00001); I² = 97%
Test for overall effect: Z = 2.15 (P = 0.03)

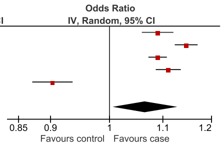

rs10757275

Study or Subgroup	log[Odds Ratio]	SE	Weight	Odds Ratio IV, Fixed, 95% CI
Han et al. 2017	0.086	0.014	59.6%	1.09 [1.06, 1.12]
Lee et al. 2013	0.1	0.017	40.4%	1.11 [1.07, 1.14]
**Total (95% CI)**			**100.0%**	**1.10 [1.07, 1.12]**

Heterogeneity: Chi² = 0.40, df = 1 (P = 0.52); I² = 0%
Test for overall effect: Z = 8.48 (P < 0.00001)

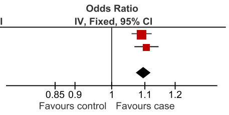

rs10757278

Study or Subgroup	log[Odds Ratio]	SE	Weight	Odds Ratio IV, Random, 95% CI
Helgadottir et al. 2007	0.045	0.004	37.3%	1.05 [1.04, 1.05]
Hirokawa et al. 2015	0.111	0.018	34.7%	1.12 [1.08, 1.16]
Yamada et al. 2018 (b)	-0.114	0.037	28.1%	0.89 [0.83, 0.96]
**Total (95% CI)**			**100.0%**	**1.02 [0.95, 1.11]**

Heterogeneity: Tau² = 0.00; Chi² = 31.80, df = 2 (P < 0.00001); I² = 94%
Test for overall effect: Z = 0.59 (P = 0.55)

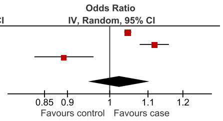

rs10774610

Study or Subgroup	log[Odds Ratio]	SE	Weight	Odds Ratio IV, Fixed, 95% CI
Yamada et al. 2018 (a)	0.225	0.068	44.6%	1.25 [1.10, 1.43]
Yamada et al. 2018 (b)	0.14	0.061	55.4%	1.15 [1.02, 1.30]
**Total (95% CI)**			**100.0%**	**1.19 [1.09, 1.31]**

Heterogeneity: Chi² = 0.87, df = 1 (P = 0.35); I² = 0%
Test for overall effect: Z = 3.92 (P < 0.0001)

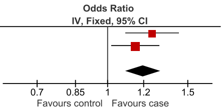

rs10840293

Study or Subgroup	log[Odds Ratio]	SE	Weight	Odds Ratio IV, Random, 95% CI
Fall et al. 2018	0.004	0.007	14.2%	1.00 [0.99, 1.02]
Nelson et al. 2017	0.021	0.004	26.6%	1.02 [1.01, 1.03]
Nikpay et al. 2015	0.025	0.004	26.6%	1.03 [1.02, 1.03]
van der Harst et al. 2018	0.02	0.003	32.6%	1.02 [1.01, 1.03]
**Total (95% CI)**			**100.0%**	**1.02 [1.01, 1.03]**

Heterogeneity: Tau² = 0.00; Chi² = 6.84, df = 3 (P = 0.08); I² = 56%
Test for overall effect: Z = 6.10 (P < 0.00001)

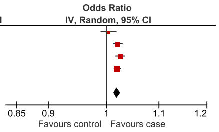

rs10841443

Study or Subgroup	log[Odds Ratio]	SE	Weight	Odds Ratio IV, Fixed, 95% CI
Klarin et al. 2017	0.021	0.004	22.0%	1.02 [1.01, 1.03]
Matsunaga et al. 2020	0.021	0.003	39.0%	1.02 [1.02, 1.03]
van der Harst et al. 2018	0.022	0.003	39.0%	1.02 [1.02, 1.03]
**Total (95% CI)**			**100.0%**	**1.02 [1.02, 1.03]**

Heterogeneity: Chi² = 0.07, df = 2 (P = 0.97); I² = 0%
Test for overall effect: Z = 11.41 (P < 0.00001)

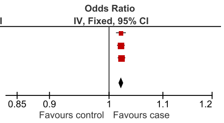

rs10849915

Study or Subgroup	log[Odds Ratio]	SE	Weight	Odds Ratio IV, Fixed, 95% CI
Yamada et al. 2018 (a)	0.212	0.069	44.7%	1.24 [1.08, 1.42]
Yamada et al. 2018 (b)	0.124	0.062	55.3%	1.13 [1.00, 1.28]
**Total (95% CI)**			**100.0%**	**1.18 [1.08, 1.29]**

Heterogeneity: Chi² = 0.90, df = 1 (P = 0.34); I² = 0%
Test for overall effect: Z = 3.54 (P = 0.0004)

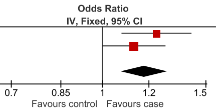

rs10857147

Study or Subgroup	log[Odds Ratio]	SE	Weight	Odds Ratio IV, Fixed, 95% CI
Charmet et al. 2018	0.009	0.039	0.3%	1.01 [0.93, 1.09]
Klarin et al. 2017	0.025	0.004	26.4%	1.03 [1.02, 1.03]
Nelson et al. 2017	0.025	0.004	26.4%	1.03 [1.02, 1.03]
van der Harst et al. 2018	0.021	0.003	46.9%	1.02 [1.02, 1.03]
**Total (95% CI)**			**100.0%**	**1.02 [1.02, 1.03]**

Heterogeneity: Chi² = 1.07, df = 3 (P = 0.78); I² = 0%
Test for overall effect: Z = 11.23 (P < 0.00001)

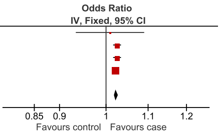

rs1088868

Study or Subgroup	log[Odds Ratio]	SE	Weight	Odds Ratio IV, Random, 95% CI
Nelson et al. 2017	0.017	0.004	46.5%	1.02 [1.01, 1.03]
van der Harst et al. 2018	0.007	0.003	53.5%	1.01 [1.00, 1.01]
**Total (95% CI)**			**100.0%**	**1.01 [1.00, 1.02]**

Heterogeneity: Tau² = 0.00; Chi² = 4.00, df = 1 (P = 0.05); I² = 75%
Test for overall effect: Z = 2.34 (P = 0.02)

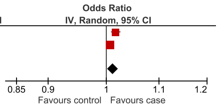

rs10947262

Study or Subgroup	log[Odds Ratio]	SE	Weight	Odds Ratio IV, Fixed, 95% CI
Hirokawa et al. 2015	0.068	0.019	84.9%	1.07 [1.03, 1.11]
Yamada et al. 2018 (b)	0.119	0.045	15.1%	1.13 [1.03, 1.23]
**Total (95% CI)**			**100.0%**	**1.08 [1.04, 1.12]**

Heterogeneity: Chi² = 1.09, df = 1 (P = 0.30); I² = 8%
Test for overall effect: Z = 4.33 (P < 0.0001)

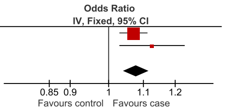

rs10947789

Study or Subgroup	log[Odds Ratio]	SE	Weight	Odds Ratio IV, Random, 95% CI
Charmet et al. 2018	0.004	0.003	36.1%	1.00 [1.00, 1.01]
Dichgans et al. 2014	0.018	0.003	36.1%	1.02 [1.01, 1.02]
Fall et al. 2018	0.029	0.041	2.0%	1.03 [0.95, 1.12]
van der Harst et al. 2018	0.027	0.007	25.8%	1.03 [1.01, 1.04]
**Total (95% CI)**			**100.0%**	**1.02 [1.00, 1.03]**

Heterogeneity: Tau² = 0.00; Chi² = 15.84, df = 3 (P = 0.001); I² = 81%
Test for overall effect: Z = 2.57 (P = 0.01)

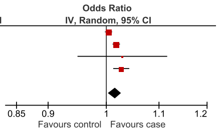

rs10953541

Study or Subgroup	log[Odds Ratio]	SE	Weight	Odds Ratio IV, Random, 95% CI
Charmet et al. 2018	0.064	0.041	4.8%	1.07 [0.98, 1.16]
Fall et al. 2018	0.004	0.002	24.8%	1.00 [1.00, 1.01]
Han et al. 2017	0.036	0.022	11.3%	1.04 [0.99, 1.08]
Hirokawa et al. 2015	0.004	0.033	6.7%	1.00 [0.94, 1.07]
Lee et al. 2013	-0.022	0.006	22.9%	0.98 [0.97, 0.99]
Peden et al. 2011	0.033	0.006	22.9%	1.03 [1.02, 1.05]
Qi et al. 2013	-0.022	0.033	6.7%	0.98 [0.92, 1.04]
**Total (95% CI)**			**100.0%**	**1.01 [0.99, 1.03]**

Heterogeneity: Tau² = 0.00; Chi² = 46.93, df = 6 (P < 0.00001); I² = 87%
Test for overall effect: Z = 0.95 (P = 0.34)

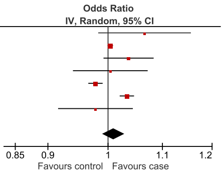

rs10965212

Study or Subgroup	log[Odds Ratio]	SE	Weight	Odds Ratio IV, Fixed, 95% CI
Samani et al. 2007	0.1	0.014	24.6%	1.11 [1.08, 1.14]
Wild et al. 2011	0.083	0.008	75.4%	1.09 [1.07, 1.10]
**Total (95% CI)**			**100.0%**	**1.09 [1.08, 1.11]**

Heterogeneity: Chi² = 1.11, df = 1 (P = 0.29); I² = 10%
Test for overall effect: Z = 12.55 (P < 0.00001)

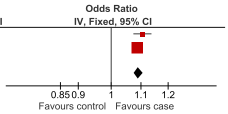

rs10965215

Study or Subgroup	log[Odds Ratio]	SE	Weight	Odds Ratio IV, Fixed, 95% CI
Samani et al. 2007	0.107	0.015	22.1%	1.11 [1.08, 1.15]
Wild et al. 2011	0.083	0.008	77.9%	1.09 [1.07, 1.10]
**Total (95% CI)**			**100.0%**	**1.09 [1.08, 1.11]**

Heterogeneity: Chi² = 1.99, df = 1 (P = 0.16); I² = 50%
Test for overall effect: Z = 12.51 (P < 0.00001)

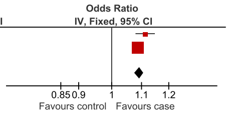

rs10965219

Study or Subgroup	log[Odds Ratio]	SE	Weight	Odds Ratio IV, Fixed, 95% CI
Pechlivanis et al. 2013	0.064	0.023	8.6%	1.07 [1.02, 1.12]
Samani et al. 2007	0.104	0.015	20.2%	1.11 [1.08, 1.14]
Wild et al. 2011	0.09	0.008	71.2%	1.09 [1.08, 1.11]
**Total (95% CI)**			**100.0%**	**1.09 [1.08, 1.11]**

Heterogeneity: Chi² = 2.14, df = 2 (P = 0.34); I² = 7%
Test for overall effect: Z = 13.43 (P < 0.00001)

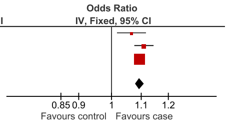

rs11042937

Study or Subgroup	log[Odds Ratio]	SE	Weight	Odds Ratio IV, Fixed, 95% CI
Charmet et al. 2018	0.025	0.035	0.7%	1.03 [0.96, 1.10]
van der Harst et al. 2018	0.018	0.003	99.3%	1.02 [1.01, 1.02]
**Total (95% CI)**			**100.0%**	**1.02 [1.01, 1.02]**

Heterogeneity: Chi² = 0.04, df = 1 (P = 0.84); I² = 0%
Test for overall effect: Z = 6.04 (P < 0.00001)

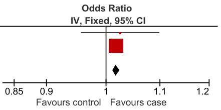

rs11057401

Study or Subgroup	log[Odds Ratio]	SE	Weight	Odds Ratio IV, Fixed, 95% CI
Klarin et al. 2017	-0.025	0.004	22.0%	0.98 [0.97, 0.98]
Nelson et al. 2017	-0.018	0.003	39.0%	0.98 [0.98, 0.99]
van der Harst et al. 2018	-0.017	0.003	39.0%	0.98 [0.98, 0.99]
**Total (95% CI)**			**100.0%**	**0.98 [0.98, 0.98]**

Heterogeneity: Chi² = 2.80, df = 2 (P = 0.25); I² = 29%
Test for overall effect: Z = 10.22 (P < 0.00001)

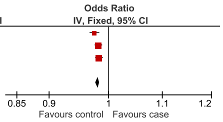

rs11057830

Study or Subgroup	log[Odds Ratio]	SE	Weight	Odds Ratio IV, Fixed, 95% CI
Nelson et al. 2017	0.029	0.005	39.0%	1.03 [1.02, 1.04]
van der Harst et al. 2018	0.036	0.004	61.0%	1.04 [1.03, 1.04]
**Total (95% CI)**			**100.0%**	**1.03 [1.03, 1.04]**

Heterogeneity: Chi² = 1.20, df = 1 (P = 0.27); I² = 16%
Test for overall effect: Z = 10.65 (P < 0.00001)

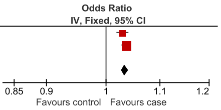

rs11065979

Study or Subgroup	log[Odds Ratio]	SE	Weight	Odds Ratio IV, Fixed, 95% CI
Nikpay et al. 2015	0.029	0.004	36.0%	1.03 [1.02, 1.04]
van der Harst et al. 2018	0.03	0.003	64.0%	1.03 [1.02, 1.04]
**Total (95% CI)**			**100.0%**	**1.03 [1.03, 1.03]**

Heterogeneity: Chi² = 0.04, df = 1 (P = 0.84); I² = 0%
Test for overall effect: Z = 12.35 (P < 0.00001)

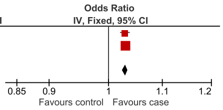

rs11066001

Study or Subgroup	log[Odds Ratio]	SE	Weight	Odds Ratio IV, Fixed, 95% CI
Lee et al. 2013	0.146	0.022	22.9%	1.16 [1.11, 1.21]
Takeuchi et al. 2012	0.152	0.012	77.1%	1.16 [1.14, 1.19]
**Total (95% CI)**			**100.0%**	**1.16 [1.14, 1.19]**

Heterogeneity: Chi² = 0.06, df = 1 (P = 0.81); I² = 0%
Test for overall effect: Z = 14.30 (P < 0.00001)

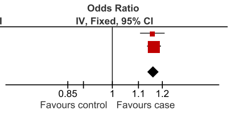

rs11066015

Study or Subgroup	log[Odds Ratio]	SE	Weight	Odds Ratio IV, Random, 95% CI
Lee et al. 2013	0.149	0.023	33.9%	1.16 [1.11, 1.21]
Yamada et al. 2018 (a)	-0.432	0.058	32.9%	0.65 [0.58, 0.73]
Yamada et al. 2018 (b)	0.253	0.051	33.2%	1.29 [1.17, 1.42]
**Total (95% CI)**			**100.0%**	**0.99 [0.71, 1.39]**

Heterogeneity: Tau² = 0.09; Chi² = 97.65, df = 2 (P < 0.00001); I² = 98%
Test for overall effect: Z = 0.04 (P = 0.97)

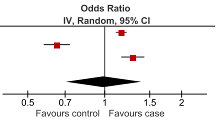

rs11066280

Study or Subgroup	log[Odds Ratio]	SE	Weight	Odds Ratio IV, Random, 95% CI
Lee et al. 2013	-0.124	0.022	26.0%	0.88 [0.85, 0.92]
Lu et al. 2012	0.076	0.011	26.3%	1.08 [1.06, 1.10]
Yamada et al. 2018 (a)	0.425	0.058	23.5%	1.53 [1.37, 1.71]
Yamada et al. 2018 (b)	0.238	0.05	24.2%	1.27 [1.15, 1.40]
**Total (95% CI)**			**100.0%**	**1.16 [0.98, 1.37]**

Heterogeneity: Tau² = 0.03; Chi² = 123.92, df = 3 (P < 0.00001); I² = 98%
Test for overall effect: Z = 1.71 (P = 0.09)

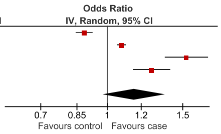

rs11099493

Study or Subgroup	log[Odds Ratio]	SE	Weight	Odds Ratio IV, Fixed, 95% CI
Matsunaga et al. 2020	0.021	0.004	36.0%	1.02 [1.01, 1.03]
van der Harst et al. 2018	0.017	0.003	64.0%	1.02 [1.01, 1.02]
**Total (95% CI)**			**100.0%**	**1.02 [1.01, 1.02]**

Heterogeneity: Chi² = 0.64, df = 1 (P = 0.42); I² = 0%
Test for overall effect: Z = 7.68 (P < 0.00001)

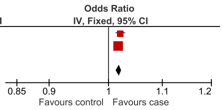

rs111245230

Study or Subgroup	log[Odds Ratio]	SE	Weight	Odds Ratio IV, Fixed, 95% CI
Nelson et al. 2017	0.049	0.01	32.9%	1.05 [1.03, 1.07]
van der Harst et al. 2018	0.043	0.007	67.1%	1.04 [1.03, 1.06]
**Total (95% CI)**			**100.0%**	**1.05 [1.03, 1.06]**

Heterogeneity: Chi² = 0.24, df = 1 (P = 0.62); I² = 0%
Test for overall effect: Z = 7.84 (P < 0.00001)

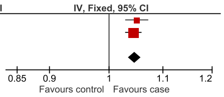

rs11170820

Study or Subgroup	log[Odds Ratio]	SE	Weight	Odds Ratio IV, Fixed, 95% CI
Nelson et al. 2017	0.037	0.008	36.0%	1.04 [1.02, 1.05]
van der Harst et al. 2018	0.042	0.006	64.0%	1.04 [1.03, 1.06]
**Total (95% CI)**			**100.0%**	**1.04 [1.03, 1.05]**

Heterogeneity: Chi² = 0.25, df = 1 (P = 0.62); I² = 0%
Test for overall effect: Z = 8.38 (P < 0.00001)

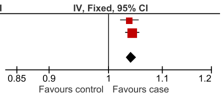

rs11172113

Study or Subgroup	log[Odds Ratio]	SE	Weight	Odds Ratio IV, Fixed, 95% CI
Nelson et al. 2017	0.017	0.004	36.0%	1.02 [1.01, 1.03]
van der Harst et al. 2018	0.015	0.003	64.0%	1.02 [1.01, 1.02]
**Total (95% CI)**			**100.0%**	**1.02 [1.01, 1.02]**

Heterogeneity: Chi² = 0.16, df = 1 (P = 0.69); I² = 0%
Test for overall effect: Z = 6.55 (P < 0.00001)

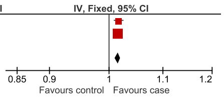

rs11191416

Study or Subgroup	log[Odds Ratio]	SE	Weight	Odds Ratio IV, Fixed, 95% CI
Charmet et al. 2018	-0.046	0.061	0.4%	0.96 [0.85, 1.08]
Nelson et al. 2017	0.033	0.005	58.8%	1.03 [1.02, 1.04]
Nikpay et al. 2015	0.033	0.006	40.8%	1.03 [1.02, 1.05]
**Total (95% CI)**			**100.0%**	**1.03 [1.03, 1.04]**

Heterogeneity: Chi² = 1.67, df = 2 (P = 0.43); I² = 0%
Test for overall effect: Z = 8.53 (P < 0.00001)

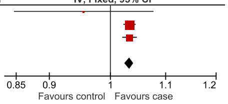

rs11203042

Study or Subgroup	log[Odds Ratio]	SE	Weight	Odds Ratio IV, Random, 95% CI
Dichgans et al. 2014	0.025	0.006	46.0%	1.03 [1.01, 1.04]
Fall et al. 2018	0.004	0.001	54.0%	1.00 [1.00, 1.01]
**Total (95% CI)**			**100.0%**	**1.01 [0.99, 1.03]**

Heterogeneity: Tau² = 0.00; Chi² = 11.92, df = 1 (P = 0.0006); I² = 92%
Test for overall effect: Z = 1.31 (P = 0.19)

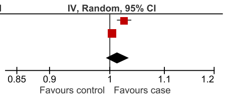

rs11206510

Study or Subgroup	log[Odds Ratio]	SE	Weight	Odds Ratio IV, Random, 95% CI
Charmet et al. 2018	0.033	0.045	1.4%	1.03 [0.95, 1.13]
Dichgans et al. 2014	0.036	0.011	9.2%	1.04 [1.01, 1.06]
Fall et al. 2018	0.009	0.003	13.7%	1.01 [1.00, 1.01]
Han et al. 2017	0	0.036	2.0%	1.00 [0.93, 1.07]
Hirokawa et al. 2015	-0.051	0.044	1.4%	0.95 [0.87, 1.04]
Kathiresan et al. 2009	0.061	0.011	9.2%	1.06 [1.04, 1.09]
Lee et al. 2013	0.037	0.025	3.7%	1.04 [0.99, 1.09]
Matsunaga et al. 2020	0.029	0.005	12.8%	1.03 [1.02, 1.04]
Nikpay et al. 2015	0.033	0.006	12.2%	1.03 [1.02, 1.05]
Peden et al. 2011	0.021	0.012	8.6%	1.02 [1.00, 1.05]
Qi et al. 2013	0.083	0.037	2.0%	1.09 [1.01, 1.17]
Reilly et al. 2011	0.004	0.007	11.6%	1.00 [0.99, 1.02]
Schunkert et al. 2011	0.033	0.006	12.2%	1.03 [1.02, 1.05]
**Total (95% CI)**			**100.0%**	**1.03 [1.02, 1.04]**

Heterogeneity: Tau² = 0.00; Chi² = 53.66, df = 12 (P < 0.00001); I² = 78%
Test for overall effect: Z = 4.83 (P < 0.00001)

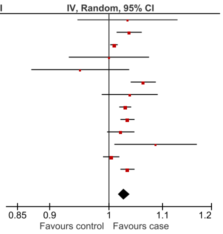

rs1122608

Study or Subgroup	log[Odds Ratio]	SE	Weight	Odds Ratio IV, Random, 95% CI
Charmet et al. 2018	-0.06	0.04	5.8%	0.94 [0.87, 1.02]
Dichgans et al. 2014	0.056	0.009	14.7%	1.06 [1.04, 1.08]
Fall et al. 2018	0.009	0.001	16.1%	1.01 [1.01, 1.01]
Kathiresan et al. 2009	0.061	0.01	14.5%	1.06 [1.04, 1.08]
Lee et al. 2013	-0.027	0.006	15.5%	0.97 [0.96, 0.98]
Qi et al. 2013	0.033	0.034	7.0%	1.03 [0.97, 1.10]
Reilly et al. 2011	0.013	0.018	11.8%	1.01 [0.98, 1.05]
Schunkert et al. 2011	0.057	0.009	14.7%	1.06 [1.04, 1.08]
**Total (95% CI)**			**100.0%**	**1.02 [1.00, 1.05]**

Heterogeneity: Tau² = 0.00; Chi² = 121.84, df = 7 (P < 0.00001); I² = 94%
Test for overall effect: Z = 1.93 (P = 0.05)

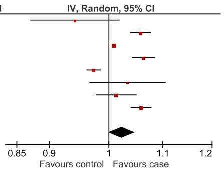

rs112635299

Study or Subgroup	log[Odds Ratio]	SE	Weight	Odds Ratio IV, Fixed, 95% CI
Nelson et al. 2017	-0.071	0.017	21.9%	0.93 [0.90, 0.96]
van der Harst et al. 2018	-0.06	0.009	78.1%	0.94 [0.93, 0.96]
**Total (95% CI)**			**100.0%**	**0.94 [0.92, 0.95]**

Heterogeneity: Chi² = 0.33, df = 1 (P = 0.57); I² = 0%
Test for overall effect: Z = 7.85 (P < 0.00001)

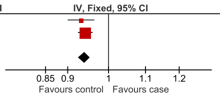

rs112941079

Study or Subgroup	log[Odds Ratio]	SE	Weight	Odds Ratio IV, Fixed, 95% CI
Nelson et al. 2017	0.027	0.006	30.8%	1.03 [1.02, 1.04]
van der Harst et al. 2018	0.027	0.004	69.2%	1.03 [1.02, 1.04]
**Total (95% CI)**			**100.0%**	**1.03 [1.02, 1.03]**

Heterogeneity: Chi² = 0.00, df = 1 (P = 1.00); I² = 0%
Test for overall effect: Z = 8.11 (P < 0.00001)

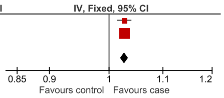

rs11509880

Study or Subgroup	log[Odds Ratio]	SE	Weight	Odds Ratio IV, Fixed, 95% CI
Nelson et al. 2017	0.013	0.003	50.0%	1.01 [1.01, 1.02]
van der Harst et al. 2018	0.017	0.003	50.0%	1.02 [1.01, 1.02]
**Total (95% CI)**			**100.0%**	**1.02 [1.01, 1.02]**

Heterogeneity: Chi² = 0.89, df = 1 (P = 0.35); I² = 0%
Test for overall effect: Z = 7.07 (P < 0.00001)

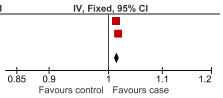

rs115287176

Study or Subgroup	log[Odds Ratio]	SE	Weight	Odds Ratio IV, Fixed, 95% CI
Yamada et al. 2018 (a)	-2	0.266	47.9%	0.14 [0.08, 0.23]
Yamada et al. 2018 (b)	-1.699	0.255	52.1%	0.18 [0.11, 0.30]
**Total (95% CI)**			**100.0%**	**0.16 [0.11, 0.23]**

Heterogeneity: Chi² = 0.67, df = 1 (P = 0.41); I² = 0%
Test for overall effect: Z = 10.01 (P < 0.00001)

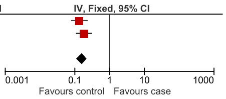

rs11556924

Study or Subgroup	log[Odds Ratio]	SE	Weight	Odds Ratio IV, Random, 95% CI
Charmet et al. 2018	-0.013	0.036	1.3%	0.99 [0.92, 1.06]
Dichgans et al. 2014	0.041	0.006	12.1%	1.04 [1.03, 1.05]
Fall et al. 2018	0.004	0.004	13.9%	1.00 [1.00, 1.01]
Han et al. 2017	0.022	0.034	1.4%	1.02 [0.96, 1.09]
Lee et al. 2013	0.045	0.046	0.8%	1.05 [0.96, 1.14]
Matsunaga et al. 2020	0.029	0.004	13.9%	1.03 [1.02, 1.04]
Nelson et al. 2017	0.029	0.004	13.9%	1.03 [1.02, 1.04]
Nikpay et al. 2015	0.033	0.005	13.0%	1.03 [1.02, 1.04]
Qi et al. 2013	-0.027	0.029	1.9%	0.97 [0.92, 1.03]
Schunkert et al. 2011	0.037	0.005	13.0%	1.04 [1.03, 1.05]
van der Harst et al. 2018	0.027	0.003	14.7%	1.03 [1.02, 1.03]
**Total (95% CI)**			**100.0%**	**1.03 [1.02, 1.04]**

Heterogeneity: Tau² = 0.00; Chi² = 49.17, df = 10 (P < 0.00001); I² = 80%
Test for overall effect: Z = 6.25 (P < 0.00001)

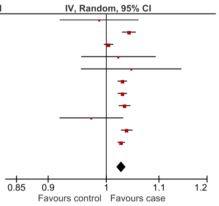

rs11591147

Study or Subgroup	log[Odds Ratio]	SE	Weight	Odds Ratio IV, Fixed, 95% CI
Nelson et al. 2017	-0.097	0.015	39.0%	0.91 [0.88, 0.93]
van der Harst et al. 2018	-0.123	0.012	61.0%	0.88 [0.86, 0.91]
**Total (95% CI)**			**100.0%**	**0.89 [0.88, 0.91]**

Heterogeneity: Chi² = 1.83, df = 1 (P = 0.18); I² = 45%
Test for overall effect: Z = 12.04 (P < 0.00001)

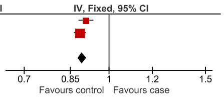

rs11617955

Study or Subgroup	log[Odds Ratio]	SE	Weight	Odds Ratio IV, Fixed, 95% CI
Nelson et al. 2017	0.036	0.006	25.1%	1.04 [1.02, 1.05]
Nikpay et al. 2015	0.037	0.007	18.4%	1.04 [1.02, 1.05]
van der Harst et al. 2018	0.035	0.004	56.5%	1.04 [1.03, 1.04]
**Total (95% CI)**			**100.0%**	**1.04 [1.03, 1.04]**

Heterogeneity: Chi² = 0.07, df = 2 (P = 0.97); I² = 0%
Test for overall effect: Z = 11.85 (P < 0.00001)

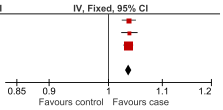

rs116843064

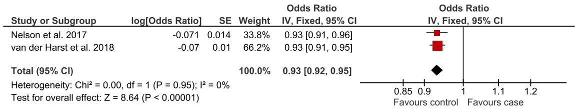

rs11728590

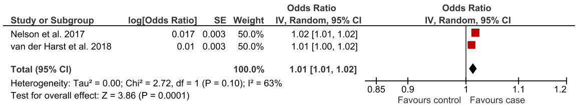

rs11752643

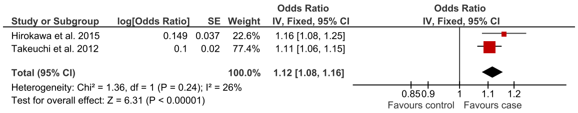

rs11810571

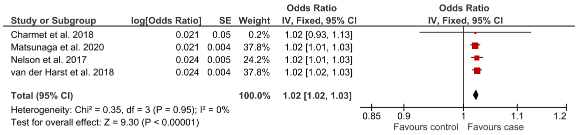

rs11823828

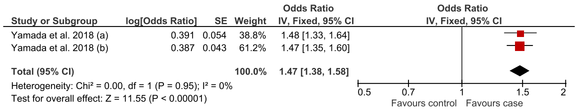

rs11838776

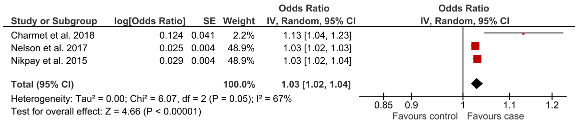

rs12146487

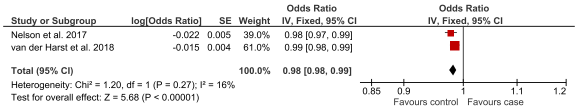

rs1214752

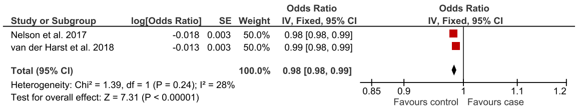

rs12190287

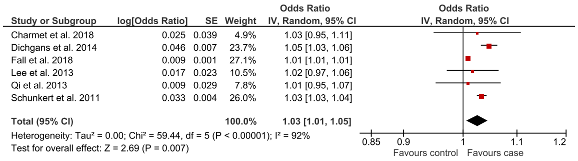

rs12202017

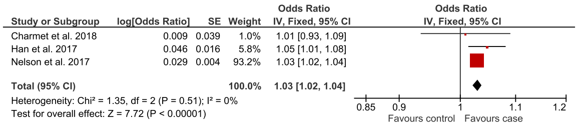

rs12229654

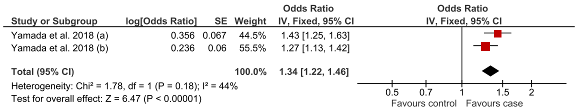

rs12231744

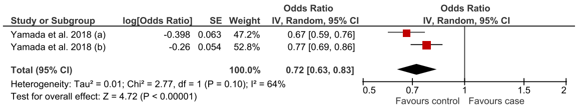

rs12413409

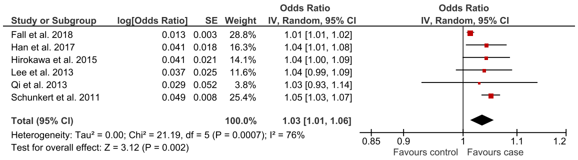

rs12493885

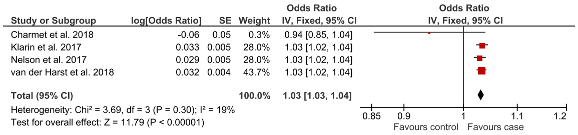

rs1250229

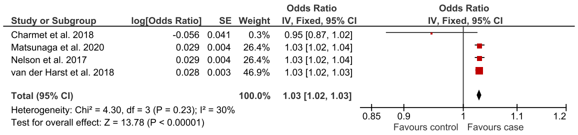

rs12524865

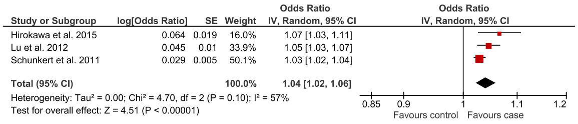

rs12526453

Study or Subgroup	log[Odds Ratio]	SE	Weight	Odds Ratio IV, Random, 95% CI
Fall et al. 2018	0.009	0.002	25.6%	1.01 [1.01, 1.01]
Kathiresan et al. 2009	0.049	0.009	21.7%	1.05 [1.03, 1.07]
Qi et al. 2013	0.037	0.029	8.6%	1.04 [0.98, 1.10]
Reilly et al. 2011	0.021	0.01	20.9%	1.02 [1.00, 1.04]
Schunkert et al. 2011	0.041	0.007	23.2%	1.04 [1.03, 1.06]
**Total (95% CI)**			**100.0%**	**1.03 [1.01, 1.05]**

Heterogeneity: Tau² = 0.00; Chi² = 37.27, df = 4 (P < 0.00001); I² = 89%
Test for overall effect: Z = 2.88 (P = 0.004)

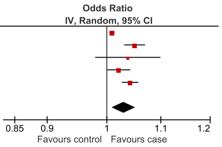

rs12619842

Study or Subgroup	log[Odds Ratio]	SE	Weight	Odds Ratio IV, Fixed, 95% CI
Matsunaga et al. 2020	-0.021	0.005	28.1%	0.98 [0.97, 0.99]
Nelson et al. 2017	-0.022	0.005	28.1%	0.98 [0.97, 0.99]
van der Harst et al. 2018	-0.024	0.004	43.9%	0.98 [0.97, 0.98]
**Total (95% CI)**			**100.0%**	**0.98 [0.97, 0.98]**

Heterogeneity: Chi² = 0.24, df = 2 (P = 0.89); I² = 0%
Test for overall effect: Z = 8.53 (P < 0.00001)

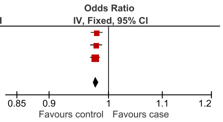

rs12740374

Study or Subgroup	log[Odds Ratio]	SE	Weight	Odds Ratio IV, Random, 95% CI
Lettre et al. 2011	0.013	0.037	31.7%	1.01 [0.94, 1.09]
van der Harst et al. 2018	-0.054	0.003	41.0%	0.95 [0.94, 0.95]
Yamada et al. 2018 (b)	-0.201	0.048	27.4%	0.82 [0.74, 0.90]
**Total (95% CI)**			**100.0%**	**0.93 [0.85, 1.01]**

Heterogeneity: Tau² = 0.00; Chi² = 12.66, df = 2 (P = 0.002); I² = 84%
Test for overall effect: Z = 1.68 (P = 0.09)

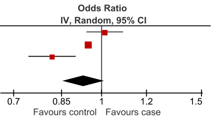

rs12801636

Study or Subgroup	log[Odds Ratio]	SE	Weight	Odds Ratio IV, Fixed, 95% CI
Charmet et al. 2018	-0.009	0.043	0.3%	0.99 [0.91, 1.08]
Matsunaga et al. 2020	-0.021	0.004	29.2%	0.98 [0.97, 0.99]
Nelson et al. 2017	-0.018	0.005	18.7%	0.98 [0.97, 0.99]
van der Harst et al. 2018	-0.019	0.003	51.9%	0.98 [0.98, 0.99]
**Total (95% CI)**			**100.0%**	**0.98 [0.98, 0.98]**

Heterogeneity: Chi² = 0.31, df = 3 (P = 0.96); I² = 0%
Test for overall effect: Z = 8.96 (P < 0.00001)

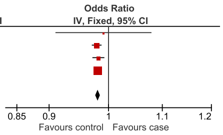

rs12922

Study or Subgroup	log[Odds Ratio]	SE	Weight	Odds Ratio IV, Fixed, 95% CI
Nelson et al. 2017	0.022	0.005	39.0%	1.02 [1.01, 1.03]
van der Harst et al. 2018	0.015	0.004	61.0%	1.02 [1.01, 1.02]
**Total (95% CI)**			**100.0%**	**1.02 [1.01, 1.02]**

Heterogeneity: Chi² = 1.20, df = 1 (P = 0.27); I² = 16%
Test for overall effect: Z = 5.68 (P < 0.00001)

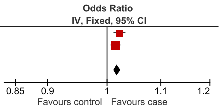

rs12936587

Study or Subgroup	log[Odds Ratio]	SE	Weight	Odds Ratio IV, Random, 95% CI
Charmet et al. 2018	0	0.035	1.8%	1.00 [0.93, 1.07]
Fall et al. 2018	0.009	0.004	30.1%	1.01 [1.00, 1.02]
Lee et al. 2013	0.025	0.03	2.4%	1.03 [0.97, 1.09]
Qi et al. 2013	-0.009	0.029	2.5%	0.99 [0.94, 1.05]
Schunkert et al. 2011	0.029	0.004	30.1%	1.03 [1.02, 1.04]
van der Harst et al. 2018	0.016	0.003	33.2%	1.02 [1.01, 1.02]
**Total (95% CI)**			**100.0%**	**1.02 [1.01, 1.03]**

Heterogeneity: Tau² = 0.00; Chi² = 14.18, df = 5 (P = 0.01); I² = 65%
Test for overall effect: Z = 3.60 (P = 0.0003)

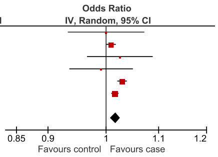

rs13003675

Study or Subgroup	log[Odds Ratio]	SE	Weight	Odds Ratio IV, Fixed, 95% CI
Nelson et al. 2017	0.017	0.003	50.0%	1.02 [1.01, 1.02]
van der Harst et al. 2018	0.015	0.003	50.0%	1.02 [1.01, 1.02]
**Total (95% CI)**			**100.0%**	**1.02 [1.01, 1.02]**

Heterogeneity: Chi² = 0.22, df = 1 (P = 0.64); I² = 0%
Test for overall effect: Z = 7.54 (P < 0.00001)

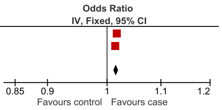

rs1333042

Study or Subgroup	log[Odds Ratio]	SE	Weight	Odds Ratio IV, Random, 95% CI
Aoki et al. 2011	0.058	0.025	10.8%	1.06 [1.01, 1.11]
Erdmann et al. 2011	0.049	0.007	14.6%	1.05 [1.04, 1.06]
Han et al. 2017	0.083	0.014	13.4%	1.09 [1.06, 1.12]
Lee et al. 2013	0.114	0.019	12.3%	1.12 [1.08, 1.16]
Lettre et al. 2011	0.004	0.039	7.7%	1.00 [0.93, 1.08]
Lu et al. 2012	0.137	0.01	14.2%	1.15 [1.12, 1.17]
Samani et al. 2007	0.127	0.018	12.5%	1.14 [1.10, 1.18]
Wild et al. 2011	0.114	0.009	14.3%	1.12 [1.10, 1.14]
**Total (95% CI)**			**100.0%**	**1.09 [1.06, 1.13]**

Heterogeneity: Tau² = 0.00; Chi² = 76.06, df = 7 (P < 0.00001); I² = 91%
Test for overall effect: Z = 5.84 (P < 0.00001)

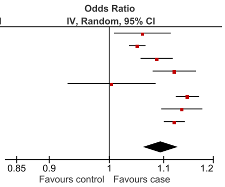

rs1333045

Study or Subgroup	log[Odds Ratio]	SE	Weight	Odds Ratio IV, Random, 95% CI
Lee et al. 2013	0.111	0.018	50.0%	1.12 [1.08, 1.16]
Wakil et al. 2016	-0.102	0.018	50.0%	0.90 [0.87, 0.94]
**Total (95% CI)**			**100.0%**	**1.00 [0.82, 1.24]**

Heterogeneity: Tau² = 0.02; Chi² = 70.01, df = 1 (P < 0.00001); I² = 99%
Test for overall effect: Z = 0.04 (P = 0.97)

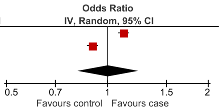

rs1333048

Study or Subgroup	log[Odds Ratio]	SE	Weight	Odds Ratio IV, Fixed, 95% CI
Lee et al. 2013	-0.111	0.018	12.8%	0.89 [0.86, 0.93]
Samani et al. 2007	-0.134	0.019	11.5%	0.87 [0.84, 0.91]
Wang et al. 2011	-0.081	0.023	7.8%	0.92 [0.88, 0.96]
Wild et al. 2011	-0.114	0.008	64.7%	0.89 [0.88, 0.91]
Yamada et al. 2018 (b)	-0.146	0.036	3.2%	0.86 [0.81, 0.93]
**Total (95% CI)**			**100.0%**	**0.89 [0.88, 0.90]**

Heterogeneity: Chi² = 3.98, df = 4 (P = 0.41); I² = 0%
Test for overall effect: Z = 17.77 (P < 0.00001)

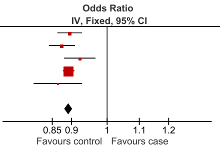

rs1333049

Study or Subgroup	log[Odds Ratio]	SE	Weight	Odds Ratio IV, Random, 95% CI
Burton et al. 2007	0.279	0.037	5.0%	1.32 [1.23, 1.42]
Erdmann et al. 2011	0.045	0.006	12.5%	1.05 [1.03, 1.06]
Lee et al. 2013	0.1	0.015	10.3%	1.11 [1.07, 1.14]
Li et al. 2018	0.097	0.008	12.2%	1.10 [1.08, 1.12]
Lu et al. 2012	0.127	0.01	11.7%	1.14 [1.11, 1.16]
Samani et al. 2007	0.134	0.015	10.3%	1.14 [1.11, 1.18]
van der Harst et al. 2018	0.082	0.003	12.9%	1.09 [1.08, 1.09]
Wang et al. 2011	0.107	0.025	7.5%	1.11 [1.06, 1.17]
Wild et al. 2011	0.104	0.007	12.4%	1.11 [1.09, 1.12]
Yamada et al. 2018 (b)	0.149	0.036	5.2%	1.16 [1.08, 1.25]
**Total (95% CI)**			**100.0%**	**1.12 [1.09, 1.14]**

Heterogeneity: Tau² = 0.00; Chi² = 115.78, df = 9 (P < 0.00001); I² = 92%
Test for overall effect: Z = 10.42 (P < 0.00001)

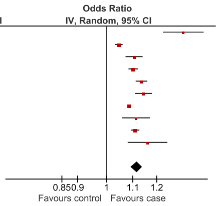

rs13723

Study or Subgroup	log[Odds Ratio]	SE	Weight	Odds Ratio IV, Fixed, 95% CI
Nelson et al. 2017	0.013	0.003	30.8%	1.01 [1.01, 1.02]
van der Harst et al. 2018	0.018	0.002	69.2%	1.02 [1.01, 1.02]
**Total (95% CI)**			**100.0%**	**1.02 [1.01, 1.02]**

Heterogeneity: Chi² = 1.92, df = 1 (P = 0.17); I² = 48%
Test for overall effect: Z = 9.89 (P < 0.00001)

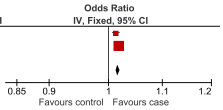

rs13734

Study or Subgroup	log[Odds Ratio]	SE	Weight	Odds Ratio IV, Fixed, 95% CI
Nelson et al. 2017	0.017	0.004	36.0%	1.02 [1.01, 1.03]
van der Harst et al. 2018	0.017	0.003	64.0%	1.02 [1.01, 1.02]
**Total (95% CI)**			**100.0%**	**1.02 [1.01, 1.02]**

Heterogeneity: Chi² = 0.00, df = 1 (P = 1.00); I² = 0%
Test for overall effect: Z = 7.08 (P < 0.00001)

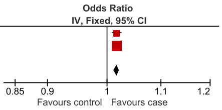

rs1378942

Study or Subgroup	log[Odds Ratio]	SE	Weight	Odds Ratio IV, Fixed, 95% CI
Siewert et al. 2018	-0.009	0.002	69.2%	0.99 [0.99, 0.99]
van der Harst et al. 2018	-0.011	0.003	30.8%	0.99 [0.98, 0.99]
**Total (95% CI)**			**100.0%**	**0.99 [0.99, 0.99]**

Heterogeneity: Chi² = 0.31, df = 1 (P = 0.58); I² = 0%
Test for overall effect: Z = 5.78 (P < 0.00001)

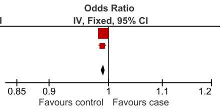

rs140570886

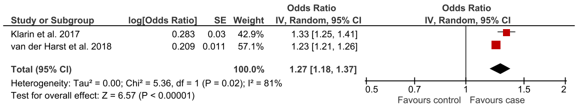

rs1412444

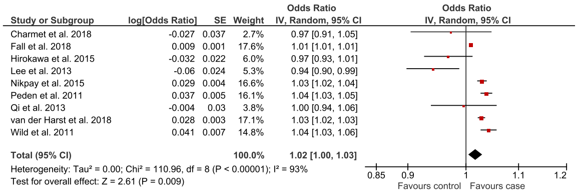

rs1412834

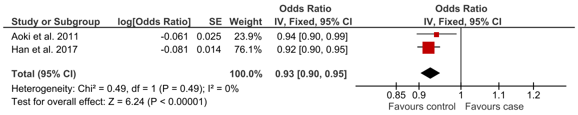

rs142695226

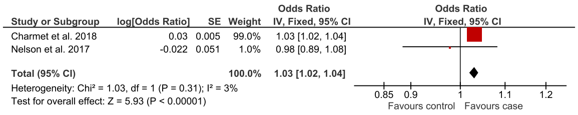

rs143803699

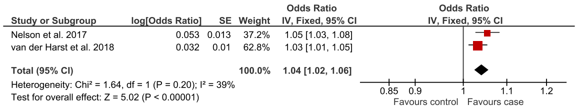

rs146092501

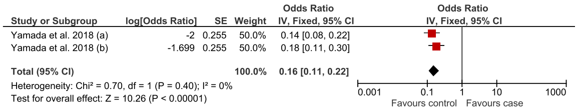

rs146879198

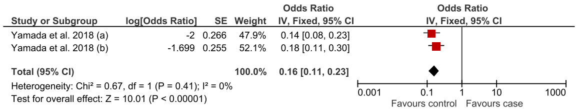

rs147555597

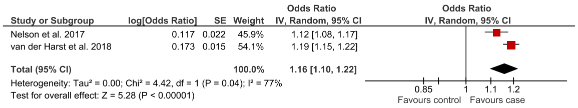

rs1537372

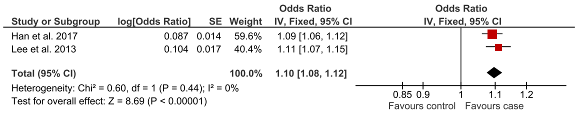

rs1537373

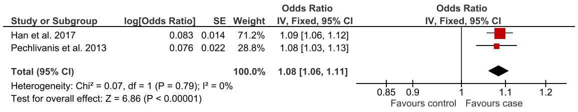

rs1537376

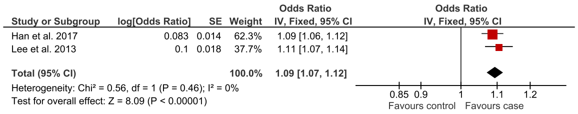

rs1561198

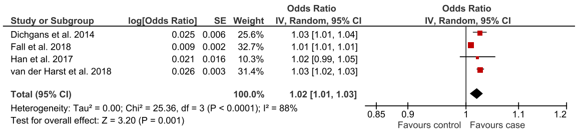

rs167479

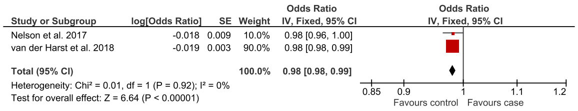

rs16986953

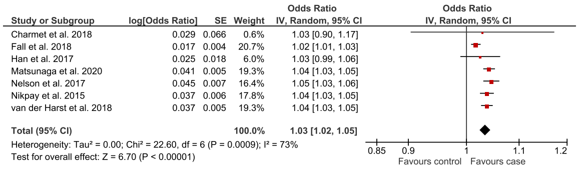

rs17087335

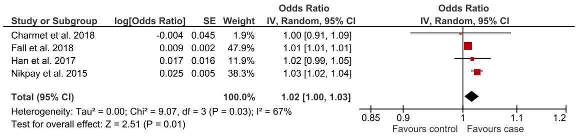

rs17114036

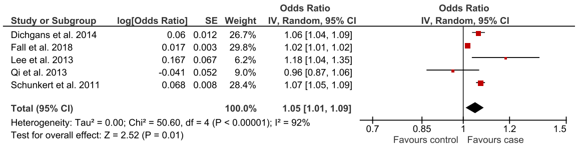

rs17228212

Study or Subgroup	log[Odds Ratio]	SE	Weight	Odds Ratio IV, Random, 95% CI
Kathiresan et al. 2009	-0.004	0.01	34.1%	1.00 [0.98, 1.02]
Samani et al. 2007	0.083	0.016	30.2%	1.09 [1.05, 1.12]
Schunkert et al. 2011	0.006	0.007	35.6%	1.01 [0.99, 1.02]
**Total (95% CI)**			**100.0%**	**1.03 [0.99, 1.07]**

Heterogeneity: Tau² = 0.00; Chi² = 22.99, df = 2 (P < 0.0001); I² = 91%
Test for overall effect: Z = 1.27 (P = 0.20)

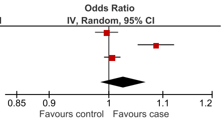

rs17411031

Study or Subgroup	log[Odds Ratio]	SE	Weight	Odds Ratio IV, Fixed, 95% CI
Han et al. 2017	0.036	0.019	6.5%	1.04 [1.00, 1.08]
Nikpay et al. 2015	0.021	0.005	93.5%	1.02 [1.01, 1.03]
**Total (95% CI)**			**100.0%**	**1.02 [1.01, 1.03]**

Heterogeneity: Chi² = 0.58, df = 1 (P = 0.45); I² = 0%
Test for overall effect: Z = 4.54 (P < 0.00001)

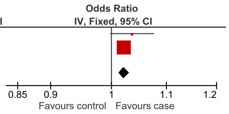

rs1746048

Study or Subgroup	log[Odds Ratio]	SE	Weight	Odds Ratio IV, Random, 95% CI
Hirokawa et al. 2015	0.013	0.02	11.4%	1.01 [0.97, 1.05]
Kathiresan et al. 2009	0.057	0.013	13.2%	1.06 [1.03, 1.09]
Lee et al. 2013	-0.041	0.006	14.5%	0.96 [0.95, 0.97]
Peden et al. 2011	0.025	0.009	14.0%	1.03 [1.01, 1.04]
Qi et al. 2013	-0.009	0.039	7.0%	0.99 [0.92, 1.07]
Reilly et al. 2011	0.009	0.005	14.6%	1.01 [1.00, 1.02]
Samani et al. 2007	0.111	0.022	10.9%	1.12 [1.07, 1.17]
Schunkert et al. 2011	0.037	0.006	14.5%	1.04 [1.03, 1.05]
**Total (95% CI)**			**100.0%**	**1.03 [1.00, 1.05]**

Heterogeneity: Tau² = 0.00; Chi² = 129.71, df = 7 (P < 0.00001); I² = 95%
Test for overall effect: Z = 1.75 (P = 0.08)

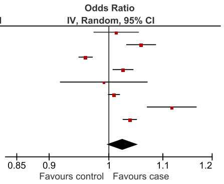

rs1746049

Study or Subgroup	log[Odds Ratio]	SE	Weight	Odds Ratio IV, Random, 95% CI
Samani et al. 2007	0.111	0.023	49.9%	1.12 [1.07, 1.17]
Wakil et al. 2016	-0.097	0.021	50.1%	0.91 [0.87, 0.95]
**Total (95% CI)**			**100.0%**	**1.01 [0.82, 1.23]**

Heterogeneity: Tau² = 0.02; Chi² = 44.60, df = 1 (P < 0.00001); I² = 98%
Test for overall effect: Z = 0.07 (P = 0.95)

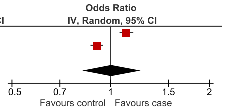

rs17464857

Study or Subgroup	log[Odds Ratio]	SE	Weight	Odds Ratio IV, Fixed, 95% CI
Charmet et al. 2018	0.049	0.049	0.2%	1.05 [0.95, 1.16]
Fall et al. 2018	0.013	0.002	99.8%	1.01 [1.01, 1.02]
**Total (95% CI)**			**100.0%**	**1.01 [1.01, 1.02]**

Heterogeneity: Chi² = 0.54, df = 1 (P = 0.46); I² = 0%
Test for overall effect: Z = 6.54 (P < 0.00001)

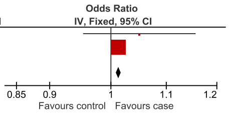

rs17465637

Study or Subgroup	log[Odds Ratio]	SE	Weight	Odds Ratio IV, Random, 95% CI
Fall et al. 2018	0.013	0.002	17.3%	1.01 [1.01, 1.02]
Han et al. 2017	0.029	0.017	8.3%	1.03 [1.00, 1.06]
Kathiresan et al. 2009	0.053	0.01	12.7%	1.05 [1.03, 1.08]
Lee et al. 2013	0.017	0.024	5.5%	1.02 [0.97, 1.07]
Qi et al. 2013	0.037	0.035	3.1%	1.04 [0.97, 1.11]
Reilly et al. 2011	0.013	0.005	16.0%	1.01 [1.00, 1.02]
Samani et al. 2007	0.079	0.017	8.3%	1.08 [1.05, 1.12]
Schunkert et al. 2011	0.057	0.011	12.0%	1.06 [1.04, 1.08]
van der Harst et al. 2018	0.035	0.003	16.9%	1.04 [1.03, 1.04]
**Total (95% CI)**			**100.0%**	**1.04 [1.02, 1.05]**

Heterogeneity: Tau² = 0.00; Chi² = 73.37, df = 8 (P < 0.00001); I² = 89%
Test for overall effect: Z = 5.15 (P < 0.00001)

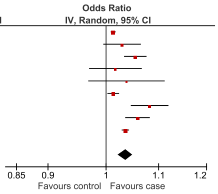

rs17514846

Study or Subgroup	log[Odds Ratio]	SE	Weight	Odds Ratio IV, Random, 95% CI
Charmet et al. 2018	0.033	0.035	2.7%	1.03 [0.97, 1.11]
Dichgans et al. 2014	0.029	0.007	22.7%	1.03 [1.02, 1.04]
Fall et al. 2018	0.013	0.001	32.6%	1.01 [1.01, 1.02]
Han et al. 2017	0.009	0.02	7.0%	1.01 [0.97, 1.05]
Hirokawa et al. 2015	-0.041	0.026	4.6%	0.96 [0.91, 1.01]
van der Harst et al. 2018	0.028	0.003	30.4%	1.03 [1.02, 1.03]
**Total (95% CI)**			**100.0%**	**1.02 [1.01, 1.03]**

Heterogeneity: Tau² = 0.00; Chi² = 31.66, df = 5 (P < 0.00001); I² = 84%
Test for overall effect: Z = 3.17 (P = 0.002)

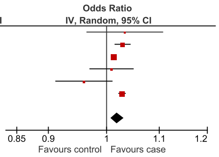

rs17517928

Study or Subgroup	log[Odds Ratio]	SE	Weight	Odds Ratio IV, Fixed, 95% CI
Klarin et al. 2017	0.025	0.004	36.0%	1.03 [1.02, 1.03]
van der Harst et al. 2018	0.021	0.003	64.0%	1.02 [1.02, 1.03]
**Total (95% CI)**			**100.0%**	**1.02 [1.02, 1.03]**

Heterogeneity: Chi² = 0.64, df = 1 (P = 0.42); I² = 0%
Test for overall effect: Z = 9.35 (P < 0.00001)

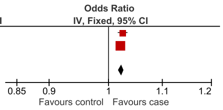

rs17581137

Study or Subgroup	log[Odds Ratio]	SE	Weight	Odds Ratio IV, Fixed, 95% CI
Nelson et al. 2017	0.018	0.005	26.5%	1.02 [1.01, 1.03]
van der Harst et al. 2018	0.017	0.003	73.5%	1.02 [1.01, 1.02]
**Total (95% CI)**			**100.0%**	**1.02 [1.01, 1.02]**

Heterogeneity: Chi² = 0.03, df = 1 (P = 0.86); I² = 0%
Test for overall effect: Z = 6.71 (P < 0.00001)

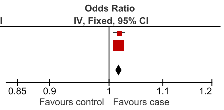

rs17608766

Study or Subgroup	log[Odds Ratio]	SE	Weight	Odds Ratio IV, Fixed, 95% CI
Charmet et al. 2018	-0.051	0.051	0.3%	0.95 [0.86, 1.05]
van der Harst et al. 2018	0.02	0.003	99.7%	1.02 [1.01, 1.03]
**Total (95% CI)**			**100.0%**	**1.02 [1.01, 1.03]**

Heterogeneity: Chi² = 1.93, df = 1 (P = 0.16); I² = 48%
Test for overall effect: Z = 6.60 (P < 0.00001)

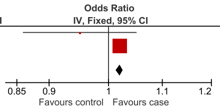

rs17609940

Study or Subgroup	log[Odds Ratio]	SE	Weight	Odds Ratio IV, Random, 95% CI
Charmet et al. 2018	0.121	0.046	5.7%	1.13 [1.03, 1.24]
Fall et al. 2018	0.004	0.002	41.8%	1.00 [1.00, 1.01]
Lee et al. 2013	0.017	0.055	4.2%	1.02 [0.91, 1.13]
Qi et al. 2013	0.004	0.035	9.0%	1.00 [0.94, 1.08]
Schunkert et al. 2011	0.029	0.005	39.3%	1.03 [1.02, 1.04]
**Total (95% CI)**			**100.0%**	**1.02 [1.00, 1.05]**

Heterogeneity: Tau² = 0.00; Chi² = 27.68, df = 4 (P < 0.0001); I² = 86%
Test for overall effect: Z = 1.78 (P = 0.07)

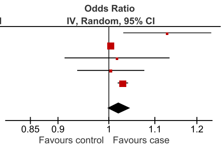

rs17672135

Study or Subgroup	log[Odds Ratio]	SE	Weight	Odds Ratio IV, Fixed, 95% CI
Burton et al. 2007	0.121	0.114	0.6%	1.13 [0.90, 1.41]
Samani et al. 2007	0.021	0.031	7.7%	1.02 [0.96, 1.09]
Schunkert et al. 2011	0.015	0.009	91.7%	1.02 [1.00, 1.03]
**Total (95% CI)**			**100.0%**	**1.02 [1.00, 1.03]**

Heterogeneity: Chi² = 0.89, df = 2 (P = 0.64); I² = 0%
Test for overall effect: Z = 1.86 (P = 0.06)

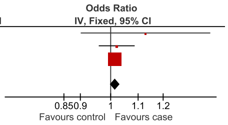

rs17678683

Study or Subgroup	log[Odds Ratio]	SE	Weight	Odds Ratio IV, Fixed, 95% CI
Charmet et al. 2018	0.09	0.07	0.2%	1.09 [0.95, 1.26]
Matsunaga et al. 2020	0.033	0.006	23.9%	1.03 [1.02, 1.05]
Nelson et al. 2017	0.029	0.006	23.9%	1.03 [1.02, 1.04]
Nikpay et al. 2015	0.041	0.007	17.6%	1.04 [1.03, 1.06]
van der Harst et al. 2018	0.024	0.005	34.4%	1.02 [1.01, 1.03]
**Total (95% CI)**			**100.0%**	**1.03 [1.03, 1.04]**

Heterogeneity: Chi² = 4.90, df = 4 (P = 0.30); I² = 18%
Test for overall effect: Z = 10.38 (P < 0.00001)

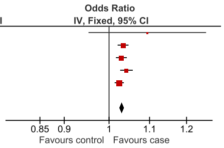

rs17843797

Study or Subgroup	log[Odds Ratio]	SE	Weight	Odds Ratio IV, Fixed, 95% CI
Klarin et al. 2017	0.029	0.005	39.0%	1.03 [1.02, 1.04]
van der Harst et al. 2018	0.029	0.004	61.0%	1.03 [1.02, 1.04]
**Total (95% CI)**			**100.0%**	**1.03 [1.02, 1.04]**

Heterogeneity: Chi² = 0.00, df = 1 (P = 1.00); I² = 0%
Test for overall effect: Z = 9.28 (P < 0.00001)

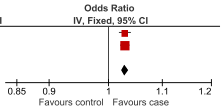

rs1800449

Study or Subgroup	log[Odds Ratio]	SE	Weight	Odds Ratio IV, Fixed, 95% CI
Klarin et al. 2017	0.029	0.004	37.9%	1.03 [1.02, 1.04]
Nelson et al. 2017	0.025	0.005	24.2%	1.03 [1.02, 1.04]
van der Harst et al. 2018	0.019	0.004	37.9%	1.02 [1.01, 1.03]
**Total (95% CI)**			**100.0%**	**1.02 [1.02, 1.03]**

Heterogeneity: Chi² = 3.16, df = 2 (P = 0.21); I² = 37%
Test for overall effect: Z = 9.85 (P < 0.00001)

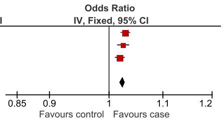

rs1800775

Study or Subgroup	log[Odds Ratio]	SE	Weight	Odds Ratio IV, Fixed, 95% CI
Charmet et al. 2018	0.004	0.035	0.7%	1.00 [0.94, 1.08]
van der Harst et al. 2018	-0.018	0.003	99.3%	0.98 [0.98, 0.99]
**Total (95% CI)**			**100.0%**	**0.98 [0.98, 0.99]**

Heterogeneity: Chi² = 0.39, df = 1 (P = 0.53); I² = 0%
Test for overall effect: Z = 5.97 (P < 0.00001)

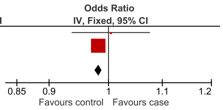

rs1801251

Study or Subgroup	log[Odds Ratio]	SE	Weight	Odds Ratio IV, Fixed, 95% CI
Charmet et al. 2018	0.061	0.036	0.7%	1.06 [0.99, 1.14]
van der Harst et al. 2018	0.018	0.003	99.3%	1.02 [1.01, 1.02]
**Total (95% CI)**			**100.0%**	**1.02 [1.01, 1.02]**

Heterogeneity: Chi² = 1.42, df = 1 (P = 0.23); I² = 29%
Test for overall effect: Z = 6.12 (P < 0.00001)

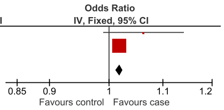

rs180803

Study or Subgroup	log[Odds Ratio]	SE	Weight	Odds Ratio IV, Fixed, 95% CI
Charmet et al. 2018	0.072	0.186	0.2%	1.07 [0.75, 1.55]
Han et al. 2017	0.027	0.028	8.2%	1.03 [0.97, 1.09]
Nelson et al. 2017	0.072	0.011	53.4%	1.07 [1.05, 1.10]
Nikpay et al. 2015	0.079	0.013	38.2%	1.08 [1.05, 1.11]
**Total (95% CI)**			**100.0%**	**1.07 [1.06, 1.09]**

Heterogeneity: Chi² = 2.86, df = 3 (P = 0.41); I² = 0%
Test for overall effect: Z = 8.83 (P < 0.00001)

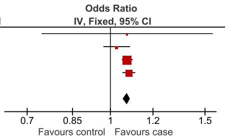

rs1831733

Study or Subgroup	log[Odds Ratio]	SE	Weight	Odds Ratio IV, Random, 95% CI
Han et al. 2017	0.078	0.014	46.7%	1.08 [1.05, 1.11]
Vujkovic et al. 2020	0.025	0.003	53.3%	1.03 [1.02, 1.03]
**Total (95% CI)**			**100.0%**	**1.05 [1.00, 1.11]**

Heterogeneity: Tau² = 0.00; Chi² = 13.70, df = 1 (P = 0.0002); I² = 93%
Test for overall effect: Z = 1.88 (P = 0.06)

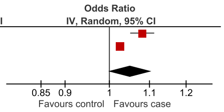

rs1842896

Study or Subgroup	log[Odds Ratio]	SE	Weight	Odds Ratio IV, Random, 95% CI
Hirokawa et al. 2015	-0.022	0.022	46.8%	0.98 [0.94, 1.02]
Lu et al. 2012	0.057	0.009	53.2%	1.06 [1.04, 1.08]
**Total (95% CI)**			**100.0%**	**1.02 [0.94, 1.10]**

Heterogeneity: Tau² = 0.00; Chi² = 11.05, df = 1 (P = 0.0009); I² = 91%
Test for overall effect: Z = 0.51 (P = 0.61)

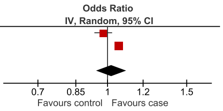

rs186696265

Study or Subgroup	log[Odds Ratio]	SE	Weight	Odds Ratio IV, Fixed, 95% CI
Nelson et al. 2017	0.21	0.016	36.0%	1.23 [1.20, 1.27]
van der Harst et al. 2018	0.237	0.012	64.0%	1.27 [1.24, 1.30]
**Total (95% CI)**			**100.0%**	**1.26 [1.23, 1.28]**

Heterogeneity: Chi² = 1.82, df = 1 (P = 0.18); I² = 45%
Test for overall effect: Z = 23.67 (P < 0.00001)

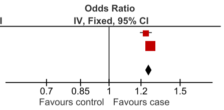

rs1867624

Study or Subgroup	log[Odds Ratio]	SE	Weight	Odds Ratio IV, Fixed, 95% CI
Charmet et al. 2018	-0.025	0.036	0.3%	0.98 [0.91, 1.05]
van der Harst et al. 2018	0.016	0.002	99.7%	1.02 [1.01, 1.02]
**Total (95% CI)**			**100.0%**	**1.02 [1.01, 1.02]**

Heterogeneity: Chi² = 1.29, df = 1 (P = 0.26); I² = 23%
Test for overall effect: Z = 7.95 (P < 0.00001)

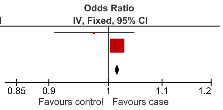

rs1870634

Study or Subgroup	log[Odds Ratio]	SE	Weight	Odds Ratio IV, Random, 95% CI
Nelson et al. 2017	0.021	0.003	35.6%	1.02 [1.02, 1.03]
Nikpay et al. 2015	0.033	0.004	28.8%	1.03 [1.03, 1.04]
van der Harst et al. 2018	0.026	0.003	35.6%	1.03 [1.02, 1.03]
**Total (95% CI)**			**100.0%**	**1.03 [1.02, 1.03]**

Heterogeneity: Tau² = 0.00; Chi² = 5.79, df = 2 (P = 0.06); I² = 65%
Test for overall effect: Z = 8.08 (P < 0.00001)

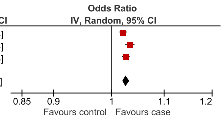

rs1878406

Study or Subgroup	log[Odds Ratio]	SE	Weight	Odds Ratio IV, Random, 95% CI
Charmet et al. 2018	0.027	0.054	5.1%	1.03 [0.92, 1.14]
Dichgans et al. 2014	0.041	0.008	43.4%	1.04 [1.03, 1.06]
Fall et al. 2018	0.013	0.002	51.5%	1.01 [1.01, 1.02]
**Total (95% CI)**			**100.0%**	**1.03 [1.00, 1.05]**

Heterogeneity: Tau² = 0.00; Chi² = 11.58, df = 2 (P = 0.003); I² = 83%
Test for overall effect: Z = 2.01 (P = 0.04)

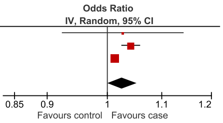

rs188378669

Study or Subgroup	log[Odds Ratio]	SE	Weight	Odds Ratio IV, Fixed, 95% CI
Yamada et al. 2018 (a)	-2	0.255	47.6%	0.14 [0.08, 0.22]
Yamada et al. 2018 (b)	-1.699	0.243	52.4%	0.18 [0.11, 0.29]
**Total (95% CI)**			**100.0%**	**0.16 [0.11, 0.22]**

Heterogeneity: Chi² = 0.73, df = 1 (P = 0.39); I² = 0%
Test for overall effect: Z = 10.47 (P < 0.00001)

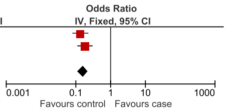

rs1883838

Study or Subgroup	log[Odds Ratio]	SE	Weight	Odds Ratio IV, Random, 95% CI
Aoki et al. 2011	-0.01	0.024	53.3%	0.99 [0.94, 1.04]
Kertai et al. 2015	0.241	0.069	46.7%	1.27 [1.11, 1.46]
**Total (95% CI)**			**100.0%**	**1.11 [0.87, 1.42]**

Heterogeneity: Tau² = 0.03; Chi² = 11.80, df = 1 (P = 0.0006); I² = 92%
Test for overall effect: Z = 0.86 (P = 0.39)

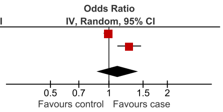

rs1892094

Study or Subgroup	log[Odds Ratio]	SE	Weight	Odds Ratio IV, Fixed, 95% CI
Charmet et al. 2018	0.033	0.035	0.3%	1.03 [0.97, 1.11]
van der Harst et al. 2018	-0.016	0.002	99.7%	0.98 [0.98, 0.99]
**Total (95% CI)**			**100.0%**	**0.98 [0.98, 0.99]**

Heterogeneity: Chi² = 1.95, df = 1 (P = 0.16); I² = 49%
Test for overall effect: Z = 7.93 (P < 0.00001)

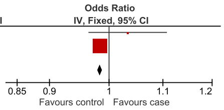

rs192210727

Study or Subgroup	log[Odds Ratio]	SE	Weight	Odds Ratio IV, Fixed, 95% CI
Yamada et al. 2018 (a)	-1.222	0.23	39.3%	0.29 [0.19, 0.46]
Yamada et al. 2018 (b)	-1.155	0.185	60.7%	0.32 [0.22, 0.45]
**Total (95% CI)**			**100.0%**	**0.31 [0.23, 0.41]**

Heterogeneity: Chi² = 0.05, df = 1 (P = 0.82); I² = 0%
Test for overall effect: Z = 8.19 (P < 0.00001)

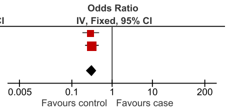

rs1924981

Study or Subgroup	log[Odds Ratio]	SE	Weight	Odds Ratio IV, Fixed, 95% CI
Nelson et al. 2017	0.021	0.004	50.0%	1.02 [1.01, 1.03]
Nikpay et al. 2015	0.021	0.004	50.0%	1.02 [1.01, 1.03]
**Total (95% CI)**			**100.0%**	**1.02 [1.02, 1.03]**

Heterogeneity: Chi² = 0.00, df = 1 (P = 1.00); I² = 0%
Test for overall effect: Z = 7.42 (P < 0.00001)

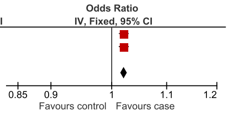

rs194937

Study or Subgroup	log[Odds Ratio]	SE	Weight	Odds Ratio IV, Fixed, 95% CI
Nelson et al. 2017	0.021	0.004	50.0%	1.02 [1.01, 1.03]
van der Harst et al. 2018	0.016	0.004	50.0%	1.02 [1.01, 1.02]
**Total (95% CI)**			**100.0%**	**1.02 [1.01, 1.02]**

Heterogeneity: Chi² = 0.78, df = 1 (P = 0.38); I² = 0%
Test for overall effect: Z = 6.54 (P < 0.00001)

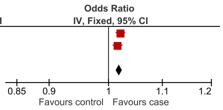

rs1964272

Study or Subgroup	log[Odds Ratio]	SE	Weight	Odds Ratio IV, Fixed, 95% CI
Charmet et al. 2018	-0.022	0.036	1.2%	0.98 [0.91, 1.05]
Nelson et al. 2017	0.02	0.004	98.8%	1.02 [1.01, 1.03]
**Total (95% CI)**			**100.0%**	**1.02 [1.01, 1.03]**

Heterogeneity: Chi² = 1.34, df = 1 (P = 0.25); I² = 26%
Test for overall effect: Z = 4.90 (P < 0.00001)

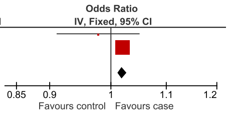

rs1994016

Study or Subgroup	log[Odds Ratio]	SE	Weight	Odds Ratio IV, Random, 95% CI
Qi et al. 2013	0.017	0.029	39.4%	1.02 [0.96, 1.08]
Reilly et al. 2011	0.076	0.01	60.6%	1.08 [1.06, 1.10]
**Total (95% CI)**			**100.0%**	**1.05 [1.00, 1.12]**

Heterogeneity: Tau² = 0.00; Chi² = 3.70, df = 1 (P = 0.05); I² = 73%
Test for overall effect: Z = 1.83 (P = 0.07)

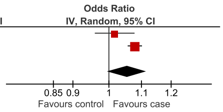

rs199921354

Study or Subgroup	log[Odds Ratio]	SE	Weight	Odds Ratio IV, Fixed, 95% CI
Yamada et al. 2018 (a)	-2	0.255	47.6%	0.14 [0.08, 0.22]
Yamada et al. 2018 (b)	-1.699	0.243	52.4%	0.18 [0.11, 0.29]
**Total (95% CI)**			**100.0%**	**0.16 [0.11, 0.22]**

Heterogeneity: Chi² = 0.73, df = 1 (P = 0.39); I² = 0%
Test for overall effect: Z = 10.47 (P < 0.00001)

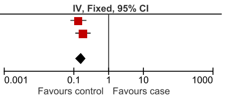

rs2000999

Study or Subgroup	log[Odds Ratio]	SE	Weight	Odds Ratio IV, Fixed, 95% CI
Matsunaga et al. 2020	0.025	0.004	50.0%	1.03 [1.02, 1.03]
van der Harst et al. 2018	0.023	0.004	50.0%	1.02 [1.02, 1.03]
**Total (95% CI)**			**100.0%**	**1.02 [1.02, 1.03]**

Heterogeneity: Chi² = 0.13, df = 1 (P = 0.72); I² = 0%
Test for overall effect: Z = 8.49 (P < 0.00001)

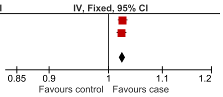

rs200787930

Study or Subgroup	log[Odds Ratio]	SE	Weight	Odds Ratio IV, Fixed, 95% CI
Yamada et al. 2018 (a)	-2	0.255	47.6%	0.14 [0.08, 0.22]
Yamada et al. 2018 (b)	-1.699	0.243	52.4%	0.18 [0.11, 0.29]
**Total (95% CI)**			**100.0%**	**0.16 [0.11, 0.22]**

Heterogeneity: Chi² = 0.73, df = 1 (P = 0.39); I² = 0%
Test for overall effect: Z = 10.47 (P < 0.00001)

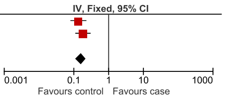

rs2011559

Study or Subgroup	log[Odds Ratio]	SE	Weight	Odds Ratio IV, Random, 95% CI
Nelson et al. 2017	0.021	0.005	45.5%	1.02 [1.01, 1.03]
van der Harst et al. 2018	0.011	0.004	54.5%	1.01 [1.00, 1.02]
**Total (95% CI)**			**100.0%**	**1.02 [1.01, 1.03]**

Heterogeneity: Tau² = 0.00; Chi² = 2.44, df = 1 (P = 0.12); I² = 59%
Test for overall effect: Z = 3.12 (P = 0.002)

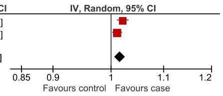

rs2023938

Study or Subgroup	log[Odds Ratio]	SE	Weight	Odds Ratio IV, Random, 95% CI
Charmet et al. 2018	0.06	0.057	3.2%	1.06 [0.95, 1.19]
Fall et al. 2018	0.011	0.003	50.0%	1.01 [1.01, 1.02]
van der Harst et al. 2018	0.033	0.005	46.7%	1.03 [1.02, 1.04]
**Total (95% CI)**			**100.0%**	**1.02 [1.00, 1.04]**

Heterogeneity: Tau² = 0.00; Chi² = 14.81, df = 2 (P = 0.0006); I² = 86%
Test for overall effect: Z = 2.16 (P = 0.03)

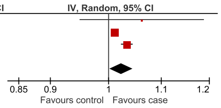

rs2047009

Study or Subgroup	log[Odds Ratio]	SE	Weight	Odds Ratio IV, Random, 95% CI
Dichgans et al. 2014	-0.025	0.006	48.5%	0.98 [0.96, 0.99]
Fall et al. 2018	0.009	0.001	51.5%	1.01 [1.01, 1.01]
**Total (95% CI)**			**100.0%**	**0.99 [0.96, 1.03]**

Heterogeneity: Tau² = 0.00; Chi² = 31.24, df = 1 (P < 0.00001); I² = 97%
Test for overall effect: Z = 0.44 (P = 0.66)

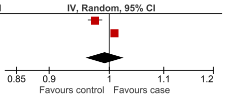

rs2048327

Study or Subgroup	log[Odds Ratio]	SE	Weight	Odds Ratio IV, Random, 95% CI
Charmet et al. 2018	0	0.037	2.7%	1.00 [0.93, 1.08]
Dichgans et al. 2014	0.029	0.005	37.9%	1.03 [1.02, 1.04]
Fall et al. 2018	0.013	0.002	47.5%	1.01 [1.01, 1.02]
Han et al. 2017	0.021	0.016	11.8%	1.02 [0.99, 1.05]
**Total (95% CI)**			**100.0%**	**1.02 [1.01, 1.03]**

Heterogeneity: Tau² = 0.00; Chi² = 9.13, df = 3 (P = 0.03); I² = 67%
Test for overall effect: Z = 3.12 (P = 0.002)

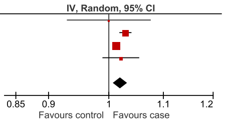

rs2072134

Study or Subgroup	log[Odds Ratio]	SE	Weight	Odds Ratio IV, Fixed, 95% CI
Yamada et al. 2018 (a)	0.233	0.089	44.7%	1.26 [1.06, 1.50]
Yamada et al. 2018 (b)	0.179	0.08	55.3%	1.20 [1.02, 1.40]
**Total (95% CI)**			**100.0%**	**1.23 [1.09, 1.38]**

Heterogeneity: Chi² = 0.20, df = 1 (P = 0.65); I² = 0%
Test for overall effect: Z = 3.41 (P = 0.0006)

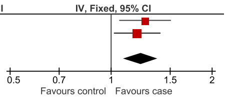

rs2074356

Study or Subgroup	log[Odds Ratio]	SE	Weight	Odds Ratio IV, Random, 95% CI
Lee et al. 2013	0.152	0.023	37.8%	1.16 [1.11, 1.22]
Yamada et al. 2018 (a)	0.394	0.062	30.3%	1.48 [1.31, 1.67]
Yamada et al. 2018 (b)	0.246	0.055	31.9%	1.28 [1.15, 1.42]
**Total (95% CI)**			**100.0%**	**1.29 [1.12, 1.49]**

Heterogeneity: Tau² = 0.01; Chi² = 14.59, df = 2 (P = 0.0007); I² = 86%
Test for overall effect: Z = 3.57 (P = 0.0004)

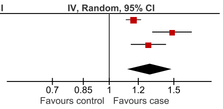

rs2075650

Study or Subgroup	log[Odds Ratio]	SE	Weight	Odds Ratio IV, Random, 95% CI
Charmet et al. 2018	0.131	0.048	19.3%	1.14 [1.04, 1.25]
Fall et al. 2018	0.017	0.002	47.5%	1.02 [1.01, 1.02]
Han et al. 2017	0.057	0.026	33.3%	1.06 [1.01, 1.11]
**Total (95% CI)**			**100.0%**	**1.05 [1.00, 1.11]**

Heterogeneity: Tau² = 0.00; Chi² = 7.96, df = 2 (P = 0.02); I² = 75%
Test for overall effect: Z = 1.91 (P = 0.06)

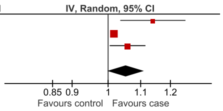

rs2107595

Study or Subgroup	log[Odds Ratio]	SE	Weight	Odds Ratio IV, Fixed, 95% CI
Charmet et al. 2018	0.057	0.046	0.2%	1.06 [0.97, 1.16]
Dichgans et al. 2014	0.021	0.008	5.3%	1.02 [1.01, 1.04]
Han et al. 2017	0.021	0.016	1.3%	1.02 [0.99, 1.05]
Matsunaga et al. 2020	0.029	0.004	21.1%	1.03 [1.02, 1.04]
Nelson et al. 2017	0.033	0.004	21.1%	1.03 [1.03, 1.04]
Nikpay et al. 2015	0.033	0.005	13.5%	1.03 [1.02, 1.04]
van der Harst et al. 2018	0.035	0.003	37.5%	1.04 [1.03, 1.04]
**Total (95% CI)**			**100.0%**	**1.03 [1.03, 1.04]**

Heterogeneity: Chi² = 4.32, df = 6 (P = 0.63); I² = 0%
Test for overall effect: Z = 17.50 (P < 0.00001)

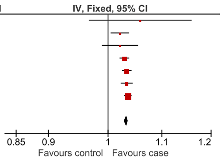

rs2128739

Study or Subgroup	log[Odds Ratio]	SE	Weight	Odds Ratio IV, Fixed, 95% CI
Nikpay et al. 2015	0.029	0.004	36.0%	1.03 [1.02, 1.04]
van der Harst et al. 2018	0.032	0.003	64.0%	1.03 [1.03, 1.04]
**Total (95% CI)**			**100.0%**	**1.03 [1.03, 1.04]**

Heterogeneity: Chi² = 0.36, df = 1 (P = 0.55); I² = 0%
Test for overall effect: Z = 12.88 (P < 0.00001)

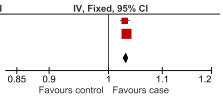

rs2153219

Study or Subgroup	log[Odds Ratio]	SE	Weight	Odds Ratio IV, Fixed, 95% CI
Nelson et al. 2017	0.022	0.005	39.0%	1.02 [1.01, 1.03]
van der Harst et al. 2018	0.019	0.004	61.0%	1.02 [1.01, 1.03]
**Total (95% CI)**			**100.0%**	**1.02 [1.01, 1.03]**

Heterogeneity: Chi² = 0.22, df = 1 (P = 0.64); I² = 0%
Test for overall effect: Z = 6.46 (P < 0.00001)

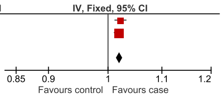

rs216172

Study or Subgroup	log[Odds Ratio]	SE	Weight	Odds Ratio IV, Random, 95% CI
Charmet et al. 2018	0.053	0.036	5.5%	1.05 [0.98, 1.13]
Fall et al. 2018	0.004	0.002	30.3%	1.00 [1.00, 1.01]
Han et al. 2017	0.017	0.017	15.1%	1.02 [0.98, 1.05]
Lee et al. 2013	0.045	0.02	12.7%	1.05 [1.01, 1.09]
Qi et al. 2013	-0.013	0.03	7.3%	0.99 [0.93, 1.05]
Schunkert et al. 2011	0.029	0.004	29.1%	1.03 [1.02, 1.04]
**Total (95% CI)**			**100.0%**	**1.02 [1.00, 1.04]**

Heterogeneity: Tau² = 0.00; Chi² = 36.69, df = 5 (P < 0.00001); I² = 86%
Test for overall effect: Z = 2.14 (P = 0.03)

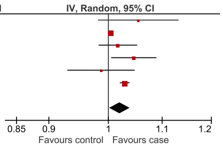

rs220702

Study or Subgroup	log[Odds Ratio]	SE	Weight	Odds Ratio IV, Random, 95% CI
Aoki et al. 2011	0.066	0.031	52.2%	1.07 [1.01, 1.14]
Kertai et al. 2015	-0.335	0.089	47.8%	0.72 [0.60, 0.85]
**Total (95% CI)**			**100.0%**	**0.88 [0.60, 1.31]**

Heterogeneity: Tau² = 0.08; Chi² = 18.10, df = 1 (P < 0.0001); I² = 94%
Test for overall effect: Z = 0.63 (P = 0.53)

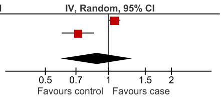

rs2241714

Study or Subgroup	log[Odds Ratio]	SE	Weight	Odds Ratio IV, Fixed, 95% CI
Matsunaga et al. 2020	0.021	0.003	50.0%	1.02 [1.02, 1.03]
van der Harst et al. 2018	0.02	0.003	50.0%	1.02 [1.01, 1.03]
**Total (95% CI)**			**100.0%**	**1.02 [1.02, 1.02]**

Heterogeneity: Chi² = 0.06, df = 1 (P = 0.81); I² = 0%
Test for overall effect: Z = 9.66 (P < 0.00001)

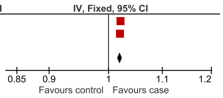

rs2244608

Study or Subgroup	log[Odds Ratio]	SE	Weight	Odds Ratio IV, Fixed, 95% CI
Klarin et al. 2017	0.021	0.004	18.0%	1.02 [1.01, 1.03]
Matsunaga et al. 2020	0.021	0.003	32.0%	1.02 [1.02, 1.03]
Nelson et al. 2017	0.022	0.004	18.0%	1.02 [1.01, 1.03]
van der Harst et al. 2018	0.022	0.003	32.0%	1.02 [1.02, 1.03]
**Total (95% CI)**			**100.0%**	**1.02 [1.02, 1.03]**

Heterogeneity: Chi² = 0.09, df = 3 (P = 0.99); I² = 0%
Test for overall effect: Z = 12.67 (P < 0.00001)

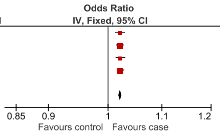

rs2246833

Study or Subgroup	log[Odds Ratio]	SE	Weight	Odds Ratio IV, Fixed, 95% CI
Dichgans et al. 2014	0.029	0.006	64.0%	1.03 [1.02, 1.04]
Wild et al. 2011	0.041	0.008	36.0%	1.04 [1.03, 1.06]
**Total (95% CI)**			**100.0%**	**1.03 [1.02, 1.04]**

Heterogeneity: Chi² = 1.44, df = 1 (P = 0.23); I² = 31%
Test for overall effect: Z = 6.94 (P < 0.00001)

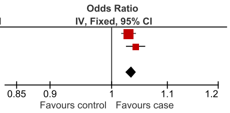

rs2246942

Study or Subgroup	log[Odds Ratio]	SE	Weight	Odds Ratio IV, Fixed, 95% CI
Nelson et al. 2017	0.033	0.004	36.0%	1.03 [1.03, 1.04]
van der Harst et al. 2018	0.031	0.003	64.0%	1.03 [1.03, 1.04]
**Total (95% CI)**			**100.0%**	**1.03 [1.03, 1.04]**

Heterogeneity: Chi² = 0.16, df = 1 (P = 0.69); I² = 0%
Test for overall effect: Z = 13.22 (P < 0.00001)

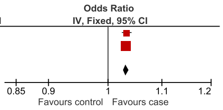

rs2252641

Study or Subgroup	log[Odds Ratio]	SE	Weight	Odds Ratio IV, Random, 95% CI
Charmet et al. 2018	0.004	0.035	1.8%	1.00 [0.94, 1.08]
Dichgans et al. 2014	0.025	0.006	22.5%	1.03 [1.01, 1.04]
Fall et al. 2018	0.004	0.002	33.0%	1.00 [1.00, 1.01]
Han et al. 2017	0.013	0.018	5.8%	1.01 [0.98, 1.05]
Hirokawa et al. 2015	0	0.023	3.8%	1.00 [0.96, 1.05]
van der Harst et al. 2018	0.016	0.002	33.0%	1.02 [1.01, 1.02]
**Total (95% CI)**			**100.0%**	**1.01 [1.00, 1.02]**

Heterogeneity: Tau² = 0.00; Chi² = 24.19, df = 5 (P = 0.0002); I² = 79%
Test for overall effect: Z = 2.75 (P = 0.006)

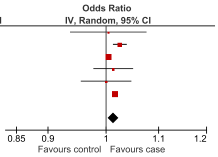

rs2257129

Study or Subgroup	log[Odds Ratio]	SE	Weight	Odds Ratio IV, Fixed, 95% CI
Matsunaga et al. 2020	0.033	0.006	43.5%	1.03 [1.02, 1.05]
Nelson et al. 2017	0.041	0.008	24.5%	1.04 [1.03, 1.06]
van der Harst et al. 2018	0.036	0.007	32.0%	1.04 [1.02, 1.05]
**Total (95% CI)**			**100.0%**	**1.04 [1.03, 1.04]**

Heterogeneity: Chi² = 0.64, df = 2 (P = 0.73); I² = 0%
Test for overall effect: Z = 9.07 (P < 0.00001)

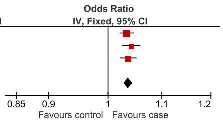

rs2259816

Study or Subgroup	log[Odds Ratio]	SE	Weight	Odds Ratio IV, Fixed, 95% CI
Erdmann et al. 2009	0.033	0.006	96.2%	1.03 [1.02, 1.05]
Qi et al. 2013	0.064	0.03	3.8%	1.07 [1.01, 1.13]
**Total (95% CI)**			**100.0%**	**1.03 [1.02, 1.05]**

Heterogeneity: Chi² = 1.03, df = 1 (P = 0.31); I² = 3%
Test for overall effect: Z = 5.81 (P < 0.00001)

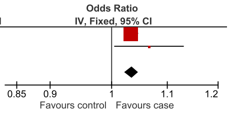

rs2306374

Study or Subgroup	log[Odds Ratio]	SE	Weight	Odds Ratio IV, Random, 95% CI
Hirokawa et al. 2015	0	0.033	5.5%	1.00 [0.94, 1.07]
Lee et al. 2013	-0.013	0.006	32.2%	0.99 [0.98, 1.00]
Schunkert et al. 2011	-0.049	0.009	26.7%	0.95 [0.94, 0.97]
van der Harst et al. 2018	-0.032	0.004	35.5%	0.97 [0.96, 0.98]
**Total (95% CI)**			**100.0%**	**0.97 [0.96, 0.99]**

Heterogeneity: Tau² = 0.00; Chi² = 13.37, df = 3 (P = 0.004); I² = 78%
Test for overall effect: Z = 3.41 (P = 0.0006)

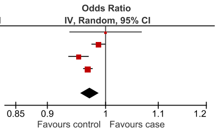

rs2315065

Study or Subgroup	log[Odds Ratio]	SE	Weight	Odds Ratio IV, Random, 95% CI
Nikpay et al. 2015	0.124	0.011	51.9%	1.13 [1.11, 1.16]
Wei et al. 2018	0.079	0.014	48.1%	1.08 [1.05, 1.11]
**Total (95% CI)**			**100.0%**	**1.11 [1.06, 1.16]**

Heterogeneity: Tau² = 0.00; Chi² = 6.39, df = 1 (P = 0.01); I² = 84%
Test for overall effect: Z = 4.55 (P < 0.00001)

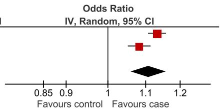

rs2327429

Study or Subgroup	log[Odds Ratio]	SE	Weight	Odds Ratio IV, Random, 95% CI
Matsunaga et al. 2020	0.057	0.008	45.9%	1.06 [1.04, 1.08]
van der Harst et al. 2018	0.031	0.003	54.1%	1.03 [1.03, 1.04]
**Total (95% CI)**			**100.0%**	**1.04 [1.02, 1.07]**

Heterogeneity: Tau² = 0.00; Chi² = 9.26, df = 1 (P = 0.002); I² = 89%
Test for overall effect: Z = 3.31 (P = 0.0009)

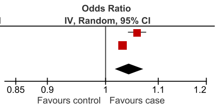

rs2383206

Study or Subgroup	log[Odds Ratio]	SE	Weight	Odds Ratio IV, Fixed, 95% CI
Aoki et al. 2011	0.046	0.023	10.3%	1.05 [1.00, 1.10]
Han et al. 2017	0.084	0.014	27.8%	1.09 [1.06, 1.12]
Lee et al. 2013	0.1	0.018	16.8%	1.11 [1.07, 1.14]
McPherson et al. 2007	0.093	0.011	45.1%	1.10 [1.07, 1.12]
**Total (95% CI)**			**100.0%**	**1.09 [1.08, 1.11]**

Heterogeneity: Chi² = 4.04, df = 3 (P = 0.26); I² = 26%
Test for overall effect: Z = 11.76 (P < 0.00001)

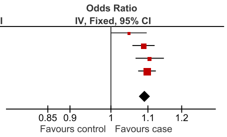

rs2383207

Study or Subgroup	log[Odds Ratio]	SE	Weight	Odds Ratio IV, Fixed, 95% CI
Han et al. 2017	0.081	0.014	41.1%	1.08 [1.06, 1.11]
Helgadottir et al. 2007	0.097	0.012	56.0%	1.10 [1.08, 1.13]
Yamada et al. 2018 (b)	0.125	0.053	2.9%	1.13 [1.02, 1.26]
**Total (95% CI)**			**100.0%**	**1.10 [1.08, 1.11]**

Heterogeneity: Chi² = 1.17, df = 2 (P = 0.56); I² = 0%
Test for overall effect: Z = 10.16 (P < 0.00001)

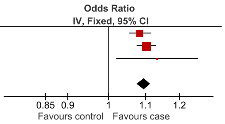

rs246600

Study or Subgroup	log[Odds Ratio]	SE	Weight	Odds Ratio IV, Fixed, 95% CI
Charmet et al. 2018	-0.009	0.035	0.3%	0.99 [0.93, 1.06]
van der Harst et al. 2018	0.021	0.002	99.7%	1.02 [1.02, 1.03]
**Total (95% CI)**			**100.0%**	**1.02 [1.02, 1.03]**

Heterogeneity: Chi² = 0.73, df = 1 (P = 0.39); I² = 0%
Test for overall effect: Z = 10.47 (P < 0.00001)

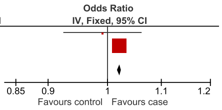

rs2487928

Study or Subgroup	log[Odds Ratio]	SE	Weight	Odds Ratio IV, Fixed, 95% CI
Charmet et al. 2018	0.033	0.035	0.5%	1.03 [0.97, 1.11]
Nikpay et al. 2015	0.025	0.004	35.8%	1.03 [1.02, 1.03]
van der Harst et al. 2018	0.024	0.003	63.7%	1.02 [1.02, 1.03]
**Total (95% CI)**			**100.0%**	**1.02 [1.02, 1.03]**

Heterogeneity: Chi² = 0.10, df = 2 (P = 0.95); I² = 0%
Test for overall effect: Z = 10.19 (P < 0.00001)

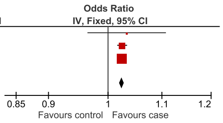

rs2505083

Study or Subgroup	log[Odds Ratio]	SE	Weight	Odds Ratio IV, Random, 95% CI
Dichgans et al. 2014	0.029	0.006	26.8%	1.03 [1.02, 1.04]
Fall et al. 2018	0.009	0.001	32.3%	1.01 [1.01, 1.01]
Hirokawa et al. 2015	-0.018	0.025	6.9%	0.98 [0.94, 1.03]
Peden et al. 2011	0.029	0.005	28.3%	1.03 [1.02, 1.04]
Qi et al. 2013	0.004	0.028	5.8%	1.00 [0.95, 1.06]
**Total (95% CI)**			**100.0%**	**1.02 [1.00, 1.03]**

Heterogeneity: Tau² = 0.00; Chi² = 26.72, df = 4 (P < 0.0001); I² = 85%
Test for overall effect: Z = 2.41 (P = 0.02)

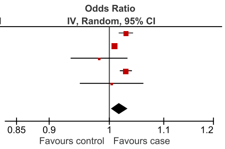

rs2519093

Study or Subgroup	log[Odds Ratio]	SE	Weight	Odds Ratio IV, Fixed, 95% CI
Charmet et al. 2018	-0.009	0.045	0.5%	0.99 [0.91, 1.08]
Nikpay et al. 2015	0.033	0.005	38.8%	1.03 [1.02, 1.04]
van der Harst et al. 2018	0.028	0.004	60.7%	1.03 [1.02, 1.04]
**Total (95% CI)**			**100.0%**	**1.03 [1.02, 1.04]**

Heterogeneity: Chi² = 1.36, df = 2 (P = 0.51); I² = 0%
Test for overall effect: Z = 9.55 (P < 0.00001)

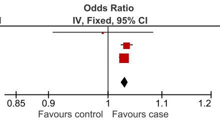

rs2523644

Study or Subgroup	log[Odds Ratio]	SE	Weight	Odds Ratio IV, Fixed, 95% CI
Yamada et al. 2018 (a)	0.246	0.047	44.4%	1.28 [1.17, 1.40]
Yamada et al. 2018 (b)	0.201	0.042	55.6%	1.22 [1.13, 1.33]
**Total (95% CI)**			**100.0%**	**1.25 [1.17, 1.33]**

Heterogeneity: Chi² = 0.51, df = 1 (P = 0.48); I² = 0%
Test for overall effect: Z = 7.06 (P < 0.00001)

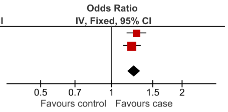

rs2571445

Study or Subgroup	log[Odds Ratio]	SE	Weight	Odds Ratio IV, Fixed, 95% CI
Charmet et al. 2018	0.021	0.036	0.3%	1.02 [0.95, 1.10]
van der Harst et al. 2018	0.018	0.002	99.7%	1.02 [1.01, 1.02]
**Total (95% CI)**			**100.0%**	**1.02 [1.01, 1.02]**

Heterogeneity: Chi² = 0.01, df = 1 (P = 0.93); I² = 0%
Test for overall effect: Z = 9.02 (P < 0.00001)

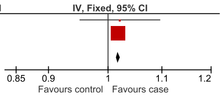

rs2596548

Study or Subgroup	log[Odds Ratio]	SE	Weight	Odds Ratio IV, Fixed, 95% CI
Yamada et al. 2018 (a)	0.31	0.054	45.2%	1.36 [1.23, 1.52]
Yamada et al. 2018 (b)	0.246	0.049	54.8%	1.28 [1.16, 1.41]
**Total (95% CI)**			**100.0%**	**1.32 [1.23, 1.41]**

Heterogeneity: Chi² = 0.77, df = 1 (P = 0.38); I² = 0%
Test for overall effect: Z = 7.58 (P < 0.00001)

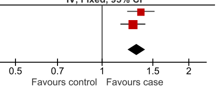

rs2616407

Study or Subgroup	log[Odds Ratio]	SE	Weight	Odds Ratio IV, Fixed, 95% CI
Nelson et al. 2017	0.021	0.004	50.0%	1.02 [1.01, 1.03]
van der Harst et al. 2018	0.017	0.004	50.0%	1.02 [1.01, 1.03]
**Total (95% CI)**			**100.0%**	**1.02 [1.01, 1.02]**

Heterogeneity: Chi² = 0.50, df = 1 (P = 0.48); I² = 0%
Test for overall effect: Z = 6.72 (P < 0.00001)

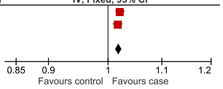

rs264

Study or Subgroup	log[Odds Ratio]	SE	Weight	Odds Ratio IV, Random, 95% CI
Charmet et al. 2018	-0.027	0.048	2.1%	0.97 [0.89, 1.07]
Dichgans et al. 2014	0.046	0.009	23.5%	1.05 [1.03, 1.07]
Fall et al. 2018	0.013	0.004	33.4%	1.01 [1.01, 1.02]
Hirokawa et al. 2015	0.004	0.023	7.7%	1.00 [0.96, 1.05]
van der Harst et al. 2018	0.027	0.004	33.4%	1.03 [1.02, 1.04]
**Total (95% CI)**			**100.0%**	**1.02 [1.01, 1.04]**

Heterogeneity: Tau² = 0.00; Chi² = 15.39, df = 4 (P = 0.004); I² = 74%
Test for overall effect: Z = 3.33 (P = 0.0009)

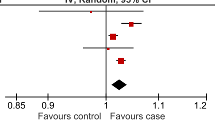

rs2681472

Study or Subgroup	log[Odds Ratio]	SE	Weight	Odds Ratio IV, Fixed, 95% CI
Han et al. 2017	0.017	0.016	3.7%	1.02 [0.99, 1.05]
Nelson et al. 2017	0.029	0.004	58.7%	1.03 [1.02, 1.04]
Nikpay et al. 2015	0.033	0.005	37.6%	1.03 [1.02, 1.04]
**Total (95% CI)**			**100.0%**	**1.03 [1.02, 1.04]**

Heterogeneity: Chi² = 1.08, df = 2 (P = 0.58); I² = 0%
Test for overall effect: Z = 9.81 (P < 0.00001)

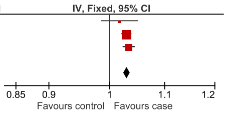

rs2727020

Study or Subgroup	log[Odds Ratio]	SE	Weight	Odds Ratio IV, Fixed, 95% CI
Nelson et al. 2017	0.018	0.005	26.5%	1.02 [1.01, 1.03]
van der Harst et al. 2018	0.013	0.003	73.5%	1.01 [1.01, 1.02]
**Total (95% CI)**			**100.0%**	**1.01 [1.01, 1.02]**

Heterogeneity: Chi² = 0.74, df = 1 (P = 0.39); I² = 0%
Test for overall effect: Z = 5.57 (P < 0.00001)

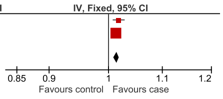

rs273909

Study or Subgroup	log[Odds Ratio]	SE	Weight	Odds Ratio IV, Random, 95% CI
Charmet et al. 2018	-0.027	0.057	1.3%	0.97 [0.87, 1.09]
Dichgans et al. 2014	0.029	0.009	18.8%	1.03 [1.01, 1.05]
Fall et al. 2018	0.004	0.002	27.6%	1.00 [1.00, 1.01]
Han et al. 2017	-0.013	0.033	3.6%	0.99 [0.93, 1.05]
Nikpay et al. 2015	0.025	0.006	23.1%	1.03 [1.01, 1.04]
van der Harst et al. 2018	0.023	0.004	25.7%	1.02 [1.02, 1.03]
**Total (95% CI)**			**100.0%**	**1.02 [1.00, 1.03]**

Heterogeneity: Tau² = 0.00; Chi² = 31.16, df = 5 (P < 0.00001); I² = 84%
Test for overall effect: Z = 2.59 (P = 0.010)

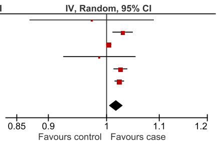

rs2789422

Study or Subgroup	log[Odds Ratio]	SE	Weight	Odds Ratio IV, Fixed, 95% CI
Nelson et al. 2017	-0.018	0.003	50.0%	0.98 [0.98, 0.99]
van der Harst et al. 2018	-0.013	0.003	50.0%	0.99 [0.98, 0.99]
**Total (95% CI)**			**100.0%**	**0.98 [0.98, 0.99]**

Heterogeneity: Chi² = 1.39, df = 1 (P = 0.24); I² = 28%
Test for overall effect: Z = 7.31 (P < 0.00001)

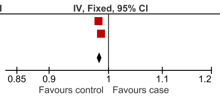

rs2820315

Study or Subgroup	log[Odds Ratio]	SE	Weight	Odds Ratio IV, Fixed, 95% CI
Charmet et al. 2018	-0.086	0.202	0.0%	0.92 [0.62, 1.36]
Nelson et al. 2017	0.017	0.003	30.8%	1.02 [1.01, 1.02]
van der Harst et al. 2018	0.016	0.002	69.2%	1.02 [1.01, 1.02]
**Total (95% CI)**			**100.0%**	**1.02 [1.01, 1.02]**

Heterogeneity: Chi² = 0.33, df = 2 (P = 0.85); I² = 0%
Test for overall effect: Z = 9.80 (P < 0.00001)

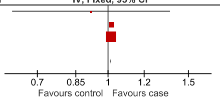

rs2843152

Study or Subgroup	log[Odds Ratio]	SE	Weight	Odds Ratio IV, Fixed, 95% CI
Nelson et al. 2017	0.018	0.005	26.5%	1.02 [1.01, 1.03]
van der Harst et al. 2018	0.018	0.003	73.5%	1.02 [1.01, 1.02]
**Total (95% CI)**			**100.0%**	**1.02 [1.01, 1.02]**

Heterogeneity: Chi² = 0.00, df = 1 (P = 1.00); I² = 0%
Test for overall effect: Z = 7.00 (P < 0.00001)

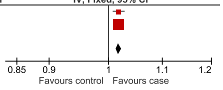

rs28451064

Study or Subgroup	log[Odds Ratio]	SE	Weight	Odds Ratio IV, Fixed, 95% CI
Charmet et al. 2018	0.134	0.055	0.3%	1.14 [1.03, 1.27]
Klarin et al. 2017	0.072	0.013	5.1%	1.07 [1.05, 1.10]
Nelson et al. 2017	0.057	0.006	23.8%	1.06 [1.05, 1.07]
Nikpay et al. 2015	0.057	0.007	17.5%	1.06 [1.04, 1.07]
van der Harst et al. 2018	0.052	0.004	53.4%	1.05 [1.05, 1.06]
**Total (95% CI)**			**100.0%**	**1.06 [1.05, 1.06]**

Heterogeneity: Chi² = 4.52, df = 4 (P = 0.34); I² = 11%
Test for overall effect: Z = 18.91 (P < 0.00001)

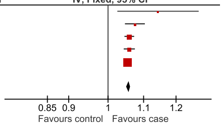

rs2880099

Study or Subgroup	log[Odds Ratio]	SE	Weight	Odds Ratio IV, Random, 95% CI
Kertai et al. 2015	0.314	0.081	46.1%	1.37 [1.17, 1.60]
van der Harst et al. 2018	0.026	0.004	53.9%	1.03 [1.02, 1.03]
**Total (95% CI)**			**100.0%**	**1.17 [0.88, 1.55]**

Heterogeneity: Tau² = 0.04; Chi² = 12.61, df = 1 (P = 0.0004); I² = 92%
Test for overall effect: Z = 1.11 (P = 0.27)

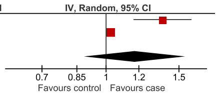

rs288187

Study or Subgroup	log[Odds Ratio]	SE	Weight	Odds Ratio IV, Fixed, 95% CI
Nelson et al. 2017	-0.018	0.006	30.8%	0.98 [0.97, 0.99]
van der Harst et al. 2018	-0.014	0.004	69.2%	0.99 [0.98, 0.99]
**Total (95% CI)**			**100.0%**	**0.98 [0.98, 0.99]**

Heterogeneity: Chi² = 0.31, df = 1 (P = 0.58); I² = 0%
Test for overall effect: Z = 4.58 (P < 0.00001)

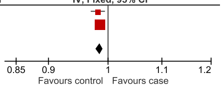

rs2891168

Study or Subgroup	log[Odds Ratio]	SE	Weight	Odds Ratio IV, Random, 95% CI
Charmet et al. 2018	0.064	0.035	2.8%	1.07 [1.00, 1.14]
Erdmann et al. 2011	0.049	0.006	14.1%	1.05 [1.04, 1.06]
Han et al. 2017	0.087	0.014	9.1%	1.09 [1.06, 1.12]
Lee et al. 2013	0.097	0.018	7.1%	1.10 [1.06, 1.14]
Nelson et al. 2017	0.076	0.004	15.2%	1.08 [1.07, 1.09]
Nikpay et al. 2015	0.083	0.005	14.7%	1.09 [1.08, 1.10]
Samani et al. 2007	0.13	0.015	8.6%	1.14 [1.11, 1.17]
van der Harst et al. 2018	0.082	0.003	15.6%	1.09 [1.08, 1.09]
Wild et al. 2011	0.114	0.008	12.9%	1.12 [1.10, 1.14]
**Total (95% CI)**			**100.0%**	**1.09 [1.08, 1.10]**

Heterogeneity: Tau² = 0.00; Chi² = 59.01, df = 8 (P < 0.00001); I² = 86%
Test for overall effect: Z = 13.40 (P < 0.00001)

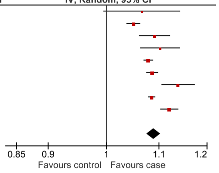

rs2895811

Study or Subgroup	log[Odds Ratio]	SE	Weight	Odds Ratio IV, Random, 95% CI
Dichgans et al. 2014	0.037	0.007	18.7%	1.04 [1.02, 1.05]
Fall et al. 2018	0.004	0.001	24.4%	1.00 [1.00, 1.01]
Han et al. 2017	0.004	0.018	8.0%	1.00 [0.97, 1.04]
Qi et al. 2013	-0.036	0.029	3.8%	0.96 [0.91, 1.02]
Schunkert et al. 2011	0.029	0.005	21.2%	1.03 [1.02, 1.04]
van der Harst et al. 2018	0.019	0.002	24.0%	1.02 [1.02, 1.02]
**Total (95% CI)**			**100.0%**	**1.02 [1.01, 1.03]**

Heterogeneity: Tau² = 0.00; Chi² = 83.39, df = 5 (P < 0.00001); I² = 94%
Test for overall effect: Z = 2.84 (P = 0.005)

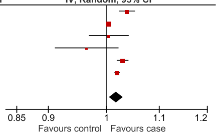

rs2943634

Study or Subgroup	log[Odds Ratio]	SE	Weight	Odds Ratio IV, Random, 95% CI
Kathiresan et al. 2009	-0.027	0.009	34.0%	0.97 [0.96, 0.99]
Samani et al. 2007	0.083	0.016	30.2%	1.09 [1.05, 1.12]
van der Harst et al. 2018	0.018	0.003	35.8%	1.02 [1.01, 1.02]
**Total (95% CI)**			**100.0%**	**1.02 [0.98, 1.07]**

Heterogeneity: Tau² = 0.00; Chi² = 40.79, df = 2 (P < 0.00001); I² = 95%
Test for overall effect: Z = 1.02 (P = 0.31)

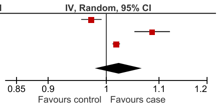

rs2954029

Study or Subgroup	log[Odds Ratio]	SE	Weight	Odds Ratio IV, Random, 95% CI
Charmet et al. 2018	0.021	0.035	2.2%	1.02 [0.95, 1.09]
Dichgans et al. 2014	0.027	0.006	19.3%	1.03 [1.02, 1.04]
Fall et al. 2018	0.009	0.002	24.3%	1.01 [1.01, 1.01]
Han et al. 2017	0.009	0.015	8.7%	1.01 [0.98, 1.04]
Nelson et al. 2017	0.025	0.004	22.1%	1.03 [1.02, 1.03]
van der Harst et al. 2018	0.029	0.003	23.3%	1.03 [1.02, 1.04]
**Total (95% CI)**			**100.0%**	**1.02 [1.01, 1.03]**

Heterogeneity: $Tau^2 = 0.00$; $Chi^2 = 39.06$, df = 5 ($P < 0.00001$); $I^2 = 87\%$
Test for overall effect: Z = 3.81 (P = 0.0001)

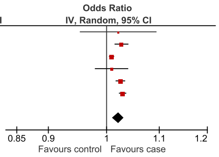

rs2972146

Study or Subgroup	log[Odds Ratio]	SE	Weight	Odds Ratio IV, Fixed, 95% CI
Klarin et al. 2017	0.025	0.003	64.0%	1.03 [1.02, 1.03]
Nelson et al. 2017	0.021	0.004	36.0%	1.02 [1.01, 1.03]
**Total (95% CI)**			**100.0%**	**1.02 [1.02, 1.03]**

Heterogeneity: $Chi^2 = 0.64$, df = 1 (P = 0.42); $I^2 = 0\%$
Test for overall effect: Z = 9.82 ($P < 0.00001$)

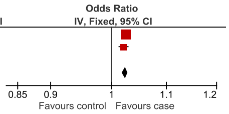

rs3130683

Study or Subgroup	log[Odds Ratio]	SE	Weight	Odds Ratio IV, Random, 95% CI
Charmet et al. 2018	-0.161	0.091	0.6%	0.85 [0.71, 1.02]
Nelson et al. 2017	0.033	0.006	44.6%	1.03 [1.02, 1.05]
van der Harst et al. 2018	0.029	0.004	54.9%	1.03 [1.02, 1.04]
**Total (95% CI)**			**100.0%**	**1.03 [1.02, 1.04]**

Heterogeneity: $Tau^2 = 0.00$; $Chi^2 = 4.72$, df = 2 (P = 0.09); $I^2 = 58\%$
Test for overall effect: Z = 4.32 ($P < 0.0001$)

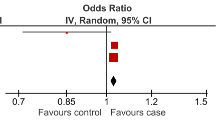

rs3133293

Study or Subgroup	log[Odds Ratio]	SE	Weight	Odds Ratio IV, Fixed, 95% CI
Nelson et al. 2017	-0.018	0.005	26.5%	0.98 [0.97, 0.99]
van der Harst et al. 2018	-0.013	0.003	73.5%	0.99 [0.98, 0.99]
**Total (95% CI)**			**100.0%**	**0.99 [0.98, 0.99]**

Heterogeneity: $Chi^2 = 0.74$, df = 1 (P = 0.39); $I^2 = 0\%$
Test for overall effect: Z = 5.57 ($P < 0.00001$)

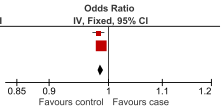

rs3184504

Study or Subgroup	log[Odds Ratio]	SE	Weight	Odds Ratio IV, Random, 95% CI
Fall et al. 2018	0.009	0.004	28.6%	1.01 [1.00, 1.02]
Peden et al. 2011	0.021	0.012	16.1%	1.02 [1.00, 1.05]
Schunkert et al. 2011	0.029	0.006	25.5%	1.03 [1.02, 1.04]
van der Harst et al. 2018	0.032	0.003	29.8%	1.03 [1.03, 1.04]
**Total (95% CI)**			**100.0%**	**1.02 [1.01, 1.04]**

Heterogeneity: $Tau^2 = 0.00$; $Chi^2 = 21.91$, df = 3 ($P < 0.0001$); $I^2 = 86\%$
Test for overall effect: Z = 3.33 (P = 0.0009)

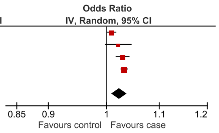

rs3217992

Study or Subgroup	log[Odds Ratio]	SE	Weight	Odds Ratio IV, Random, 95% CI
Charmet et al. 2018	0.036	0.035	18.8%	1.04 [0.97, 1.11]
Dichgans et al. 2014	0.068	0.007	39.7%	1.07 [1.06, 1.09]
Fall et al. 2018	0.021	0.002	41.5%	1.02 [1.02, 1.03]
**Total (95% CI)**			**100.0%**	**1.04 [1.00, 1.09]**

Heterogeneity: $Tau^2 = 0.00$; $Chi^2 = 41.79$, df = 2 ($P < 0.00001$); $I^2 = 95\%$
Test for overall effect: Z = 2.07 (P = 0.04)

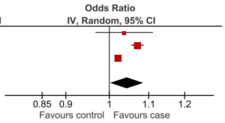

rs328

Study or Subgroup	log[Odds Ratio]	SE	Weight	Odds Ratio IV, Random, 95% CI
Hu et al. 2016	0.21	0.095	36.3%	1.23 [1.02, 1.49]
Matsunaga et al. 2020	0.029	0.005	63.7%	1.03 [1.02, 1.04]
**Total (95% CI)**			**100.0%**	**1.10 [0.93, 1.30]**

Heterogeneity: $Tau^2 = 0.01$; $Chi^2 = 3.62$, df = 1 (P = 0.06); $I^2 = 72\%$
Test for overall effect: Z = 1.09 (P = 0.28)

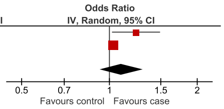

rs35146811

Study or Subgroup	log[Odds Ratio]	SE	Weight	Odds Ratio IV, Fixed, 95% CI
Nelson et al. 2017	0.017	0.003	50.0%	1.02 [1.01, 1.02]
van der Harst et al. 2018	0.015	0.003	50.0%	1.02 [1.01, 1.02]
**Total (95% CI)**			**100.0%**	**1.02 [1.01, 1.02]**

Heterogeneity: $Chi^2 = 0.22$, df = 1 (P = 0.64); $I^2 = 0\%$
Test for overall effect: Z = 7.54 ($P < 0.00001$)

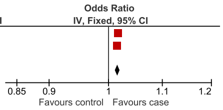

rs35465346

Study or Subgroup	log[Odds Ratio]	SE	Weight	Odds Ratio IV, Fixed, 95% CI
Nelson et al. 2017	-0.022	0.005	39.0%	0.98 [0.97, 0.99]
van der Harst et al. 2018	-0.017	0.004	61.0%	0.98 [0.98, 0.99]
**Total (95% CI)**			**100.0%**	**0.98 [0.98, 0.99]**

Heterogeneity: $Chi^2 = 0.61$, df = 1 (P = 0.43); $I^2 = 0\%$
Test for overall effect: Z = 6.07 ($P < 0.00001$)

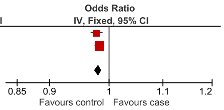

rs35489971

Study or Subgroup	log[Odds Ratio]	SE	Weight	Odds Ratio IV, Fixed, 95% CI
Nelson et al. 2017	0.022	0.005	39.0%	1.02 [1.01, 1.03]
van der Harst et al. 2018	0.019	0.004	61.0%	1.02 [1.01, 1.03]
**Total (95% CI)**			**100.0%**	**1.02 [1.01, 1.03]**

Heterogeneity: $Chi^2 = 0.22$, df = 1 (P = 0.64); $I^2 = 0\%$
Test for overall effect: Z = 6.46 ($P < 0.00001$)

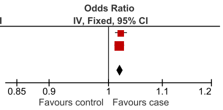

rs35895680

Study or Subgroup	log[Odds Ratio]	SE	Weight	Odds Ratio IV, Fixed, 95% CI
Nikpay et al. 2015	0.025	0.004	36.0%	1.03 [1.02, 1.03]
van der Harst et al. 2018	0.019	0.003	64.0%	1.02 [1.01, 1.03]
**Total (95% CI)**			**100.0%**	**1.02 [1.02, 1.03]**

Heterogeneity: $Chi^2 = 1.44$, df = 1 (P = 0.23); $I^2 = 31\%$
Test for overall effect: Z = 8.82 ($P < 0.00001$)

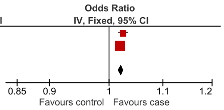

rs3739998

Study or Subgroup	log[Odds Ratio]	SE	Weight	Odds Ratio IV, Random, 95% CI
Erdmann et al. 2011	0.061	0.009	47.6%	1.06 [1.04, 1.08]
van der Harst et al. 2018	0.022	0.003	52.4%	1.02 [1.02, 1.03]
**Total (95% CI)**			**100.0%**	**1.04 [1.00, 1.08]**

Heterogeneity: $Tau^2 = 0.00$; $Chi^2 = 16.90$, df = 1 ($P < 0.0001$); $I^2 = 94\%$
Test for overall effect: Z = 2.08 (P = 0.04)

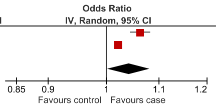

rs3782774

Study or Subgroup	log[Odds Ratio]	SE	Weight	Odds Ratio IV, Fixed, 95% CI
Nelson et al. 2017	0.017	0.003	50.0%	1.02 [1.01, 1.02]
van der Harst et al. 2018	0.014	0.003	50.0%	1.01 [1.01, 1.02]
**Total (95% CI)**			**100.0%**	**1.02 [1.01, 1.02]**

Heterogeneity: $Chi^2 = 0.50$, df = 1 (P = 0.48); $I^2 = 0\%$
Test for overall effect: Z = 7.31 ($P < 0.00001$)

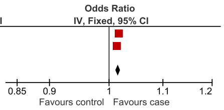

rs3782886

Study or Subgroup	log[Odds Ratio]	SE	Weight	Odds Ratio IV, Random, 95% CI
Aoki et al. 2011	0.157	0.025	17.0%	1.17 [1.11, 1.23]
Hirokawa et al. 2015	-0.164	0.02	17.2%	0.85 [0.82, 0.88]
Lee et al. 2013	0.146	0.022	17.1%	1.16 [1.11, 1.21]
Takeuchi et al. 2012	0.14	0.012	17.4%	1.15 [1.12, 1.18]
Yamada et al. 2018 (a)	0.412	0.058	15.3%	1.51 [1.35, 1.69]
Yamada et al. 2018 (b)	0.23	0.049	15.9%	1.26 [1.14, 1.39]
**Total (95% CI)**			**100.0%**	**1.16 [1.02, 1.32]**

Heterogeneity: $Tau^2 = 0.02$; $Chi^2 = 230.72$, df = 5 ($P < 0.00001$); $I^2 = 98\%$
Test for overall effect: Z = 2.28 (P = 0.02)

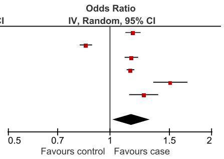

rs3798220

Study or Subgroup	log[Odds Ratio]	SE	Weight	Odds Ratio IV, Random, 95% CI
Charmet et al. 2018	0.131	0.139	8.3%	1.14 [0.87, 1.50]
Fall et al. 2018	0.068	0.006	23.3%	1.07 [1.06, 1.08]
Lee et al. 2013	-0.032	0.006	23.3%	0.97 [0.96, 0.98]
Schunkert et al. 2011	0.179	0.027	21.9%	1.20 [1.13, 1.26]
van der Harst et al. 2018	0.204	0.01	23.2%	1.23 [1.20, 1.25]
**Total (95% CI)**			**100.0%**	**1.11 [1.01, 1.23]**

Heterogeneity: $Tau^2 = 0.01$; $Chi^2 = 456.06$, df = 4 ($P < 0.00001$); $I^2 = 99\%$
Test for overall effect: Z = 2.12 (P = 0.03)

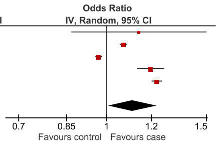

rs3809346

Study or Subgroup	log[Odds Ratio]	SE	Weight	Odds Ratio IV, Fixed, 95% CI
O'Donnell et al. 2011	0.036	0.012	5.9%	1.04 [1.01, 1.06]
van der Harst et al. 2018	0.022	0.003	94.1%	1.02 [1.02, 1.03]
**Total (95% CI)**			**100.0%**	**1.02 [1.02, 1.03]**

Heterogeneity: $Chi^2 = 1.28$, df = 1 (P = 0.26); $I^2 = 22\%$
Test for overall effect: Z = 7.84 ($P < 0.00001$)

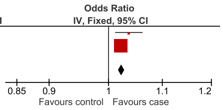

rs3825807

Study or Subgroup	log[Odds Ratio]	SE	Weight	Odds Ratio IV, Random, 95% CI
Lee et al. 2013	-0.027	0.006	37.4%	0.97 [0.96, 0.98]
Qi et al. 2013	0.029	0.03	24.8%	1.03 [0.97, 1.09]
Schunkert et al. 2011	0.033	0.004	37.8%	1.03 [1.03, 1.04]
**Total (95% CI)**			**100.0%**	**1.01 [0.96, 1.06]**

Heterogeneity: $Tau^2 = 0.00$; $Chi^2 = 69.46$, df = 2 ($P < 0.00001$); $I^2 = 97\%$
Test for overall effect: Z = 0.38 (P = 0.70)

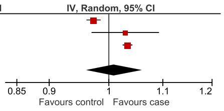

rs383830

Study or Subgroup	log[Odds Ratio]	SE	Weight	Odds Ratio IV, Random, 95% CI
Burton et al. 2007	0.283	0.07	21.0%	1.33 [1.16, 1.52]
Samani et al. 2007	0.017	0.028	36.7%	1.02 [0.96, 1.07]
Schunkert et al. 2011	0.012	0.008	42.3%	1.01 [1.00, 1.03]
**Total (95% CI)**			**100.0%**	**1.07 [0.98, 1.17]**

Heterogeneity: $Tau^2 = 0.00$; $Chi^2 = 14.80$, df = 2 ($P = 0.0006$); $I^2 = 86\%$
Test for overall effect: Z = 1.58 (P = 0.11)

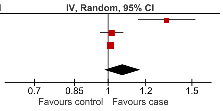

rs3918226

Study or Subgroup	log[Odds Ratio]	SE	Weight	Odds Ratio IV, Random, 95% CI
Charmet et al. 2018	-0.046	0.065	1.9%	0.96 [0.84, 1.08]
Fall et al. 2018	0.013	0.01	22.7%	1.01 [0.99, 1.03]
Nelson et al. 2017	0.053	0.008	25.2%	1.05 [1.04, 1.07]
Nikpay et al. 2015	0.057	0.01	22.7%	1.06 [1.04, 1.08]
van der Harst et al. 2018	0.053	0.006	27.5%	1.05 [1.04, 1.07]
**Total (95% CI)**			**100.0%**	**1.04 [1.03, 1.06]**

Heterogeneity: $Tau^2 = 0.00$; $Chi^2 = 16.17$, df = 4 ($P = 0.003$); $I^2 = 75\%$
Test for overall effect: Z = 4.66 (P < 0.00001)

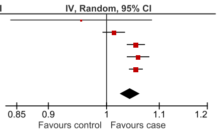

rs3993105

Study or Subgroup	log[Odds Ratio]	SE	Weight	Odds Ratio IV, Fixed, 95% CI
Charmet et al. 2018	0.013	0.039	1.0%	1.01 [0.94, 1.09]
Nelson et al. 2017	0.02	0.004	99.0%	1.02 [1.01, 1.03]
**Total (95% CI)**			**100.0%**	**1.02 [1.01, 1.03]**

Heterogeneity: $Chi^2 = 0.03$, df = 1 ($P = 0.86$); $I^2 = 0\%$
Test for overall effect: Z = 5.01 (P < 0.00001)

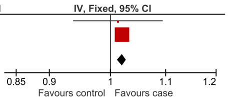

rs41272114

Study or Subgroup	log[Odds Ratio]	SE	Weight	Odds Ratio IV, Fixed, 95% CI
Nelson et al. 2017	-0.056	0.01	39.0%	0.95 [0.93, 0.96]
van der Harst et al. 2018	-0.05	0.008	61.0%	0.95 [0.94, 0.97]
**Total (95% CI)**			**100.0%**	**0.95 [0.94, 0.96]**

Heterogeneity: $Chi^2 = 0.22$, df = 1 ($P = 0.64$); $I^2 = 0\%$
Test for overall effect: Z = 8.38 (P < 0.00001)

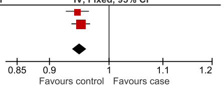

rs421329

Study or Subgroup	log[Odds Ratio]	SE	Weight	Odds Ratio IV, Fixed, 95% CI
Nelson et al. 2017	-0.018	0.003	50.0%	0.98 [0.98, 0.99]
van der Harst et al. 2018	-0.015	0.003	50.0%	0.99 [0.98, 0.99]
**Total (95% CI)**			**100.0%**	**0.98 [0.98, 0.99]**

Heterogeneity: $Chi^2 = 0.50$, df = 1 ($P = 0.48$); $I^2 = 0\%$
Test for overall effect: Z = 7.78 (P < 0.00001)

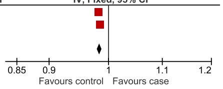

rs4244285

Study or Subgroup	log[Odds Ratio]	SE	Weight	Odds Ratio IV, Fixed, 95% CI
Verma et al. 2020	0.029	0.032	88.1%	1.03 [0.97, 1.10]
Zhong et al. 2017	-0.006	0.087	11.9%	0.99 [0.84, 1.18]
**Total (95% CI)**			**100.0%**	**1.03 [0.97, 1.09]**

Heterogeneity: $Chi^2 = 0.14$, df = 1 ($P = 0.71$); $I^2 = 0\%$
Test for overall effect: Z = 0.83 (P = 0.41)

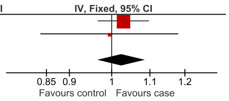

rs425105

Study or Subgroup	log[Odds Ratio]	SE	Weight	Odds Ratio IV, Fixed, 95% CI
Nelson et al. 2017	0.021	0.004	50.0%	1.02 [1.01, 1.03]
van der Harst et al. 2018	0.017	0.004	50.0%	1.02 [1.01, 1.03]
**Total (95% CI)**			**100.0%**	**1.02 [1.01, 1.02]**

Heterogeneity: $Chi^2 = 0.50$, df = 1 ($P = 0.48$); $I^2 = 0\%$
Test for overall effect: Z = 6.72 (P < 0.00001)

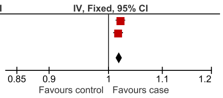

rs4252120

Study or Subgroup	log[Odds Ratio]	SE	Weight	Odds Ratio IV, Random, 95% CI
Charmet et al. 2018	-0.041	0.038	3.0%	0.96 [0.89, 1.03]
Dichgans et al. 2014	0.032	0.006	28.2%	1.03 [1.02, 1.04]
Fall et al. 2018	0.009	0.002	35.0%	1.01 [1.01, 1.01]
van der Harst et al. 2018	0.024	0.003	33.8%	1.02 [1.02, 1.03]
**Total (95% CI)**			**100.0%**	**1.02 [1.01, 1.03]**

Heterogeneity: $Tau^2 = 0.00$; $Chi^2 = 28.19$, df = 3 ($P < 0.00001$); $I^2 = 89\%$
Test for overall effect: Z = 2.80 (P = 0.005)

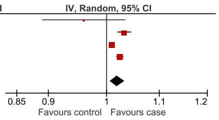

rs4252185

Study or Subgroup	log[Odds Ratio]	SE	Weight	Odds Ratio IV, Fixed, 95% CI
Charmet et al. 2018	0.061	0.064	5.2%	1.06 [0.94, 1.20]
Wei et al. 2018	0.093	0.015	94.8%	1.10 [1.07, 1.13]
**Total (95% CI)**			**100.0%**	**1.10 [1.06, 1.13]**

Heterogeneity: $Chi^2 = 0.24$, df = 1 ($P = 0.63$); $I^2 = 0\%$
Test for overall effect: Z = 6.25 (P < 0.00001)

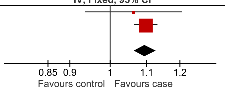

rs4299376

Study or Subgroup	log[Odds Ratio]	SE	Weight	Odds Ratio IV, Fixed, 95% CI
Nelson et al. 2017	-0.025	0.004	36.0%	0.98 [0.97, 0.98]
van der Harst et al. 2018	-0.025	0.003	64.0%	0.98 [0.97, 0.98]
**Total (95% CI)**			**100.0%**	**0.98 [0.97, 0.98]**

Heterogeneity: $Chi^2 = 0.00$, df = 1 ($P = 1.00$); $I^2 = 0\%$
Test for overall effect: Z = 10.42 (P < 0.00001)

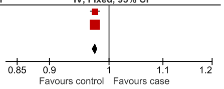

rs4380028

Study or Subgroup	log[Odds Ratio]	SE	Weight	Odds Ratio IV, Fixed, 95% CI
Peden et al. 2011	0.029	0.005	26.3%	1.03 [1.02, 1.04]
Qi et al. 2013	0	0.03	0.7%	1.00 [0.94, 1.06]
van der Harst et al. 2018	0.026	0.003	73.0%	1.03 [1.02, 1.03]
**Total (95% CI)**			**100.0%**	**1.03 [1.02, 1.03]**

Heterogeneity: $Chi^2 = 1.06$, df = 2 ($P = 0.59$); $I^2 = 0\%$
Test for overall effect: Z = 10.38 (P < 0.00001)

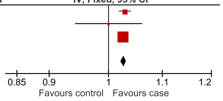

rs4420638

Study or Subgroup	log[Odds Ratio]	SE	Weight	Odds Ratio IV, Fixed, 95% CI
Charmet et al. 2018	0.079	0.044	0.6%	1.08 [0.99, 1.18]
Nikpay et al. 2015	0.041	0.006	30.6%	1.04 [1.03, 1.05]
van der Harst et al. 2018	0.04	0.004	68.8%	1.04 [1.03, 1.05]
**Total (95% CI)**			**100.0%**	**1.04 [1.03, 1.05]**

Heterogeneity: $Chi^2 = 0.79$, df = 2 ($P = 0.67$); $I^2 = 0\%$
Test for overall effect: Z = 12.21 (P < 0.00001)

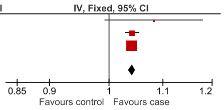

rs445925

Study or Subgroup	log[Odds Ratio]	SE	Weight	Odds Ratio IV, Fixed, 95% CI
Charmet et al. 2018	-0.06	0.056	0.1%	0.94 [0.84, 1.05]
Fall et al. 2018	0.017	0.002	99.9%	1.02 [1.01, 1.02]
**Total (95% CI)**			**100.0%**	**1.02 [1.01, 1.02]**

Heterogeneity: $Chi^2 = 1.89$, df = 1 ($P = 0.17$); $I^2 = 47\%$
Test for overall effect: Z = 8.46 (P < 0.00001)

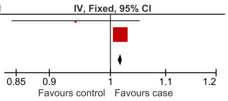

rs46522

Study or Subgroup	log[Odds Ratio]	SE	Weight	Odds Ratio IV, Random, 95% CI
Fall et al. 2018	0.004	0.002	35.0%	1.00 [1.00, 1.01]
Han et al. 2017	0.009	0.016	14.2%	1.01 [0.98, 1.04]
Lee et al. 2013	0.021	0.018	12.2%	1.02 [0.99, 1.06]
Qi et al. 2013	0.029	0.029	6.0%	1.03 [0.97, 1.09]
Schunkert et al. 2011	0.025	0.004	32.7%	1.03 [1.02, 1.03]
**Total (95% CI)**			**100.0%**	**1.02 [1.00, 1.03]**

Heterogeneity: $Tau^2 = 0.00$; $Chi^2 = 23.06$, df = 4 ($P = 0.0001$); $I^2 = 83\%$
Test for overall effect: Z = 1.95 (P = 0.05)

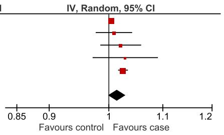

rs4773141

Study or Subgroup	log[Odds Ratio]	SE	Weight	Odds Ratio IV, Fixed, 95% CI
Nelson et al. 2017	0.025	0.004	36.0%	1.03 [1.02, 1.03]
van der Harst et al. 2018	0.026	0.003	64.0%	1.03 [1.02, 1.03]
**Total (95% CI)**			**100.0%**	**1.03 [1.02, 1.03]**

Heterogeneity: $Chi^2 = 0.04$, df = 1 ($P = 0.84$); $I^2 = 0\%$
Test for overall effect: Z = 10.68 (P < 0.00001)

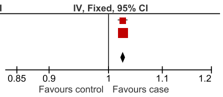

rs4773144

Study or Subgroup	log[Odds Ratio]	SE	Weight	Odds Ratio IV, Random, 95% CI
Charmet et al. 2018	0.004	0.036	5.1%	1.00 [0.94, 1.08]
Dichgans et al. 2014	0.033	0.007	22.8%	1.03 [1.02, 1.05]
Fall et al. 2018	0.004	0.002	26.1%	1.00 [1.00, 1.01]
Lee et al. 2013	0.049	0.017	13.7%	1.05 [1.02, 1.09]
Qi et al. 2013	-0.018	0.029	7.1%	0.98 [0.93, 1.04]
Schunkert et al. 2011	0.029	0.004	25.2%	1.03 [1.02, 1.04]
**Total (95% CI)**			**100.0%**	**1.02 [1.00, 1.04]**

Heterogeneity: Tau² = 0.00; Chi² = 48.39, df = 5 (P < 0.00001); I² = 90%
Test for overall effect: Z = 2.38 (P = 0.02)

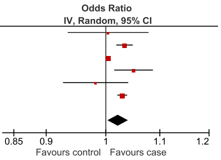

rs4845625

Study or Subgroup	log[Odds Ratio]	SE	Weight	Odds Ratio IV, Random, 95% CI
Dichgans et al. 2014	0.025	0.006	24.9%	1.03 [1.01, 1.04]
Fall et al. 2018	0.004	0.001	32.5%	1.00 [1.00, 1.01]
Han et al. 2017	0.009	0.015	10.9%	1.01 [0.98, 1.04]
van der Harst et al. 2018	0.019	0.002	31.7%	1.02 [1.02, 1.02]
**Total (95% CI)**			**100.0%**	**1.01 [1.00, 1.03]**

Heterogeneity: Tau² = 0.00; Chi² = 53.82, df = 3 (P < 0.00001); I² = 94%
Test for overall effect: Z = 2.39 (P = 0.02)

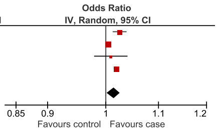

rs4977574

Study or Subgroup	log[Odds Ratio]	SE	Weight	Odds Ratio IV, Random, 95% CI
Erdmann et al. 2011	0.049	0.006	7.9%	1.05 [1.04, 1.06]
Fall et al. 2018	0.025	0.001	8.1%	1.03 [1.02, 1.03]
Han et al. 2017	0.037	0.005	7.9%	1.04 [1.03, 1.05]
Hirokawa et al. 2015	-0.086	0.019	6.4%	0.92 [0.88, 0.95]
Kathiresan et al. 2009	0.107	0.009	7.6%	1.11 [1.09, 1.13]
Lee et al. 2013	0.1	0.018	6.6%	1.11 [1.07, 1.14]
Lettre et al. 2011	0.072	0.052	2.8%	1.07 [0.97, 1.19]
Peden et al. 2011	0.079	0.008	7.7%	1.08 [1.07, 1.10]
Qi et al. 2013	0.045	0.029	5.1%	1.05 [0.99, 1.11]
Reilly et al. 2011	0.025	0.024	5.7%	1.03 [0.98, 1.07]
Samani et al. 2007	0.13	0.016	6.8%	1.14 [1.10, 1.18]
Schunkert et al. 2011	0.111	0.011	7.4%	1.12 [1.09, 1.14]
van der Harst et al. 2018	0.078	0.002	8.0%	1.08 [1.08, 1.09]
Wild et al. 2011	0.114	0.008	7.7%	1.12 [1.10, 1.14]
Yamada et al. 2018 (b)	0.155	0.037	4.1%	1.17 [1.09, 1.26]
**Total (95% CI)**			**100.0%**	**1.07 [1.05, 1.09]**

Heterogeneity: Tau² = 0.00; Chi² = 889.52, df = 14 (P < 0.00001); I² = 98%
Test for overall effect: Z = 6.38 (P < 0.00001)

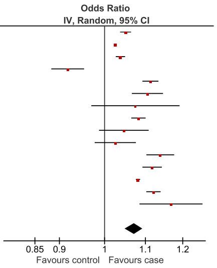

rs501120

Study or Subgroup	log[Odds Ratio]	SE	Weight	Odds Ratio IV, Random, 95% CI
Charmet et al. 2018	0.072	0.054	3.2%	1.07 [0.97, 1.19]
Dichgans et al. 2014	0.036	0.008	21.6%	1.04 [1.02, 1.05]
Fall et al. 2018	0.009	0.002	24.7%	1.01 [1.01, 1.01]
Han et al. 2017	0.022	0.016	15.5%	1.02 [0.99, 1.05]
Samani et al. 2007	0.124	0.023	11.1%	1.13 [1.08, 1.18]
van der Harst et al. 2018	0.034	0.004	24.0%	1.03 [1.03, 1.04]
**Total (95% CI)**			**100.0%**	**1.04 [1.02, 1.06]**

Heterogeneity: Tau² = 0.00; Chi² = 62.00, df = 5 (P < 0.00001); I² = 92%
Test for overall effect: Z = 3.66 (P = 0.0003)

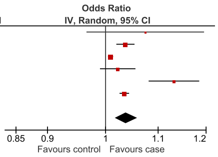

rs507666

Study or Subgroup	log[Odds Ratio]	SE	Weight	Odds Ratio IV, Random, 95% CI
Matsunaga et al. 2020	0.057	0.01	17.2%	1.06 [1.04, 1.08]
Nelson et al. 2017	0.033	0.004	38.0%	1.03 [1.03, 1.04]
van der Harst et al. 2018	0.032	0.003	42.3%	1.03 [1.03, 1.04]
Yamada et al. 2018 (b)	0.083	0.032	2.5%	1.09 [1.02, 1.16]
**Total (95% CI)**			**100.0%**	**1.04 [1.03, 1.05]**

Heterogeneity: Tau² = 0.00; Chi² = 8.14, df = 3 (P = 0.04); I² = 63%
Test for overall effect: Z = 7.38 (P < 0.00001)

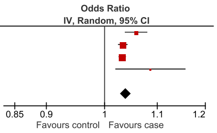

rs514659

Study or Subgroup	log[Odds Ratio]	SE	Weight	Odds Ratio IV, Fixed, 95% CI
Qi et al. 2013	0.049	0.029	21.1%	1.05 [0.99, 1.11]
Reilly et al. 2011	0.083	0.015	78.9%	1.09 [1.06, 1.12]
**Total (95% CI)**			**100.0%**	**1.08 [1.05, 1.11]**

Heterogeneity: Chi² = 1.08, df = 1 (P = 0.30); I² = 8%
Test for overall effect: Z = 5.69 (P < 0.00001)

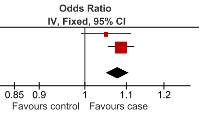

rs515135

Study or Subgroup	log[Odds Ratio]	SE	Weight	Odds Ratio IV, Random, 95% CI
Dichgans et al. 2014	0.027	0.008	22.9%	1.03 [1.01, 1.04]
Fall et al. 2018	0.009	0.004	35.3%	1.01 [1.00, 1.02]
Han et al. 2017	0	0.029	3.4%	1.00 [0.94, 1.06]
van der Harst et al. 2018	0.024	0.003	38.4%	1.02 [1.02, 1.03]
**Total (95% CI)**			**100.0%**	**1.02 [1.01, 1.03]**

Heterogeneity: Tau² = 0.00; Chi² = 10.45, df = 3 (P = 0.02); I² = 71%
Test for overall effect: Z = 3.33 (P = 0.0009)

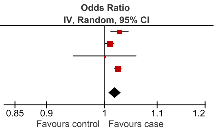

rs518394

Study or Subgroup	log[Odds Ratio]	SE	Weight	Odds Ratio IV, Fixed, 95% CI
Samani et al. 2007	0.093	0.015	22.1%	1.10 [1.07, 1.13]
Wild et al. 2011	0.072	0.008	77.9%	1.07 [1.06, 1.09]
**Total (95% CI)**			**100.0%**	**1.08 [1.06, 1.09]**

Heterogeneity: Chi² = 1.53, df = 1 (P = 0.22); I² = 34%
Test for overall effect: Z = 10.86 (P < 0.00001)

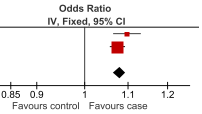

rs523096

Study or Subgroup	log[Odds Ratio]	SE	Weight	Odds Ratio IV, Fixed, 95% CI
Samani et al. 2007	0.086	0.015	17.9%	1.09 [1.06, 1.12]
Wild et al. 2011	0.079	0.007	82.1%	1.08 [1.07, 1.10]
**Total (95% CI)**			**100.0%**	**1.08 [1.07, 1.10]**

Heterogeneity: Chi² = 0.18, df = 1 (P = 0.67); I² = 0%
Test for overall effect: Z = 12.65 (P < 0.00001)

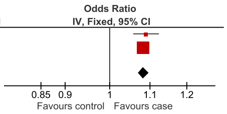

rs55730499

Study or Subgroup	log[Odds Ratio]	SE	Weight	Odds Ratio IV, Random, 95% CI
Charmet et al. 2018	0.086	0.061	4.8%	1.09 [0.97, 1.23]
Nikpay et al. 2015	0.137	0.01	32.3%	1.15 [1.12, 1.17]
van der Harst et al. 2018	0.146	0.006	35.9%	1.16 [1.14, 1.17]
Wei et al. 2018	0.086	0.015	27.0%	1.09 [1.06, 1.12]
**Total (95% CI)**			**100.0%**	**1.13 [1.10, 1.16]**

Heterogeneity: Tau² = 0.00; Chi² = 14.51, df = 3 (P = 0.002); I² = 79%
Test for overall effect: Z = 8.66 (P < 0.00001)

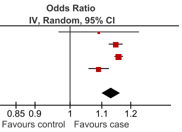

rs55940034

Study or Subgroup	log[Odds Ratio]	SE	Weight	Odds Ratio IV, Fixed, 95% CI
Han et al. 2017	0.021	0.024	2.7%	1.02 [0.97, 1.07]
Matsunaga et al. 2020	0.025	0.004	97.3%	1.03 [1.02, 1.03]
**Total (95% CI)**			**100.0%**	**1.03 [1.02, 1.03]**

Heterogeneity: Chi² = 0.03, df = 1 (P = 0.87); I² = 0%
Test for overall effect: Z = 6.31 (P < 0.00001)

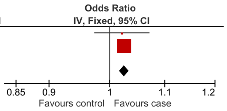

rs56062135

Study or Subgroup	log[Odds Ratio]	SE	Weight	Odds Ratio IV, Random, 95% CI
Charmet et al. 2018	0.029	0.042	2.5%	1.03 [0.95, 1.12]
Fall et al. 2018	0.009	0.001	25.8%	1.01 [1.01, 1.01]
Matsunaga et al. 2020	0.033	0.004	23.9%	1.03 [1.03, 1.04]
Nikpay et al. 2015	0.029	0.005	22.9%	1.03 [1.02, 1.04]
van der Harst et al. 2018	0.028	0.003	24.8%	1.03 [1.02, 1.03]
**Total (95% CI)**			**100.0%**	**1.02 [1.01, 1.04]**

Heterogeneity: Tau² = 0.00; Chi² = 76.23, df = 4 (P < 0.00001); I² = 95%
Test for overall effect: Z = 3.48 (P = 0.0005)

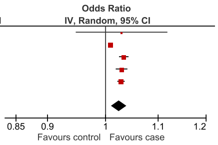

rs56131196

Study or Subgroup	log[Odds Ratio]	SE	Weight	Odds Ratio IV, Fixed, 95% CI
Matsunaga et al. 2020	0.037	0.004	61.0%	1.04 [1.03, 1.05]
Nelson et al. 2017	0.029	0.005	39.0%	1.03 [1.02, 1.04]
**Total (95% CI)**			**100.0%**	**1.03 [1.03, 1.04]**

Heterogeneity: Chi² = 1.56, df = 1 (P = 0.21); I² = 36%
Test for overall effect: Z = 10.85 (P < 0.00001)

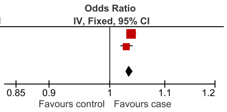

rs56210063

Study or Subgroup	log[Odds Ratio]	SE	Weight	Odds Ratio IV, Fixed, 95% CI
Nelson et al. 2017	0.029	0.007	42.4%	1.03 [1.02, 1.04]
van der Harst et al. 2018	0.025	0.006	57.6%	1.03 [1.01, 1.04]
**Total (95% CI)**			**100.0%**	**1.03 [1.02, 1.04]**

Heterogeneity: Chi² = 0.19, df = 1 (P = 0.66); I² = 0%
Test for overall effect: Z = 5.86 (P < 0.00001)

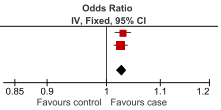

rs56289821

Study or Subgroup	log[Odds Ratio]	SE	Weight	Odds Ratio IV, Fixed, 95% CI
Charmet et al. 2018	0.041	0.057	0.5%	1.04 [0.93, 1.16]
Nikpay et al. 2015	0.057	0.007	33.6%	1.06 [1.04, 1.07]
van der Harst et al. 2018	0.059	0.005	65.9%	1.06 [1.05, 1.07]
**Total (95% CI)**			**100.0%**	**1.06 [1.05, 1.07]**

Heterogeneity: Chi² = 0.15, df = 2 (P = 0.93); I² = 0%
Test for overall effect: Z = 14.35 (P < 0.00001)

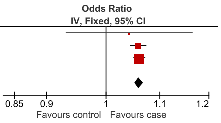

rs56313611

Study or Subgroup	log[Odds Ratio]	SE	Weight	Odds Ratio IV, Fixed, 95% CI
Nelson et al. 2017	-0.027	0.006	30.8%	0.97 [0.96, 0.98]
van der Harst et al. 2018	-0.023	0.004	69.2%	0.98 [0.97, 0.98]
**Total (95% CI)**			**100.0%**	**0.98 [0.97, 0.98]**

Heterogeneity: Chi² = 0.31, df = 1 (P = 0.58); I² = 0%
Test for overall effect: Z = 7.28 (P < 0.00001)

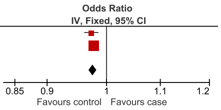

rs56336142

Study or Subgroup	log[Odds Ratio]	SE	Weight	Odds Ratio IV, Fixed, 95% CI
Charmet et al. 2018	0.017	0.043	1.3%	1.02 [0.93, 1.11]
Han et al. 2017	0.027	0.021	5.3%	1.03 [0.99, 1.07]
Nikpay et al. 2015	0.029	0.005	93.4%	1.03 [1.02, 1.04]
**Total (95% CI)**			**100.0%**	**1.03 [1.02, 1.04]**

Heterogeneity: Chi² = 0.08, df = 2 (P = 0.96); I² = 0%
Test for overall effect: Z = 5.95 (P < 0.00001)

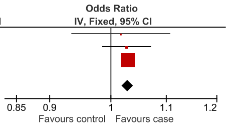

rs56393506

Study or Subgroup	log[Odds Ratio]	SE	Weight	Odds Ratio IV, Fixed, 95% CI
Matsunaga et al. 2020	0.061	0.005	80.0%	1.06 [1.05, 1.07]
Wei et al. 2018	0.049	0.01	20.0%	1.05 [1.03, 1.07]
**Total (95% CI)**			**100.0%**	**1.06 [1.05, 1.07]**

Heterogeneity: Chi² = 1.15, df = 1 (P = 0.28); I² = 13%
Test for overall effect: Z = 13.10 (P < 0.00001)

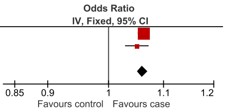

rs564398

Study or Subgroup	log[Odds Ratio]	SE	Weight	Odds Ratio IV, Fixed, 95% CI
Samani et al. 2007	0.1	0.016	20.0%	1.11 [1.07, 1.14]
Wild et al. 2011	0.083	0.008	80.0%	1.09 [1.07, 1.10]
**Total (95% CI)**			**100.0%**	**1.09 [1.08, 1.11]**

Heterogeneity: Chi² = 0.90, df = 1 (P = 0.34); I² = 0%
Test for overall effect: Z = 12.07 (P < 0.00001)

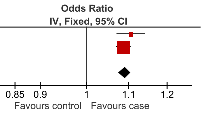

rs571353

Study or Subgroup	log[Odds Ratio]	SE	Weight	Odds Ratio IV, Fixed, 95% CI
Nelson et al. 2017	-0.018	0.005	26.5%	0.98 [0.97, 0.99]
van der Harst et al. 2018	-0.019	0.003	73.5%	0.98 [0.98, 0.99]
**Total (95% CI)**			**100.0%**	**0.98 [0.98, 0.99]**

Heterogeneity: Chi² = 0.03, df = 1 (P = 0.86); I² = 0%
Test for overall effect: Z = 7.28 (P < 0.00001)

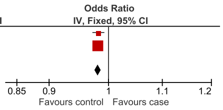

rs579459

Study or Subgroup	log[Odds Ratio]	SE	Weight	Odds Ratio IV, Random, 95% CI
Fall et al. 2018	0.004	0.002	34.4%	1.00 [1.00, 1.01]
Lee et al. 2013	0.033	0.019	21.4%	1.03 [1.00, 1.07]
Qi et al. 2013	0.017	0.034	11.6%	1.02 [0.95, 1.09]
Schunkert et al. 2011	0.041	0.006	32.6%	1.04 [1.03, 1.05]
**Total (95% CI)**			**100.0%**	**1.02 [1.00, 1.05]**

Heterogeneity: Tau² = 0.00; Chi² = 36.05, df = 3 (P < 0.00001); I² = 92%
Test for overall effect: Z = 1.67 (P = 0.09)

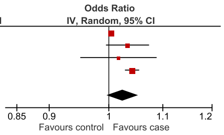

rs590121

Study or Subgroup	log[Odds Ratio]	SE	Weight	Odds Ratio IV, Random, 95% CI
Charmet et al. 2018	-0.097	0.04	43.9%	0.91 [0.84, 0.98]
van der Harst et al. 2018	0.017	0.003	56.1%	1.02 [1.01, 1.02]
**Total (95% CI)**			**100.0%**	**0.97 [0.87, 1.08]**

Heterogeneity: Tau² = 0.01; Chi² = 8.08, df = 1 (P = 0.004); I² = 88%
Test for overall effect: Z = 0.58 (P = 0.56)

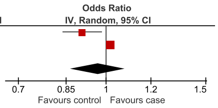

rs59898454

Study or Subgroup	log[Odds Ratio]	SE	Weight	Odds Ratio IV, Fixed, 95% CI
Nelson et al. 2017	0.066	0.014	33.8%	1.07 [1.04, 1.10]
van der Harst et al. 2018	0.04	0.01	66.2%	1.04 [1.02, 1.06]
**Total (95% CI)**			**100.0%**	**1.05 [1.03, 1.07]**

Heterogeneity: Chi² = 2.28, df = 1 (P = 0.13); I² = 56%
Test for overall effect: Z = 6.00 (P < 0.00001)

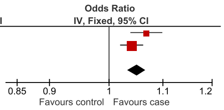

rs599839

Study or Subgroup	log[Odds Ratio]	SE	Weight	Odds Ratio IV, Random, 95% CI
Lee et al. 2013	0.127	0.04	12.6%	1.14 [1.05, 1.23]
O'Donnell et al. 2011	0.039	0.013	26.0%	1.04 [1.01, 1.07]
Samani et al. 2007	0.111	0.019	22.8%	1.12 [1.08, 1.16]
Schunkert et al. 2011	0.045	0.007	28.6%	1.05 [1.03, 1.06]
Yamada et al. 2018 (b)	0.174	0.048	10.1%	1.19 [1.08, 1.31]
**Total (95% CI)**			**100.0%**	**1.09 [1.05, 1.13]**

Heterogeneity: Tau² = 0.00; Chi² = 21.56, df = 4 (P = 0.0002); I² = 81%
Test for overall effect: Z = 4.37 (P < 0.0001)

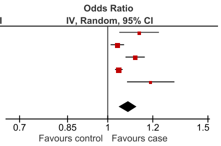

rs602633

Study or Subgroup	log[Odds Ratio]	SE	Weight	Odds Ratio IV, Random, 95% CI
Dichgans et al. 2014	-0.046	0.007	29.7%	0.96 [0.94, 0.97]
van der Harst et al. 2018	-0.049	0.003	32.8%	0.95 [0.95, 0.96]
Vujkovic et al. 2020	-0.022	0.002	33.2%	0.98 [0.97, 0.98]
Yamada et al. 2018 (b)	-0.194	0.05	4.3%	0.82 [0.75, 0.91]
**Total (95% CI)**			**100.0%**	**0.96 [0.93, 0.98]**

Heterogeneity: Tau² = 0.00; Chi² = 71.43, df = 3 (P < 0.00001); I² = 96%
Test for overall effect: Z = 4.07 (P < 0.0001)

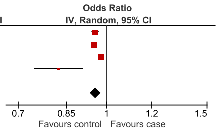

rs6129767

Study or Subgroup	log[Odds Ratio]	SE	Weight	Odds Ratio IV, Fixed, 95% CI
Nelson et al. 2017	0.017	0.004	36.0%	1.02 [1.01, 1.03]
van der Harst et al. 2018	0.017	0.003	64.0%	1.02 [1.01, 1.02]
**Total (95% CI)**			**100.0%**	**1.02 [1.01, 1.02]**

Heterogeneity: Chi² = 0.00, df = 1 (P = 1.00); I² = 0%
Test for overall effect: Z = 7.08 (P < 0.00001)

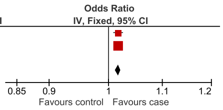

rs61734696

Study or Subgroup	log[Odds Ratio]	SE	Weight	Odds Ratio IV, Fixed, 95% CI
Yamada et al. 2018 (a)	-2	0.255	50.0%	0.14 [0.08, 0.22]
Yamada et al. 2018 (b)	-1.699	0.255	50.0%	0.18 [0.11, 0.30]
**Total (95% CI)**			**100.0%**	**0.16 [0.11, 0.22]**

Heterogeneity: Chi² = 0.70, df = 1 (P = 0.40); I² = 0%
Test for overall effect: Z = 10.26 (P < 0.00001)

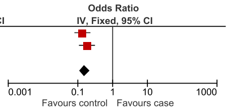

rs62076439

Study or Subgroup	log[Odds Ratio]	SE	Weight	Odds Ratio IV, Fixed, 95% CI
Nelson et al. 2017	0.021	0.003	50.0%	1.02 [1.02, 1.03]
van der Harst et al. 2018	0.021	0.003	50.0%	1.02 [1.02, 1.03]
**Total (95% CI)**			**100.0%**	**1.02 [1.02, 1.03]**

Heterogeneity: Chi² = 0.00, df = 1 (P = 1.00); I² = 0%
Test for overall effect: Z = 9.90 (P < 0.00001)

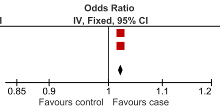

rs62253653

Study or Subgroup	log[Odds Ratio]	SE	Weight	Odds Ratio IV, Fixed, 95% CI
Nelson et al. 2017	0.018	0.003	50.0%	1.02 [1.01, 1.02]
van der Harst et al. 2018	0.012	0.003	50.0%	1.01 [1.01, 1.02]
**Total (95% CI)**			**100.0%**	**1.02 [1.01, 1.02]**

Heterogeneity: Chi² = 2.00, df = 1 (P = 0.16); I² = 50%
Test for overall effect: Z = 7.07 (P < 0.00001)

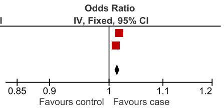

rs629301

Study or Subgroup	log[Odds Ratio]	SE	Weight	Odds Ratio IV, Random, 95% CI
Lettre et al. 2011	0.037	0.032	53.3%	1.04 [0.97, 1.10]
Yamada et al. 2018 (b)	0.187	0.05	46.7%	1.21 [1.09, 1.33]
**Total (95% CI)**			**100.0%**	**1.11 [0.96, 1.29]**

Heterogeneity: Tau² = 0.01; Chi² = 6.38, df = 1 (P = 0.01); I² = 84%
Test for overall effect: Z = 1.43 (P = 0.15)

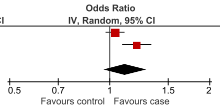

rs634552

Study or Subgroup	log[Odds Ratio]	SE	Weight	Odds Ratio IV, Fixed, 95% CI
Nelson et al. 2017	0.021	0.005	39.0%	1.02 [1.01, 1.03]
van der Harst et al. 2018	0.025	0.004	61.0%	1.03 [1.02, 1.03]
**Total (95% CI)**			**100.0%**	**1.02 [1.02, 1.03]**

Heterogeneity: Chi² = 0.39, df = 1 (P = 0.53); I² = 0%
Test for overall effect: Z = 7.50 (P < 0.00001)

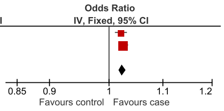

rs643319

Study or Subgroup	log[Odds Ratio]	SE	Weight	Odds Ratio IV, Random, 95% CI
Samani et al. 2007	0.097	0.016	38.6%	1.10 [1.07, 1.14]
Wild et al. 2011	0.068	0.008	61.4%	1.07 [1.05, 1.09]
**Total (95% CI)**			**100.0%**	**1.08 [1.05, 1.11]**

Heterogeneity: Tau² = 0.00; Chi² = 2.63, df = 1 (P = 0.10); I² = 62%
Test for overall effect: Z = 5.61 (P < 0.00001)

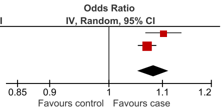

rs643434

Study or Subgroup	log[Odds Ratio]	SE	Weight	Odds Ratio IV, Random, 95% CI
Matsunaga et al. 2020	0.021	0.003	52.8%	1.02 [1.02, 1.03]
Reilly et al. 2011	0.079	0.014	47.2%	1.08 [1.05, 1.11]
**Total (95% CI)**			**100.0%**	**1.05 [0.99, 1.11]**

Heterogeneity: Tau² = 0.00; Chi² = 16.41, df = 1 (P < 0.0001); I² = 94%
Test for overall effect: Z = 1.67 (P = 0.09)

Odds Ratio IV, Random, 95% CI: 0.7 0.85 1 1.2 1.5 — Favours control / Favours case

rs646776

Study or Subgroup	log[Odds Ratio]	SE	Weight	Odds Ratio IV, Random, 95% CI
Fall et al. 2018	0.025	0.005	16.5%	1.03 [1.02, 1.04]
Han et al. 2017	0.049	0.027	9.6%	1.05 [1.00, 1.11]
Hirokawa et al. 2015	-0.108	0.037	7.0%	0.90 [0.83, 0.97]
Kathiresan et al. 2009	0.068	0.012	14.7%	1.07 [1.05, 1.10]
Klarin et al. 2017	0.068	0.011	15.0%	1.07 [1.05, 1.09]
Peden et al. 2011	0.057	0.01	15.3%	1.06 [1.04, 1.08]
Qi et al. 2013	0.083	0.036	7.2%	1.09 [1.01, 1.17]
Reilly et al. 2011	0.029	0.026	9.9%	1.03 [0.98, 1.08]
Yamada et al. 2018 (b)	0.187	0.05	4.7%	1.21 [1.09, 1.33]
**Total (95% CI)**			**100.0%**	**1.05 [1.02, 1.08]**

Heterogeneity: Tau² = 0.00; Chi² = 50.17, df = 8 (P < 0.00001); I² = 84%
Test for overall effect: Z = 3.76 (P = 0.0002)

Odds Ratio IV, Random, 95% CI: 0.7 0.85 1 1.2 1.5 — Favours control / Favours case

rs6475606

Study or Subgroup	log[Odds Ratio]	SE	Weight	Odds Ratio IV, Random, 95% CI
Aoki et al. 2011	0.042	0.025	20.1%	1.04 [0.99, 1.10]
Erdmann et al. 2011	0.045	0.006	25.5%	1.05 [1.03, 1.06]
Lettre et al. 2011	0.299	0.088	5.6%	1.35 [1.13, 1.60]
Samani et al. 2007	0.127	0.015	23.5%	1.14 [1.10, 1.17]
Wild et al. 2011	0.111	0.008	25.2%	1.12 [1.10, 1.14]
**Total (95% CI)**			**100.0%**	**1.10 [1.05, 1.15]**

Heterogeneity: Tau² = 0.00; Chi² = 65.41, df = 4 (P < 0.00001); I² = 94%
Test for overall effect: Z = 4.01 (P < 0.0001)

Odds Ratio IV, Random, 95% CI: 0.7 0.85 1 1.2 1.5 — Favours control / Favours case

rs6494488

Study or Subgroup	log[Odds Ratio]	SE	Weight	Odds Ratio IV, Fixed, 95% CI
Charmet et al. 2018	-0.004	0.052	0.3%	1.00 [0.90, 1.10]
van der Harst et al. 2018	0.019	0.003	99.7%	1.02 [1.01, 1.03]
**Total (95% CI)**			**100.0%**	**1.02 [1.01, 1.03]**

Heterogeneity: Chi² = 0.19, df = 1 (P = 0.66); I² = 0%
Test for overall effect: Z = 6.32 (P < 0.00001)

Odds Ratio IV, Fixed, 95% CI: 0.85 0.9 1 1.1 1.2 — Favours control / Favours case

rs6504218

Study or Subgroup	log[Odds Ratio]	SE	Weight	Odds Ratio IV, Fixed, 95% CI
Nelson et al. 2017	0.017	0.004	36.0%	1.02 [1.01, 1.03]
van der Harst et al. 2018	0.017	0.003	64.0%	1.02 [1.01, 1.02]
**Total (95% CI)**			**100.0%**	**1.02 [1.01, 1.02]**

Heterogeneity: Chi² = 0.00, df = 1 (P = 1.00); I² = 0%
Test for overall effect: Z = 7.08 (P < 0.00001)

Odds Ratio IV, Fixed, 95% CI: 0.85 0.9 1 1.1 1.2 — Favours control / Favours case

rs6511720

Study or Subgroup	log[Odds Ratio]	SE	Weight	Odds Ratio IV, Fixed, 95% CI
Lettre et al. 2011	0.004	0.051	0.5%	1.00 [0.91, 1.11]
Nelson et al. 2017	-0.056	0.005	49.8%	0.95 [0.94, 0.95]
van der Harst et al. 2018	-0.057	0.005	49.8%	0.94 [0.94, 0.95]
**Total (95% CI)**			**100.0%**	**0.95 [0.94, 0.95]**

Heterogeneity: Chi² = 1.42, df = 2 (P = 0.49); I² = 0%
Test for overall effect: Z = 15.94 (P < 0.00001)

Odds Ratio IV, Fixed, 95% CI: 0.85 0.9 1 1.1 1.2 — Favours control / Favours case

rs6538176

Study or Subgroup	log[Odds Ratio]	SE	Weight	Odds Ratio IV, Random, 95% CI
Nelson et al. 2017	0.021	0.004	45.7%	1.02 [1.01, 1.03]
van der Harst et al. 2018	0.012	0.003	54.3%	1.01 [1.01, 1.02]
**Total (95% CI)**			**100.0%**	**1.02 [1.01, 1.03]**

Heterogeneity: Tau² = 0.00; Chi² = 3.24, df = 1 (P = 0.07); I² = 69%
Test for overall effect: Z = 3.59 (P = 0.0003)

Odds Ratio IV, Random, 95% CI: 0.85 0.9 1 1.1 1.2 — Favours control / Favours case

rs6544713

Study or Subgroup	log[Odds Ratio]	SE	Weight	Odds Ratio IV, Random, 95% CI
Dichgans et al. 2014	0.025	0.007	22.9%	1.03 [1.01, 1.04]
Fall et al. 2018	0.009	0.002	38.6%	1.01 [1.01, 1.01]
Hirokawa et al. 2015	-0.284	0.623	0.0%	0.75 [0.22, 2.55]
van der Harst et al. 2018	0.021	0.002	38.6%	1.02 [1.02, 1.03]
**Total (95% CI)**			**100.0%**	**1.02 [1.01, 1.03]**

Heterogeneity: Tau² = 0.00; Chi² = 20.19, df = 3 (P = 0.0002); I² = 85%
Test for overall effect: Z = 3.43 (P = 0.0006)

Odds Ratio IV, Random, 95% CI: 0.2 0.5 1 2 5 — Favours control / Favours case

rs657152

Study or Subgroup	log[Odds Ratio]	SE	Weight	Odds Ratio IV, Fixed, 95% CI
Hirokawa et al. 2015	-0.06	0.019	35.2%	0.94 [0.91, 0.98]
Reilly et al. 2011	-0.076	0.014	64.8%	0.93 [0.90, 0.95]
**Total (95% CI)**			**100.0%**	**0.93 [0.91, 0.95]**

Heterogeneity: Chi² = 0.46, df = 1 (P = 0.50); I² = 0%
Test for overall effect: Z = 6.24 (P < 0.00001)

Odds Ratio IV, Fixed, 95% CI: 0.85 0.9 1 1.1 1.2 — Favours control / Favours case

rs663129

Study or Subgroup	log[Odds Ratio]	SE	Weight	Odds Ratio IV, Random, 95% CI
Charmet et al. 2018	0.004	0.041	4.7%	1.00 [0.93, 1.09]
Fall et al. 2018	0.004	0.002	41.4%	1.00 [1.00, 1.01]
Han et al. 2017	0.045	0.02	14.7%	1.05 [1.01, 1.09]
Nikpay et al. 2015	0.025	0.004	39.2%	1.03 [1.02, 1.03]
**Total (95% CI)**			**100.0%**	**1.02 [1.00, 1.04]**

Heterogeneity: Tau² = 0.00; Chi² = 25.42, df = 3 (P < 0.0001); I² = 88%
Test for overall effect: Z = 1.92 (P = 0.05)

Odds Ratio IV, Random, 95% CI: 0.85 0.9 1 1.1 1.2 — Favours control / Favours case

rs6689306

Study or Subgroup	log[Odds Ratio]	SE	Weight	Odds Ratio IV, Fixed, 95% CI
Nelson et al. 2017	0.021	0.004	50.0%	1.02 [1.01, 1.03]
Nikpay et al. 2015	0.025	0.004	50.0%	1.03 [1.02, 1.03]
**Total (95% CI)**			**100.0%**	**1.02 [1.02, 1.03]**

Heterogeneity: Chi² = 0.50, df = 1 (P = 0.48); I² = 0%
Test for overall effect: Z = 8.13 (P < 0.00001)

Odds Ratio IV, Fixed, 95% CI: 0.85 0.9 1 1.1 1.2 — Favours control / Favours case

rs6700559

Study or Subgroup	log[Odds Ratio]	SE	Weight	Odds Ratio IV, Random, 95% CI
Charmet et al. 2018	0.061	0.034	39.2%	1.06 [0.99, 1.14]
van der Harst et al. 2018	-0.012	0.002	60.8%	0.99 [0.98, 0.99]
**Total (95% CI)**			**100.0%**	**1.02 [0.95, 1.09]**

Heterogeneity: Tau² = 0.00; Chi² = 4.59, df = 1 (P = 0.03); I² = 78%
Test for overall effect: Z = 0.47 (P = 0.64)

Odds Ratio IV, Random, 95% CI: 0.7 0.85 1 1.2 1.5 — Favours control / Favours case

rs671

Study or Subgroup	log[Odds Ratio]	SE	Weight	Odds Ratio IV, Random, 95% CI
Takeuchi et al. 2012	0.155	0.012	36.7%	1.17 [1.14, 1.20]
Yamada et al. 2018 (a)	0.433	0.059	30.9%	1.54 [1.37, 1.73]
Yamada et al. 2018 (b)	0.255	0.05	32.4%	1.29 [1.17, 1.42]
**Total (95% CI)**			**100.0%**	**1.31 [1.12, 1.54]**

Heterogeneity: Tau² = 0.02; Chi² = 24.32, df = 2 (P < 0.00001); I² = 92%
Test for overall effect: Z = 3.40 (P = 0.0007)

Odds Ratio IV, Random, 95% CI: 0.5 0.7 1 1.5 2 — Favours control / Favours case

rs67180937

Study or Subgroup	log[Odds Ratio]	SE	Weight	Odds Ratio IV, Random, 95% CI
Matsunaga et al. 2020	0.053	0.008	13.8%	1.05 [1.04, 1.07]
Nelson et al. 2017	0.029	0.004	28.6%	1.03 [1.02, 1.04]
Nikpay et al. 2015	0.033	0.005	23.8%	1.03 [1.02, 1.04]
van der Harst et al. 2018	0.035	0.003	33.8%	1.04 [1.03, 1.04]
**Total (95% CI)**			**100.0%**	**1.04 [1.03, 1.04]**

Heterogeneity: Tau² = 0.00; Chi² = 7.34, df = 3 (P = 0.06); I² = 59%
Test for overall effect: Z = 9.86 (P < 0.00001)

Odds Ratio IV, Random, 95% CI: 0.85 0.9 1 1.1 1.2 — Favours control / Favours case

rs6725887

Study or Subgroup	log[Odds Ratio]	SE	Weight	Odds Ratio IV, Random, 95% CI
Dichgans et al. 2014	0.057	0.009	15.6%	1.06 [1.04, 1.08]
Fall et al. 2018	0.013	0.002	17.0%	1.01 [1.01, 1.02]
Han et al. 2017	0.072	0.057	3.6%	1.07 [0.96, 1.20]
Hirokawa et al. 2015	0.004	0.12	1.0%	1.00 [0.79, 1.27]
Kathiresan et al. 2009	0.068	0.011	15.0%	1.07 [1.05, 1.09]
Lee et al. 2013	0.14	0.072	2.5%	1.15 [1.00, 1.32]
Peden et al. 2011	0.045	0.014	13.9%	1.05 [1.02, 1.08]
Qi et al. 2013	-0.051	0.041	5.8%	0.95 [0.88, 1.03]
Reilly et al. 2011	0.021	0.024	10.3%	1.02 [0.97, 1.07]
Schunkert et al. 2011	0.057	0.01	15.3%	1.06 [1.04, 1.08]
**Total (95% CI)**			**100.0%**	**1.04 [1.02, 1.07]**

Heterogeneity: Tau² = 0.00; Chi² = 71.14, df = 9 (P < 0.00001); I² = 87%
Test for overall effect: Z = 3.40 (P = 0.0007)

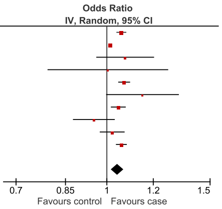

rs68170813

Study or Subgroup	log[Odds Ratio]	SE	Weight	Odds Ratio IV, Fixed, 95% CI
Nikpay et al. 2015	0.021	0.005	26.5%	1.02 [1.01, 1.03]
van der Harst et al. 2018	0.015	0.003	73.5%	1.02 [1.01, 1.02]
**Total (95% CI)**			**100.0%**	**1.02 [1.01, 1.02]**

Heterogeneity: Chi² = 1.06, df = 1 (P = 0.30); I² = 6%
Test for overall effect: Z = 6.45 (P < 0.00001)

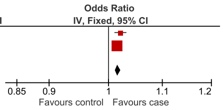

rs6841581

Study or Subgroup	log[Odds Ratio]	SE	Weight	Odds Ratio IV, Fixed, 95% CI
Nelson et al. 2017	0.033	0.005	26.5%	1.03 [1.02, 1.04]
van der Harst et al. 2018	0.033	0.003	73.5%	1.03 [1.03, 1.04]
**Total (95% CI)**			**100.0%**	**1.03 [1.03, 1.04]**

Heterogeneity: Chi² = 0.00, df = 1 (P = 1.00); I² = 0%
Test for overall effect: Z = 12.83 (P < 0.00001)

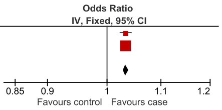

rs6842241

Study or Subgroup	log[Odds Ratio]	SE	Weight	Odds Ratio IV, Random, 95% CI
Hirokawa et al. 2015	-0.018	0.02	43.2%	0.98 [0.94, 1.02]
van der Harst et al. 2018	0.035	0.004	56.8%	1.04 [1.03, 1.04]
**Total (95% CI)**			**100.0%**	**1.01 [0.96, 1.07]**

Heterogeneity: Tau² = 0.00; Chi² = 6.75, df = 1 (P = 0.009); I² = 85%
Test for overall effect: Z = 0.46 (P = 0.64)

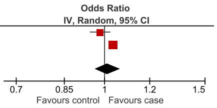

rs688034

Study or Subgroup	log[Odds Ratio]	SE	Weight	Odds Ratio IV, Random, 95% CI
Burton et al. 2007	0.21	0.042	24.2%	1.23 [1.14, 1.34]
Samani et al. 2007	0.025	0.014	36.9%	1.03 [1.00, 1.05]
Schunkert et al. 2011	-0.004	0.007	38.8%	1.00 [0.98, 1.01]
**Total (95% CI)**			**100.0%**	**1.06 [0.99, 1.13]**

Heterogeneity: Tau² = 0.00; Chi² = 27.47, df = 2 (P < 0.00001); I² = 93%
Test for overall effect: Z = 1.76 (P = 0.08)

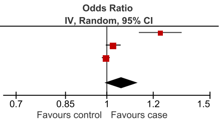

rs6903956

Study or Subgroup	log[Odds Ratio]	SE	Weight	Odds Ratio IV, Random, 95% CI
Fall et al. 2018	0	0.002	27.1%	1.00 [1.00, 1.00]
Han et al. 2017	0.086	0.03	24.2%	1.09 [1.03, 1.16]
Hirokawa et al. 2015	-0.013	0.033	23.7%	0.99 [0.93, 1.05]
Wang et al. 2011	0.179	0.026	24.9%	1.20 [1.14, 1.26]
**Total (95% CI)**			**100.0%**	**1.06 [0.97, 1.16]**

Heterogeneity: Tau² = 0.01; Chi² = 55.29, df = 3 (P < 0.00001); I² = 95%
Test for overall effect: Z = 1.38 (P = 0.17)

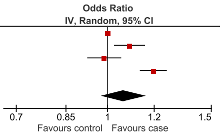

rs6905288

Study or Subgroup	log[Odds Ratio]	SE	Weight	Odds Ratio IV, Fixed, 95% CI
Nelson et al. 2017	0.017	0.004	36.0%	1.02 [1.01, 1.03]
van der Harst et al. 2018	0.021	0.003	64.0%	1.02 [1.02, 1.03]
**Total (95% CI)**			**100.0%**	**1.02 [1.01, 1.02]**

Heterogeneity: Chi² = 0.64, df = 1 (P = 0.42); I² = 0%
Test for overall effect: Z = 8.15 (P < 0.00001)

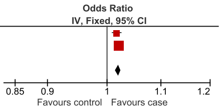

rs6909752

Study or Subgroup	log[Odds Ratio]	SE	Weight	Odds Ratio IV, Fixed, 95% CI
Charmet et al. 2018	0.004	0.037	0.4%	1.00 [0.93, 1.08]
Nelson et al. 2017	0.022	0.004	35.8%	1.02 [1.01, 1.03]
van der Harst et al. 2018	0.019	0.003	63.7%	1.02 [1.01, 1.03]
**Total (95% CI)**			**100.0%**	**1.02 [1.02, 1.03]**

Heterogeneity: Chi² = 0.55, df = 2 (P = 0.76); I² = 0%
Test for overall effect: Z = 8.36 (P < 0.00001)

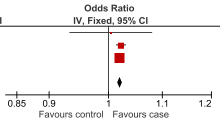

rs6922269

Study or Subgroup	log[Odds Ratio]	SE	Weight	Odds Ratio IV, Random, 95% CI
Burton et al. 2007	0.217	0.049	12.2%	1.24 [1.13, 1.37]
Kathiresan et al. 2009	0.017	0.011	29.6%	1.02 [1.00, 1.04]
Samani et al. 2007	0.09	0.016	27.3%	1.09 [1.06, 1.13]
Schunkert et al. 2011	0.027	0.007	30.9%	1.03 [1.01, 1.04]
**Total (95% CI)**			**100.0%**	**1.07 [1.02, 1.11]**

Heterogeneity: Tau² = 0.00; Chi² = 29.58, df = 3 (P < 0.00001); I² = 90%
Test for overall effect: Z = 2.95 (P = 0.003)

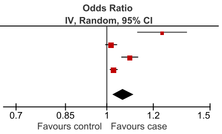

rs7044859

Study or Subgroup	log[Odds Ratio]	SE	Weight	Odds Ratio IV, Fixed, 95% CI
Samani et al. 2007	0.1	0.015	59.0%	1.11 [1.07, 1.14]
Wild et al. 2011	0.068	0.018	41.0%	1.07 [1.03, 1.11]
**Total (95% CI)**			**100.0%**	**1.09 [1.07, 1.12]**

Heterogeneity: Chi² = 1.87, df = 1 (P = 0.17); I² = 46%
Test for overall effect: Z = 7.54 (P < 0.00001)

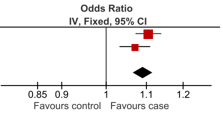

rs7136259

Study or Subgroup	log[Odds Ratio]	SE	Weight	Odds Ratio IV, Random, 95% CI
Fall et al. 2018	0	0.001	51.2%	1.00 [1.00, 1.00]
Lu et al. 2012	0.045	0.007	48.8%	1.05 [1.03, 1.06]
**Total (95% CI)**			**100.0%**	**1.02 [0.98, 1.07]**

Heterogeneity: Tau² = 0.00; Chi² = 40.50, df = 1 (P < 0.00001); I² = 98%
Test for overall effect: Z = 0.98 (P = 0.33)

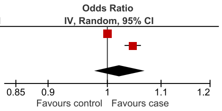

rs7173743

Study or Subgroup	log[Odds Ratio]	SE	Weight	Odds Ratio IV, Random, 95% CI
Charmet et al. 2018	0	0.035	2.8%	1.00 [0.93, 1.07]
Dichgans et al. 2014	0.032	0.006	24.3%	1.03 [1.02, 1.04]
Fall et al. 2018	0.013	0.001	31.3%	1.01 [1.01, 1.02]
Han et al. 2017	0.056	0.015	11.0%	1.06 [1.03, 1.09]
van der Harst et al. 2018	0.028	0.002	30.5%	1.03 [1.02, 1.03]
**Total (95% CI)**			**100.0%**	**1.03 [1.01, 1.04]**

Heterogeneity: Tau² = 0.00; Chi² = 59.14, df = 4 (P < 0.00001); I² = 93%
Test for overall effect: Z = 4.30 (P < 0.0001)

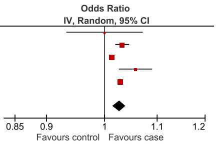

rs7212798

Study or Subgroup	log[Odds Ratio]	SE	Weight	Odds Ratio IV, Fixed, 95% CI
Charmet et al. 2018	-0.009	0.049	1.4%	0.99 [0.90, 1.09]
Han et al. 2017	0.037	0.024	5.8%	1.04 [0.99, 1.09]
Nikpay et al. 2015	0.033	0.006	92.8%	1.03 [1.02, 1.05]
**Total (95% CI)**			**100.0%**	**1.03 [1.02, 1.04]**

Heterogeneity: Chi² = 0.76, df = 2 (P = 0.68); I² = 0%
Test for overall effect: Z = 5.65 (P < 0.00001)

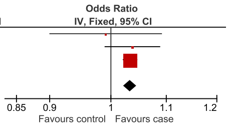

rs7214245

Study or Subgroup	log[Odds Ratio]	SE	Weight	Odds Ratio IV, Fixed, 95% CI
Han et al. 2017	0.009	0.026	2.3%	1.01 [0.96, 1.06]
Nikpay et al. 2015	0.017	0.004	97.7%	1.02 [1.01, 1.03]
**Total (95% CI)**			**100.0%**	**1.02 [1.01, 1.02]**

Heterogeneity: Chi² = 0.09, df = 1 (P = 0.76); I² = 0%
Test for overall effect: Z = 4.25 (P < 0.0001)

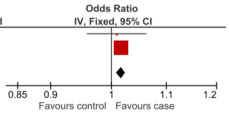

rs7250581

Study or Subgroup	log[Odds Ratio]	SE	Weight	Odds Ratio IV, Random, 95% CI
Burton et al. 2007	0.146	0.063	12.6%	1.16 [1.02, 1.31]
Samani et al. 2007	0.013	0.031	31.3%	1.01 [0.95, 1.08]
Schunkert et al. 2011	0.01	0.008	56.1%	1.01 [0.99, 1.03]
**Total (95% CI)**			**100.0%**	**1.03 [0.98, 1.08]**

Heterogeneity: Tau² = 0.00; Chi² = 4.59, df = 2 (P = 0.10); I² = 56%
Test for overall effect: Z = 1.11 (P = 0.27)

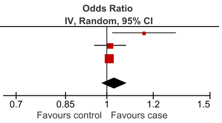

rs72689147

Study or Subgroup	log[Odds Ratio]	SE	Weight	Odds Ratio IV, Fixed, 95% CI
Charmet et al. 2018	0.053	0.046	1.2%	1.05 [0.96, 1.15]
Nikpay et al. 2015	0.029	0.005	98.8%	1.03 [1.02, 1.04]
**Total (95% CI)**			**100.0%**	**1.03 [1.02, 1.04]**

Heterogeneity: Chi² = 0.27, df = 1 (P = 0.60); I² = 0%
Test for overall effect: Z = 5.89 (P < 0.00001)

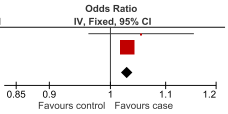

rs72743461

Study or Subgroup	log[Odds Ratio]	SE	Weight	Odds Ratio IV, Fixed, 95% CI
Han et al. 2017	-0.051	0.037	0.5%	0.95 [0.88, 1.02]
Nelson et al. 2017	-0.032	0.005	26.3%	0.97 [0.96, 0.98]
van der Harst et al. 2018	-0.025	0.003	73.2%	0.98 [0.97, 0.98]
**Total (95% CI)**			**100.0%**	**0.97 [0.97, 0.98]**

Heterogeneity: Chi² = 1.87, df = 2 (P = 0.39); I² = 0%
Test for overall effect: Z = 10.51 (P < 0.00001)

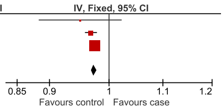

rs73045269

Study or Subgroup	log[Odds Ratio]	SE	Weight	Odds Ratio IV, Fixed, 95% CI
Nelson et al. 2017	0.025	0.005	39.0%	1.03 [1.02, 1.04]
van der Harst et al. 2018	0.03	0.004	61.0%	1.03 [1.02, 1.04]
**Total (95% CI)**			**100.0%**	**1.03 [1.02, 1.03]**

Heterogeneity: Chi² = 0.61, df = 1 (P = 0.43); I² = 0%
Test for overall effect: Z = 8.98 (P < 0.00001)

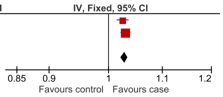

rs7412

Study or Subgroup	log[Odds Ratio]	SE	Weight	Odds Ratio IV, Random, 95% CI
Nelson et al. 2017	-0.056	0.006	47.5%	0.95 [0.93, 0.96]
van der Harst et al. 2018	-0.07	0.005	49.2%	0.93 [0.92, 0.94]
Yamada et al. 2018 (b)	-0.252	0.066	3.2%	0.78 [0.68, 0.88]
**Total (95% CI)**			**100.0%**	**0.93 [0.91, 0.96]**

Heterogeneity: Tau² = 0.00; Chi² = 11.28, df = 2 (P = 0.004); I² = 82%
Test for overall effect: Z = 5.65 (P < 0.00001)

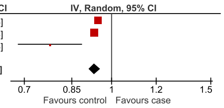

rs742115

Study or Subgroup	log[Odds Ratio]	SE	Weight	Odds Ratio IV, Fixed, 95% CI
Nelson et al. 2017	-0.013	0.003	50.0%	0.99 [0.98, 0.99]
van der Harst et al. 2018	-0.014	0.003	50.0%	0.99 [0.98, 0.99]
**Total (95% CI)**			**100.0%**	**0.99 [0.98, 0.99]**

Heterogeneity: Chi² = 0.06, df = 1 (P = 0.81); I² = 0%
Test for overall effect: Z = 6.36 (P < 0.00001)

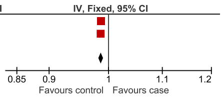

rs74416240

Study or Subgroup	log[Odds Ratio]	SE	Weight	Odds Ratio IV, Fixed, 95% CI
Yamada et al. 2018 (a)	0.164	0.038	45.9%	1.18 [1.09, 1.27]
Yamada et al. 2018 (b)	0.13	0.035	54.1%	1.14 [1.06, 1.22]
**Total (95% CI)**			**100.0%**	**1.16 [1.10, 1.22]**

Heterogeneity: Chi² = 0.43, df = 1 (P = 0.51); I² = 0%
Test for overall effect: Z = 5.66 (P < 0.00001)

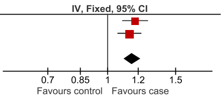

rs74617384

Study or Subgroup	log[Odds Ratio]	SE	Weight	Odds Ratio IV, Random, 95% CI
Fall et al. 2018	0.14	0.02	45.9%	1.15 [1.11, 1.20]
Wei et al. 2018	0.09	0.014	54.1%	1.09 [1.06, 1.12]
**Total (95% CI)**			**100.0%**	**1.12 [1.07, 1.18]**

Heterogeneity: Tau² = 0.00; Chi² = 4.19, df = 1 (P = 0.04); I² = 76%
Test for overall effect: Z = 4.53 (P < 0.00001)

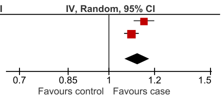

rs748431

Study or Subgroup	log[Odds Ratio]	SE	Weight	Odds Ratio IV, Fixed, 95% CI
Klarin et al. 2017	0.021	0.004	61.0%	1.02 [1.01, 1.03]
Nelson et al. 2017	0.018	0.005	39.0%	1.02 [1.01, 1.03]
**Total (95% CI)**			**100.0%**	**1.02 [1.01, 1.03]**

Heterogeneity: Chi² = 0.22, df = 1 (P = 0.64); I² = 0%
Test for overall effect: Z = 6.35 (P < 0.00001)

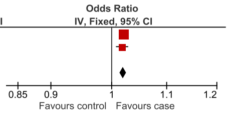

rs7500448

Study or Subgroup	log[Odds Ratio]	SE	Weight	Odds Ratio IV, Fixed, 95% CI
Charmet et al. 2018	0.037	0.043	0.3%	1.04 [0.95, 1.13]
Klarin et al. 2017	0.025	0.005	20.9%	1.03 [1.02, 1.04]
Nelson et al. 2017	0.027	0.005	20.9%	1.03 [1.02, 1.04]
van der Harst et al. 2018	0.027	0.003	58.0%	1.03 [1.02, 1.03]
**Total (95% CI)**			**100.0%**	**1.03 [1.02, 1.03]**

Heterogeneity: Chi² = 0.19, df = 3 (P = 0.98); I² = 0%
Test for overall effect: Z = 11.65 (P < 0.00001)

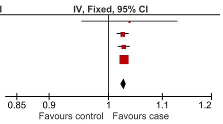

rs7528419

Study or Subgroup	log[Odds Ratio]	SE	Weight	Odds Ratio IV, Fixed, 95% CI
Charmet et al. 2018	0.045	0.043	0.7%	1.05 [0.96, 1.14]
Nelson et al. 2017	0.045	0.005	49.7%	1.05 [1.04, 1.06]
Nikpay et al. 2015	0.049	0.005	49.7%	1.05 [1.04, 1.06]
**Total (95% CI)**			**100.0%**	**1.05 [1.04, 1.06]**

Heterogeneity: Chi² = 0.32, df = 2 (P = 0.85); I² = 0%
Test for overall effect: Z = 13.33 (P < 0.00001)

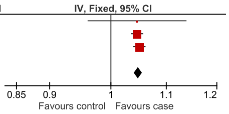

rs7538207

Study or Subgroup	log[Odds Ratio]	SE	Weight	Odds Ratio IV, Fixed, 95% CI
Nelson et al. 2017	-0.041	0.01	39.0%	0.96 [0.94, 0.98]
van der Harst et al. 2018	-0.032	0.008	61.0%	0.97 [0.95, 0.98]
**Total (95% CI)**			**100.0%**	**0.97 [0.95, 0.98]**

Heterogeneity: Chi² = 0.49, df = 1 (P = 0.48); I² = 0%
Test for overall effect: Z = 5.68 (P < 0.00001)

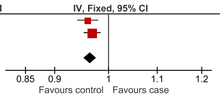

rs7568458

Study or Subgroup	log[Odds Ratio]	SE	Weight	Odds Ratio IV, Fixed, 95% CI
Charmet et al. 2018	0.025	0.036	0.4%	1.03 [0.96, 1.10]
Nelson et al. 2017	0.025	0.003	63.7%	1.03 [1.02, 1.03]
Nikpay et al. 2015	0.025	0.004	35.8%	1.03 [1.02, 1.03]
**Total (95% CI)**			**100.0%**	**1.03 [1.02, 1.03]**

Heterogeneity: Chi² = 0.00, df = 2 (P = 1.00); I² = 0%
Test for overall effect: Z = 10.44 (P < 0.00001)

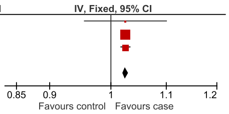

rs7623687

Study or Subgroup	log[Odds Ratio]	SE	Weight	Odds Ratio IV, Fixed, 95% CI
Charmet et al. 2018	-0.018	0.052	0.3%	0.98 [0.89, 1.09]
Klarin et al. 2017	0.033	0.005	28.0%	1.03 [1.02, 1.04]
Nelson et al. 2017	0.032	0.005	28.0%	1.03 [1.02, 1.04]
van der Harst et al. 2018	0.023	0.004	43.7%	1.02 [1.02, 1.03]
**Total (95% CI)**			**100.0%**	**1.03 [1.02, 1.03]**

Heterogeneity: Chi² = 3.98, df = 3 (P = 0.26); I² = 25%
Test for overall effect: Z = 10.66 (P < 0.00001)

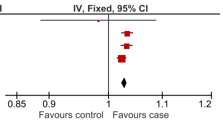

rs7678555

Study or Subgroup	log[Odds Ratio]	SE	Weight	Odds Ratio IV, Fixed, 95% CI
Charmet et al. 2018	-0.036	0.04	0.3%	0.96 [0.89, 1.04]
Klarin et al. 2017	0.025	0.004	26.4%	1.03 [1.02, 1.03]
Nelson et al. 2017	0.022	0.004	26.4%	1.02 [1.01, 1.03]
van der Harst et al. 2018	0.021	0.003	46.9%	1.02 [1.02, 1.03]
**Total (95% CI)**			**100.0%**	**1.02 [1.02, 1.03]**

Heterogeneity: Chi² = 2.77, df = 3 (P = 0.43); I² = 0%
Test for overall effect: Z = 10.79 (P < 0.00001)

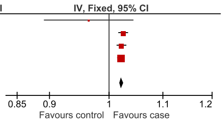

rs7692387

Study or Subgroup	log[Odds Ratio]	SE	Weight	Odds Ratio IV, Random, 95% CI
Dichgans et al. 2014	0.036	0.008	22.4%	1.04 [1.02, 1.05]
Fall et al. 2018	0.013	0.002	30.6%	1.01 [1.01, 1.02]
Han et al. 2017	0.032	0.002	30.6%	1.03 [1.03, 1.04]
van der Harst et al. 2018	0.03	0.012	16.5%	1.03 [1.01, 1.05]
**Total (95% CI)**			**100.0%**	**1.03 [1.01, 1.04]**

Heterogeneity: Tau² = 0.00; Chi² = 48.23, df = 3 (P < 0.00001); I² = 94%
Test for overall effect: Z = 3.78 (P = 0.0002)

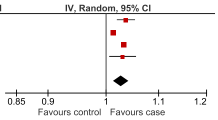

rs781622

Study or Subgroup	log[Odds Ratio]	SE	Weight	Odds Ratio IV, Fixed, 95% CI
Nelson et al. 2017	0.013	0.003	50.0%	1.01 [1.01, 1.02]
van der Harst et al. 2018	0.012	0.003	50.0%	1.01 [1.01, 1.02]
**Total (95% CI)**			**100.0%**	**1.01 [1.01, 1.02]**

Heterogeneity: Chi² = 0.06, df = 1 (P = 0.81); I² = 0%
Test for overall effect: Z = 5.89 (P < 0.00001)

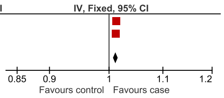

rs7865618

Study or Subgroup	log[Odds Ratio]	SE	Weight	Odds Ratio IV, Random, 95% CI
Aoki et al. 2011	0.033	0.033	13.0%	1.03 [0.97, 1.10]
Samani et al. 2007	0.1	0.015	34.6%	1.11 [1.07, 1.14]
Wild et al. 2011	0.072	0.007	52.4%	1.07 [1.06, 1.09]
**Total (95% CI)**			**100.0%**	**1.08 [1.05, 1.11]**

Heterogeneity: Tau² = 0.00; Chi² = 4.58, df = 2 (P = 0.10); I² = 56%
Test for overall effect: Z = 5.73 (P < 0.00001)

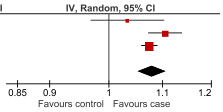

rs7969300

Study or Subgroup	log[Odds Ratio]	SE	Weight	Odds Ratio IV, Random, 95% CI
Yamada et al. 2018 (a)	-0.357	0.057	47.5%	0.70 [0.63, 0.78]
Yamada et al. 2018 (b)	-0.244	0.051	52.5%	0.78 [0.71, 0.87]
**Total (95% CI)**			**100.0%**	**0.74 [0.66, 0.83]**

Heterogeneity: Tau² = 0.00; Chi² = 2.18, df = 1 (P = 0.14); I² = 54%
Test for overall effect: Z = 5.27 (P < 0.00001)

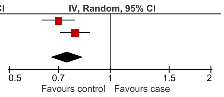

rs79716828

Study or Subgroup	log[Odds Ratio]	SE	Weight	Odds Ratio IV, Random, 95% CI
Nelson et al. 2017	0.049	0.011	45.4%	1.05 [1.03, 1.07]
van der Harst et al. 2018	0.024	0.008	54.6%	1.02 [1.01, 1.04]
**Total (95% CI)**			**100.0%**	**1.04 [1.01, 1.06]**

Heterogeneity: Tau² = 0.00; Chi² = 3.38, df = 1 (P = 0.07); I² = 70%
Test for overall effect: Z = 2.84 (P = 0.005)

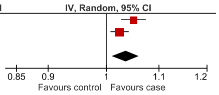

rs8042271

Study or Subgroup	log[Odds Ratio]	SE	Weight	Odds Ratio IV, Random, 95% CI
Charmet et al. 2018	-0.032	0.098	0.8%	0.97 [0.80, 1.17]
Fall et al. 2018	0.004	0.008	27.6%	1.00 [0.99, 1.02]
Han et al. 2017	0.032	0.019	13.1%	1.03 [0.99, 1.07]
Nikpay et al. 2015	0.041	0.008	27.6%	1.04 [1.03, 1.06]
van der Harst et al. 2018	0.032	0.006	30.9%	1.03 [1.02, 1.04]
**Total (95% CI)**			**100.0%**	**1.03 [1.01, 1.04]**

Heterogeneity: Tau² = 0.00; Chi² = 12.45, df = 4 (P = 0.01); I² = 68%
Test for overall effect: Z = 3.05 (P = 0.002)

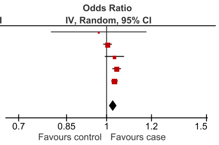

rs8055236

Study or Subgroup	log[Odds Ratio]	SE	Weight	Odds Ratio IV, Random, 95% CI
Burton et al. 2007	0.348	0.079	17.7%	1.42 [1.21, 1.65]
Samani et al. 2007	0.025	0.023	39.0%	1.03 [0.98, 1.07]
Schunkert et al. 2011	0.017	0.008	43.3%	1.02 [1.00, 1.03]
**Total (95% CI)**			**100.0%**	**1.08 [0.99, 1.18]**

Heterogeneity: Tau² = 0.00; Chi² = 17.41, df = 2 (P = 0.0002); I² = 89%
Test for overall effect: Z = 1.83 (P = 0.07)

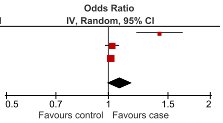

rs8108632

Study or Subgroup	log[Odds Ratio]	SE	Weight	Odds Ratio IV, Fixed, 95% CI
Charmet et al. 2018	0.045	0.037	0.6%	1.05 [0.97, 1.12]
Klarin et al. 2017	0.021	0.004	49.7%	1.02 [1.01, 1.03]
Nelson et al. 2017	0.022	0.004	49.7%	1.02 [1.01, 1.03]
**Total (95% CI)**			**100.0%**	**1.02 [1.02, 1.03]**

Heterogeneity: Chi² = 0.43, df = 2 (P = 0.81); I² = 0%
Test for overall effect: Z = 7.67 (P < 0.00001)

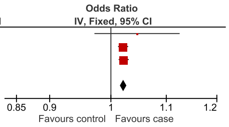

rs833509

Study or Subgroup	log[Odds Ratio]	SE	Weight	Odds Ratio IV, Fixed, 95% CI
Nelson et al. 2017	-0.018	0.005	26.5%	0.98 [0.97, 0.99]
van der Harst et al. 2018	-0.017	0.003	73.5%	0.98 [0.98, 0.99]
**Total (95% CI)**			**100.0%**	**0.98 [0.98, 0.99]**

Heterogeneity: Chi² = 0.03, df = 1 (P = 0.86); I² = 0%
Test for overall effect: Z = 6.71 (P < 0.00001)

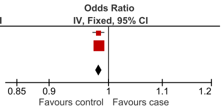

rs867186

Study or Subgroup	log[Odds Ratio]	SE	Weight	Odds Ratio IV, Random, 95% CI
Charmet et al. 2018	0.1	0.057	40.0%	1.11 [0.99, 1.24]
van der Harst et al. 2018	-0.027	0.004	60.0%	0.97 [0.97, 0.98]
**Total (95% CI)**			**100.0%**	**1.02 [0.91, 1.16]**

Heterogeneity: Tau² = 0.01; Chi² = 4.94, df = 1 (P = 0.03); I² = 80%
Test for overall effect: Z = 0.38 (P = 0.70)

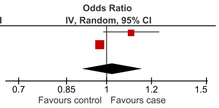

rs886126

Study or Subgroup	log[Odds Ratio]	SE	Weight	Odds Ratio IV, Fixed, 95% CI
Aoki et al. 2011	0.07	0.024	20.0%	1.07 [1.02, 1.12]
Lee et al. 2013	0.057	0.012	80.0%	1.06 [1.03, 1.08]
**Total (95% CI)**			**100.0%**	**1.06 [1.04, 1.08]**

Heterogeneity: Chi² = 0.23, df = 1 (P = 0.63); I² = 0%
Test for overall effect: Z = 5.55 (P < 0.00001)

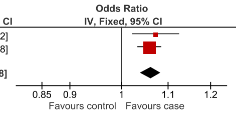

rs916524

Study or Subgroup	log[Odds Ratio]	SE	Weight	Odds Ratio IV, Fixed, 95% CI
Aoki et al. 2011	0.065	0.043	20.7%	1.07 [0.98, 1.16]
Wild et al. 2011	0.072	0.022	79.3%	1.07 [1.03, 1.12]
**Total (95% CI)**			**100.0%**	**1.07 [1.03, 1.12]**

Heterogeneity: Chi² = 0.02, df = 1 (P = 0.88); I² = 0%
Test for overall effect: Z = 3.60 (P = 0.0003)

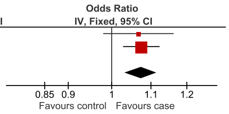

rs918949

Study or Subgroup	log[Odds Ratio]	SE	Weight	Odds Ratio IV, Random, 95% CI
van der Harst et al. 2018	-0.016	0.003	57.3%	0.98 [0.98, 0.99]
Yamada et al. 2018 (b)	-0.102	0.033	42.7%	0.90 [0.85, 0.96]
**Total (95% CI)**			**100.0%**	**0.95 [0.87, 1.03]**

Heterogeneity: Tau² = 0.00; Chi² = 6.74, df = 1 (P = 0.009); I² = 85%
Test for overall effect: Z = 1.24 (P = 0.22)

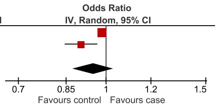

rs925368

Study or Subgroup	log[Odds Ratio]	SE	Weight	Odds Ratio IV, Fixed, 95% CI
Yamada et al. 2018 (a)	0.173	0.04	44.8%	1.19 [1.10, 1.29]
Yamada et al. 2018 (b)	0.137	0.036	55.2%	1.15 [1.07, 1.23]
**Total (95% CI)**			**100.0%**	**1.17 [1.11, 1.23]**

Heterogeneity: Chi² = 0.45, df = 1 (P = 0.50); I² = 0%
Test for overall effect: Z = 5.72 (P < 0.00001)

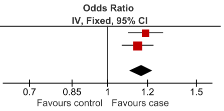

rs9319428

Study or Subgroup	log[Odds Ratio]	SE	Weight	Odds Ratio IV, Random, 95% CI
Charmet et al. 2018	0.013	0.038	1.5%	1.01 [0.94, 1.09]
Dichgans et al. 2014	0.025	0.008	18.0%	1.03 [1.01, 1.04]
Fall et al. 2018	0.004	0.002	35.0%	1.00 [1.00, 1.01]
Han et al. 2017	0.021	0.018	5.8%	1.02 [0.99, 1.06]
Hirokawa et al. 2015	0.009	0.02	4.8%	1.01 [0.97, 1.05]
van der Harst et al. 2018	0.016	0.002	35.0%	1.02 [1.01, 1.02]
**Total (95% CI)**			**100.0%**	**1.01 [1.00, 1.02]**

Heterogeneity: Tau² = 0.00; Chi² = 21.76, df = 5 (P = 0.0006); I² = 77%
Test for overall effect: Z = 2.83 (P = 0.005)

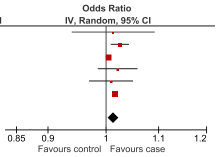

rs9349379

Study or Subgroup	log[Odds Ratio]	SE	Weight	Odds Ratio IV, Random, 95% CI
Charmet et al. 2018	0.029	0.036	3.8%	1.03 [0.96, 1.10]
Hager et al. 2012	0.126	0.021	6.7%	1.13 [1.09, 1.18]
Han et al. 2017	0.061	0.015	8.3%	1.06 [1.03, 1.09]
Klarin et al. 2017	0.061	0.009	9.8%	1.06 [1.04, 1.08]
Lu et al. 2012	0.061	0.011	9.3%	1.06 [1.04, 1.09]
Nelson et al. 2017	0.045	0.004	10.7%	1.05 [1.04, 1.05]
Nikpay et al. 2015	0.057	0.004	10.7%	1.06 [1.05, 1.07]
O'Donnell et al. 2011	0.053	0.013	8.8%	1.05 [1.03, 1.08]
Pechlivanis et al. 2013	0.1	0.023	6.2%	1.11 [1.06, 1.16]
van der Harst et al. 2018	0.052	0.003	10.9%	1.05 [1.05, 1.06]
Winsvold et al. 2017	-0.022	0.006	10.4%	0.98 [0.97, 0.99]
Yamada et al. 2018 (b)	0.097	0.033	4.2%	1.10 [1.03, 1.18]
**Total (95% CI)**			**100.0%**	**1.06 [1.04, 1.08]**

Heterogeneity: Tau² = 0.00; Chi² = 168.57, df = 11 (P < 0.00001); I² = 93%
Test for overall effect: Z = 6.41 (P < 0.00001)

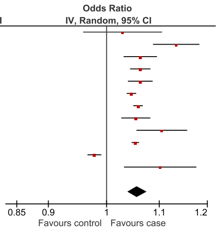

rs9369640

Study or Subgroup	log[Odds Ratio]	SE	Weight	Odds Ratio IV, Random, 95% CI
Dichgans et al. 2014	-0.041	0.006	60.8%	0.96 [0.95, 0.97]
Yamada et al. 2018 (b)	-0.137	0.045	39.2%	0.87 [0.80, 0.95]
**Total (95% CI)**			**100.0%**	**0.92 [0.84, 1.01]**

Heterogeneity: Tau² = 0.00; Chi² = 4.47, df = 1 (P = 0.03); I² = 78%
Test for overall effect: Z = 1.68 (P = 0.09)

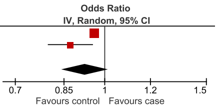

rs944797

Study or Subgroup	log[Odds Ratio]	SE	Weight	Odds Ratio IV, Fixed, 95% CI
Han et al. 2017	0.083	0.014	28.0%	1.09 [1.06, 1.12]
Hirokawa et al. 2015	0.083	0.018	16.9%	1.09 [1.05, 1.13]
Lee et al. 2013	0.1	0.018	16.9%	1.11 [1.07, 1.14]
Takeuchi et al. 2012	0.097	0.012	38.1%	1.10 [1.08, 1.13]
**Total (95% CI)**			**100.0%**	**1.10 [1.08, 1.11]**

Heterogeneity: Chi² = 1.02, df = 3 (P = 0.80); I² = 0%
Test for overall effect: Z = 12.31 (P < 0.00001)

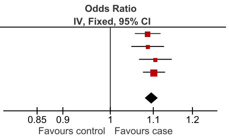

rs9501744

Study or Subgroup	log[Odds Ratio]	SE	Weight	Odds Ratio IV, Fixed, 95% CI
Nelson et al. 2017	-0.027	0.006	30.8%	0.97 [0.96, 0.98]
van der Harst et al. 2018	-0.022	0.004	69.2%	0.98 [0.97, 0.99]
**Total (95% CI)**			**100.0%**	**0.98 [0.97, 0.98]**

Heterogeneity: Chi² = 0.48, df = 1 (P = 0.49); I² = 0%
Test for overall effect: Z = 7.07 (P < 0.00001)

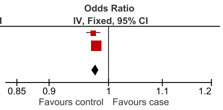

rs9515203

Study or Subgroup	log[Odds Ratio]	SE	Weight	Odds Ratio IV, Random, 95% CI
Charmet et al. 2018	0.079	0.044	2.6%	1.08 [0.99, 1.18]
Dichgans et al. 2014	0.036	0.008	20.5%	1.04 [1.02, 1.05]
Fall et al. 2018	0.004	0.002	26.3%	1.00 [1.00, 1.01]
Nelson et al. 2017	0.025	0.004	24.9%	1.03 [1.02, 1.03]
van der Harst et al. 2018	0.026	0.003	25.7%	1.03 [1.02, 1.03]
**Total (95% CI)**			**100.0%**	**1.02 [1.01, 1.04]**

Heterogeneity: Tau² = 0.00; Chi² = 58.28, df = 4 (P < 0.00001); I² = 93%
Test for overall effect: Z = 3.13 (P = 0.002)

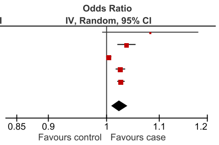

rs9591012

Study or Subgroup	log[Odds Ratio]	SE	Weight	Odds Ratio IV, Fixed, 95% CI
Nelson et al. 2017	-0.018	0.003	50.0%	0.98 [0.98, 0.99]
van der Harst et al. 2018	-0.018	0.003	50.0%	0.98 [0.98, 0.99]
**Total (95% CI)**			**100.0%**	**0.98 [0.98, 0.99]**

Heterogeneity: Chi² = 0.00, df = 1 (P = 1.00); I² = 0%
Test for overall effect: Z = 8.49 (P < 0.00001)

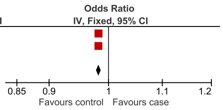

rs9632884

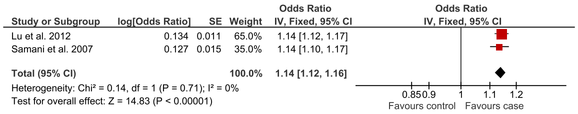

rs964184

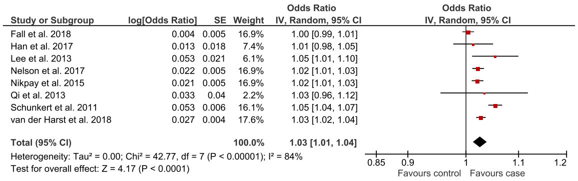

rs974819

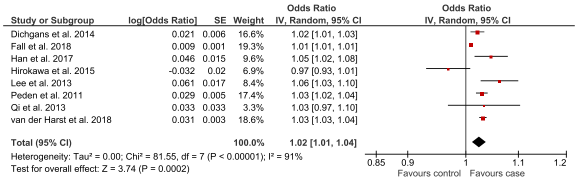

rs9818870

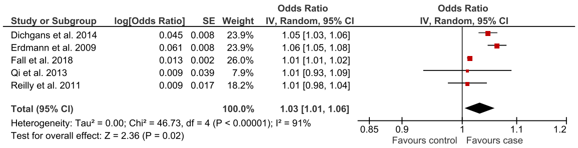

rs9970807

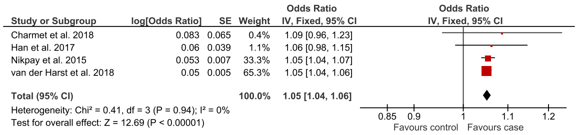

rs9982601

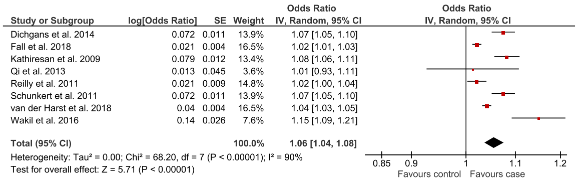

Figure S1. Forest plots for all single nucleotide polymorphisms from 44 GWAS publications included in this paper except for rs4593108.
