# Supplementary material for: Genetic Factors for Coronary Heart Disease and Their Mechanisms: A Meta-Analysis and Comprehensive Review of Common Variants from Genome-Wide Association Studies
Source: Diagnostics (Basel). 2022 Oct 21;12(10):2561. doi: 10.3390/diagnostics12102561 (PMC9601486; doi:10.3390/diagnostics12102561)
Supplement: Supplementary file 1 [file diagnostics-12-02561-s001.zip › Figure S2.pdf]

a) Before correction

Study or Subgroup	log[Odds Ratio]	SE	Weight	Odds Ratio IV, Fixed, 95% CI
Aoki et al. 2011	0.004	0.025	1.1%	1.00 [0.96, 1.05]
Charmet et al. 2018	-0.032	0.045	0.3%	0.97 [0.89, 1.06]
Han et al. 2017	0.022	0.015	3.0%	1.02 [0.99, 1.05]
Nelson et al. 2017	0.029	0.005	26.8%	1.03 [1.02, 1.04]
Nikpay et al. 2015	0.029	0.005	26.8%	1.03 [1.02, 1.04]
van der Harst et al. 2018	0.027	0.004	41.9%	1.03 [1.02, 1.04]
**Total (95% CI)**			**100.0%**	**1.03 [1.02, 1.03]**

Heterogeneity: Chi² = 2.96, df = 5 (P = 0.71); I² = 0%
Test for overall effect: Z = 10.61 (P < 0.00001)

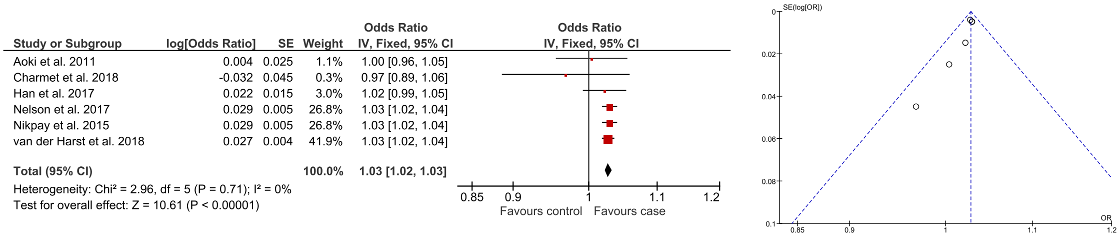

b) After correction

Study or Subgroup	log[Odds Ratio]	SE	Weight	Odds Ratio IV, Fixed, 95% CI
Aoki et al. 2011	0.004	0.025	1.1%	1.00 [0.96, 1.05]
Han et al. 2017	0.022	0.015	3.0%	1.02 [0.99, 1.05]
Nelson et al. 2017	0.029	0.005	26.9%	1.03 [1.02, 1.04]
Nikpay et al. 2015	0.029	0.005	26.9%	1.03 [1.02, 1.04]
van der Harst et al. 2018	0.027	0.004	42.1%	1.03 [1.02, 1.04]
**Total (95% CI)**			**100.0%**	**1.03 [1.02, 1.03]**

Heterogeneity: Chi² = 1.21, df = 4 (P = 0.88); I² = 0%
Test for overall effect: Z = 10.67 (P < 0.00001)

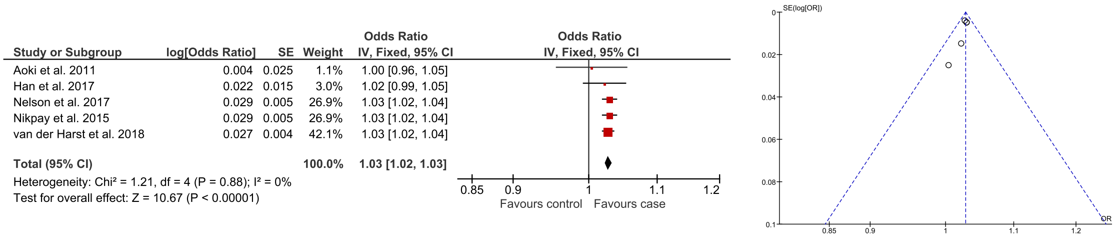

Figure S2. Forest and funnel plots for rs4593108 before and after correction for publication bias.
