# Supplementary material for: Genetic Factors for Coronary Heart Disease and Their Mechanisms: A Meta-Analysis and Comprehensive Review of Common Variants from Genome-Wide Association Studies
Source: Diagnostics (Basel). 2022 Oct 21;12(10):2561. doi: 10.3390/diagnostics12102561 (PMC9601486; doi:10.3390/diagnostics12102561)
Supplement: Supplementary file 1 [file diagnostics-12-02561-s001.zip › Figure S3.pdf]

rs10139550

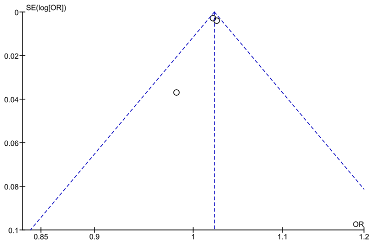

rs10168194

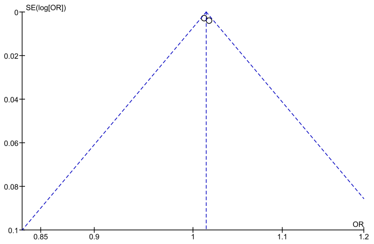

rs10237377

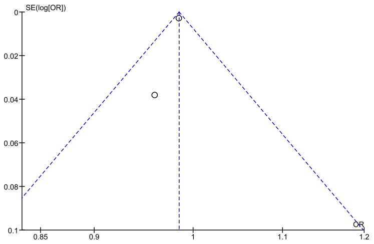

rs10455872

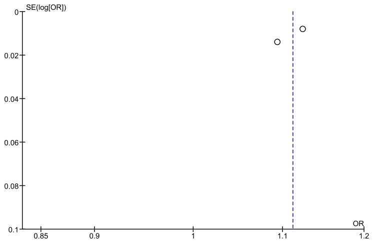

rs1050362

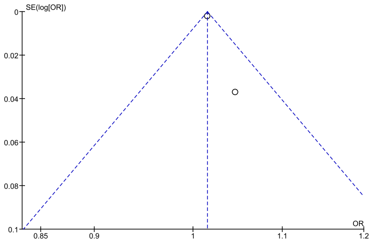

rs10738607

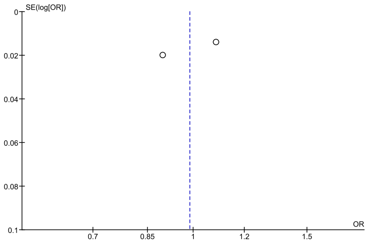

rs10738609

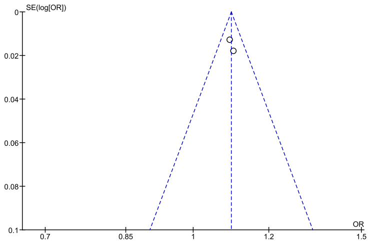

rs10746041

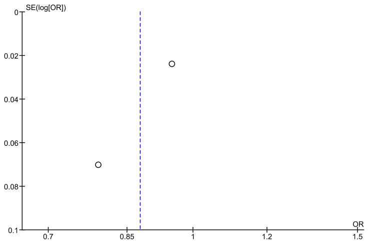

rs10757264

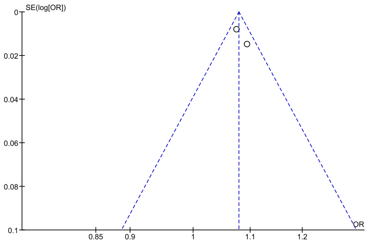

rs10757270

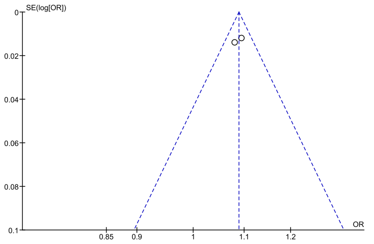

rs10757272

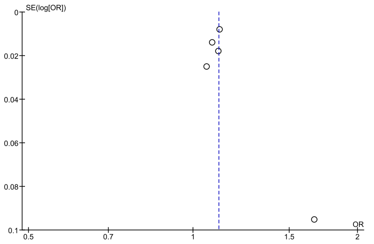

rs10757274

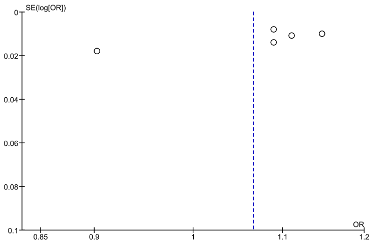

rs10757275

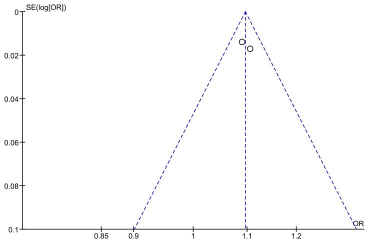

rs10757278

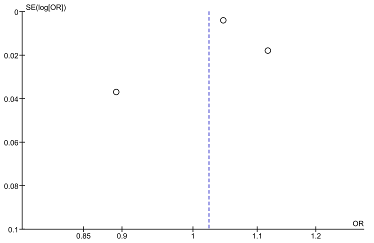

rs10774610

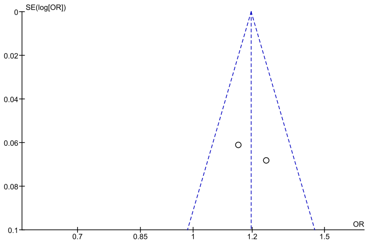

rs10840293

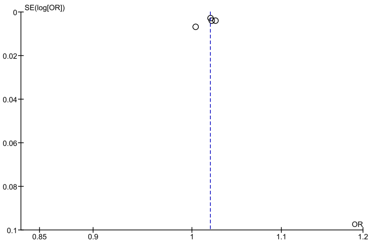

rs10841443

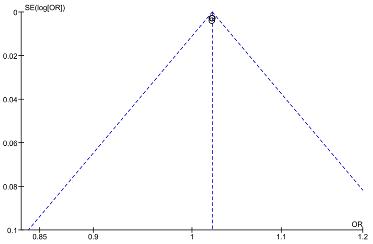

rs10849915

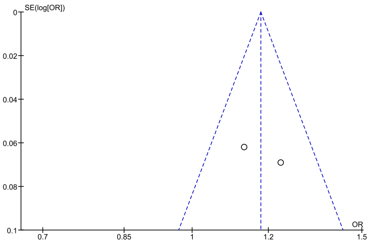

rs10857147

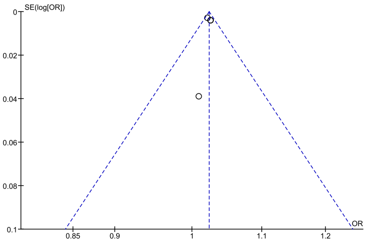

rs1088868

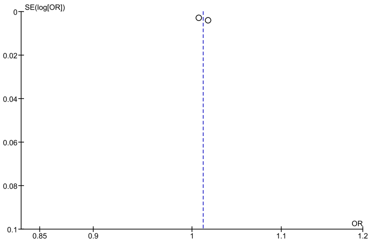

rs10947262

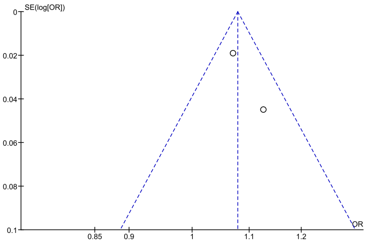

rs10947789

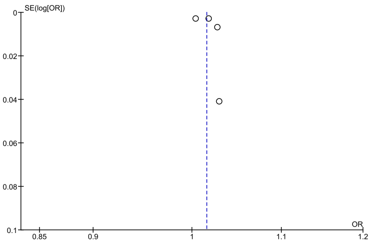

rs10953541

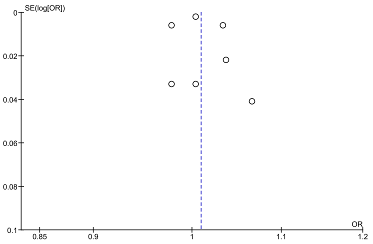

rs10965212

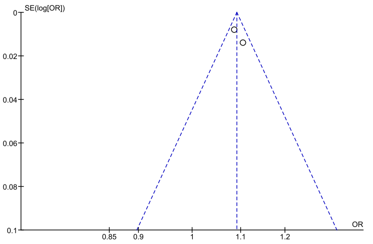

rs10965215

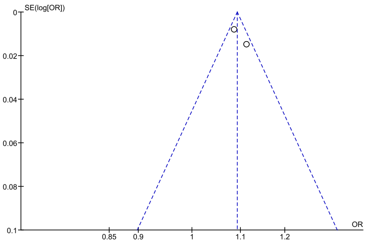

rs10965219

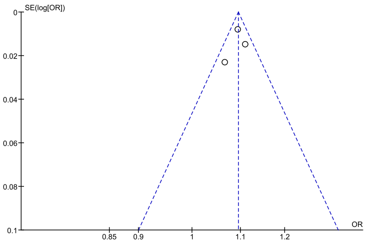

rs11042937

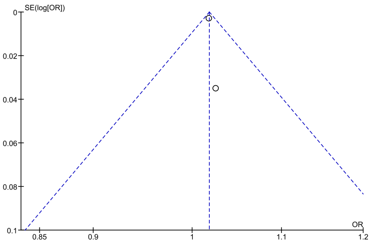

rs11057401

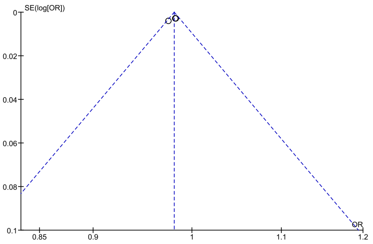

rs11057830

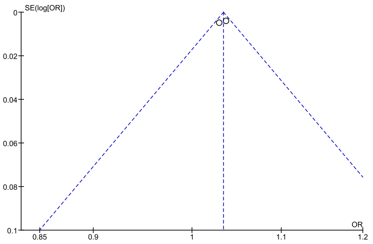

rs11065979

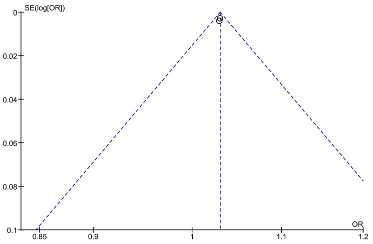

rs11066001

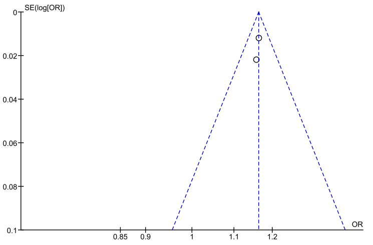

rs11066015

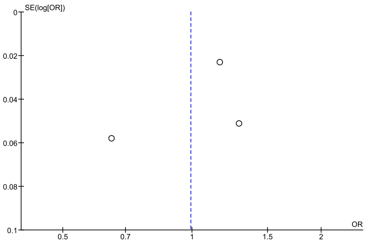

rs11066280

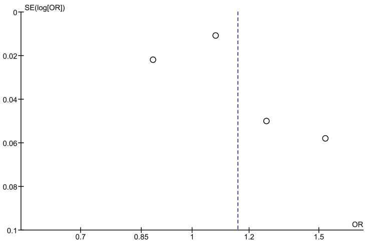

rs11099493

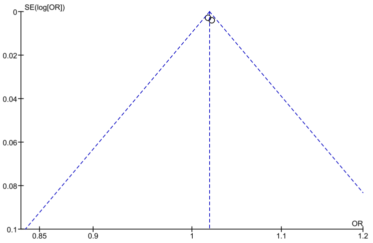

rs111245230

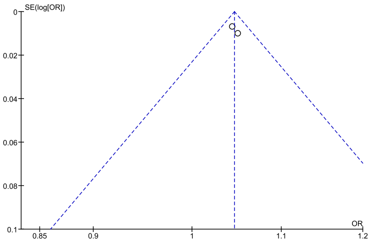

rs11170820

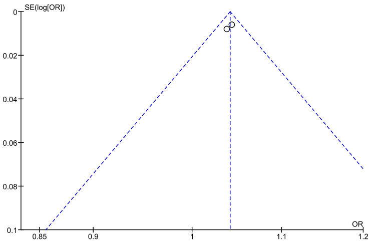

rs11172113

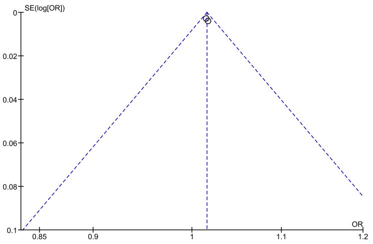

rs11191416

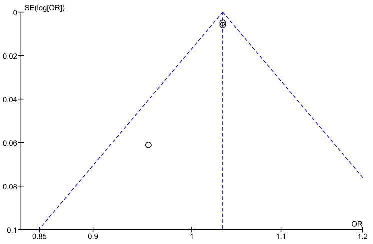

rs11203042

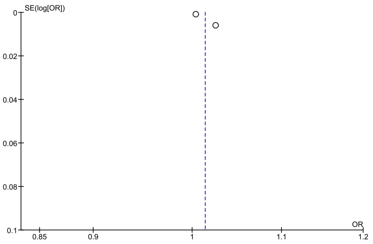

rs11206510

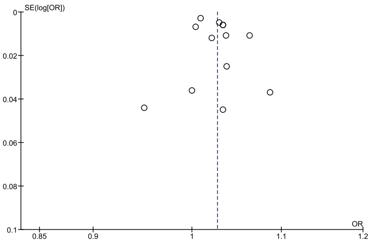

rs1122608

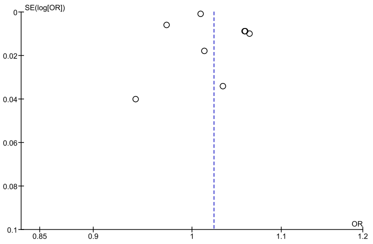

rs112635299

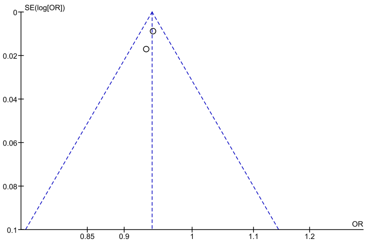

rs112941079

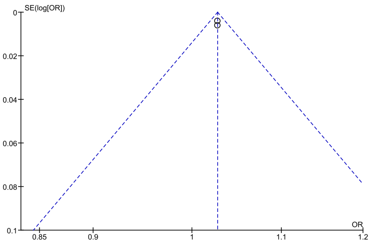

rs11509880

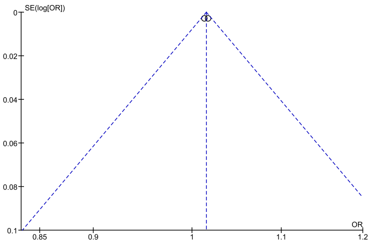

rs115287176

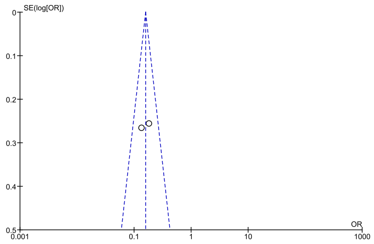

rs11556924

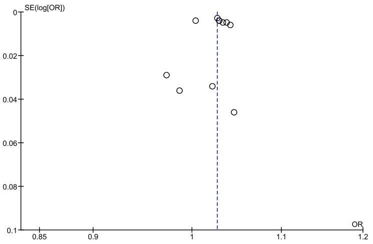

rs11591147

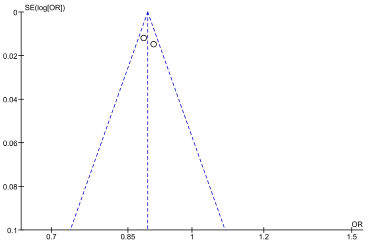

rs11617955

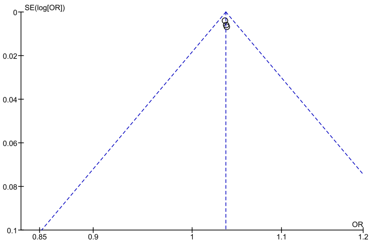

rs116843064

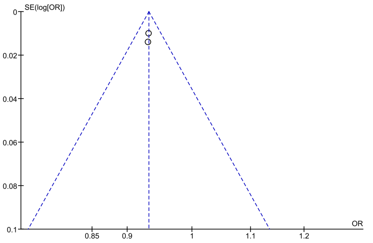

rs11728590

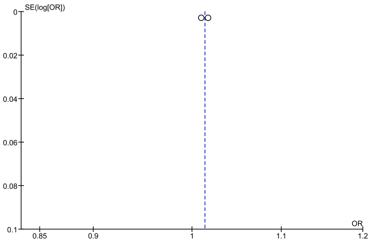

rs11752643

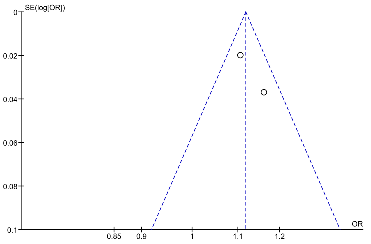

rs11810571

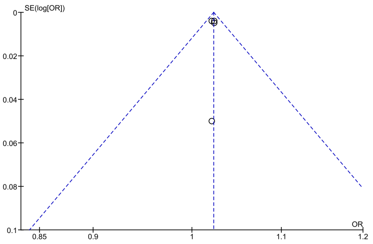

rs11823828

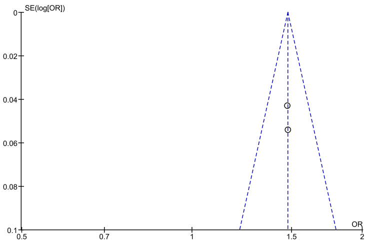

rs11838776

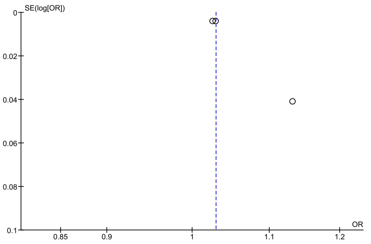

rs12146487

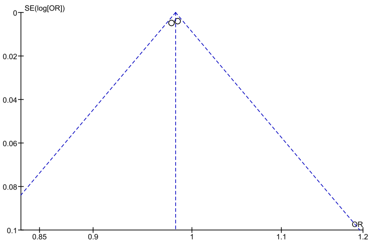

rs1214752

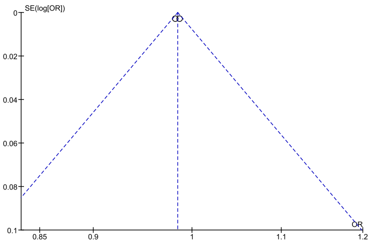

rs12190287

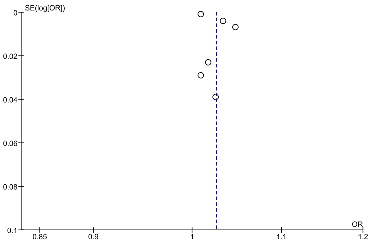

rs12202017

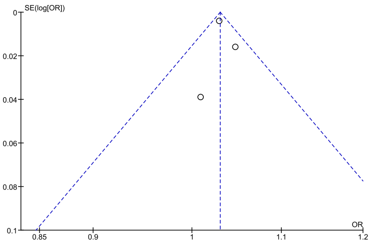

rs12229654

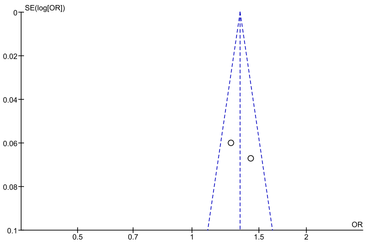

rs12231744

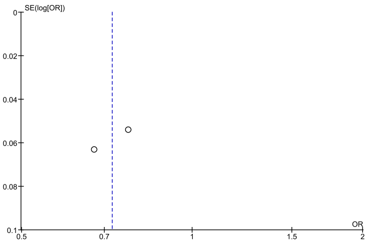

rs12413409

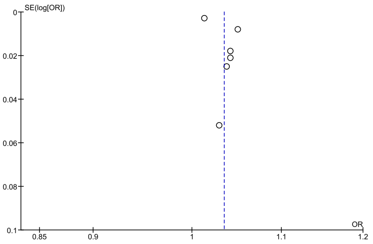

rs12493885

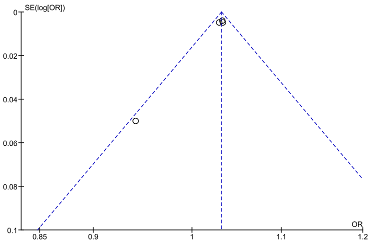

rs1250229

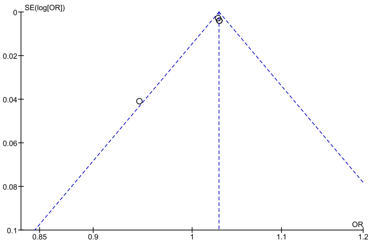

rs12524865

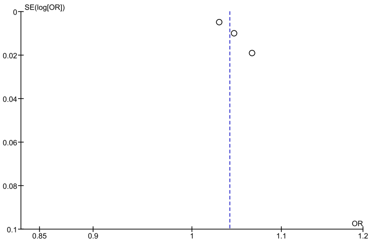

rs12526453

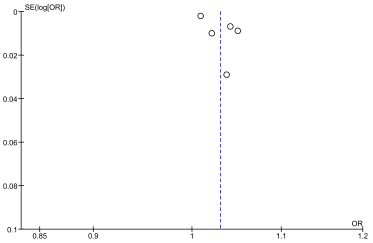

rs12619842

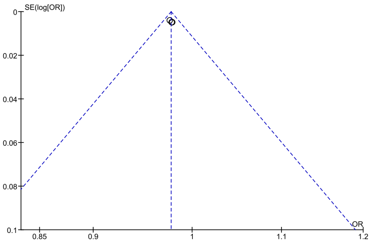

rs12740374

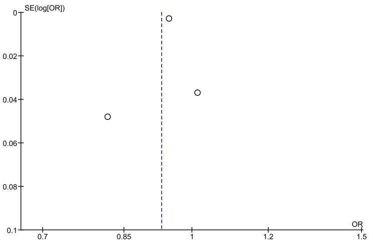

rs12801636

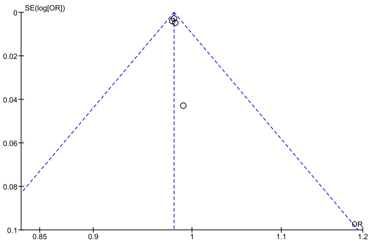

rs12922

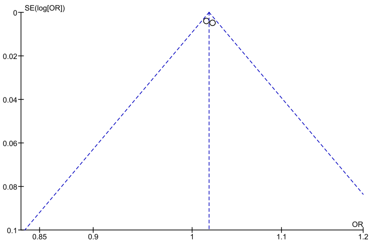

rs12936587

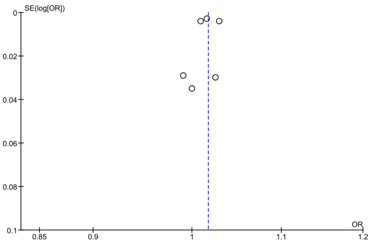

rs13003675

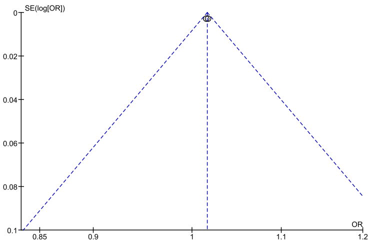

rs1333042

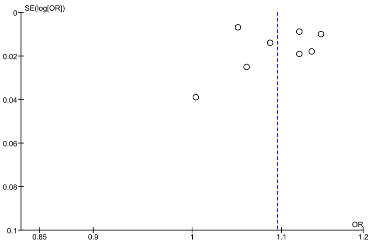

rs1333045

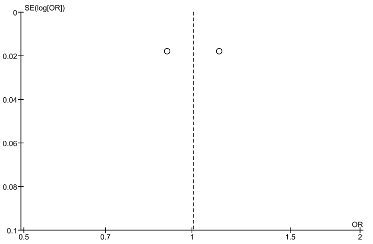

rs1333048

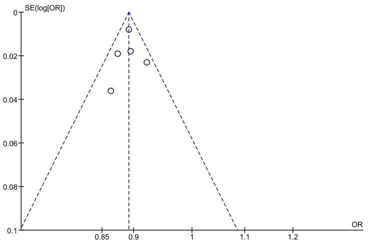

rs1333049

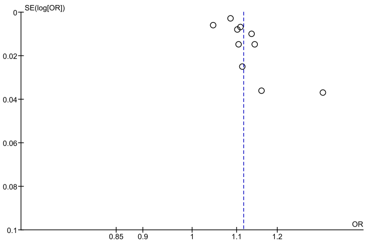

rs13723

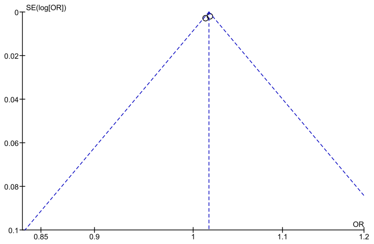

rs13734

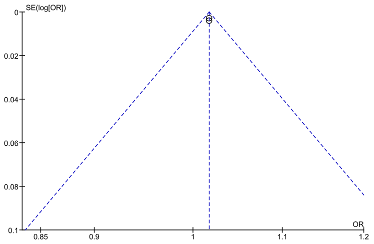

rs1378942

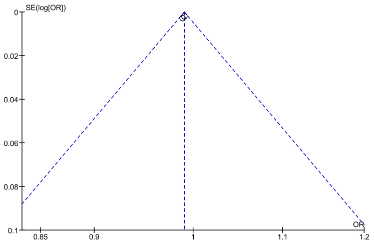

rs140570886

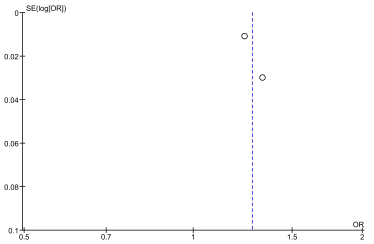

rs1412444

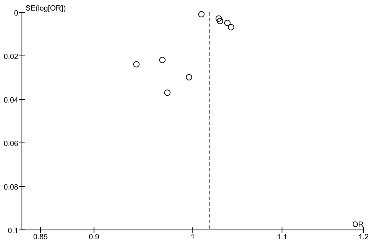

rs1412834

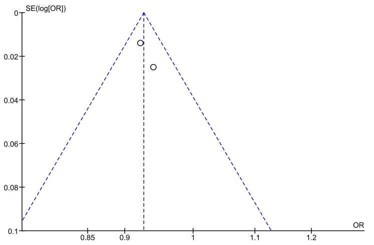

rs142695226

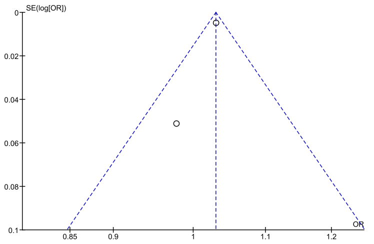

rs143803699

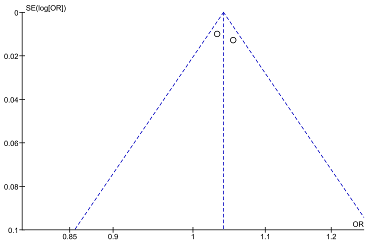

rs146092501

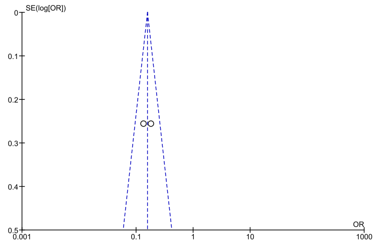

rs146879198

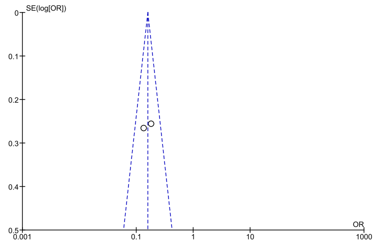

rs147555597

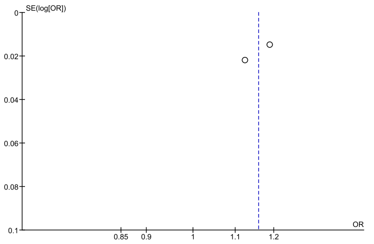

rs1537372

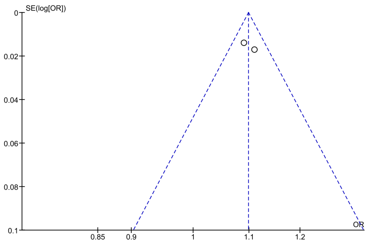

rs1537373

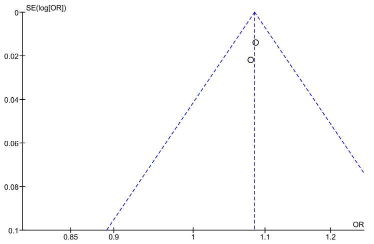

rs1537376

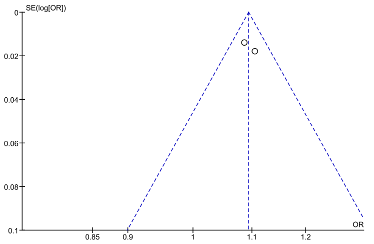

rs1561198

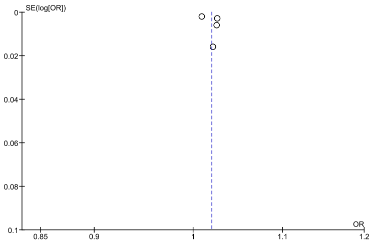

rs167479

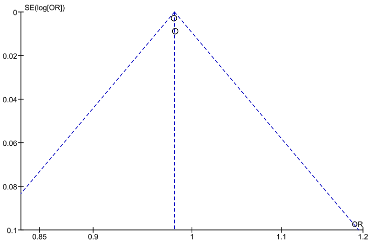

rs16986953

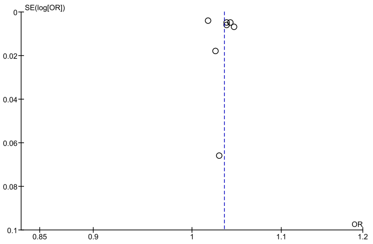

rs17087335

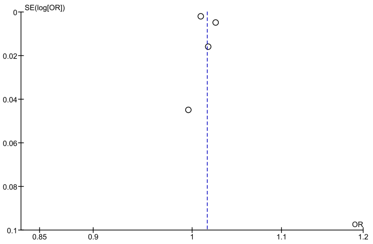

rs17114036

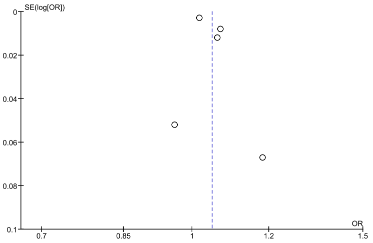

rs17228212

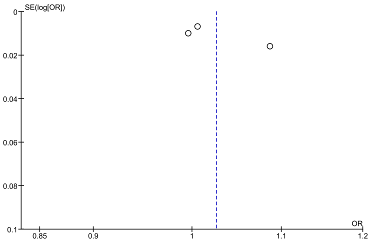

rs17411031

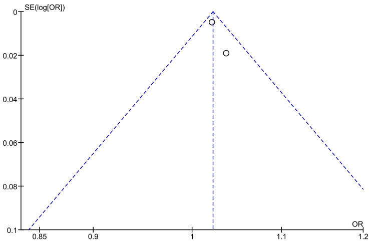

rs1746048

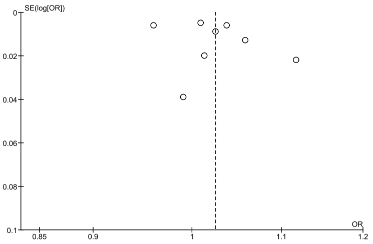

rs1746049

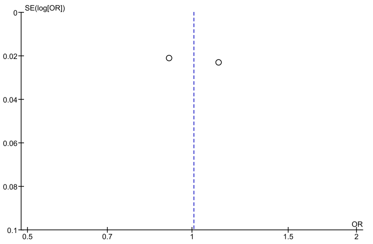

rs17464857

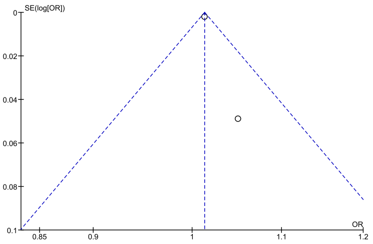

rs17465637

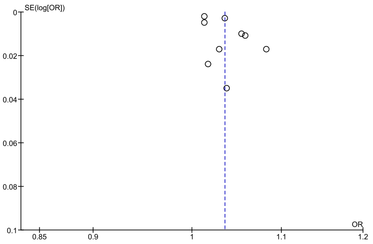

rs17514846

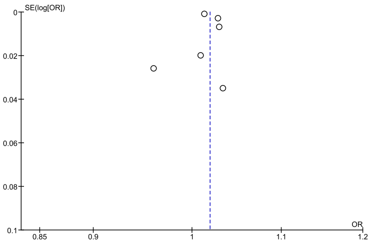

rs17517928

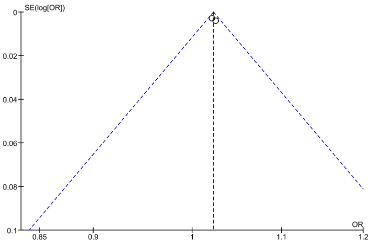

rs17581137

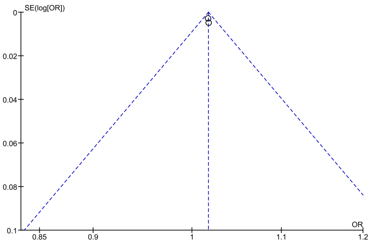

rs17608766

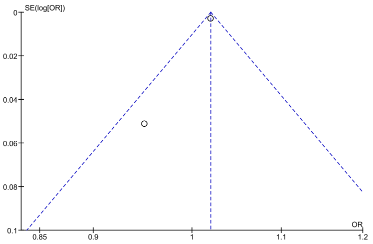

rs17609940

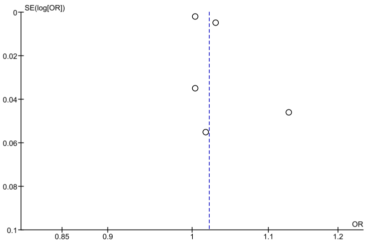

rs17672135

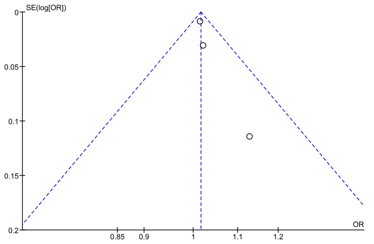

rs17678683

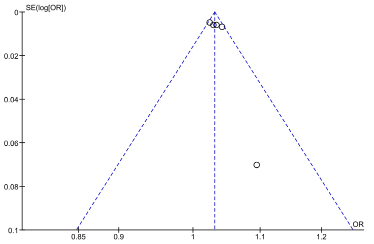

rs17843797

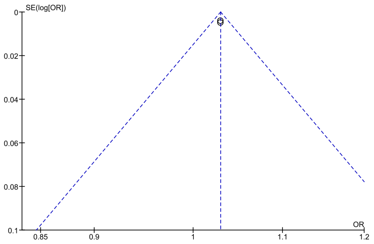

rs1800449

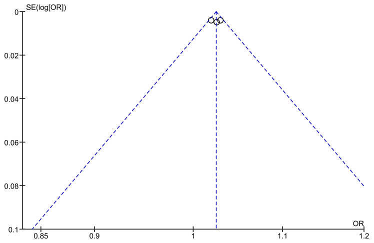

rs1800775

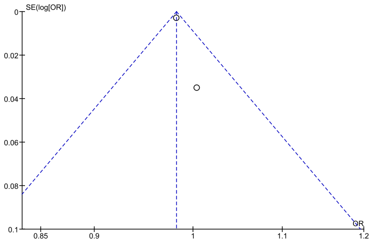

rs1801251

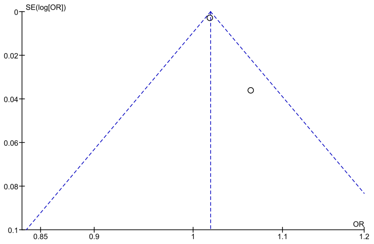

rs180803

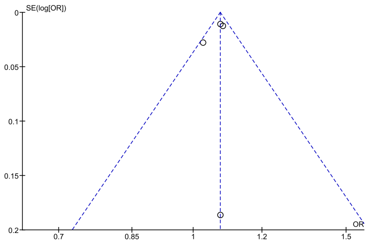

rs1831733

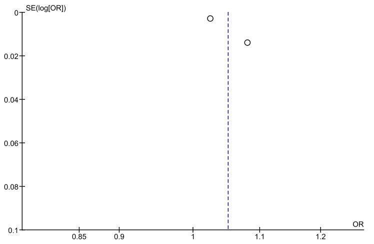

rs1842896

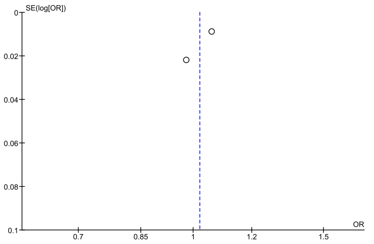

rs186696265

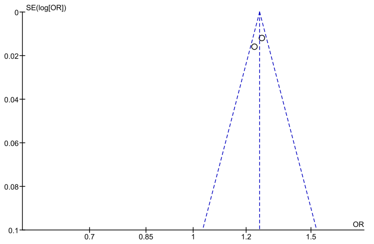

rs1867624

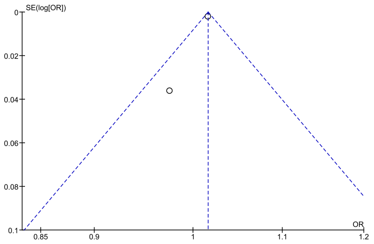

rs1870634

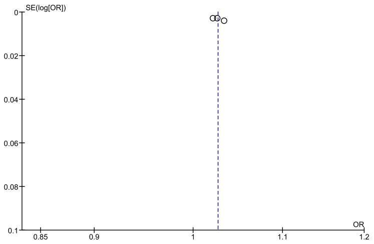

rs1878406

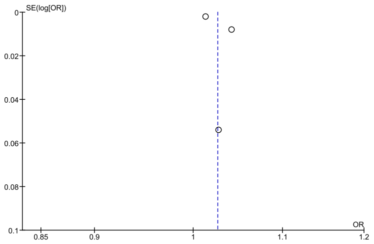

rs188378669

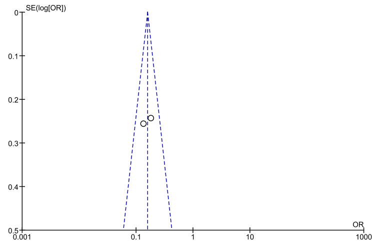

rs1883838

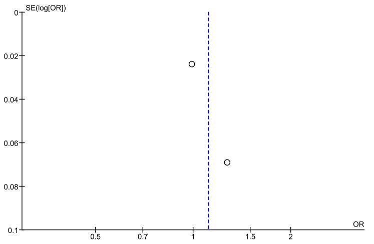

rs1892094

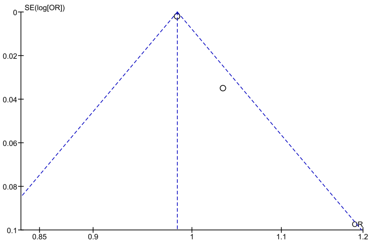

rs192210727

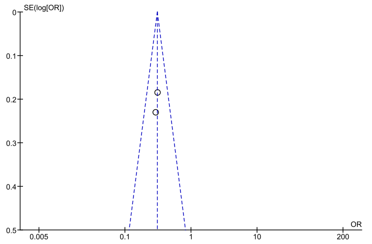

rs1924981

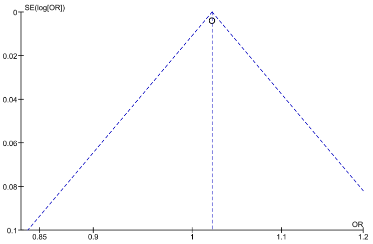

rs194937

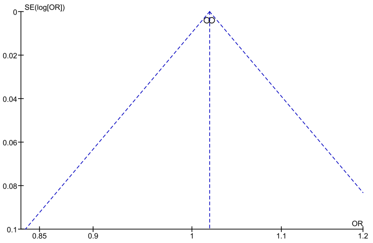

rs1964272

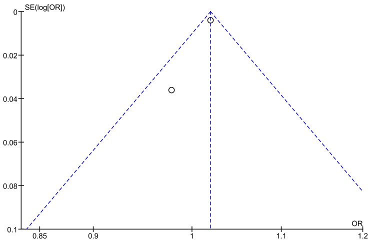

rs1994016

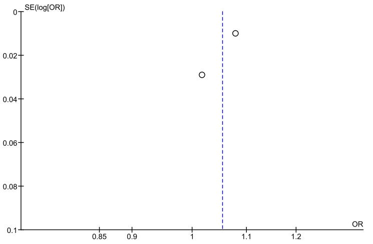

rs199921354

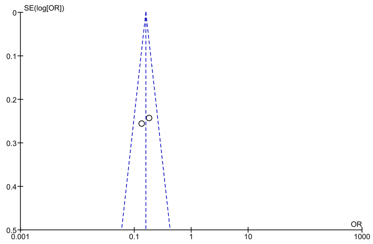

rs2000999

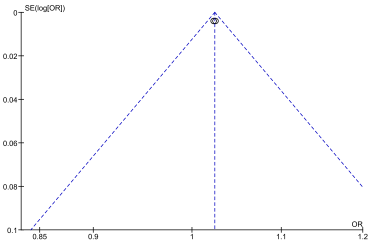

rs200787930

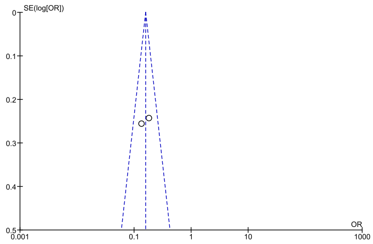

rs2011559

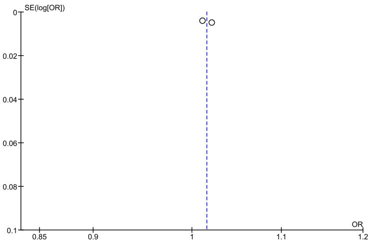

rs2023938

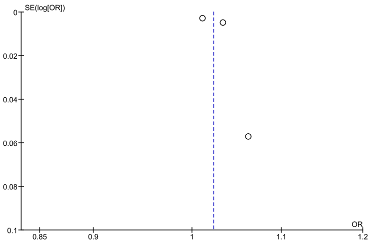

rs2047009

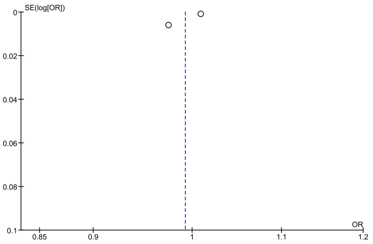

rs2048327

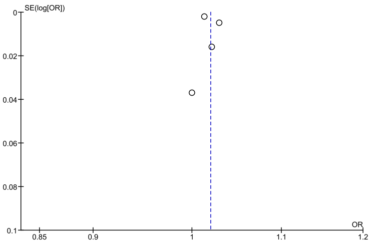

rs2072134

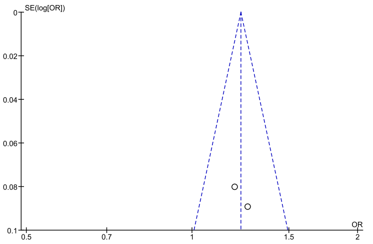

rs2074356

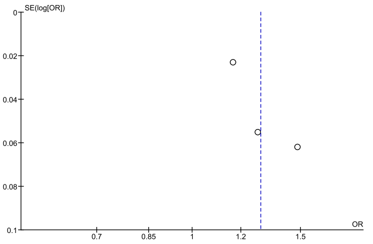

rs2075650

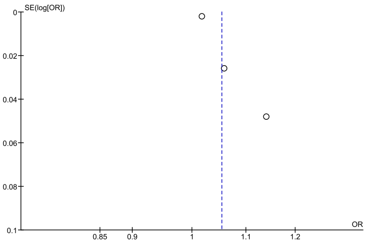

rs2107595

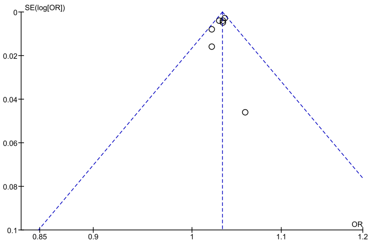

rs2128739

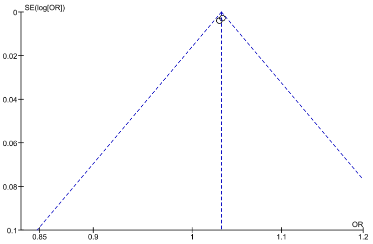

rs2153219

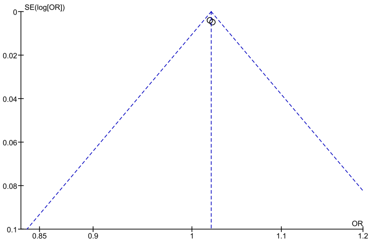

rs216172

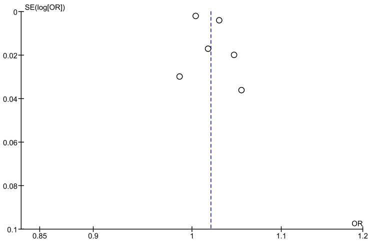

rs220702

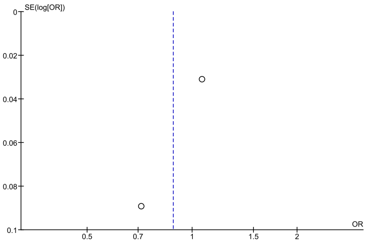

rs2241714

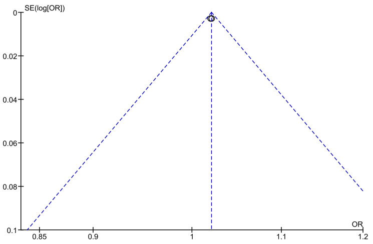

rs2244608

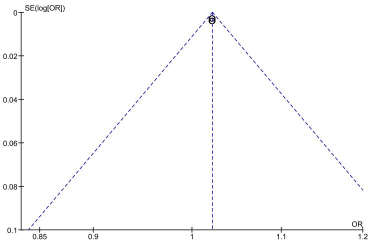

rs2246833

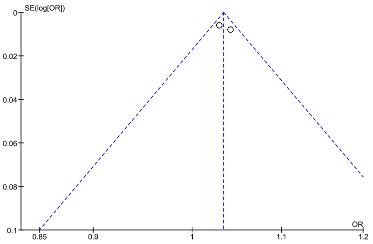

rs2246942

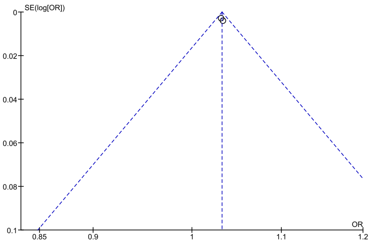

rs2252641

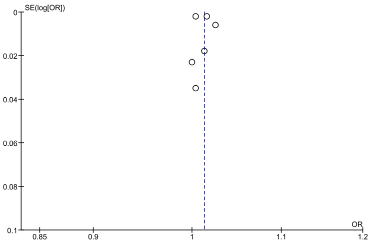

rs2257129

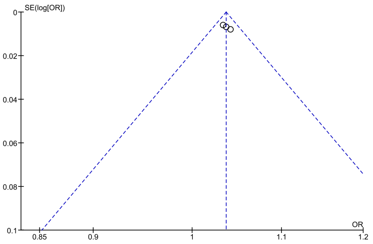

rs2259816

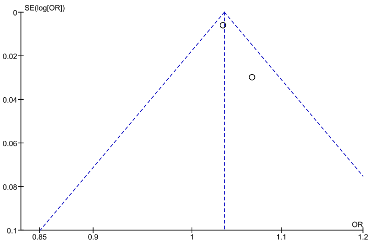

rs2306374

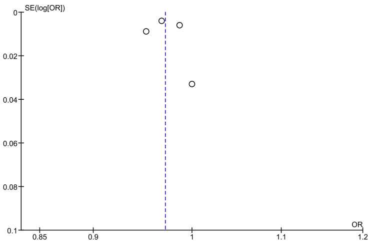

rs2315065

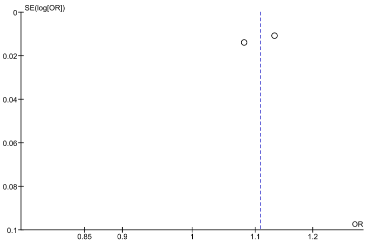

rs2327429

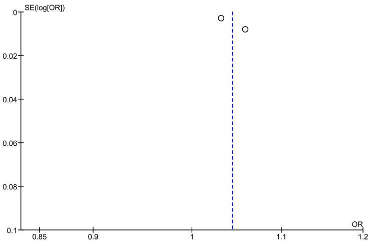

rs2383206

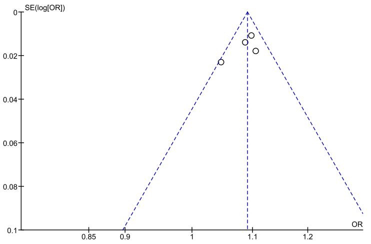

rs2383207

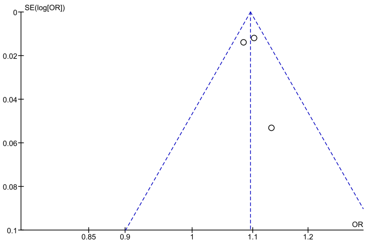

rs246600

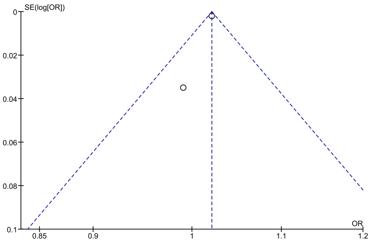

rs2487928

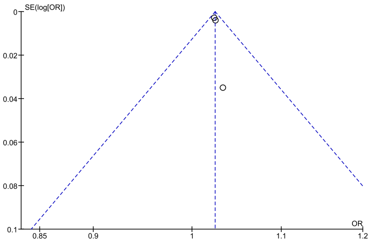

rs2505083

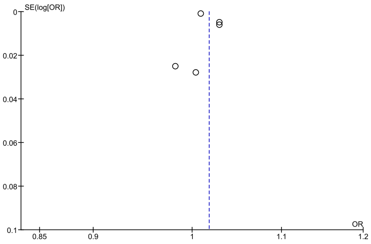

rs2519093

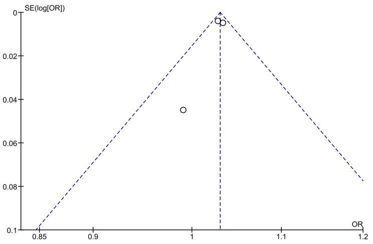

rs2523644

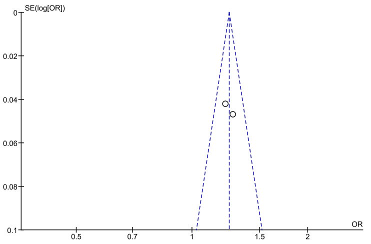

rs2571445

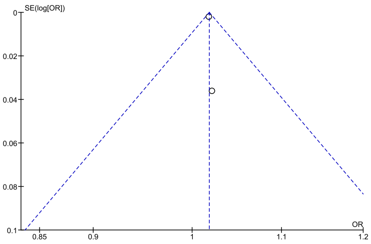

rs2596548

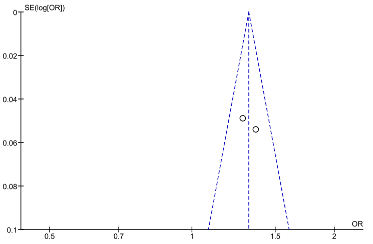

rs2616407

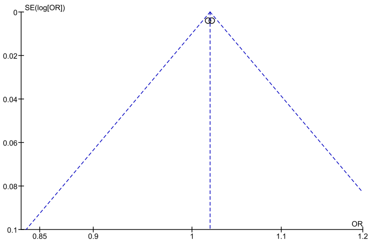

rs264

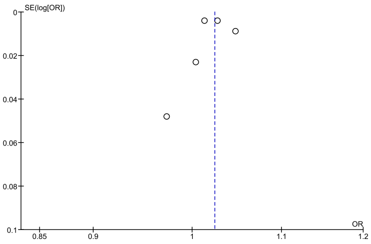

rs2681472

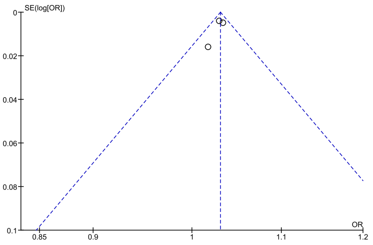

rs2727020

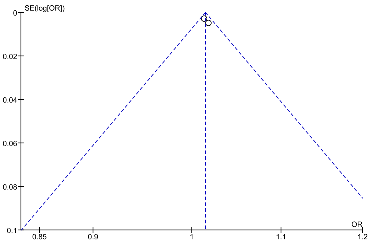

rs273909

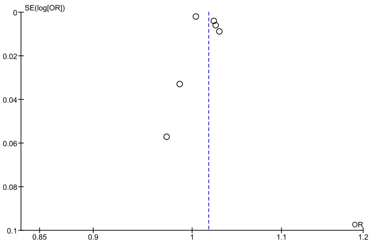

rs2789422

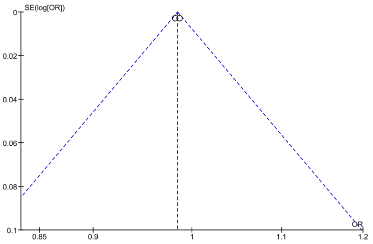

rs2820315

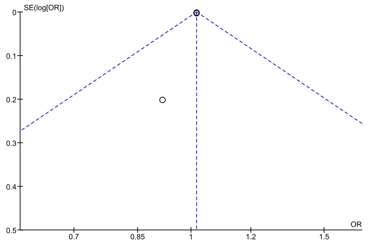

rs2843152

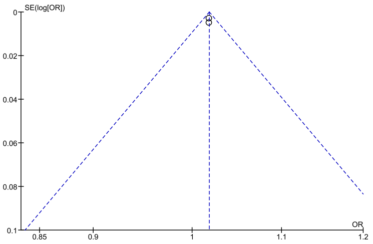

rs28451064

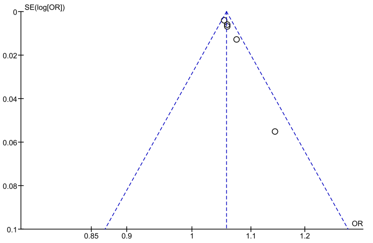

rs2880099

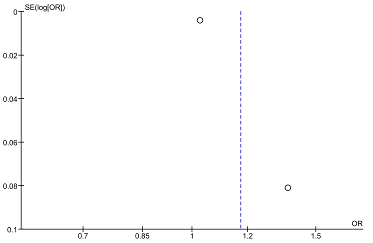

rs288187

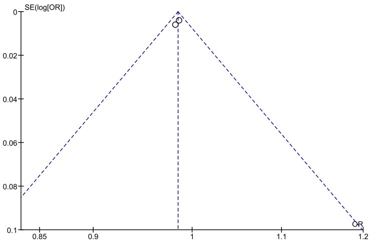

rs2891168

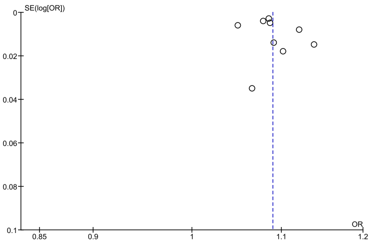

rs2895811

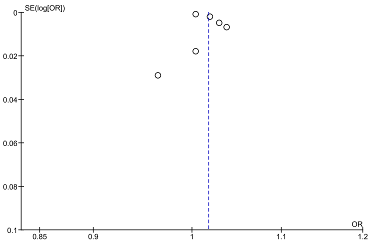

rs2943634

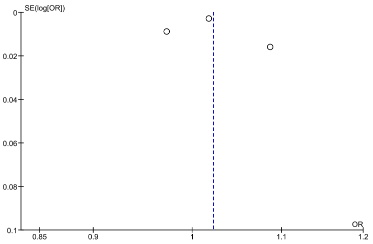

rs2954029

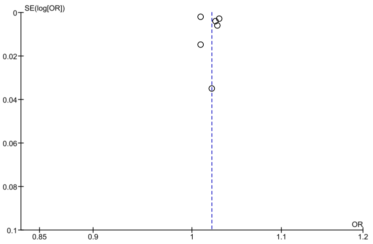

rs2972146

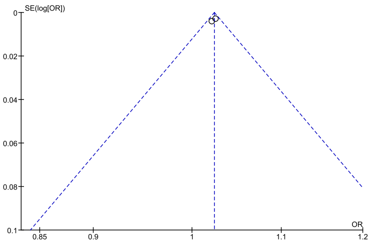

rs3130683

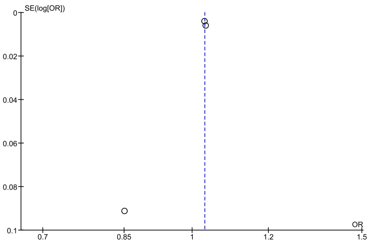

rs3133293

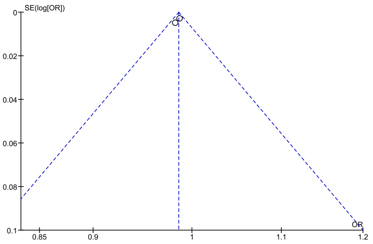

rs3184504

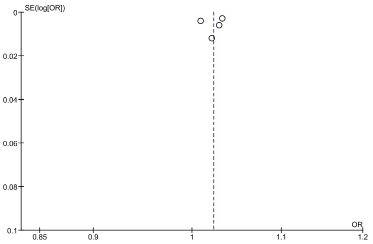

rs3217992

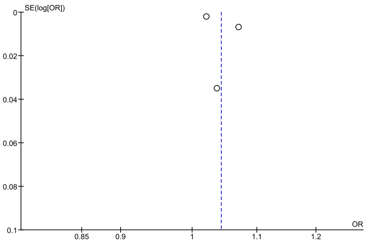

rs328

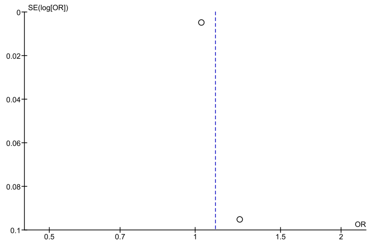

rs35146811

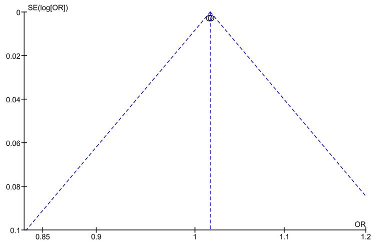

rs35465346

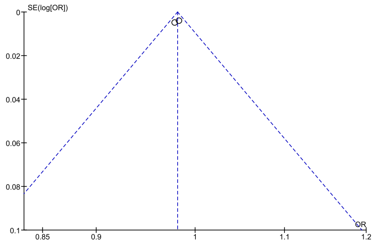

rs35489971

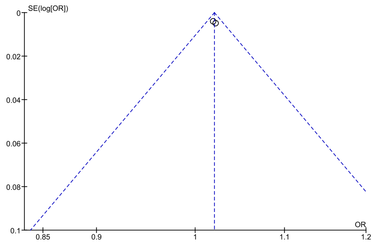

rs35895680

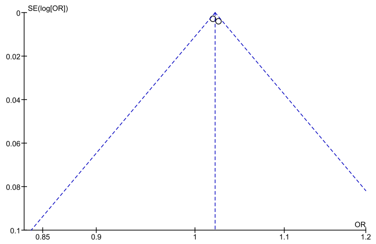

rs3739998

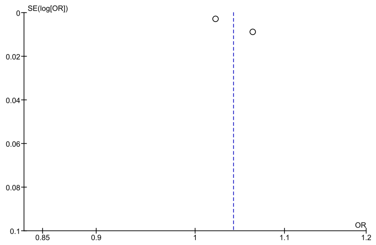

rs3782774

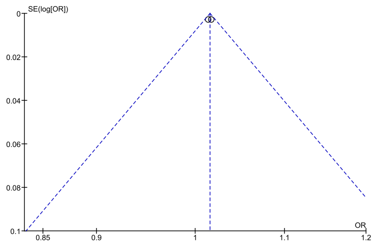

rs3782886

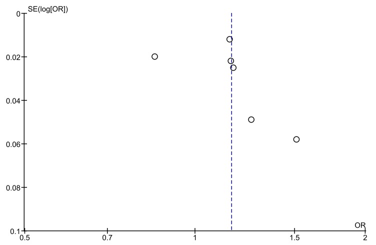

rs3798220

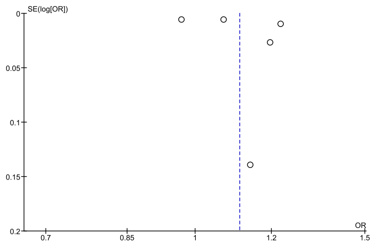

rs3809346

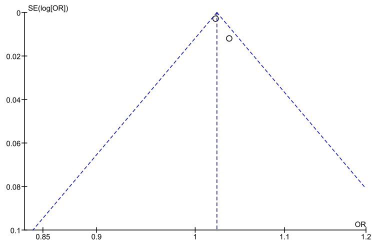

rs3825807

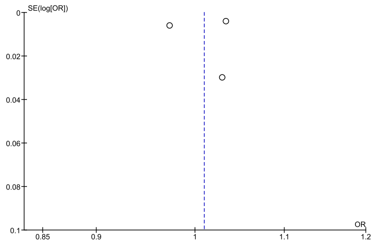

rs383830

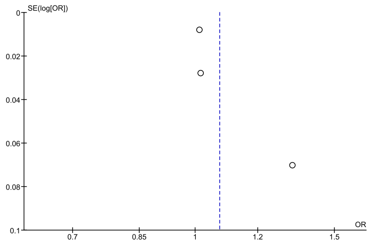

rs3918226

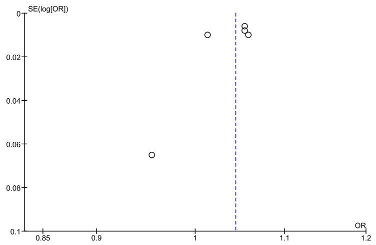

rs3993105

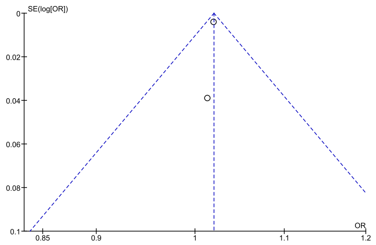

rs41272114

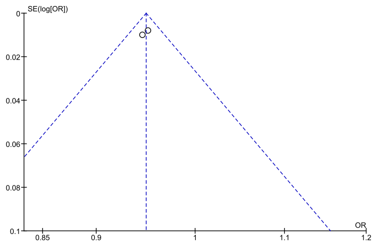

rs421329

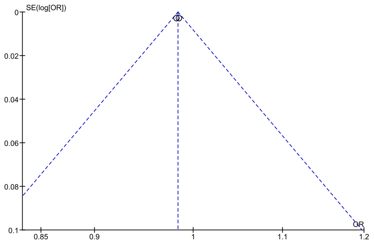

rs4244285

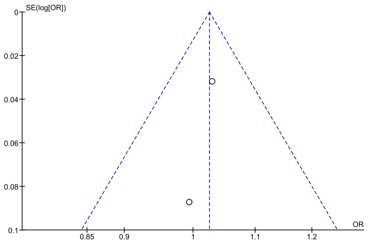

rs425105

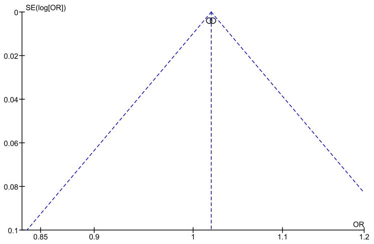

rs4252120

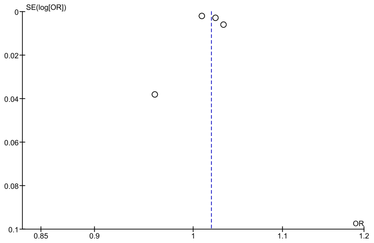

rs4252185

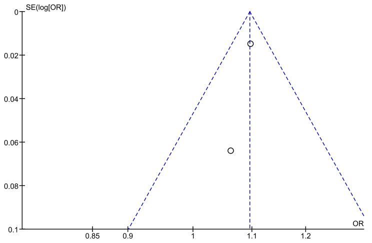

rs4299376

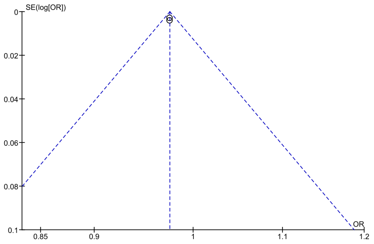

rs4380028

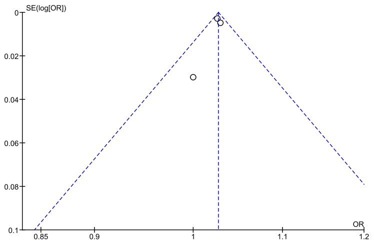

rs4420638

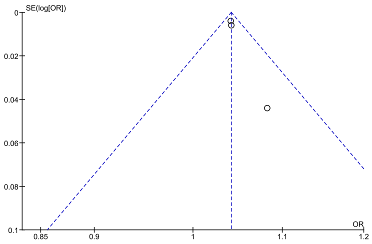

rs445925

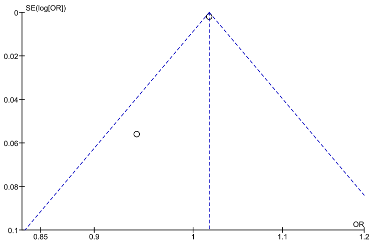

rs46522

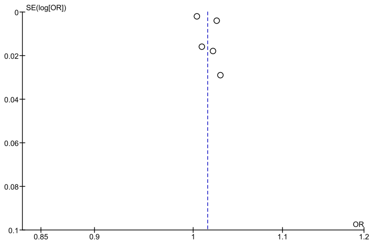

rs4773141

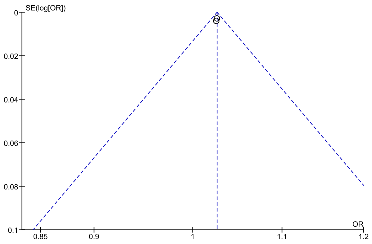

rs4773144

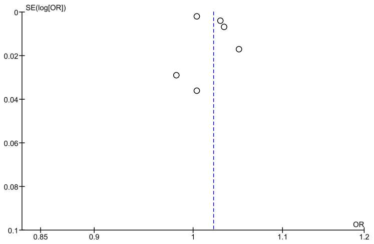

rs4845625

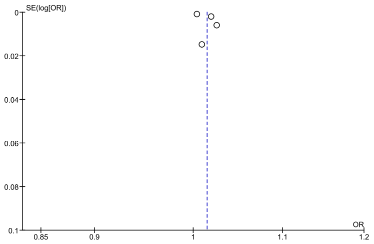

rs4977574

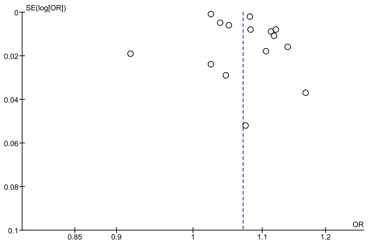

rs501120

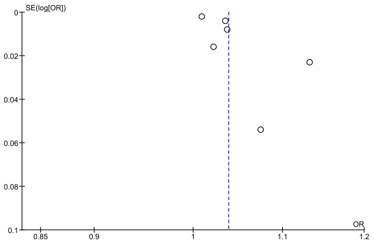

rs507666

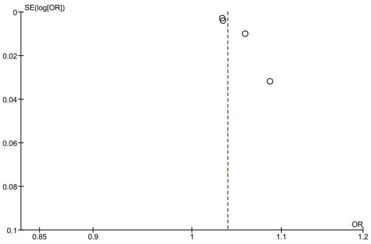

rs514659

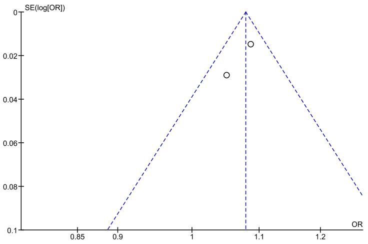

rs515135

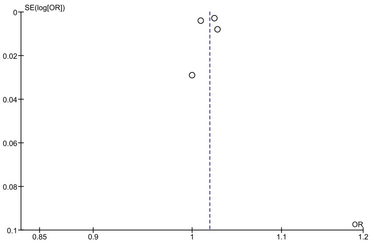

rs518394

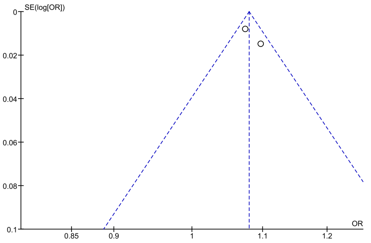

rs523096

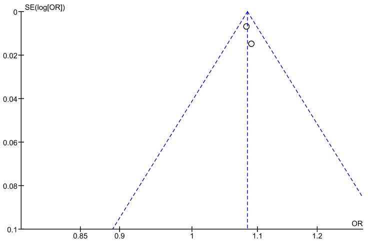

rs55730499

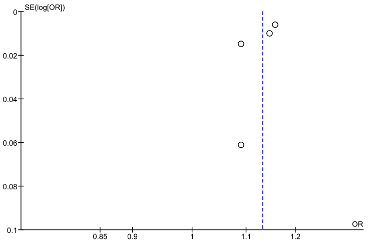

rs55940034

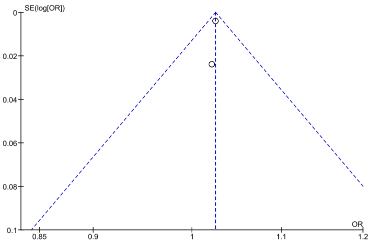

rs56062135

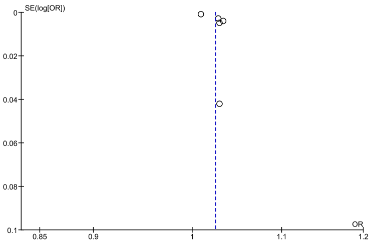

rs56131196

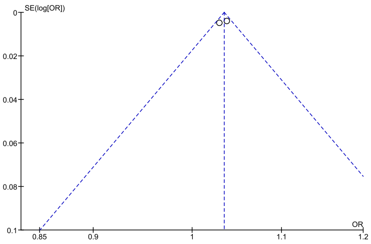

rs56210063

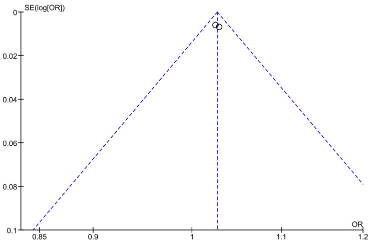

rs56289821

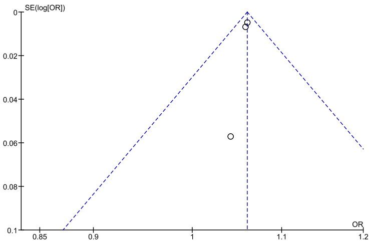

rs56313611

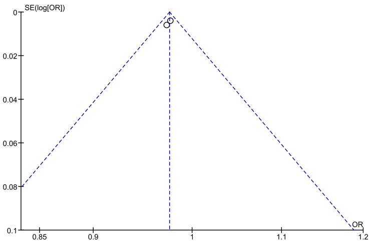

rs56336142

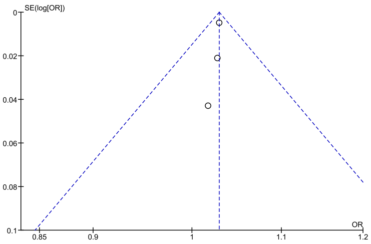

rs56393506

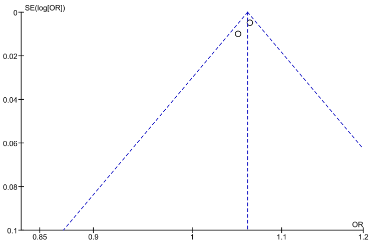

rs564398

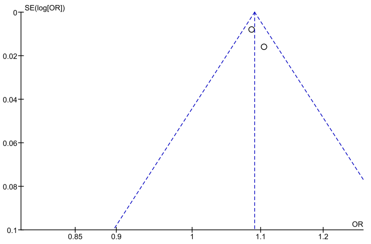

rs571353

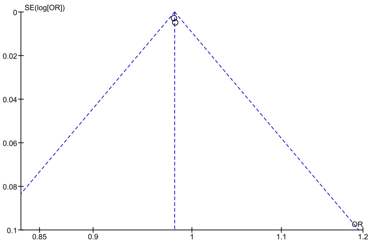

rs579459

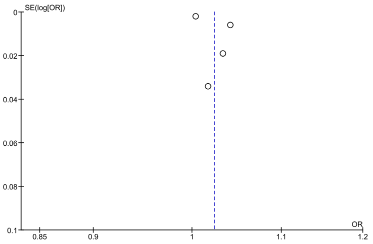

rs590121

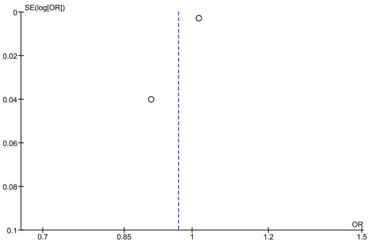

rs59898454

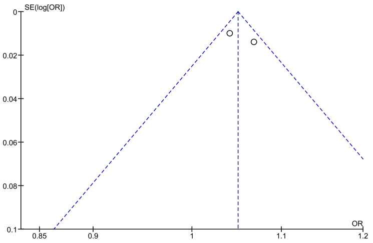

rs599839

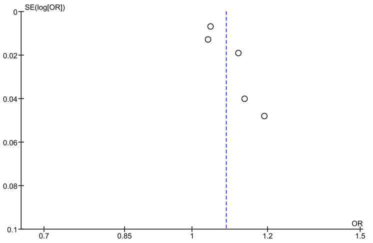

rs602633

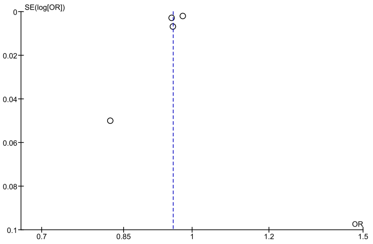

rs6129767

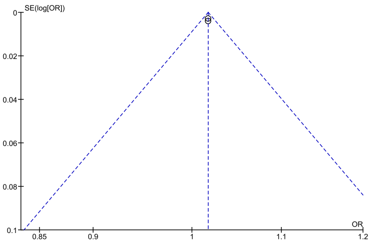

rs61734696

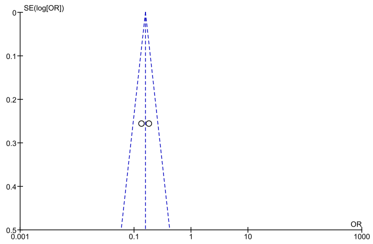

rs62076439

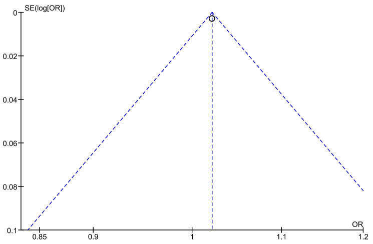

rs62253653

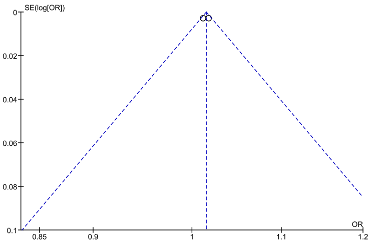

rs629301

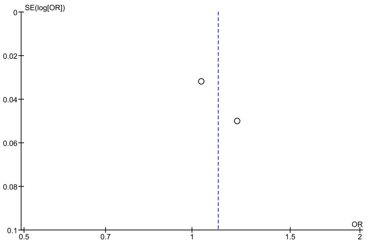

rs634552

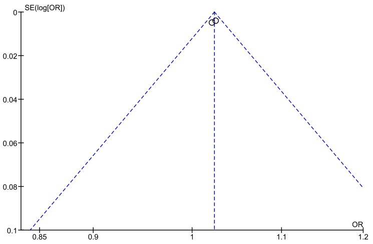

rs643319

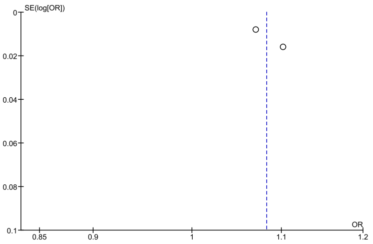

rs643434

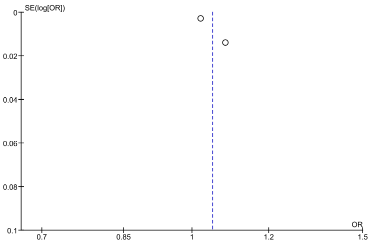

rs646776

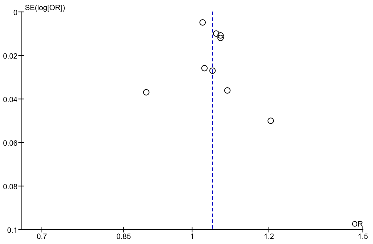

rs6475606

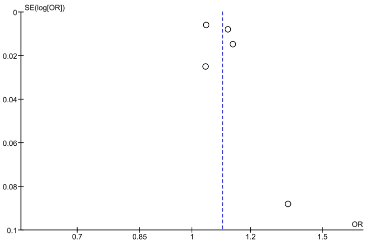

rs6494488

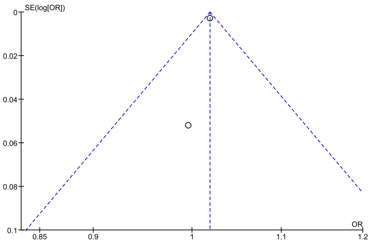

rs6504218

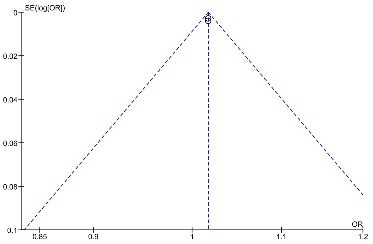

rs6511720

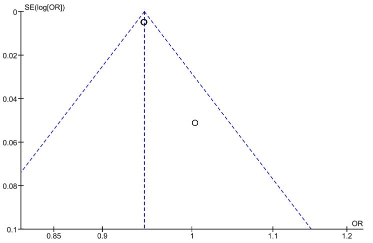

rs6538176

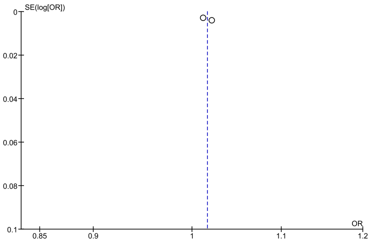

rs6544713

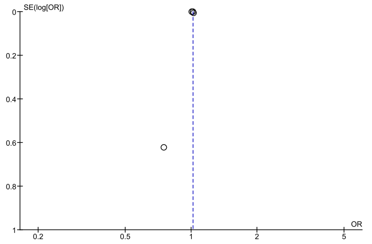

rs657152

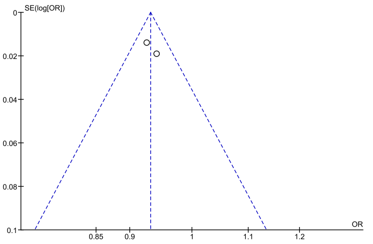

rs663129

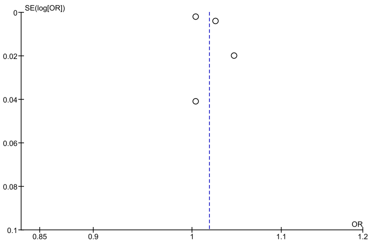

rs6689306

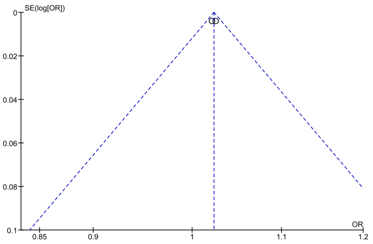

rs6700559

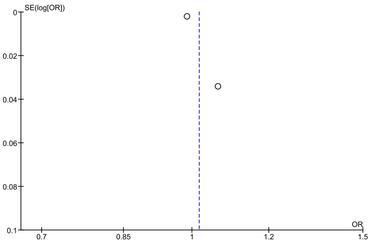

rs671

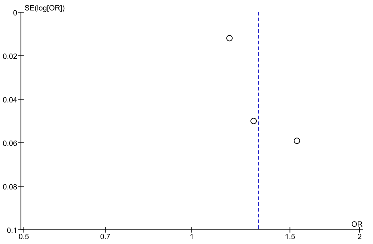

rs67180937

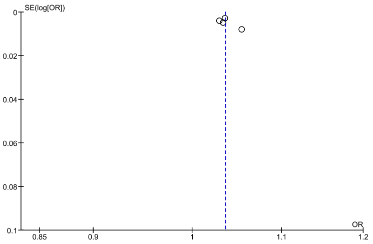

rs6725887

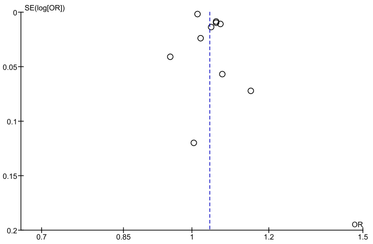

rs68170813

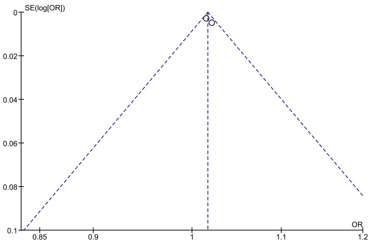

rs6841581

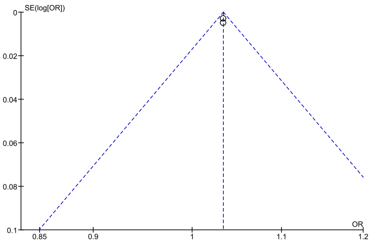

rs6842241

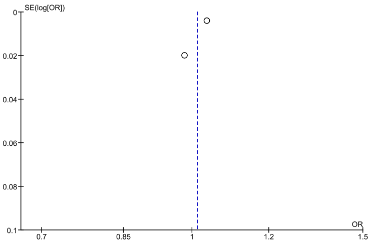

rs688034

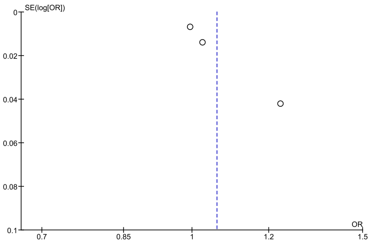

rs6903956

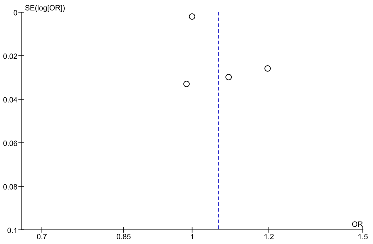

rs6905288

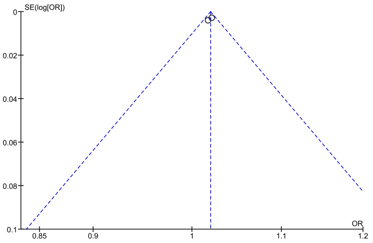

rs6909752

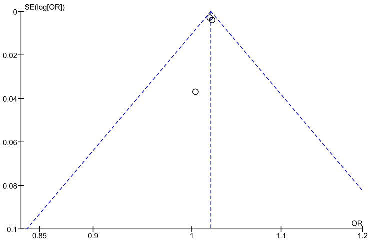

rs6922269

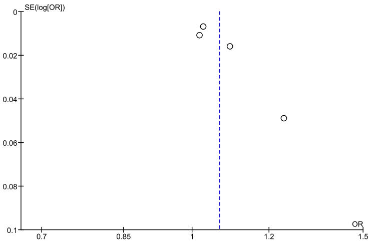

rs7044859

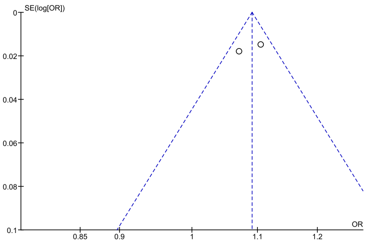

rs7136259

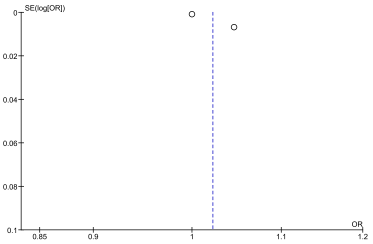

rs7173743

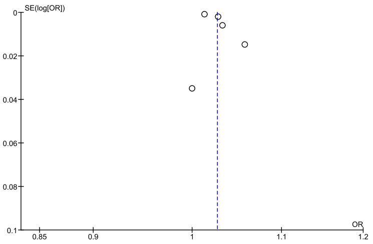

rs7212798

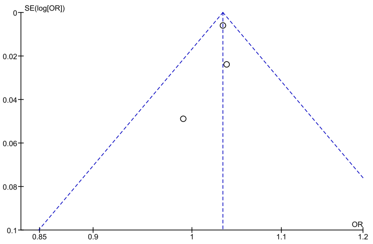

rs7214245

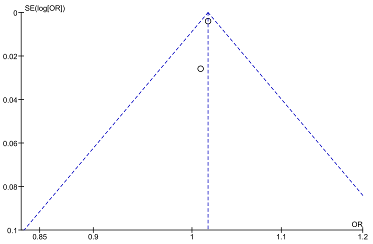

rs7250581

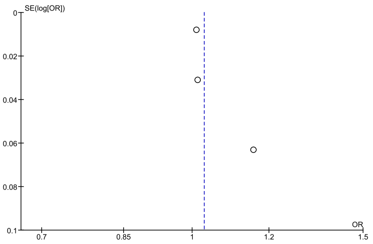

rs72689147

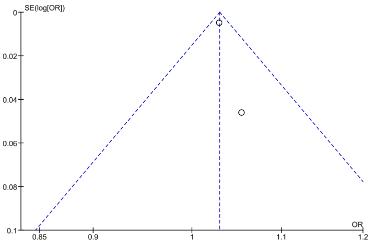

rs72743461

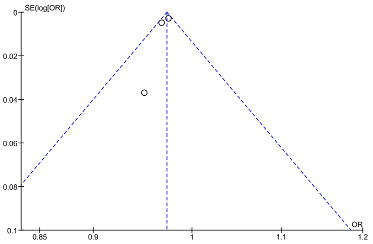

rs73045269

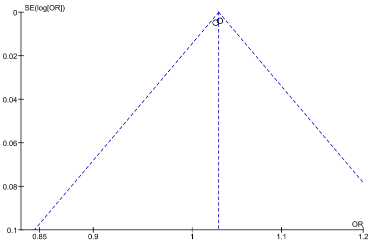

rs7412

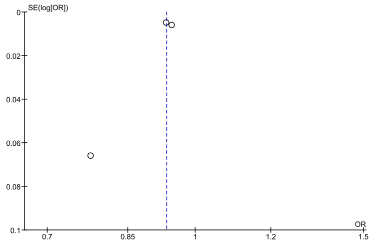

rs742115

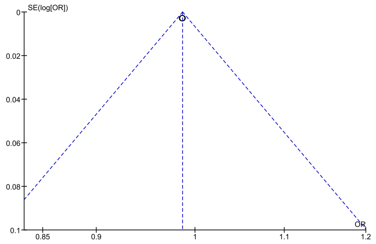

rs74416240

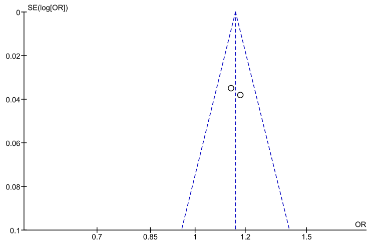

rs74617384

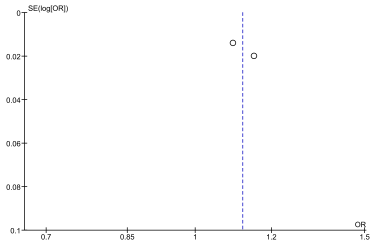

rs748431

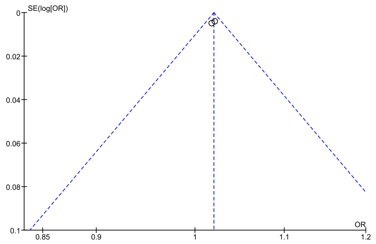

rs7500448

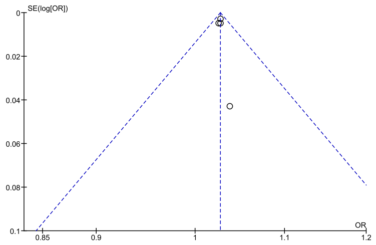

rs7528419

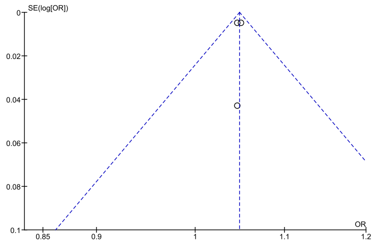

rs7538207

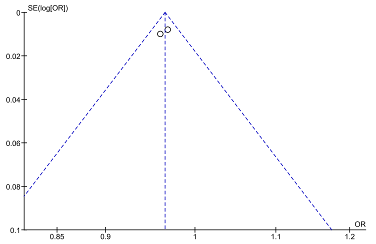

rs7568458

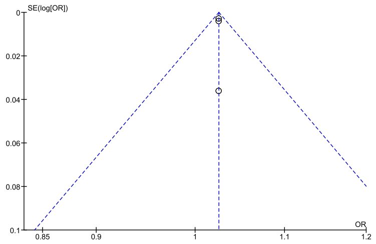

rs7623687

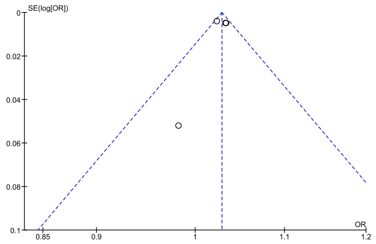

rs7678555

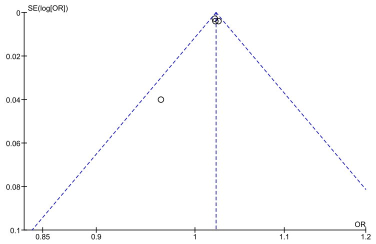

rs7692387

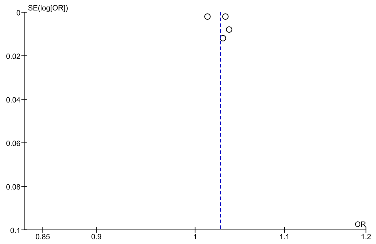

rs781622

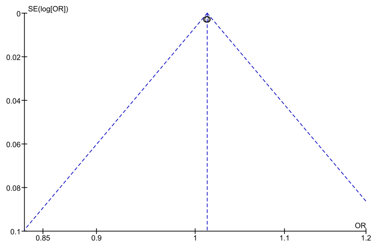

rs7865618

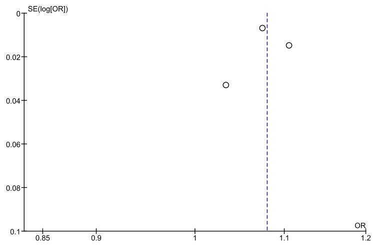

rs7969300

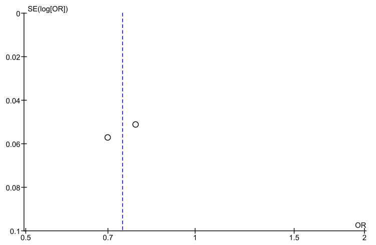

rs79716828

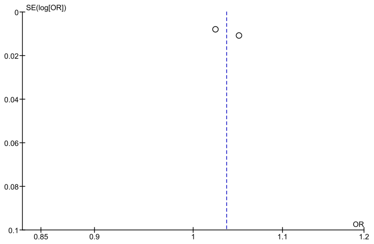

rs8042271

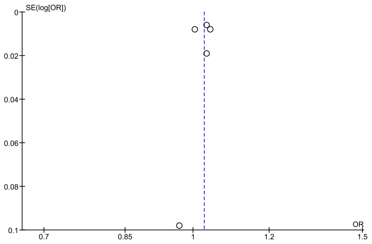

rs8055236

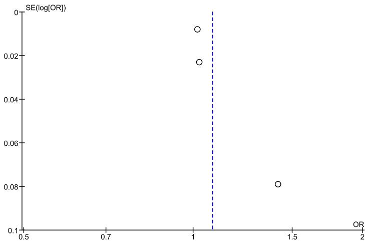

rs8108632

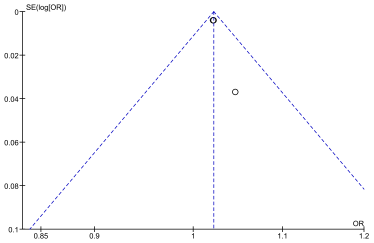

rs833509

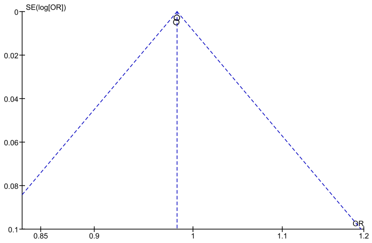

rs867186

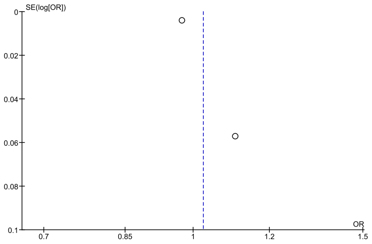

rs886126

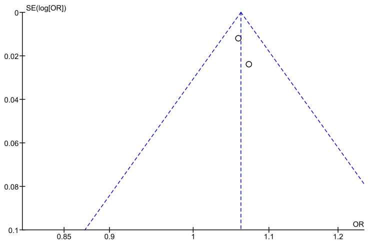

rs916524

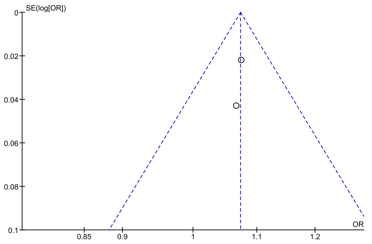

rs918949

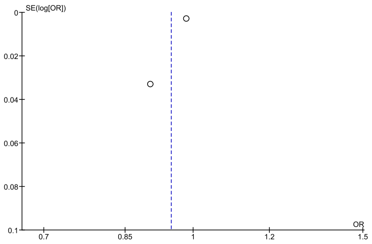

rs925368

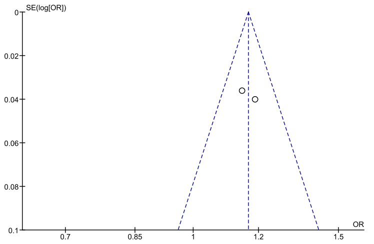

rs9319428

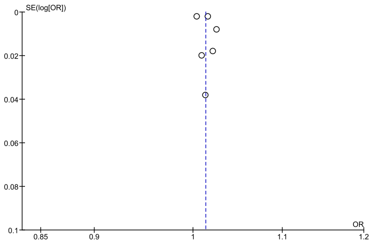

rs9349379

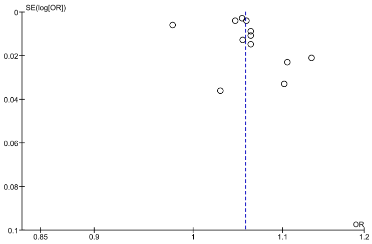

rs9369640

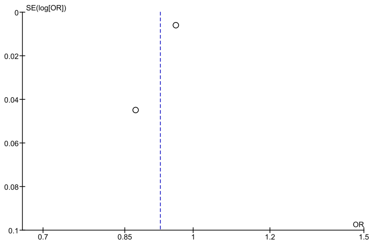

rs944797

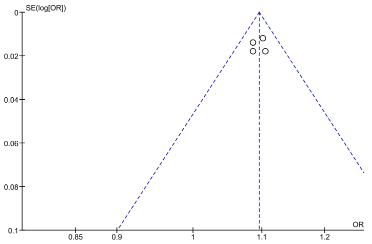

rs9501744

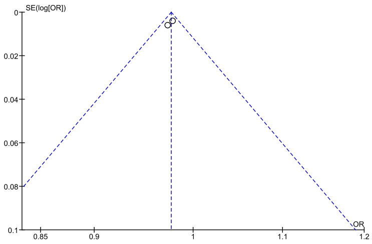

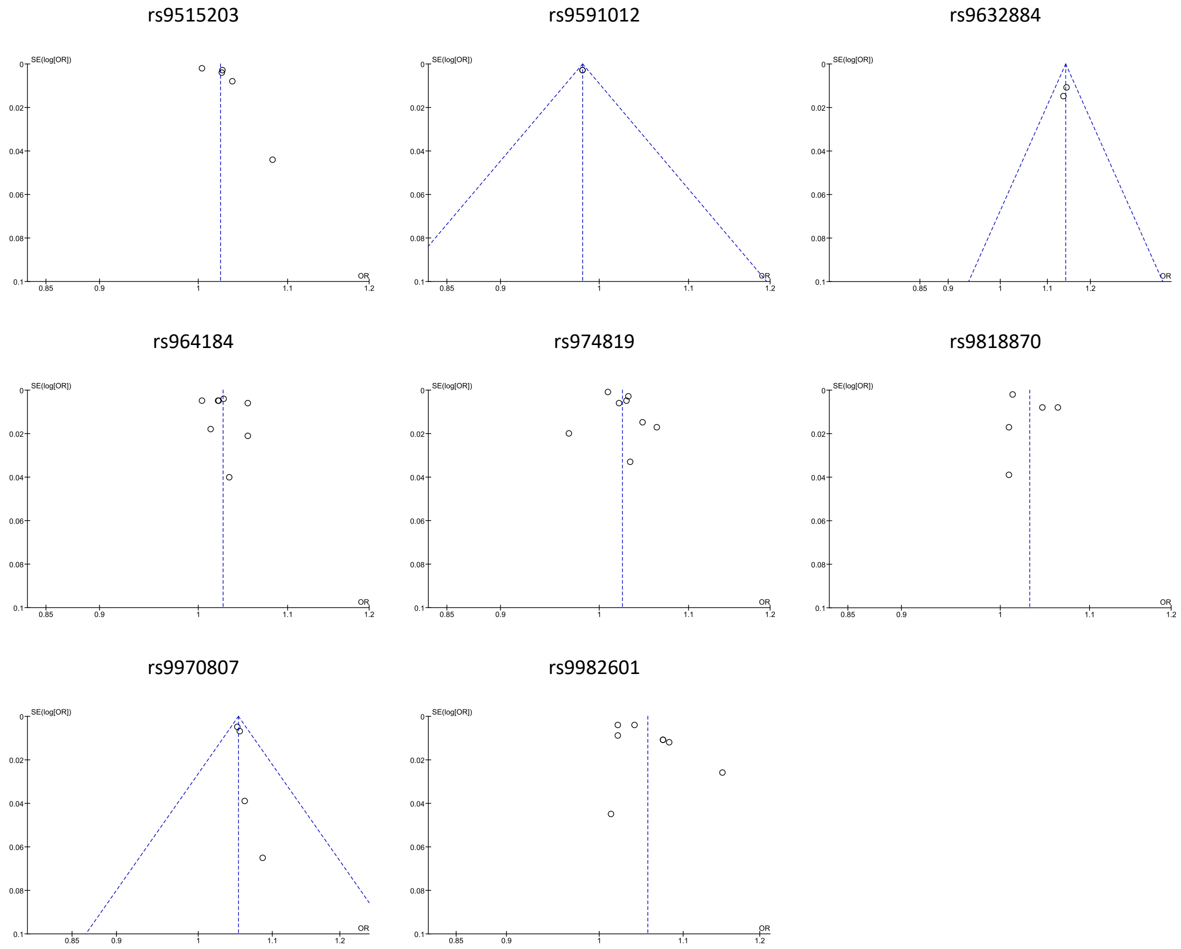

Figure S3. Funnel plots for all single nucleotide polymorphisms from 44 GWAS publications included in this paper except for rs4593108.
