# Supplementary material for: Genetic Factors for Coronary Heart Disease and Their Mechanisms: A Meta-Analysis and Comprehensive Review of Common Variants from Genome-Wide Association Studies
Source: Diagnostics (Basel). 2022 Oct 21;12(10):2561. doi: 10.3390/diagnostics12102561 (PMC9601486; doi:10.3390/diagnostics12102561)
Supplement: Supplementary file 1 [file diagnostics-12-02561-s001.zip › PRISMA 2020 Abstract Checklist.pdf]

PRISMA 2020 for Abstracts Checklist

Section and Topic	Item #	Checklist item	Reported (Yes/No)
**TITLE**			
Title	1	Identify the report as a systematic review.	Manuscript title
**BACKGROUND**			
Objectives	2	Provide an explicit statement of the main objective(s) or question(s) the review addresses.	Lines 18-21
**METHODS**			
Eligibility criteria	3	Specify the inclusion and exclusion criteria for the review.	Not mentioned in abstract due to word limitation
Information sources	4	Specify the information sources (e.g. databases, registers) used to identify studies and the date when each was last searched.	Lines 21-24
Risk of bias	5	Specify the methods used to assess risk of bias in the included studies.	Not mentioned in abstract due to word limitation
Synthesis of results	6	Specify the methods used to present and synthesise results.	Lines 21-24
**RESULTS**			
Included studies	7	Give the total number of included studies and participants and summarise relevant characteristics of studies.	Not mentioned in abstract due to word limitation
Synthesis of results	8	Present results for main outcomes, preferably indicating the number of included studies and participants for each. If meta-analysis was done, report the summary estimate and confidence/credible interval. If comparing groups, indicate the direction of the effect (i.e. which group is favoured).	Lines 24-31
**DISCUSSION**			
Limitations of evidence	9	Provide a brief summary of the limitations of the evidence included in the review (e.g. study risk of bias, inconsistency and imprecision).	Not mentioned in abstract due to word limitation
Interpretation	10	Provide a general interpretation of the results and important implications.	Lines 31-34
**OTHER**			
Funding	11	Specify the primary source of funding for the review.	Not mentioned in abstract due to word limitation
Registration	12	Provide the register name and registration number.	Not registered

From: Page MJ, McKenzie JE, Bossuyt PM, Boutron I, Hoffmann TC, Mulrow CD, et al. The PRISMA 2020 statement: an updated guideline for reporting systematic reviews. BMJ 2021;372:n71. doi: 10.1136/bmj.n71

For more information, visit: http://www.prisma-statement.org/
